# A Pediatric Emergency Medicine Refresher Course for Generalist Healthcare Providers in Belize: Respiratory Emergencies

**DOI:** 10.21980/J84063

**Published:** 2021-04-19

**Authors:** Adeola Adekunbi Kosoko, Alicia E Genisca, Marideth Rus, Shreya Ramayya, Lisa Johnson, Joy Mackey

**Affiliations:** * McGovern Medical School at the University of Texas Health Science Center at Houston, Department of Emergency Medicine, Houston, TX; ^ The Warren Alpert Medical School of Medicine Brown University/Hasbro Children’s Hospital, Departments of Emergency Medicine and Pediatrics, Providence, RI; † Texas Children’s Hospital/Baylor College of Medicine, Section of Emergency Medicine, Houston, TX; ** The Warren Alpert Medical School of Medicine Brown University, Providence, RI; ^^ Karl Heusner Memorial Hospital, Former Director of Medical Services and Trauma Surgery Consultant, Belize City, Belize; †† Henry J.N. Taub Baylor College of Medicine, Department of Emergency Medicine, Houston TX

## Abstract

**Audience and type of curriculum:**

This is a refresher curriculum utilizing multiple methods of education to augment the skills of generalist healthcare providers in low- and middle-income countries (LMICs) in the identification and stabilization of pediatric respiratory emergencies. Our audience of implementation was Belizean generalist providers.

**Length of curriculum:**

Nine hours

**Introduction:**

In the pediatric population, early recognition and stabilization can improve patient outcomes. Compared with many Western systems that rely on specialists and even subspecialists, in many lower-resource settings, generalists provide most emergency medical care. The purpose of this module is to present a curriculum focused on the identification and stabilization of common pediatric respiratory emergencies for general practitioners (physicians and nurses) working in the acute care setting. Our aim is to provide a care framework and refresher training for the management of pediatric respiratory emergencies for providers who may regularly see the acutely ill pediatric patient but who may not have had recent or any extensive teaching in the management of acute pediatric airway management, bronchiolitis, pneumonia, and asthma.

**Educational Goals:**

This curriculum presents a refresher course in recognizing and stabilizing pediatric acute respiratory complaints for generalist healthcare providers practicing in LMICs. Our goal is to implement this curriculum in the small LMIC of Belize. This module focuses on common respiratory complaints, including asthma, bronchiolitis, pneumonia and acute airway management.

**Educational Methods:**

The educational strategies used in this curriculum include didactic lectures, medical simulation, small-group sessions, and a skills lab.

**Research Methods:**

We scored written pretests before and posttests after intervention and retested participants to evaluate for knowledge retention. Participants provided qualitative feedback on the module.

**Results:**

We taught 26 providers. Twenty-one providers completed the posttest and eight completed the retest. The mean test scores improved from 8.3 ± 2.8 in the pretest to 9.7 ± 1.3 to the posttest (mean difference = 1.4; P = 0.027). The mean test score at pretest was 8.0 ± 4.0, which increased to 9.9 ± 2.5 at retest four months later (mean difference = 1.9, P = 0.049). Fifteen (71.4%) participants found the course “extremely useful,” and 28 (28.5%) participants “very useful.”

**Discussion:**

This curriculum is an effective and well-received training tool for Belizean generalist providers. Although limited by sample size and 20% attrition for the retest, there was a statistically significant improvement in test performance. We believe that our pilot in Belize shows that this type of refresher course could be useful for teaching generalist providers in LMICs to optimize care of the acutely ill pediatric patient with respiratory ailment. Evaluation of other modules in this curriculum, application of the curriculum in other locations, and measuring clinical patient outcomes will be included in future investigations.

**Topics:**

Medical simulation, rapid cycle deliberate practice (RCDP), Belize, bronchiolitis, pneumonia, asthma, airway, respiratory distress, low- and middle-income country (LMIC), collaboration, global health.

## USER GUIDE

**Table t3-jetem-6-2-c73:** 

List of Resources:	
Abstract	73
User Guide	75
Didactics and Hands on Curriculum Chart	81
Appendix A: Pre-Test Questions	85
Appendix B: Pre-Test Answers	92
Appendix C: Bronchiolitis Lecture	99
Appendix D: Bronchiolitis Lecture Synopsis	100
Appendix E: Bronchiolitis Small Group Discussion	105
Appendix F: Pneumonia Lecture	110
Appendix G: Pneumonia Lecture Synopsis	111
Appendix H: Pediatric Pneumonia Rapid Cycle Deliberate Practice (RCDP) Case	115
Appendix I: Asthma Lecture	138
Appendix J: Asthma Lecture Synopsis	139
Appendix K: Pediatric Asthma Rapid Cycle Deliberate Practice (RCDP) Case	143
Appendix L: Airway Lecture	164
Appendix M: Airway and Intubation Lecture Synopsis	165
Appendix N: Airway Lab	171
Appendix O: Post-Test Questions	175
Appendix P: Post-Test Answers	181
Appendix Q: Debriefing Techniques	187
Appendix R: Sample Itinerary	188


[Table t3-jetem-6-2-c73]

**Learner Audience:**

Medical Students, Interns, Junior Residents, General Practitioners (physicians, nurses), Physicians Assistants, Nurse Practitioners

**Length of Curriculum:**

The entire course was designed to be presented over about 9–10 hours total. It could be completed over a day, but we divided the course into 2 days.

- There are 2 simulation sessions, each lasting about 45 minutes.
- There is one small group session, having about 45 minutes of discussion.
- There are 4 didactic lectures, each lasting about 1 hour each.
- There is one skills lab that lasts about 1 hour.
- Most participants used about 20–30 minutes to complete each of the written tests.

**Topics:**

Medical simulation, rapid cycle deliberate practice (RCDP), Belize, bronchiolitis, pneumonia, asthma, airway, respiratory distress, low- and middle-income country (LMIC), collaboration, global health.

**Objectives:**

By the end of this course, learners will:

1. Rapidly assess and initiate emergency interventions for a child in respiratory distress.
2. Gain familiarity with respiratory pathophysiology and interventions unique to pediatric populations.
3. Identify diagnostic criteria for asthma, bronchiolitis, and pneumonia in a pediatric patient.
4. Improve communication and teamwork when managing the acutely ill pediatric patient.
5. Physicians will increase proficiency in performing emergency procedures, particularly airway management and intubation. Supporting staff will increase proficiency facilitating these tasks.

Bronchiolitis Lecture and Small Group Objectives:

1. The learner will demonstrate the ability to recognize the clinical presentation of bronchiolitis
2. Apply the recommendations made in the current American Academy of Pediatrics clinical practice guideline for diagnosis and management of bronchiolitis
3. Understand the pathophysiology of bronchiolitis
4. Explain the role of laboratory testing in the diagnosis of bronchiolitis
5. Assess for serious bacterial infections in patients who have bronchiolitis
6. Advise families on the prognosis and risk of recurrent wheezing in patients diagnosed with bronchiolitis

Pneumonia Lecture and Simulation Objectives:

1. Identify pneumonia as a top cause of mortality for children worldwide
2. Identify the common bacterial pathogens that cause pediatric pneumonia
3. Explain the utility of imaging and diagnostic testing indicated for pediatric pneumonia
4. Identify the antibiotic options for the treatment of pediatric pneumonia

Asthma Lecture and Simulation Objectives:

1. Recognize the clinical presentation of acute asthma
2. Understand the pathophysiology of pediatric asthma exacerbations
3. Delineate the efficacy of current therapeutic interventions in the treatment of acute asthma
4. Discuss the role of clinical severity scores in assessing acute asthma and outline a clinical approach/protocol to the treatment of acute asthma

Airway Lecture and Procedure Lab Objectives:

1. Demonstrate the ability to identify indications for intubation
2. Describe the differences of a pediatric airway compared to an adult airway
3. Identify equipment used for airway support
4. List the “P’s” of intubation
5. Recognize complications associated with rapid sequence intubation (RSI) and contraindications to common RSI medications

### Brief introduction

It has been estimated that 80% of deaths in children younger than five years in limited-resource settings are avoidable.^1^ Practitioners in many low- and middle-income countries (LMICs) often do not have distinct training in the care of acutely ill children, or the training is minimal.^2^,^3^ Most physicians in Belize are Belizean nationals trained abroad who have returned home to practice; many are immigrants to Belize. Moreover, Belize, like many other LMICs, does not have clear-cut national guidelines on most care topics and there tends not to be a consensus to which caregivers regularly subscribe. Instead, individual experience, local patterns, guidelines set by other countries, and sometimes, international guidelines, tend to be the basis of patterns of care. However, interventions including triage training, educational initiatives, and use of clinical practice guidelines have been suggested to improve order and patient care. Other studies have shown that targeted multidisciplinary and multicultural team training can be effective in stressful situations.^4^,^5^

This module was developed to teach core pediatric emergency topics to physicians, nurses, and affiliated providers together in one group. Respiratory diseases are a leading cause of death and disability worldwide, and the leading childhood chronic disease worldwide is asthma, affecting 14% of children. In children younger than 5 years, pneumonia is the leading non-traumatic cause of death.^9^

At Karl Heusner Memorial Hospital Authority (KHMHA), the most common pediatric diagnoses are respiratory in nature. Therefore, this module focuses on the diagnoses and management of the most common pediatric respiratory diseases (ie, asthma, bronchiolitis, and pneumonia) and on nonsurgical management of the pediatric airway.

### Problem identification, general and targeted needs assessment

In 2015, we conducted a formal needs assessment of Karl Heusner Memorial Hospital Authority (KHMHA) in Belize City. We identified a desire for improved care for the acutely ill child. In collaboration with administration, we focused on provider education and identified appropriate subject matter based on review of the Accident and Emergency (A&E) Department logbook and discussions with staff and administration.

We decided to use an integrated approach to the curriculum that would incorporate both active and passive learning. Although neither technique is superior, the learners were more familiar with passive learning techniques (eg, reading and didactic lectures).^6^ Simulation-based medical education provides the opportunity to reproducibly practice high-risk scenarios in a safe learning environment. Clinical knowledge, procedural skills, confidence, teamwork, and effective communication practices are fostered in simulation. The rapid cycle deliberate practice (RCDP) format was specifically chosen for this population because of its suitability to those less exposed to learning using medical simulation and to those with the goal of attaining mastery.^5^ RCDP is an instructional method for simulation-based learning that incorporates multiple shorter repetitions of cases with intermixed feedback. It has been useful in improving key performance measures.^6^ Small-group clinical cases help learners think critically rather than depend on memorization, reveal the relevance to clinical practice of the material being taught, and integrate multiple concepts.^7^,^8^ Thus, by revisiting previous content and demonstrating clinical connections, the learning experience is enhanced.

This curriculum was designed based on the 2015 formal needs assessment of the KHMHA A&E and pediatrics departments and on core pediatric emergency competencies from the American Board of Emergency Medicine, the American Academy of Pediatrics, and the care recommendations made by the World Health Organization. The aim is that it can be applied in similar LMICs. It is intended to be an integrated curriculum, with physicians and nurses with various levels of training being the target audience, teaching various health professionals to work collaboratively. This module includes two low-fidelity simulation teaching scenarios using RCDP (asthma and pneumonia), two small-group clinical discussions (bronchiolitis and pneumonia), an airway skills laboratory, and a pre- and post- multiple-choice written test. Due to the lack of participant experience with simulation-based learning and the goal of providing timely feedback, we chose RCDP with opportunities to immediately apply feedback and debriefing for our given scenarios.^7^ The small-group exercises are intended to foster active learning and fill gaps in understanding potentially left by the didactic sessions and simulation exercises. The pretest and posttest consisted of multiple-choice evaluations of the topics covered during the module. This study received institutional review board approval from The University of Texas Health Sciences Center at Houston and Baylor College of Medicine with approval of the KHMHA administration.

### Goals of the curriculum

The goal of this curriculum is to familiarize generalist healthcare providers in LMICs with the identification and stabilization of pediatric respiratory emergencies and management of the pediatric airway.

### Objectives of the curriculum

By the end of this course, learners will:

1. Rapidly assess and initiate emergency interventions for a child in respiratory distress.
2. Gain familiarity with respiratory pathophysiology and interventions unique to pediatric populations.
3. Identify diagnostic criteria for asthma, bronchiolitis, and pneumonia in a pediatric patient.
4. Improve communication and teamwork when managing the acutely ill pediatric patient.
5. Physicians will increase proficiency in performing emergency procedures, particularly airway management and intubation. Supporting staff will increase proficiency facilitating these tasks.

Bronchiolitis Lecture and Small Group Objectives:

1. The learner will demonstrate the ability to recognize the clinical presentation of bronchiolitis
2. Apply the recommendations made in the current American Academy of Pediatrics clinical practice guideline for diagnosis and management of bronchiolitis
3. Understand the pathophysiology of bronchiolitis
4. Explain the role of laboratory testing in the diagnosis of bronchiolitis
5. Assess for serious bacterial infections in patients who have bronchiolitis
6. Advise families on the prognosis and risk of recurrent wheezing in patients diagnosed with bronchiolitis

Pneumonia Lecture and Simulation Objectives:

1. Identify pneumonia as a top cause of mortality for children worldwide
2. Identify the common bacterial pathogens that cause pediatric pneumonia
3. Explain the utility of imaging and diagnostic testing indicated for pediatric pneumonia
4. Identify the antibiotic options for the treatment of pediatric pneumonia

Asthma Lecture and Simulation Objectives:

1. Recognize the clinical presentation of acute asthma
2. Understand the pathophysiology of pediatric asthma exacerbations
3. Delineate the efficacy of current therapeutic interventions in the treatment of acute asthma
4. Discuss the role of clinical severity scores in assessing acute asthma and outline a clinical approach/protocol to the treatment of acute asthma

Airway Lecture and Procedure Lab Objectives:

1. Demonstrate the ability to identify indications for intubation
2. Describe the differences of a pediatric airway compared to an adult airway
3. Identify equipment used for airway support
4. List the “P’s” of intubation
5. Recognize complications associated with rapid sequence intubation (RSI) and contraindications to common RSI medications

### Educational Strategies

(See curriculum chart) Please refer to the curriculum chart of linked objectives and educational strategies.

### Equipment/Environment

The following were required to carry out the module:

- A large room (with a capacity of at least 50 people) with multiple tables and ample floor space, or multiple rooms if available
- A computer and projector setup
- For each group of 3 to 5 learners, one equipment setup includes the following:
  ○ A low-fidelity full-body simulation mannequin. If available, higher-fidelity mannequins can be used (we used a MegaCode Kid, and Laerdal ALS Baby mannequins)
  ○ An intravenous arm task trainer (if the mannequin is not equipped)
  ○ Lower extremity capable of intraosseous (IO) insertion (if mannequin is not equipped)
  ○ A medical resuscitation setup including the standard resuscitation equipment available in the A&E department (intravenous line starter kits, intravenous fluids, IO drill, IO needles, medical tape, bag valve mask, mock medications, etc.)

### Personnel

- One simulation instructor/debriefing facilitator per group of 3 to 6 learners
  ○ This person should be well-versed in the medical theory taught by the simulations presented
  One confederate/assistant per group of 3 to 6 learners

### Results and tips for successful implementation

#### Implementation

This module was conducted at Karl Heusner Memorial Hospital Authority (KHMHA) over 2 days (total of 9 hours). On day 1, multiple-choice pretesting (Appendices A and B), two didactic lectures (Appendices C, D, F, and G), one small-group session (Appendix E), and one simulation scenario (Appendix H) were carried out. The participants were divided into groups of 3 to 6 members depending on the number of facilitators and simulation materials. Each simulation scenario was repeated using Rapid Cycle Deliberate Practice (RCDP) for up to 45 minutes. The participants took turns acting out different roles within each scenario. On day 2, we completed the didactic lectures (Appendices I, J, L, and M), a simulation session (Appendix K), an airway laboratory (Appendix N), and written multiple-choice posttests (Appendices O and P). The elements of the curriculum may be presented to learners in any order, but ideally are presented as topics grouped together and with the lecture as the initial learning modality followed by the same topic’s small group, simulation, or procedure laboratory (Appendix R) (eg, first bronchiolitis lecture followed by bronchiolitis small group).

#### Assessment

Before starting the module, each participant completed the multiple-choice pretest (Appendices A and B). An instructor then gave the first lecture (Appendices C, D, F, G, I, J, L, and M) to the entire class. After this, the participants were randomly assigned into groups of 3 to 6 people, ideally incorporating learners of differing backgrounds and experiences equally into each group. Each group then carried out the corresponding small-group clinical case discussion or the simulation scenario or procedure laboratory matched by topic (Appendices E, H, K, and N). Two topics (ie, asthma and bronchiolitis) were presented on the first day. The other two topics (ie, pneumonia and airway management) were discussed on the second day. After all the topics were covered, a multiple-choice posttest was administered to evaluate the learners (Appendices O and P).

Participants were invited to provide anonymous feedback on the module itself and the instructors.

After 4 months, before another module was offered; providers who were present for the original pretest completed the posttest (Appendices O and P) again to evaluate knowledge retention after the intervention.

#### Quantitative Methods

To measure the impact of training sessions on the knowledge of participants, a paired t test was used to compare pretest and posttest scores. To determine knowledge retention, a paired t test was again used to compare pretest and retest scores taken 4 months after the training module. Results of the hypothesis testing were considered statistically significant at p < 0.05. Stata SE version 15.1 (StataCorp, College Station, TX) was used for all statistical analyses.

#### Qualitative Methods

Open-ended questions were used to elicit feedback from participants regarding improvement of the training. Each entry was reviewed for words or phrases representing one main idea (open codes), and open codes that represent related ideas were categorized into main themes.

#### Debriefing

Simulation sessions were conducted using a method of instruction called rapid cycle deliberate practice (RCDP). In traditional post-scenario debriefing, trainees reflect on the scenario and identify gaps in knowledge and how performance could be improved. However, they have no opportunity to immediately apply the knowledge gained during the debriefing session. RCDP, in comparison, utilizes multiple stopping points during a scenario to provide immediate directed feedback and an opportunity for immediate deliberate practice.^6^ With RCDP, participants are stopped at multiple points during the scenario and feedback on performance is given. They are then able to return to the scenario and immediately apply the knowledge gained. The goal is to obtain mastery. Our RCDP scenarios were developed to have several rounds, with each round increasing in difficulty. Each round had set learning objectives to be discussed before moving on to the next, more difficult round (Appendix Q). Feedback can be given at predetermined stopping points at the end of each round, or with brief pauses within a round if critical actions have been missed or completed exceptionally well. Feedback can include both suggestions for improvement and praise for tasks completely effectively. After feedback, the scenario can be restarted where it was, rewound to a recent major event, or restarted completely. Participants were prebriefed on the expected pauses and format of the sessions prior to commencing simulation sessions.

### Evaluation and Feedback

#### Demographic Characteristics

**Figure 1:**
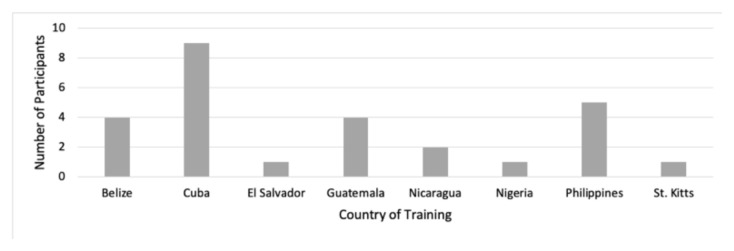
Countries where participants reported being medically trained.

A total of 26 learners participated. There were 14 (58%) women and 12 (32%) men. The participants included 14 physicians, 11 nurses, and one respiratory therapist who were trained in several countries (Figure 1). Most participants (n = 9) were trained in Cuba, all of whom were physicians. Participants had been in practice for a mean of 8.2 years (range, 1–29 years). The mode value of practice was 4 years. Participants were asked to report how comfortable they felt in the management of pediatric patient care in general, on a scale of 1 (“extremely uncomfortable”) through 5 (“extremely comfortable”). The mean rating among participants prior to the intervention was 3.56 (range, 2–5).

#### Test Scores

Participants were required to complete a 20-question multiple-choice test before (pretest) and after (posttest) the training to determine their baseline knowledge and the impact of the curriculum on their knowledge. A total of 21 providers completed both the pretest and the posttest (Table 1). At baseline (pretest), the mean (SD) test score was 8.3 (2.9). After the training (posttest), the mean (SD) test score increased to 9.7 (1.3) (mean difference = 1.4; P = 0.027).

To indirectly assess knowledge retention, the impact of the curriculum on participants’ knowledge four months after training was determined via a scored test (retest) that was compared with the pretest. Eight providers participated in both the pretest and retest (Table 1). At baseline (pretest), the mean (SD) test score was 8.0 (4.0). Four months after training (retest), the mean (SD) test score increased significantly to 9.9 (2.5) (mean difference = 1.9, P = 0.049).

#### Participant Evaluation

The participants reported on how useful they found the training; it was rated highly on a scale of 1 (“not useful”) through 5 (“extremely useful”). The median rating among participants was 5.0 (range, 4–5).

In addition, for participant feedback on improvement of the training, common ideas (codes) in participants’ comments were combined into thematic categories. There were 15 open codes identified from the open-ended questions. These open codes were organized into four thematic categories: “Advance Course Organization,” “Increase Access to Study Material,” “Improve Simulation Authenticity,” and “Enhance Recruitment.” Table 2 presents the details of open codes and themes.

In Belize, like most LMICs, most pediatric and emergency patient care is provided by general practitioners. In response to an identified need to improve care of the acutely ill pediatric patient, we created a curriculum to equip general practitioners with the knowledge, skills, and a framework building on remote lessons in the management of the child with acute respiratory disease. This curriculum is unique in is multimodal approach and interdisciplinary inclusion.

This study shows that this curriculum format and content are appropriate, effective, and a welcome means of teaching relevant concepts. Overall, there were significant improvements in test scores demonstrating that not only did participants learn, but they also retained the knowledge several months later when retested. Additionally, participants valued the module, rating it “extremely useful.” Furthermore, this module has minimal associated costs for implementation and can be presented as a short course (nine to ten hours over 2 days) requiring a nominal time commitment from instructors and learners. This is helpful when considering the staffing needs and educational leave time available for an A&E department.

A limitation of this study is the small numbers of participants. Due to schedule constraints as well as high turnover in staff, we were able to provide this module to only hospital staff available at the time of the course offering. A larger number from the hospital as well as local healthcare facilities would improve the power of the study to show the effect of improved test scores. An additional limitation of this study is that it was designed based on the needs assessment for one hospital in Belize. Although the development of this curriculum was specifically based on the needs that we determined in the largest healthcare center in Belize (ie, KHMHA), we found that the resources and structure of KHMHA are not dissimilar to other LMICs.^3^ We strongly believe that with only minor adaptations, this curriculum would be useful in other LMICs, particularly for providers in the same geographic region who regularly care children with acute respiratory disease.

Valuable feedback for improvement was provided by participants and have already been implemented in the subsequent modules. Some other suggestions for improvement were outside the scope of this course but can be targeted for future interventions. At the request of participants, time was made available each day for those who wanted to practice interacting and gaining familiarity with the manikins. The curriculum was designed to be taught at minimal cost; because of their high costs, we were unable to purchase advanced audio/visual materials or higher-fidelity systems. The KHMHA administration graciously supported the curriculum through providing breakfast and refreshments in subsequent modules. We deliberately did not review testing results with each participant in order to minimize interference with subsequent testing. Outside of evaluating this pilot curriculum, however, there would have been no barrier to sharing and distributing test results to the learners.

Future efforts will be needed to evaluate for the application of concepts taught in the module as it applies to actual patient care. Moreover, as another measure of this curriculum’s efficacy, we would like to evaluate the clinical practice of those who participated in the curriculum compared with that of those who did not. Additionally, we would like to target other regional hospitals and hospitals in other LMICs for training.

This module enhances the performance of generalist practitioners without altering the scope of their practice and provides a refresher for core pediatric respiratory and airway skills and concepts. This curriculum offers a framework for variably trained providers in disseminating practical knowledge and standardizing clinical procedures, which, we anticipate, when applied, will improve medical care and patient outcomes for pediatric patients.

### Associated Content

- Instructors can learn more about teaching rapid cycle deliberate practice using this video.
- Debrief2Learn. Rapid Cycle Deliberate Practice [Video]. YouTube. https://youtu.be/yAhZ8HHtTaI. Published November 12, 2016.

### Appendices

- Pretest Questions.docx
- Pretest Answers.docx
- Bronchiolitis Lecture.pptx
- Bronchiolitis Lecture Synopsis.docx
- Bronchiolitis Small Group.docx
- Pneumonia Lecture.pptx
- Pneumonia Lecture Synopsis.docx
- Pneumonia Simulation.docx
- Asthma Lecture.pptx
- Asthma Lecture Synopsis.docx
- Asthma Simulation.docx
- Airway Lecture.pptx
- Airway Lecture Synopsis.docx
- Airway Lab.docx
- Posttest Questions.docx
- Posttest Answers.docx
- Debriefing Techniques.docx
- Example Itinerary.pptx

## Figures and Tables

**Table t4-jetem-6-2-c73:** 

Topic	Recommended Educational Strategy	Educational Content	Objectives	Learners	Timing, Resources Needed (Space, Instructors, Equipment, Citations of JETem pubs or other literature)	Recommended Assessment, Milestones Addressed
Pediatric Airway	Brief lecture describing the management of the pediatric airway Hands-on procedure lab immediately following lecture For a full description of this session, see Kosoko A, et al. A Pediatric Emergency Medicine Refresher Course for Generalist Healthcare Providers: Respiratory Emergencies; JETem 2021.	Acute management of the pediatric airway	The learner will demonstrate the ability to identify indications for intubation, describe the differences of a pediatric airway compared to an adult airway, identify equipment used for airway support, list the “P’s” of intubation and recognize complications associated with rapid sequence induction (RSI) and contraindications to common RSI medications.	Generalist physician and nurses	Lecture 60 minutes 1 instructor Equipment: PowerPoint capable computer, projector, screen Procedure Lab 60 minutes 1 instructor per 3–5 learners Equipment: - A large room with multiple tables and ample floor space, or multiple rooms if available For each group: - 1 full medical simulation mannequin or bust capable of airway manipulation (child or infant size) - Oral pharyngeal airways of different sizes - Nasopharyngeal airways of different sizes - Bag valve mask with masks of different sizes - Mannequin safe lubricant	Milestone: PC1, PC2, PC3, PC4, PC5, PC6, PC7, PC10, MK, ICS1, ICS2 Assessment: Lecture: Module posttest Procedure Lab: In-person feedback given in real-time
Pediatric Asthma	Brief lecture describing the diagnosis and management of the child with an asthma exacerbation Simulation session immediately following lecture For a full description of this session, see Kosoko A, et al. A Pediatric Emergency Medicine Refresher Course for Generalist Healthcare Providers: Respiratory Emergencies; JETem 2021.	Diagnosis, management and disposition of the child with an acute asthma exacerbation	The learner will demonstrate the ability to recognize the clinical presentation of acute asthma, understand the pathophysiology of pediatric asthma exacerbations, delineate the efficacy of current therapeutic interventions in the treatment of acute asthma, discuss the role of clinical severity scores in assessing acute asthma and outline a clinical approach/protocol to the treatment of acute asthma.	Generalist physician and nurses	Lecture 60 minutes 1 instructor Equipment: PowerPoint capable computer, projector, screen Simulation Session 45 minutes (simulation session) 1 instructor per 5–6 learners Equipment: A large room with multiple tables and ample floor space, or multiple rooms if available	Milestone: PC1, PC2, PC3, PC4, PC5, PC6, PC7, MK, ICS1, ICS2 Assessment: Lecture: Module posttest Simulation: In-person feedback given in real-time
Pediatric Bronchiolitis	Brief lecture describing the diagnosis and management of the child with bronchiolitis Small group discussion session immediately following lecture For a full description of this session, see Kosoko A, et al. A Pediatric Emergency Medicine Refresher Course for Generalist Healthcare Providers: Respiratory Emergencies; JETem 2021.	Diagnosis, management, and disposition of the child with bronchiolitis	The learner will demonstrate the ability to recognize the clinical presentation of bronchiolitis, apply the recommendations made in the current American Academy of Pediatrics clinical practice guideline for diagnosis and management of bronchiolitis, understand the pathophysiology of bronchiolitis, explain the role of laboratory testing in the diagnosis of bronchiolitis, assess for serious bacterial infections in patients who have bronchiolitis, and advise families on the prognosis and risk of recurrent wheezing in patients diagnosed with bronchiolitis.	Generalist physician and nurses	Lecture 60 minutes 1 instructor Equipment: PowerPoint capable computer, projector, screen Small Group Discussion 45 minutes 1 instructor per 3–5 learners Equipment: A large room with multiple tables and ample floor space, or multiple rooms if available	Milestone: PC1, PC2, PC3, PC4, PC5, PC6, PC7, MK, ICS1, ICS2 Assessment: Lecture: Module posttest Small Group Discussion: In-person feedback in real time
Pediatric Pneumonia	Brief lecture describing the diagnosis and management of the child with pneumonia Simulation session immediately following lecture For a full description of this session, see Kosoko A, et al. A Pediatric Emergency Medicine Refresher Course for Generalist Healthcare Providers: Respiratory Emergencies; JETem 2021.	Diagnosis, management and disposition of the child with pneumonia	The learner will demonstrate the ability to identify pneumonia as a top cause of mortality for children worldwide, identify the common bacterial pathogens that cause pediatric pneumonia, explain the utility of imaging and diagnostic testing indicated for pediatric pneumonia, and identify the antibiotic options for the treatment of pediatric pneumonia.	Generalist physician and nurses	Lecture 60 minutes 1 instructor Equipment: PowerPoint capable computer, projector, screen Simulation 45 minutes 1 instructor per 5–6 learners Equipment: A large room with multiple tables and ample floor space, or multiple rooms if available	Milestone: PC1, PC2, PC3, PC4, PC5, PC6, PC7, MK, ICS1, ICS2 Assessment: Lecture: Module posttest Simulation: Inperson feedback given in real-time

**Table 1:** Participants’ test scores

Testing	Pretest Mean	Pretest SD	Posttest Mean	Posttest SD	Mean Difference	SE	P Value	N
Pretest vs. posttest	**8.3**	**2.9**	**9.7**	**1.3**	**1.4**	**0.6**	0.027	21
Pretest vs. retest	**8**	**4**	**9.9**	**2.5**	**1.9**	**0.8**	0.049	8

**Table 2:** Participant feedback for training improvement

Open Codes	Themes	Included Open Codes
Split groupings	Advance course organization	Split groupings, add workstations, improve audio, add video simulations, include breakfast and lunch, include male instructors
Printed lecture material		
Add workstations		
Location-relevant medications and doses		
Real case studies		
Broader recruitment		
Use more realistic monitors and mannequins	Increase access to study material	Printed lecture material, provide lecture material after pretest
Improve audio		
Add video simulations		
Increase frequency of sessions		
Increase frequency of training to biannual		
Include breakfast and lunch	Improve simulation authenticity	Location-relevant medications and doses, real case studies, use more realistic monitors and mannequins, add example scenario
Provide lecture material after pretest		
Include male instructors		
Add example scenario		
	Enhance recruitment	Broader recruitment, increase frequency of sessions, increase frequency of training to biannual

**Figure f2-jetem-6-2-c73:**
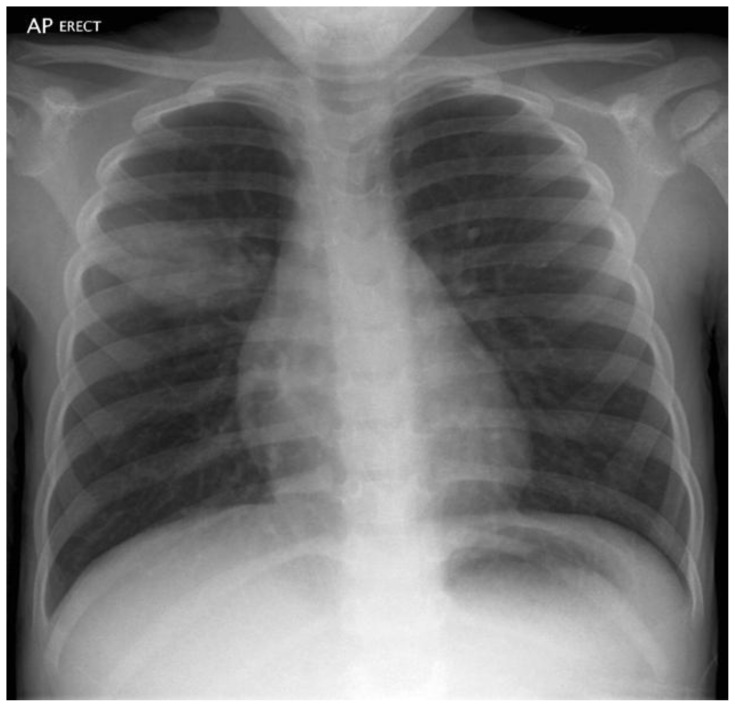
http://radiopaedia.org/articles/round-pneumonia-1

**Figure f3-jetem-6-2-c73:**
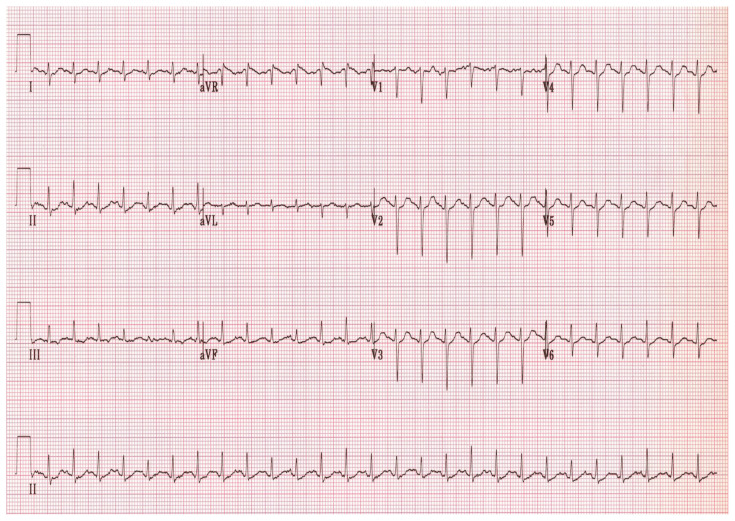
http://lifeinthefastlane.com/ecg-library/

## DIDACTICS AND HANDS-ON CURRICULUM

[Table t4-jetem-6-2-c73]

## Appendix A Pre-Test Questions

1. A 4-month-old female with a history of prematurity presents to the ED with cough, nasal congestion, and fever of the past 3 days. The infant’s respiratory rate is 80, severe chest indrawing (retractions) and diffuse crackles on lung exam. She is otherwise ill in appearance.
  What should be the providers’ first step?
  - Albuterol inhaler
  - Oral corticosteroids
  - Perform rapid sequence intubation
  - Supplemental oxygen
  - Epinephrine injection
2. Which of the following statements regarding the pediatric airway as compared with the adult airway is true?
  - A child has a smaller tongue relative to the size of the oral cavity
  - An infant’s epiglottis is relatively short and thicker
  - In children younger than 10 years, the narrowest portion of the airway is below the vocal cords
  - The vocal cords in infants are omega-shaped
3. An 8-month-old infant born at term is brought in for cough, rhinorrhea, and congestion for three days. Tmax at home was 101 °F. Her past medical history and birth history are unremarkable. Vital signs are T 100.1 °°F, HR 129, RR 42, BP 90/60, and SpO2 98% on room air. The patient is smiling and active and tachypneic. There is scant wheezing and mild retractions.
  What is true regarding this patient’s condition?
  - Albuterol therapy has been shown to reduce 30-day mortality
  - Corticosteroids reduce the hospitalization rates
  - Respiratory syncytial virus (RSV) is the most common cause
  - Ribavirin is indicated
  - The patient has a 75% chance of developing asthma as a child
4. Which of the following statements regarding pneumonia in children is true?
  - Blood cultures frequently reveal the cause of pneumonia in children
  - Cough is the best single predictor of pneumonia
  - Cough is the most prominent symptom in neonates with pneumonia
  - Dehydration is the most common systemic complication
  - Mycoplasma pneumoniae is the most common cause of bacterial pneumonia
5. Which of the following is true regarding selection of agents for rapid sequence intubation (RSI)?
  - Ketamine is known to be safely used in a patient with a ruptured globe.
  - Neuromuscular blocking agents are contraindicated in patients who are having seizures.
  - Rocuronium at a dose of 1–1.2 mg/kg achieves similar intubating conditions as succinylcholine 1.5–2mg/kg for emergent RSI in children.
  - Succinylcholine would be recommended for use in a child with glomerulonephritis and the following labs (mEq/L): Na 138, K 5.8, Cl 101, HCO3 17.
6. Regarding the use of corticosteroids in asthma management, which statement is true?
  - IV corticosteroids have been proven more effective than oral corticosteroids
  - Inhaled corticosteroids are not useful for long-term asthma management
  - Long-term systemic corticosteroid use may be complicated by weight gain, aseptic necrosis of long bones, and peptic ulcer disease (PUD)
  - Patients who require steroids upon discharge to home require tapered oral corticosteroids for 10 or more days
  - The onset of action for IV corticosteroids is within 1 hour
7. A mother brings her 6-month-old male infant to your office. She reports that her son has been breathing faster than usual for the past 2 days, and she has noted occasional wheezing. She states that prior to the difficulty breathing, she noticed some clear nasal discharge for several days. The infant was born full-term, with no complications, and no significant medical history. Vital signs are T 38 ° C, BP 100/60 mmHg, HR 120 bpm, RR 40 rpm, SpO2 95%. Physical exam reveals expiratory wheezing and crackles, and intercostal retractions are noted.
  Which of the following is the most appropriate next step in management?
  - CT scan of the chest
  - Inhaled fluticasone
  - Intubation
  - Non-invasive supportive care
  - Oral amoxicillin
8. All the following patients are at high risk for morbidity and mortality from RSV infection EXCEPT:
  - A 1-month old child with an unrepaired congenital heart disease
  - A 2-month old with cerebral palsy
  - A 4-month old who was born at 32 weeks gestational age with bronchopulmonary dysplasia (BPD)
  - A full-term infant with hyperbilirubinemia
  - A premature infant
9. What size cuffed endotracheal tube would you request for a 4-year-old child?
  - 3.5
  - 4
  - 4.5
  - 5
  - 6
10. A previously healthy 3-year-old male presents to the A&E for evaluation of fever and cough for 3 days. He has a decreased appetite but taking fluids and does not appear dehydrated. In triage, his T 39.5 °C, HR 120, RR 36, SpO2 96% on room air. On exam, he is alert and ambulatory with no distress in his breathing. Diminished breath sounds are noted in the right base.
  The next appropriate step would be:
  - A call to the pediatrician for admission
  - Immediate CBC, blood culture, and ABG (arterial blood gas)
  - Immediate IV placement for fluids and antibiotics
  - Inspiratory/expiratory radiographs
  - Oral antibiotics and discharge home
11. Which choice would be the most appropriate antibiotic(s) to start for the above case?
  - Amoxicillin
  - Azithromycin
  - Ceftriaxone
  - Oseltamivir
  - Trimethoprim-sulfamethoxazole
12. A 5-year-old girl presents for fever for the past 2 days and now is lethargic and has BP 70/50 and HR 150 at triage. Which may improve perfusion in a patient receiving sedation during RSI for septic shock?
  - Etomidate
  - Ketamine
  - Midazolam
  - Thiopental
13. Which is true regarding the use of ipratropium in asthma management?
  - If a patient responded well to ipratropium for status asthmaticus, he should be discharged to home with a prescription for ipratropium.
  - Ipratropium has never been proven to be of benefit in patients with acute asthma exacerbations
  - Ipratropium is most useful as an adjunct for patients with severe asthma exacerbations
  - Ipratropium is useful by itself as a bronchodilator in the treatment of acute asthma exacerbations
  - The main benefit of ipratropium instead of other anticholinergic drugs is its rapid onset of action
14. A previously healthy 4-year-old girl is brought into the A&E for concern for fever. After taking a complete history and performing a physical exam, you have diagnosed her with community acquired pneumonia. Which of the following does NOT necessitate inpatient admission?
  - Decreased oral intake
  - Incomplete immunization profile
  - Lethargy
  - Oxygen requirement
  - Persistent tachypnea
15. A 3-month-old male (born at 38 weeks gestational age) comes to the emergency room with a history of upper respiratory symptoms for 3 days, fever, decreased appetite and increased work of breathing. On physical exam his vitals are T 99 °F, RR 55, HR 185, BP 90/65 and Sat 96% on room air, and he has nasal congestion, mild retractions, and coarse breath sounds. He has an older sister in primary school. Of the following, the MOST appropriate next step in management is:
  - Give oral corticosteroid
  - Prescription for nasal decongestants
  - Start albuterol
  - Suction
  - Supplementary oxygen
16. Aminophylline (theophylline) is prescribed for a patient with asthma. A nurse administers the medication, knowing that the primary action of this medication is to:
  - Improve PEEP (positive end-expiratory pressure) to the lungs
  - Prevent infection
  - Promote expectoration
  - Reduce pain associated with cough
  - Relax smooth muscles
17. A 12-year-old boy presents with an asthma exacerbation. Over several hours, he is given oral steroids and several nebulized albuterol and ipratropium bromide treatments. Vital signs are P 110, RR 20, O2 saturation 95%, PEFR (peak respiratory flow rate) 250L/min (40% of his normal). He can speak in full sentences but is still wheezing. What should be the next step in his management?
  - Continue albuterol and consider admission
  - Discharge on albuterol and prescription for corticosteroids
  - Give subcutaneous epinephrine and reassess
  - Give subcutaneous terbutaline and admit to the ICU
  - Initiate noninvasive positive pressure
18. Which of the following is most true regarding pediatric community-acquired pneumonia (CAP)?
  - It is easier to differentiate between typical and atypical pneumonia in pediatric patients
  - Streptococcus pneumoniae is the most commonly isolated organism in children aged 5 years to 15 years
  - The incidence of CAP in children younger than 5 years old is higher than in middle-aged adult smokers
  - The most common cause of pneumonia in the neonate is Mycoplasma pneumoniae
19. Which of the following medications is the most effective in the immediate treatment of an acute asthma exacerbation?
  - Inhaled corticosteroids
  - IV ketamine
  - IV magnesium
  - Nebulized albuterol or salbutamol
  - Nebulized saline
20. A 10-year-old girl presents with altered mental status. GCS (Glasgow Coma Scale) is 8 on initial evaluation in the A&E. Please match the rapid sequence intubation (RSI) pretreatment with the correct indication for administration.
  - Atropine – to prevent bradycardia
  - Etomidate – to promote adrenal stimulation
  - Fentanyl – to decrease secretions
  - Lidocaine – to cause dissociation

## Appendix B Pre-Test Answers

1. A 4-month-old female with a history of prematurity presents to the ED with cough, nasal congestion, and fever of the past 3 days. The infant’s respiratory rate is 80, severe chest indrawing (retractions) and diffuse crackles on lung exam. She is otherwise ill in appearance.
  What should be the providers’ first step?
  - Albuterol inhaler
  - Oral corticosteroids
  - Perform rapid sequence intubation
  - **Supplemental oxygen**
  - Epinephrine injection
2. Which of the following statements regarding the pediatric airway as compared with the adult airway is true?
  - A child has a smaller tongue relative to the size of the oral cavity
  - An infant’s epiglottis is relatively short and thicker
  - **In children younger than 10 years, the narrowest portion of the airway is below the vocal cords**
  - The vocal cords in infants are omega-shaped
3. An 8-month-old infant born at term is brought in for cough, rhinorrhea, and congestion for three days. Tmax at home was 101 °F. Her past medical history and birth history are unremarkable. Vital signs are T 100.1 °°F, HR 129, RR 42, BP 90/60, and SpO2 98% on room air. The patient is smiling and active and tachypneic. There is scant wheezing and mild retractions.
  What is true regarding this patient’s condition?
  - Albuterol therapy has been shown to reduce 30-day mortality
  - Corticosteroids reduce the hospitalization rates
  - **Respiratory syncytial virus (RSV) is the most common cause**
  - Ribavirin is indicated
  - The patient has a 75% chance of developing asthma as a child
4. Which of the following statements regarding pneumonia in children is true?
  - Blood cultures frequently reveal the cause of pneumonia in children
  - Cough is the best single predictor of pneumonia
  - Cough is the most prominent symptom in neonates with pneumonia
  - **Dehydration is the most common systemic complication**
  - Mycoplasma pneumoniae is the most common cause of bacterial pneumonia
5. Which of the following is true regarding selection of agents for rapid sequence intubation (RSI)?
  - Ketamine is known to be safely used in a patient with a ruptured globe.
  - Neuromuscular blocking agents are contraindicated in patients who are having seizures.
  - **Rocuronium at a dose of 1–1.2 mg/kg achieves similar intubating conditions as succinylcholine 1.5–2mg/kg for emergent RSI in children.**
  - Succinylcholine would be recommended for use in a child with glomerulonephritis and the following labs (mEq/L): Na 138, K 5.8, Cl 101, HCO3 17.
6. Regarding the use of corticosteroids in asthma management, which statement is true?
  - IV corticosteroids have been proven more effective than oral corticosteroids
  - Inhaled corticosteroids are not useful for long-term asthma management
  - **Long-term systemic corticosteroid use may be complicated by weight gain, aseptic necrosis of long bones, and peptic ulcer disease (PUD)**
  - Patients who require steroids upon discharge to home require tapered oral corticosteroids for 10 or more days
  - The onset of action for IV corticosteroids is within 1 hour
7. A mother brings her 6-month-old male infant to your office. She reports that her son has been breathing faster than usual for the past 2 days, and she has noted occasional wheezing. She states that prior to the difficulty breathing, she noticed some clear nasal discharge for several days. The infant was born full-term, with no complications, and no significant medical history. Vital signs are T 38 ° C, BP 100/60 mmHg, HR 120 bpm, RR 40 rpm, SpO2 95%. Physical exam reveals expiratory wheezing and crackles, and intercostal retractions are noted.
  Which of the following is the most appropriate next step in management?
  - CT scan of the chest
  - Inhaled fluticasone
  - Intubation
  - **Non-invasive supportive care**
  - Oral amoxicillin
8. All the following patients are at high risk for morbidity and mortality from RSV infection EXCEPT:
  - A 1-month old child with an unrepaired congenital heart disease
  - A 2-month old with cerebral palsy
  - A 4-month old who was born at 32 weeks gestational age with bronchopulmonary dysplasia (BPD)
  - **A full-term infant with hyperbilirubinemia**
  - A premature infant
9. What size cuffed endotracheal tube would you request for a 4-year-old child?
  - 3.5
  - 4
  - 4.5
  - **5**
  - 6
10. A previously healthy 3-year-old male presents to the A&E for evaluation of fever and cough for 3 days. He has a decreased appetite but taking fluids and does not appear dehydrated. In triage, his T 39.5 °C, HR 120, RR 36, SpO2 96% on room air. On exam, he is alert and ambulatory with no distress in his breathing. Diminished breath sounds are noted in the right base.
  The next appropriate step would be:
  - A call to the pediatrician for admission
  - Immediate CBC, blood culture, and ABG (arterial blood gas)
  - Immediate IV placement for fluids and antibiotics
  - Inspiratory/expiratory radiographs
  - **Oral antibiotics and discharge home**
11. Which choice would be the most appropriate antibiotic(s) to start for the above case?
  - **Amoxicillin**
  - Azithromycin
  - Ceftriaxone
  - Oseltamivir
  - Trimethoprim-sulfamethoxazole
12. A 5-year-old girl presents for fever for the past 2 days and now is lethargic and has BP 70/50 and HR 150 at triage. Which may improve perfusion in a patient receiving sedation during RSI for septic shock?
  - Etomidate
  - **Ketamine**
  - Midazolam
  - Thiopental
13. Which is true regarding the use of ipratropium in asthma management?
  - If a patient responded well to ipratropium for status asthmaticus, he should be discharged to home with a prescription for ipratropium.
  - Ipratropium has never been proven to be of benefit in patients with acute asthma exacerbations
  - **Ipratropium is most useful as an adjunct for patients with severe asthma exacerbations**
  - Ipratropium is useful by itself as a bronchodilator in the treatment of acute asthma exacerbations
  - The main benefit of ipratropium instead of other anticholinergic drugs is its rapid onset of action
14. A previously healthy 4-year-old girl is brought into the A&E for concern for fever. After taking a complete history and performing a physical exam, you have diagnosed her with community acquired pneumonia. Which of the following does NOT necessitate inpatient admission?
  - Decreased oral intake
  - **Incomplete immunization profile**
  - Lethargy
  - Oxygen requirement
  - Persistent tachypnea
15. A 3-month-old male (born at 38 weeks gestational age) comes to the emergency room with a history of upper respiratory symptoms for 3 days, fever, decreased appetite and increased work of breathing. On physical exam his vitals are T 99 °F, RR 55, HR 185, BP 90/65 and Sat 96% on room air, and he has nasal congestion, mild retractions, and coarse breath sounds. He has an older sister in primary school.
  Of the following, the MOST appropriate next step in management is:
  - Give oral corticosteroid
  - Prescription for nasal decongestants
  - Start albuterol
  - **Suction**
  - Supplementary oxygen
16. Aminophylline (theophylline) is prescribed for a patient with asthma. A nurse administers the medication, knowing that the primary action of this medication is to:
  - Improve PEEP (positive end-expiratory pressure) to the lungs
  - Prevent infection
  - Promote expectoration
  - Reduce pain associated with cough
  - **Relax smooth muscles**
17. A 12-year-old boy presents with an asthma exacerbation. Over several hours, he is given oral steroids and several nebulized albuterol and ipratropium bromide treatments. Vital signs are P 110, RR 20, O2 saturation 95%, PEFR (peak respiratory flow rate) 250L/min (40% of his normal). He can speak in full sentences but is still wheezing. What should be the next step in his management?
  - **Continue albuterol and consider admission**
  - Discharge on albuterol and prescription for corticosteroids
  - Give subcutaneous epinephrine and reassess
  - Give subcutaneous terbutaline and admit to the ICU
  - Initiate noninvasive positive pressure
18. Which of the following is most true regarding pediatric community-acquired pneumonia (CAP)?
  - It is easier to differentiate between typical and atypical pneumonia in pediatric patients
  - **Streptococcus pneumoniae is the most commonly isolated organism in children aged 5 years to 15 years**
  - The incidence of CAP in children younger than 5 years old is higher than in middle-aged adult smokers
  - The most common cause of pneumonia in the neonate is Mycoplasma pneumoniae
19. Which of the following medications is the most effective in the immediate treatment of an acute asthma exacerbation?
  - Inhaled corticosteroids
  - IV ketamine
  - IV magnesium
  - **Nebulized albuterol or salbutamol**
  - Nebulized saline
20. A 10-year-old girl presents with altered mental status. GCS (Glasgow Coma Scale) is 8 on initial evaluation in the A&E. Please match the rapid sequence intubation (RSI) pretreatment with the correct indication for administration.
  - **Atropine – to prevent bradycardia**
  - Etomidate – to promote adrenal stimulation
  - Fentanyl – to decrease secretions
  - Lidocaine – to cause dissociation

## Appendix C Bronchiolitis Lecture

Please see associated PowerPoint file

## Appendix D Bronchiolitis Lecture Synopsis

**Target Audience:**
Generalist healthcare providers in low- and middle-income countries
**Educational Methods:**
PowerPoint didactic lecture
**Time required for implementation:**
About 60 minutes
**Learning Objectives:**

1. Recognize the clinical presentation of bronchiolitis.
2. Apply the recommendations made in the current American Academy of Pediatrics Clinical Practice Guideline for Diagnosis and Management of bronchiolitis.
3. Explain the role of laboratory testing in the diagnosis of bronchiolitis.
4. Explain the efficacy of current therapeutic interventions in the treatment of bronchiolitis.
5. Assess for serious bacterial infections in patients who have bronchiolitis.
6. Advise families on the prognosis and risk of recurrent wheezing in patients diagnosed with bronchiolitis.

**Equipment/Environment:**

- A large room (with a capacity of at least 50 people) with multiple tables and ample floor space, or multiple rooms if available
- A computer with PowerPoint capability and projector setup
- Personnel needed: one lecturer

**Recommended pre-reading for instructor:**

- Ralston SL, Lieberthal AS, Meissner HC, et al. Clinical Practice Guideline: The Diagnosis, Management, and Prevention of Bronchiolitis. 2014;134(5):e1474–e1502. *Pediatrics*. Reference correction: 2015;136(4).

### Lecture Script

**Table t5-jetem-6-2-c73:**

Slide 1	Introduction to topic. Opportunity to review objectives of lecture.
Slide 2	An introduction to a common presentation of a child with bronchiolitis. Read case aloud.
Slide 3	The lecture format is as though a practitioner is having a discussion with concerned parents about the lesser known/understood diagnosis of bronchiolitis. “Parental questions will be in purple and can be proposed to the group.”
Slide 4	A lower respiratory tract infection (LRTI): **THE** most common LRTI in children <2 years old Typically occurs less than 2 years old with peak incidence at 2–6 months Can cause disease up to 5 years old One of the leading causes of hospitalization in infants and young children Accounts for 60% of all lower respiratory tract infections in the first year of life
Slide 5	Bronchiolitis is usually due to viruses. Respiratory syncytial virus (RSV) is the most common cause of bronchiolitis, but not all cases of bronchiolitis are due to RSV.
Slide 6	Higher risk populations for bronchiolitis and complications from bronchiolitis.
Slide 7	Environmental risk factors for exposure to and complications from bronchiolitis.
Slide 8	Bronchiolitis is seasonal: Typically, prevalent in what are considered the Winter months to early Spring. Tropical climates see a predominance of bronchiolitis during the rainy season.
Slide 9	Bronchiolitis is a clinical diagnosis made on history and physical exam. There is no laboratory test or imaging exam that can make the diagnosis of bronchiolitis. Identifying the causative virus is generally not warranted because it rarely alters the treatment or outcomes. Signs and symptoms: Systemic findings: decreased oral intake and low-grade fever are common Respiratory symptoms: cough, wheezing, crackles, tachypnea, retractions, apnea Apnea can be the only clinical sign in infants <6 weeks old
Slide 10	No routine testing is indicated to make the diagnosis of bronchiolitis. A complete blood count would not change management. A blood gas analysis can help evaluate for impending respiratory failure, but a clinical exam can often yield the same conclusion. A chest x-ray (CXR) can help evaluate for pneumonia, effusion, or heart disease if the clinical picture is less clear. Chest x-rays are not routinely indicated. However, if a chest x-ray is obtained, the listed findings would suggest bronchiolitis over other diagnoses.
Slide 11	Pneumonia is a common diagnosis that can be difficult to decipher from bronchiolitis. Features that can point to the alternative diagnosis of pneumonia are fevers >39 °C and unilateral signs on chest exam, a worsening clinical course (if simply providing the supportive management indicated for bronchiolitis).
Slide 12	This study investigated the utility of obtaining radiographs in the setting of acute bronchiolitis. Objective: To determine the proportion of radiographs inconsistent with bronchiolitis in children with typical presentation of bronchiolitis and to compare rates of intended antibiotic therapy before radiography versus those given antibiotics after radiography. Conclusions: Infants with typical bronchiolitis do not need imaging because it is almost always consistent with bronchiolitis. Risk of airspace disease appears particularly low in children with saturation higher than 92% and mild to moderate distress. Schuh S, Lalani A, Allen U, et al. Evaluation of the Utility of Radiography in Acute Bronchiolitis. *The Journal of Pediatrics*. 2007;150(4):429–433.
Slide 13	The American Academy of Pediatrics (AAP) does not routinely recommend obtaining CXRs in uncomplicated cases of bronchiolitis. A CXR should only be obtained when there is high pre-test probability of a complication of bronchiolitis, or an alternate diagnosis, or a patient is ill and requiring critical care. Ralston SL, Lieberthal AS, Meissner HC, et al. Clinical Practice Guideline: The Diagnosis, Management, and Prevention of Bronchiolitis. Pediatrics. 2014;134(5):e1474–e1502. *Pediatrics*. Reference correction: 2015;136(4).
Slide 14	The typical clinical course for bronchiolitis is often predictable: upper respiratory infection (URI) symptoms, then lower respiratory infection (LRI) symptoms, then resolution. We can often predict when the course will get a little worse before it gets better. Deviation from this typical course should raise suspicion for alternative diagnoses or complications.
Slide 15	Indications for hospitalization are usually due to inability for the caregivers to provide necessary care at home, often due to poor feeding and dehydration and/or respiratory distress or failure.
Slide 16	Supportive measures for bronchiolitis are ensuring proper hydration and appropriate pulmonary toilet. Maintain adequate fluid intake with PO, NG, or IV fluids Nasal bulb suctioning as needed to clear nasal obstruction Routine deep suctioning is not recommended Antipyretics are used as a comfort measure The child should be allowed to rest Mechanical ventilation (pressure support or intubation) should be performed if any indication of respiratory failure. Oxygen goal should be >90–92% Mechanical ventilation indicated for hypercarbia or apnea Consider early fluid support due to insensible losses from heavy breathing and decreased intake.
Slide 17	Few therapeutic options exist for the management of bronchiolitis: Chest physiotherapy – does not reduce oxygen need or shorten hospitalization; may increase distress and irritability. Bronchodilators – a subset of children with Hx of pulmonary disease, or reactive airway disease, or atopy with significant wheezing may respond; a trial of albuterol or epinephrine may be appropriate; discontinue if not clearly helpful. Corticosteroids – not recommended if previously healthy child with 1^st^ episode of bronchiolitis and no response to bronchodilators; may help with chronic lung disease or history of recurrent wheezing. Antivirals (oseltamivir, ribavirin) – modest effectiveness and costly; may be useful if confirmed RSV and severe disease, but must be given early in course of illness. Antibiotics – only if there is evidence of concomitant bacterial infection (positive urine culture, acute otitis media, consolidation on CXR). Surfactant – may decrease duration of mechanical ventilation or ICU stay, but not routinely recommended.
Slide 18	Complications of bronchiolitis are few, but most commonly include: Apnea particularly in children <6 weeks old Respiratory failure Concomitant or superimposed bacterial failure
Slide 19	The majority of children will be able to be discharged to home in the care of their parents/guardians. Caretakers need to be educated on the expectant clinical course and signs that the child may be deviating from that course. Caretakers need to be educated on how to properly suction the child and when to suction the child (ie, if having difficulty breathing or difficulty feeding). The healthcare provider needs to empower the caretaker to care for the child at home and recognize a worsening clinical course.
Slide 20	An explanation of the meaning of the American Academy of Pediatrics (AAP) recommendations based on aggregate evidence quality.
Slide 21	AAP Recommendations for diagnosis: The AAP revised recommendations about treatment of bronchiolitis in 2014. Bronchiolitis should be a clinical diagnosis, not relying on laboratory or imaging studies. Severe disease includes age less than 12 weeks, history of prematurity, underlying cardiopulmonary disease, immunodeficiency. Ralston SL, Lieberthal AS, Meissner HC, et al; American Academy of Pediatrics. Clinical practice guideline: the diagnosis, management, and prevention of bronchiolitis. *Pediatrics*. 2014;134(5):e1474-e1502.
Slide 22	AAP recommendations for management Beta-agonist are only recommended in certain populations – specifically for populations with chronic lung disease. Previously healthy children should not routinely receive beta-agonists. Hypertonic saline may be more useful in admitted patients in the inpatient setting than patients in the emergency setting.
Slide 23	AAP recommendations for management: Corticosteroids are indicated in reactive airway disease or sometimes in other chronic lung disease states, but they are not indicated in acute bronchiolitis in the previously healthy child. A clinician should ensure that a child is staying hydrated due to the high risk of dehydration. Avoid routine use of antibiotics for this condition that is usually due to a viral source unless there is a strong suspicion for superinfection or concomitant infection.

[Table t5-jetem-6-2-c73]

## Appendix E Bronchiolitis Small Group Discussion

**Target Audience:**
Generalist healthcare providers in low- and middle-income countries
**Educational Methods:**
Small group discussion
**Time required for implementation:**
About 45 minutes
**Learning Objectives:**
The learner will be able to:

1. Describe how to triage a child with respiratory symptoms.
2. Describe how to take a history and perform a physical exam for a child with respiratory symptoms.
3. List a differential diagnosis for a wheezing child.
4. Describe management of a child with bronchiolitis in the acute care setting.
5. Determine the appropriate disposition for a patient diagnosed with bronchiolitis in the acute care setting.

**Equipment/Environment/Personnel:**

- A large room (with a capacity of at least 50 people) with multiple tables and ample floor space, or multiple rooms if available
  ○ For each group of 3 to 5 learners, one setup includes 3 to 5 seats set up in a circle
- One small group facilitator per group of 3 to 5 learners

**Recommended pre-reading for instructor:**

- Pocket Book of Hospital Care for Children: Guidelines for the Management of Common Childhood Illnesses. 2nd edition. Geneva: World Health Organization; 2013. 1, Triage and emergency conditions. Available from: https://www.ncbi.nlm.nih.gov/books/NBK154450/ Available from: https://www.ncbi.nlm.nih.gov/books/NBK154450/

**Case Studies**

- Smith DK, Seales S, Budzik C. Respiratory Syncytial Virus Bronchiolitis in Children. *Am Fam Physician*. 2017;95(2):94–99.

**Abbreviations:**

HR – Heart Rate

RR – Respiratory Rate

BP – Blood Pressure

T – Temperature

O2 Sat – Oxygen Saturation

IV – intravenous

**Tips for successful implementation:**

- The facilitator should read the prompts (**in bold**) to the learners.
- Following the prompts are cues that can be given from the facilitator.
- Because the providers are from varying disciplines and with different backgrounds and experiences, discussion can be facilitated by asking participants to speak on their experiences with similar patients.
  ○ It is beneficial to have various disciplines represented in each group, rather than having the nurses be in one group and physicians in another. It can help lead to more robust discussion.
  ○ Nurses may not be very comfortable in advanced medical decision-making but they may feel comfortable in making triage decisions or sharing tips/tricks in achieving goals with pediatric patients.
  ○ The facilitator should ask follow-up questions to elicit clinical areas where practitioners feel that they perform well and areas in which they can improve, utilizing the group to help understand each other’s difficulties and problem-solve together.
  ○ Some participants may be very confident and talkative, but because this is meant to be a group exercise, it may be useful to initially have any member of the group respond, but then to selectively encourage those who are more reserved to give their responses.

**Case 1:** You are called to see a 10-month-old child brought in by her mother with cough, congestion and runny nose for 5 days that seems to be worse today. She has decreased oral intake. She is also feeling warm.

**Question Prompts:**

1. Describe how you would determine triage of this child.
  - A – Airway
  - B – Breathing
  - C – Circulation
  - D – Disability

Vital Signs[Table t6-jetem-6-2-c73]

**Table t6-jetem-6-2-c73:**

HR 150	RR 74	BP 82/58	T 100.7	O2 Sat: 89%

She is crying and clinging to her mother.

She has moderate nasal flaring and coughing.

There is nasal congestion and dry, thick mucus in the nose.

Her mucus membranes are tacky.

She has diffuse wheezing throughout the lung fields but good and symmetric air entry.

There are intercostal retractions. Her hands are warm and capillary refill is 4 seconds.

Skin pinch is normal. She has no signs of malnutrition.

*Emergency*

- Signs of respiratory distress
- Prolonged capillary refill

**Table t7-jetem-6-2-c73:**

Emergency Signs	(WHO)
Obstructed or absent breathing	
Severe respiratory distress	
Central cyanosis	
Signs of shock (cold extremities with capillary refill time >3s and weak and fast pulse)	
Coma (or seriously reduced level of consciousness)	
Seizures	
Diarrhea with 2 signs of severe dehydration	

[Table t7-jetem-6-2-c73]

2. What is your differential diagnosis?[Table t8-jetem-6-2-c73]
  **Table t8-jetem-6-2-c73:**
  Priority Signs	(WHO)
  Tiny infant (sick child < 2months)	
  Temperature	
  Trauma	
  Pallor	
  Poisoning	
  Pain (severe)	
  Respiratory distress	
  Restless, irritable, or lethargic	
  Referral	
  Malnutrition (visible, severe wasting)	
  Edema	
  Burns	
  - Bronchiolitis, pneumonia, foreign body, heart failure, asthma
3. What information will help you determine your most likely diagnosis?
  - *More history:*
    - Any sick contacts?
    - Any choking event (foreign body)?
    - Any difficulty feeding or turning blue with feedings (heart failure)?
    - Is the child small for weight (heart failure)?
    - Are there any close family members with asthma?
  - *Physical exam:*
    - Shifting atelectasis
    - No focality to lung exam (pneumonia)
    - No difference in air entry on one side (pneumonia, foreign body)
    - No abnormal heart sounds, hepatosplenomegaly, edema of lower extremities (heart failure)
  - Her older brother and father have a cold.
  - She has not had any choking events.
  - She eats well and is gaining weight appropriately.
4. What do you think is the most likely diagnosis?
  - *Bronchiolitis*
5. What are your next steps?
  - Nasal suction
  - Supplementary oxygen
  - IV fluids
  - Consider a trial of beta agonist
  - Reassess vital signs and physical exam

She improves.

Her O2 Sat is 98% on room air, her RR is 45 and her wheeze is barely audible.

She has very good air entry. She has made a full wet diaper.

6. What are your next steps?
  - Continue to observe patient
  - Determine whether the improvement can be attributed to the beta agonist. If so, consider another dose of the beta-agonist
    - Beta-agonists and ipratropium bromide, an aerosolized anticholinergic agent, have not routinely shown effectiveness in the management of infants with bronchiolitis and wheezing
  - Evaluate how the child is tolerating feeding
  - Consider discharge if patient has met discharge criteria
    - Well appearing
    - Good feeding
    - Clinically hydrated
    - No apnea or cyanosis
    - No atelectasis (if chest x-ray is obtained)
    - Older than 3 months old
    - Oxygen saturation >90%
    - Parent(s) can care for the child at home
  - Anticipatory guidance and return precautions
7. What if this same child on reassessment?
  Her O2 sat is 97% with supplementary O2 but 90% on room air, her retractions are still present, but moderate and her RR is 60. She has not had a wet diaper yet since arrival. She looks more comfortable.
  What are your next steps?
  - Admit to hospital
  - Nursing/Respiratory goal to titrate oxygen for O2 sat >90%
  - IV hydration
  - Escalate respiratory support as necessary
8. What if this same child’s condition worsens?
  Her O2 sat is 93% while receiving oxygen; she has deeper retractions. Her RR is 70. She is not as alert as before.
  - Obtain a chest x-ray (if not completed already) to look for other causes of respiratory distress
  - Consider intubation and intensive care admission for respiratory failure

**Case 2:** You are called to see a 10-month-old child brought in by her mother with cough, congestion and runny nose for 5 days that seems to be worse today. She has decreased oral intake. She’s also feeling warm.

**Question Prompts:**

1. Describe how you would determine triage of this child.
  - A – Airway
  - B – Breathing
  - C – Circulation
  - D – Disability

Vital Signs[Table t9-jetem-6-2-c73]

**Table t9-jetem-6-2-c73:**

HR 125	RR 52	BP 82/58	T 100.7	O2 Sat: 95%

She smiles as you look at her. She has some mild nasal flaring, wet cough and sneeze. + congestion and dry, thick mucus in nose. Moist mucus membranes. Diffuse, barely audible wheeze throughout the lung fields. Good air entry. She has no signs of malnutrition.

*Priority*

- Temperature
- Respiratory distress

2. What are your next steps?
  - Suction nares
  - Reassess vital signs and physical exam
    - If condition has improved, evaluate how the child is tolerating feeding and discharge home with anticipatory guidance and return precautions
    - If condition has not improved or deteriorated, consider other supportive measures indicated for bronchiolitis
      1. Oxygen
      2. Beta agonist trial
      3. IV fluids
      4. Antipyretics

## Appendix F Pneumonia Lecture

Please see associated PowerPoint file

## Appendix G Pneumonia Lecture Synopsis

**Target Audience:**
Generalist healthcare providers in low- and middle-income countries
**Educational Methods:**
PowerPoint didactic lecture
**Time required for implementation:**
About 60 minutes
**Learning Objectives:**
The learner will be able to:

1. Identify pneumonia as a top cause of mortality for children worldwide.
2. Identify the common bacterial pathogens that cause pediatric pneumonia.
3. Explain the utility of imaging and diagnostic testing indicated for pediatric pneumonia.
4. Identify the antibiotic options for treatment of pediatric pneumonia.

**Equipment/Environment:**

- A large room (with a capacity of at least 50 people) with multiple tables and ample floor space, or multiple rooms if available
- A computer with PowerPoint capability and projector setup
- Personnel needed: one lecturer

**Recommended pre-reading for instructor:**

- Messinger AI, Kupfer O, Hurst A, et al. Management of Community Acquired Bacterial Pneumonia. *Pediatrics in Review*. 2017;38(9):394–409.

### Lecture Script

**Table t10-jetem-6-2-c73:**

Slide 1	Introduction to topic. Opportunity to review objectives of lecture.
Slide 2	Present the clinical case. This is an opportunity to discuss a differential diagnosis.
Slide 3	The lecture format is as though a practitioner is having a discussion with intrigued students about pneumonia. “Questions will headline slides and can be proposed to the group.”
Slide 4	Introduce “wives’ tales” and colloquial causes of pneumonia which leads to the next slide explaining pneumonia etiology.
Slide 5	Review what is pneumonia. Although the focus of the lecture is bacterial pneumonia, it is important to recognize that pneumonia can also be caused by viruses.
Slide 6	Pneumonia (acute respiratory infection) is a top cause of mortality for children under 5, especially in children 1 month to 5 years old. WHO. Causes of Child Mortality, 2017. Accessed July 1, 2019. At: https://www.who.int/gho/child_health/mortality/causes/en/
Slide 7	Most of pediatric pneumonia is caused by bacterial pathogens. Michelow IC, Olsen K, Lozano J, et al. Anatomy and assessment of the pediatric airway. *Pediatrics*. 2004;113(4):701–7.
Slide 8	Review of most common bacterial pathogens by age.
Slide 9	Review characteristics of *S. pneumoniae* pneumonia
Slide 10	Review characteristics of *M. pneumoniae* pneumonia
Slide 11	Review characteristics of *S. aureus* pneumonia
Slide 12	Review characteristics of Group B Strep pneumonia
Slide 13	Review characteristics of *C. trachomatis* & *pneumoniae* pneumonia
Slide 14	Less common pathogens should be considered in certain cases - Anaerobes for patients who have risk of aspiration (neurologic disease, swallow dysfunction, tracheostomy/ventilator dependent) - Tuberculosis for patients who have persistent cough despite antibiotics, are immunocompromised or have high risk contacts (known TB contacts, incarcerated contacts)
Slide 15	It is important to remember that pneumonia is a clinical diagnosis! But if there is diagnostic uncertainty, it may be helpful to pursue imaging and laboratory testing.
Slide 16	A chest x-ray can be helpful in the diagnosis of pneumonia. A chest x-ray that is positive for pneumonia can show you an infiltrate, which may correspond to what you hear on your physical exam. It is important to not only get an AP view, but also a lateral to look for retrocardiac opacities that may not be well visualized on the AP film. Additionally, a chest x-ray can be helpful for adding or crossing off diagnoses from your differential diagnosis such as cardiac failure, pulmonary edema, empyema or tuberculosis. Remember that a chest x-ray’s appearance may lag behind the clinical findings.
Slide 17	Ultrasound can be incredibly helpful for diagnosis whether there is an effusion or empyema.
Slide 18	
Slide 19	Full Blood Count (FBC) Prior to the introduction of the PCV vaccine, a full blood count was helpful for finding occult pneumonia. One study of febrile children found that if a febrile patient had a WBC >20k, 19% had an occult pneumonia. After the introduction of PCV, this number dropped to 9%. It is important to consider getting an FBC in a febrile patient without a source because they may have an occult pneumonia. Bachur R, Perry H, Harper MB. Occult pneumonias: empiric chest radiographs in febrile children with leukocytosis. *Ann Emer Med.* 1999; 33(2): 166–73. **Rutman MS, Bachur R, Harper MB. Radiographic pneumonia in young, highly febrile children with leukocytosis before and after universal conjugate pneumococcal vaccination. *****Pediatr Emerg Care*****. 2009; 25(1): 1–7.**
Slide 20	Blood cultures Blood cultures are most useful in patients who sick enough to go to the pediatric intensive care unit, are immunocompromised, have a pleural effusion or empyema.
Slide 21	
Slide 22	To decide how to treat a patient, it is important to determine the severity of pneumonia. This classification is from the World Health Organization.
Slide 23	Not only is it important to consider the severity of the patient’s pneumonia, it is also important to account for the patient’s age when determining your antibiotic choice for treating pneumonia. These are the recommendations for children less than 3 months old.
Slide 24	These are the recommendations for children older than three months up until 2 years of age.
Slide 25	These are the recommendations for children older than 2 years up until age 5.
Slide 26	These are the recommendations for children older than 5 years old.
Slide 27	Dehydration is the most common complication from pneumonia, due to increased insensible losses. It is important to consider the patient’s fluid needs and support those needs appropriately.
Slide 28	Any questions?
Slide 29	Here is a chart showing the World Health Organization’s classification of pneumonia with the corresponding treatment
Slide 30	Pediatric vital signs vary depending on the age of the patient. Here is a helpful chart that shows normal respiratory rates of children based on their age.

[Table t10-jetem-6-2-c73]

## Appendix H Pediatric Pneumonia Rapid Cycle Deliberate Practice (RCDP) Case

**Target Audience:**
Generalist healthcare providers in low- and middle-income countries
**Educational Methods:**
Simulation Rapid Cycle Deliberate Practice
**Recommended Number of Learners per Instructor:**

- One simulation instructor/debriefing facilitator per group of 3 to 6 learners
  ○ This person should be well-versed in the medical theory taught by the simulations presented
- One confederate/assistant per group of 3 to 6 learners

**Time required for implementation:**
~ 45 minutes for multiple rounds of RCDP
**Learning Objectives:**
By the end of the session, learners should be able to: Cognitive:

1. Recognize septic shock
2. Understand that there may be multiple etiologies of shock
3. Consider various causes of shortness of breath
4. Consider various causes of fever

Technical:

1. Perform a rapid initial assessment
2. Perform peripheral IV line or IO placement
3. Perform airway management if patient clinically deteriorates

Behavioral:

1. Communicate clear leadership roles with delegation of roles
2. Perform early fluid resuscitation of a patient in shock
3. Administer appropriate fluid in a patient with septic shock
4. Administer early antibiotics for a patient with concern for septic shock and pneumonia
5. Early respiratory interventions in a patient with respiratory complaint

**Learner responsible content:**

- Appendix F. Pneumonia Lecture

**Abbreviations**

AED = automatic external defibrillator

BP = blood pressure

Bpm = beats per minute

BVM = bag valve mask

CAP=community acquired pneumonia

CPR = cardiopulmonary resuscitation

EKG = electrocardiogram

EMS = emergency medical services

ETT=endotracheal tube

FH=family history

GCS = Glasgow Coma Scale

HPI=history of present illness

HR = heart rate

Hx=history

IO = intraosseous

IV = intravenous

LR = Ringer’s lactate

NS = normal saline

O2 = oxygen

PEA = pulseless electrical activity

PIV=peripheral intravenous line

PO=per ora, by mouth

Pt = patient

RCDP = rapid cycle deliberate practice

ROS=review of symptoms

RR = respiratory rate

RSI=rapid sequence intubation

O_2_Sat = oxygen saturation

T = temperature

**Case Title:** Pediatric Pneumonia RCDP Practice Case

**Case Description & Diagnosis (short synopsis):** Kimberly is brought to the A&E bed. She has unstable vital signs. She requires fluid resuscitation, respiratory support, and early antibiotics for acute CAP and septic shock. Despite appropriate interventions, she will progress to impending respiratory failure. If inappropriate or delayed interventions, she will decompensate to Pulseless Electrical Activity (PEA) arrest due to acidosis, hypovolemia, and hypoxic respiratory failure.

**Equipment or props needed:**

Setup for All Rounds:

- Room configuration: typical A&E bed/stretcher (or table)
- For each group of 3 to 5 learners, one equipment setup includes the following:
  ○ A low-fidelity full-body simulation mannequin. If available, higher-fidelity mannequins can be used (we used a MegaCode Kid, and Laerdal ALS Baby mannequins)
  ○ An intravenous arm task trainer (if the mannequin is not equipped)
  ○ Lower extremity capable of intraosseous (IO) insertion (if mannequin is not equipped)
  ○ A medical resuscitation setup including the standard resuscitation equipment available in the A&E department (intravenous line starter kits, intravenous fluids, IO drill, IO needles, medical tape, bag valve mask, mock medications, etc.)
- Personnel:
  ○ Simulation instructor/debriefer
  ○ Confederates: patient’s mother (Shelia), inpatient pediatrician
  ○ Demonstration items needed for debriefing: same as equipment

**How the Scenario Unfolds:**

**Ideal Scenario Flow:**

Kimberly is brought to the A&E bed. The learners are expected to obtain a full set of vital signs and recognize that her vital signs are unstable. They should also perform a rapid initial assessment. Participants should be concerned for hypovolemic shock/septic shock and they should give supplemental O2 and obtain vascular access for labs and medication administration. They learners should give an appropriate weight-based dose of isotonic fluids rapidly and initiate antibiotics early. They should be concerned for community acquired pneumonia as the source of septic shock and obtain and interpret a chest x-ray. They will also need to interpret the laboratory studies that they obtained.

After their interventions, they should reassess the patient including a reassessment of vital signs. Despite appropriate interventions, she will progress to impending respiratory failure. The learners should support her airway to their best ability (ie, bag valve mask, endotracheal intubation, etc.).

If inappropriate or delayed interventions, she will decompensate to Pulseless Electrical Activity (PEA) arrest due to acidosis, hypovolemia, and hypoxic respiratory failure.

The participants should end the case with a handoff to the inpatient physician – summarizing the case using proper medical terminology.

**Critical actions:**

Round 1:

1. Rapid assessment of Airway, Breathing & Circulation
2. Check vital signs
3. Obtain IV/IO access
4. Recognize abnormal vital signs
5. Take basic history from the patient

Round 2:

1. Recognize shock (and etiology)
2. Give supplemental O2
3. IV fluids
4. Check glucose
5. Administer glucose
6. Re-assess after fluid administration

Round 3:

1. Recognize continued tachycardia
2. Obtain labs
3. Obtain a chest x-ray
4. Give epinephrine IV/IO (1:10,000)
5. Give early antibiotics
6. Recognize need to admit to hospital

Round 4:

1. Recognize continued tachycardia and tachypnea then slowed breathing
2. Airway intervention
3. Administer Rapid Sequence Intubation (RSI)
4. Confirm ETT placement
5. Admission to intensive care unit

**Expected Endpoint of the Scenario:** maximum 10-minute time limit per round, but facilitator can stop at any point during scenario

**Possible Distractors Within Scenario**: high acuity, family members want to take patient home to care for her, family does not believe hospital will help their child

**Optional Challenges for Higher Level Learners**:

- Evaluation for altered mental status
- Considering other types of shock

**Roles of Participants/Trainees:** usual roles within the A&E

**Roles of Confederates (if applicable):**

Mother (Sheila): provide medical history, primary caretaker of patient

Inpatient Physician: take handoff for transition of care from learners

**Anticipated Management Errors:**

1. Failure to recognize the unresponsive patient: Because this is a low-fidelity simulation and a simulated cardiac monitor is not used, learners may not realize that the patient is not interacting, and they often do not realize the deterioration in the vital signs. It is useful for the confederate to clearly indicate that there is an acute change in vital signs and that the patient has deterioration of vital signs even if the provider does not check.

**Case Title:** Pediatric Pneumonia RCDP Practice Case

**Chief Complaint:** “Not acting ‘herself’”[Table t11-jetem-6-2-c73]

**Table t11-jetem-6-2-c73:**

**Initial Vitals for all rounds:** HR 140	BP 78/42	RR 40	Temp 39.2 °C	SpO2 91%
Weight 20kg				

**Initial Physical Exam for All Rounds:**

**General appearance:** She appears fatigued. Dry mucous membranes. Increased work of breathing. Lethargic. Pale.

**Initial Assessment:**

- **Airway/Breathing:** Airway open. Speaks in clear sentences. Lungs with shallow respirations. Rhonchi at right middle and lower lobes and scant wheezes in the same distribution. Tachypneic. Occasionally coughing.
- **Circulation**: Tachycardic. No murmur, weak central and peripheral pulses. Capillary refill 4–5 seconds, extremities cool. Pale. Dry mucous membranes.
- **Mental Status:** Lethargic

**Case Title:** Pediatric Pneumonia RCDP Practice Case

**ROUND 1: Initial assessment of patient**

**Objectives introduced this round**:

1. Perform a rapid initial assessment
2. Perform peripheral IV placement

**Prompt for Team:** “A 5-year-old girl is brought to the A&E because she has not been acting ‘herself.’ She has had cough and fever for 3 days.”

**Pertinent history (if asked):**

- **History of present illness:** The child has been having fever and cough for the past 3 days. Today, she seems significantly more tired and has not been interacting much with her family.
  She has not been eating since yesterday and has only been drinking small sips.
  Mother insists that she is usually quite energetic and finds herself in “time out” because she can be a handful at home.
  No known sick contacts.
  Mother gave acetaminophen this morning. No other medications tried at home.
  She feels feverish, but no chills. She does not have nausea or vomiting. No diarrhea.
  Last bowel movement was last night and described as “normal.”
  Last urination was last night.
- **ROS:**
  ○ Positive: fever, fatigue, listless, cough
  ○ Negative: night sweats, weight loss, lymphadenopathy, ecchymosis, rash, vision change, rhinorrhea, sinus pain, epistaxis, dental problems, weight change, palpitations, syncope, edema, cyanosis, orthopnea, hemoptysis, nausea, vomiting, diarrhea, blood in stool, appetite change, abdominal pain, dysuria, frequency, urgency, hematuria, joint pain or swelling, muscle pain, back pain, headache, weakness
- **Medications:** acetaminophen
- **Past medical & surgical history:** Admitted for jaundice as a neonate. Immunizations are up to date.
- **Family history**: Grandfather has type 2 diabetes.
- **Social history:** Lives at home with her mother, father, grandfather, and younger sisters. She is in kindergarten.

[Table t12-jetem-6-2-c73]

**Table t12-jetem-6-2-c73:**

**Vital signs** (need to be obtained): HR 140	BP 78/42	RR 40	Temp 39.2 °C	SpO2 91%
Weight 20kg				

**Physical exam findings (if asked):**

**Initial Assessment:**

- **Airway/Breathing:** Airway open. Speaks in clear sentences. Lungs with shallow respirations. Rhonchi at right middle and lower lobes and scant wheezes in the same distribution. Tachypneic. Occasionally coughing.
- **Circulation**: Tachycardic. No murmur, weak central and peripheral pulses. Capillary refill 4–5 seconds, extremities cool. Pale. Dry mucous membranes.
- **Mental Status:** Lethargic

*No scenario progression yet.*

**End Round 1.**

**Round 1 Debriefing and Evaluation:**

Review the initial exam, reinforce the importance of rapid initial assessment, checking vital signs and obtaining IV/IO access in a critically ill patient.

Refer to expected actions and teaching points below:[Table t13-jetem-6-2-c73]

**Table t13-jetem-6-2-c73:**

Expected Action	Teaching Point	Result
Rapid assessment of Airway, Breathing, and Circulation	When presented with ill patient, assess circulation, airway and breathing and immediately intervene if emergent situation found	Patient is in shock
Check vital signs	Document initial vitals	
Obtain IV/IO access	Try PIV first, particularly if patient awake. IO if critically ill, particularly if altered mental status/coma	If re-doing case, can make PIV unattainable
Recognize abnormal vitals	HR for age: greater than 120bpm is abnormal BP: less than 78/42 is considered hypotensive for child’s age RR greater than 28 is tachypneic for child’s age	
Take basic history from the patient and mother	History is important to management. Focus should be on the past medical history, surgical history, and medications. This will guide management. Try to obtain information while performing other tasks. This can be achieved with appropriate role delegation.	

**END SCENARIO (1)**

**Case Title:** Pediatric Pneumonia RCDP Practice Case

**ROUND 2: Initial management of shock**

**Objectives introduced this round:**

1. Recognize shock and etiology
2. Give supplemental oxygen
3. Perform early fluid resuscitation of a patient in shock
4. Assess and treat hypoglycemia
5. Re-assess patient after IV fluid administration

**Restart scenario from beginning.**

**Prompt for team:** “A 5-year-old girl is brought to the A&E because she has not been acting ‘herself.’ She has had cough and fever for 3 days.”

**Pertinent history (if asked):**

- **History of present illness:** The child has been having fever and cough for the past 3 days. Today, she seems significantly more tired and has not been interacting much with her family.
  She has not been eating since yesterday and has only been drinking small sips.
  Mother insists that she is usually quite energetic and finds herself in “time out” because she can be a handful at home.
  No known sick contacts.
  Mother gave acetaminophen this morning. No other medications tried at home.
  She feels feverish, but no chills. She does not have nausea or vomiting. No diarrhea.
  Last bowel movement was last night and described as “normal.”
  Last urination was last night.
- **ROS:**
  ○ Positive: fever, fatigue, listless, cough
  ○ Negative: night sweats, weight loss, lymphadenopathy, ecchymosis, rash, vision change, rhinorrhea, sinus pain, epistaxis, dental problems, weight change, palpitations, syncope, edema, cyanosis, orthopnea, hemoptysis, nausea, vomiting, diarrhea, blood in stool, appetite change, abdominal pain, dysuria, frequency, urgency, hematuria, joint pain or swelling, muscle pain, back pain, headache, weakness
- **Medications:** acetaminophen
- **Past medical & surgical history:** Admitted for jaundice as a neonate. Immunizations are up to date.
- **Family history**: Grandfather has type 2 diabetes.
- **Social history:** Lives at home with her mother, father, grandfather, and younger sisters. She is in kindergarten.[Table t14-jetem-6-2-c73]

**Table t14-jetem-6-2-c73:**

**Vital signs** (need to be obtained): HR 140	BP 78/42	RR 40	Temp 39.2 °C	SpO2 91%
Weight 20kg				

**Physical exam findings** (if asked):

**Initial Assessment:**

- **Airway/Breathing:** Airway open. Speaks in clear sentences. Lungs with shallow respirations. Rhonchi at right middle and lower lobes and scant wheezes in the same distribution. Tachypneic. Occasionally coughing.
- **Circulation**: Tachycardic. No murmur, weak central and peripheral pulses. Capillary refill 4–5 seconds, extremities cool. Pale. Dry mucous membranes.
- **Mental Status:** Lethargic

**Expected actions after verbal prompt:**

□ Rapid assessment of ABC
□ Check vital signs, and recognize abnormal vitals
□ Obtain peripheral IV, or IO if unable
□ Take basic history from patient
□ Recognize shock and etiology and verbalize
□ Give isotonic fluids
□ Reassess patient after bolus

*No scenario progression yet.*

**END Round 2.**

**Round 2 Debriefing and Evaluation:**

Review initial exam, reinforce importance of rapid initiation of IVF in shock, appropriate fluid volume for pediatric patients, and immediate reassessment after fluids. Discuss learners’ differential diagnosis and why checking glucose is important in an acutely ill pediatric patient.

Refer to expected actions and teaching points below:[Table t15-jetem-6-2-c73]

**Table t15-jetem-6-2-c73:**

Expected Action	Teaching Point	Result
Recognize shock (& etiology).	In a hypotensive, tachycardic patient with cough and tachypnea and fever, strongly consider septic shock.	
Give supplemental O2.	A reasonable goal for SpO2 is >93%. Titrate to SpO2.	NC → 92% Simple face mask →96%
Give IV fluids.	Rapid bolus, 20mL/kg (800mL) rapid infusion for a previously pediatric patient with suspected septic shock. Only isotonic fluids (NS or LR) should be given as a bolus.	Improved HR to 125 and BP to 98/60.
Check glucose.	Always check for hypoglycemia in a patient with altered mental status (ie, lethargy). If the learners do not check glucose, the patient should have a non-sustained seizure.	Glucose is 50mg/dL (2.8mmol/L). Seizures should abate with a benzodiazepine and with glucose administration.
Administer glucose.	Be familiar with appropriate glucose supplementation in a pediatric patient. 10mL/kg of D5 or 5mL/kg of D10.	Reassess patient and glucose after intervention. Glucose recheck is 90mg/dL (5mmol/L).
Reassess after bolus.	Always recheck vitals, perfusion, and respiratory status, and mental status after performing interventions in a critically ill patient.	Improved vital signs, but not within normal limits.

**END SCENARIO (2)**

**Case Title:** Pediatric Pneumonia RCDP Practice Case

**ROUND 3: Assessment and Treatment of Septic Shock & Pneumonia**

**Objectives introduced this round:**

1. Recognize continued tachycardia
2. Obtain labs
3. Obtain a chest x-ray
4. Give early antibiotics
5. Recognize need to admit to hospital

**Restart from beginning of scenario.**

**Prompt for team:** “A 5-year-old girl is brought to the A&E because she has not been acting ‘herself.’ She has had cough and fever for 3 days.”

**Pertinent history (if asked):**

- **History of present illness:** The child has been having fever and cough for the past 3 days. Today, she seems significantly more tired and has not been interacting much with her family.
  She has not been eating since yesterday and has only been drinking small sips.
  Mother insists that she is usually quite energetic and finds herself in “time out” because she can be a handful at home.
  No known sick contacts.
  Mother gave acetaminophen this morning. No other medications tried at home.
  She feels feverish, but no chills. She does not have nausea or vomiting. No diarrhea.
  Last bowel movement was last night and described as “normal.”
  Last urination was last night.
- **ROS:**
  ○ Positive: fever, fatigue, listless, cough
  ○ Negative: night sweats, weight loss, lymphadenopathy, ecchymosis, rash, vision change, rhinorrhea, sinus pain, epistaxis, dental problems, weight change, palpitations, syncope, edema, cyanosis, orthopnea, hemoptysis, nausea, vomiting, diarrhea, blood in stool, appetite change, abdominal pain, dysuria, frequency, urgency, hematuria, joint pain or swelling, muscle pain, back pain, headache, weakness
- **Medications:** acetaminophen
- **Past medical & surgical history:** Admitted for jaundice as a neonate. Immunizations are up to date.
- **Family history**: Grandfather has type 2 diabetes.
- **Social history:** Lives at home with her mother, father, grandfather, and younger sisters. She is in kindergarten.[Table t16-jetem-6-2-c73]

**Table t16-jetem-6-2-c73:**

**Vital signs (need to be obtained):** HR 140	BP 78/42	RR 40	Temp 39.2 °C	SpO2 91%
Weight 20kg				

**Physical exam findings** (if asked):

- **Airway/Breathing:** Airway open. Speaks in clear sentences. Lungs with shallow respirations. Rhonchi at right middle and lower lobes and scant wheezes in the same distribution. Tachypneic. Occasionally coughing.
- **Circulation**: Tachycardic. No murmur, weak central and peripheral pulses. Capillary refill 4–5 seconds, extremities cool. Pale. Dry mucous membranes.
- **Mental Status:** Lethargic

**Expected actions after verbal prompt:**

□ Rapid assessment of ABC
□ Check vital signs, and recognize abnormal vitals
□ Obtain peripheral IV, or IO if unable
□ Take basic history from patient
□ Recognize shock and etiology and verbalize
□ Consider other causes of lethargy
□ Obtain glucose measurement
□ Give isotonic fluids
□ Reassess patient after bolus

**Scenario progression #1:** *After the first bolus, HR 130, BP 98/60, RR 35, sat 95% on facemask/nasal cannula (92% on RA). T 38.9 °C.*

*The patient continues to have tachypnea.*

*Updated physical exam:*

*CIRCULATION: Normotensive. Tachycardic. Capillary refill 3sec. Hands are now warm.*

**Expected actions after scenario progression:**

□ Recognize continued tachycardia
□ Give antipyretic (if not already given)
□ Obtain lab work
□ Obtain chest x-ray
□ Start broad-spectrum IV antibiotics
□ Admit to the hospital

**END Round 3.**

**Round 3 Debriefing and Evaluation:**

Review ongoing management including fluids. Providers should be able to advocate for an appropriate level of care for a critically ill patient. Septic shock requires early fluid replacement and antibiotic administration for best outcomes.

Refer to expected actions and teaching points below:[Table t17-jetem-6-2-c73]

**Table t17-jetem-6-2-c73:**

Expected Action	Teaching Point	Result
Recognize continued tachycardia.	Recheck vitals after every critical intervention. Consider EKG.	EKG shows sinus tachycardia.
Obtain labs.	WBC elevated. This supports diagnosis of septic shock but is not necessary for diagnosis.	
Obtain a chest x-ray.	Right middle lobar pneumonia; probably S. pneumoniae due to patient demographics.	
Give early antibiotics.	Ceftriaxone 50mg/kg IV q 12 hours Cefuroxime 50mg/kg IV q 6 hours Consider adding: Azithromycin 10mg/kg day 1; 5mg/kg days 2–5 Erythromycin 30–50 mg/kg/day PO divided q6–12hr	Broad coverage against community acquired pneumonia and atypical bacterial pneumonia particularly because the patient is in septic shock.
Admission to hospital.	High risk patient needs to be admitted with continuous monitoring and antibiotics with respiratory support.	

**END SCENARIO (3)**

**Case Title:** Pediatric Pneumonia RCDP Practice Case

**ROUND 4: Recognition& Intervention for Respiratory Failure**

**Objectives introduced this round:**

1. Recognize impending respiratory failure
2. Airway support
3. Admission to intensive care unit

**Restart from beginning of scenario.**

**Prompt for team:** “A 5-year-old girl is brought to the A&E because she has not been acting ‘herself.’ She has had cough and fever for 3 days.”

**Pertinent history** (if asked):

- **History of present illness:** The child has been having fever and cough for the past 3 days. Today, she seems significantly more tired and has not been interacting much with her family.
  She has not been eating since yesterday and has only been drinking small sips.
  Mother insists that she is usually quite energetic and finds herself in “time out” because she can be a handful at home.
  No known sick contacts.
  Mother gave acetaminophen this morning. No other medications tried at home.
  She feels feverish, but no chills. She does not have nausea or vomiting. No diarrhea.
  Last bowel movement was last night and described as “normal.”
  Last urination was last night.
- **ROS:**
  ○ Positive: fever, fatigue, listless, cough
  ○ Negative: night sweats, weight loss, lymphadenopathy, ecchymosis, rash, vision change, rhinorrhea, sinus pain, epistaxis, dental problems, weight change, palpitations, syncope, edema, cyanosis, orthopnea, hemoptysis, nausea, vomiting, diarrhea, blood in stool, appetite change, abdominal pain, dysuria, frequency, urgency, hematuria, joint pain or swelling, muscle pain, back pain, headache, weakness
- **Medications:** acetaminophen
- **Past medical & surgical history:** Admitted for jaundice as a neonate. Immunizations are up to date.
- **Family history**: Grandfather has type 2 diabetes.
- **Social history:** Lives at home with her mother, father, grandfather, and younger sisters. She is in kindergarten.[Table t18-jetem-6-2-c73]

**Table t18-jetem-6-2-c73:**

**Vital signs (need to be obtained):** HR 140	BP 78/42	RR 40	Temp 39.2 °C	SpO2 91%
Weight 20kg				

**Physical exam findings** (if asked):

- **Airway/Breathing:** Airway open. Speaks in clear sentences. Lungs with shallow respirations. Rhonchi at right middle and lower lobes and scant wheezes in the same distribution. Tachypneic. Occasionally coughing.
- **Circulation**: Tachycardic. No murmur, weak central and peripheral pulses. Capillary refill 4–5 seconds, extremities cool. Pale. Dry mucous membranes.
- **Mental Status:** Lethargic

**Expected actions after verbal prompt:**

□ Rapid assessment of ABC
□ Check vital signs, and recognize abnormal vitals
□ Obtain peripheral IV, or IO if unable
□ Take basic history from patient
□ Recognize shock and etiology and verbalize
□ Give isotonic fluids
□ Reassess patient after bolus

**Scenario progression #1:** *After the first bolus, HR 135, BP 98/60, RR 15, sat 92% on facemask/nasal cannula (86% on RA). T 38.9 °C.*

*The patient continues to have tachycardia and is now bradypneic.*

*Updated physical exam:*

*AIRWAY/BREATHING: Now with retractions and bradypnea.*

*CIRCULATION: Normotensive. Tachycardic. Capillary refill 3sec. Hands are now warm.*

*MENTAL STATUS: Decreased responsiveness. Responds to pinching but not voice.*

**Expected actions after scenario progression:**

□ Recognize continued tachycardia
□ Give antipyretic (if not already given)
□ Obtain lab work
□ Obtain chest x-ray
□ Start broad-spectrum IV antibiotics
□ Bag mask ventilate the patient

**Scenario progression #2:** *You are supporting the patient’s breaths with bag-mask ventilation (improved O2 saturation to 94%) but patient continues to be bradypneic.*

*Updated physical exam:*

*AIRWAY/BREATHING: Continued retractions and bradypnea.*

*CIRCULATION: Tachycardic, normotensive. Capillary refill <3secs. Warm hands.*

*MENTAL STATUS: Only responds to voice.*

**Expected actions after scenario progression:**

- Providers who are not skilled in endotracheal intubation should provide respiratory support by demonstrating BVM
- Providers who are skilled in endotracheal intubation should:
  ○ Prepare for rapid sequence intubation
    ■ Name which medication to be used and reasoning
    ■ Name what equipment should be available (including endotracheal tube & laryngoscope size)
  ○ Intubate patient
  ○ Post-intubation
    ■ Endotracheal tube placement confirmation
- Admit patient to the pediatric intensive care unit

**END Round 4.**

**Round 4 Debriefing and Evaluation:**

Review ongoing management. Providers should be able to advocate for an appropriate level of care for a critically ill patient. Impending respiratory failure requires supporting the patient’s breathing which can be as minimal as bag mask ventilation or as invasive as intubation depending on the operator’s skillset.

Refer to expected actions and teaching points below:[Table t19-jetem-6-2-c73]

**Table t19-jetem-6-2-c73:**

Expected Action	Teaching Point	Result
Recognize impending respiratory failure.	Recheck vitals after every intervention. Bag mask ventilation must be started to address bradypnea and worsening oxygenation.	Improved oxygenation to 94% but continued bradypnea. If no bag mask ventilation done, patient can go into cardiac arrest and provider will have to provide CPR.
Bag valve mask.	Practice correct BVM technique in patient with respiratory failure.	Improved O2 sat, improved RR, improved patient perfusion.
Prepare for rapid sequence intubation.	Be comfortable with medications used for rapid sequence intubation in pediatric patients.	
Endotracheal tube placement confirmation.	There are several ways to confirm endotracheal tube placement. Provider should be able to list several.	If ETT placement not confirmed, patient should have persistent hypoxia and unequal breath sounds on exam (increased on the right for possible right mainstem intubation).
Admission to hospital.	High risk patient intubated for respiratory failure needs to be admitted to the intensive care unit with continuous monitoring and antibiotics with respiratory support.	

**END SCENARIO (4)**

**Case Title:** Pediatric Pneumonia RCDP Practice Case

**ROUND 5: Full scenario**

If time allows, restart from the beginning of the scenario and run the full scenario without interruption. If initial actions are performed well, can restart where needed.

**END OF RCDP Session.**

**Final debriefing and feedback**

- Praise learners for tasks accomplished well
- Provide areas for continued improvement.

*Chest Radiograph* [Fig f2-jetem-6-2-c73]

*Electrocardiogram (ECG)* [Fig f3-jetem-6-2-c73]

*Complete Blood Count* [Table t20-jetem-6-2-c73]

**Table t20-jetem-6-2-c73:**

White blood count (WBC)	18.7 × 1000/mm3
Hemoglobin (Hgb)	12.3 g/dL
Hematocrit (HCT)	36.9%
Platelets (Plt)	325 ×1000/mm3

**Table t21-jetem-6-2-c73:**

Segmented Neutrophils	66.7 K/μL
Bands	1.76%
Lymphocytes	25.8 K/μL

[Table t21-jetem-6-2-c73]

## Appendix I Asthma Lecture

Please see associated PowerPoint file

## Appendix J Asthma Lecture Synopsis

**Target Audience:**
Generalist healthcare providers in low- and middle-income countries
**Educational Methods:**
PowerPoint didactic lecture
**Time required for implementation:**
About 60 minutes
**Learning Objectives:**
The learner will be able to:

1. Recognize the clinical presentation of acute asthma.
2. Understand the pathophysiology of pediatric asthma exacerbations.
3. Delineate the efficacy of current therapeutic interventions in the treatment of acute asthma.
4. Discuss the role of clinical severity scores in assessing acute asthma.
5. Outline a clinical approach/protocol to the treatment of acute asthma.

**Equipment/Environment:**

- A large room (with a capacity of at least 50 people) with multiple tables and ample floor space, or multiple rooms if available
- A computer with PowerPoint capability and projector setup
- Personnel needed: one lecturer

**Recommended pre-reading for instructor:**

- Patel SJ, Teach SJ. Asthma. *Pediatrics in Review*. 2019;40(11):549–567. doi:10.1542/pir.2018-0282[Table t22-jetem-6-2-c73]

### Lecture Script

**Table t22-jetem-6-2-c73:**

Slide 1	Introduction to topic.
Slide 2	Opportunity to review objectives of lecture.
Slide 3	Asthma is the most common childhood illness globally. The majority of cases will present before age 5 and can present in various ways: Viral-induced, Seasonal, Exercise-induced, or Allergens/Irritant-induced.
Slide 4	Discuss risk factors for asthma. We all know there is a genetic component, as well as an association with eczema (up to 80% develop asthma) and allergic rhinitis. The environment also plays a role.
Slide 5	“However, not all wheezing equals asthma.” What else can cause wheezing? *Have audience call out suggestions; then review list.*
Slide 6	Let’s briefly review pathophysiology. Would anyone like to explain their understanding of asthma? Asthma is a disease that affects the lower airways. The triad of bronchial constriction, airway hyperresponsiveness and airway inflammation is what causes the clinical presentation. Note that children’s airways are smaller than adults to begin with, so children will be more prone to wheeze. Typically, an exacerbation is caused by a viral URI (RSV, influenza, rhinovirus) which attacks respiratory epithelial cells, causing sloughing and autoimmune inflammation leading to edema and smooth muscle disruptions. If mucous from viral infection, then clogs up bronchioles even more; this leads to worsening obstruction.
Slide 7	To put this into context, let’s start with a case. *Ask for audience participation/response as you read the slide prompts.*
Slide 8	How do you assess asthma severity? *Again, ask for audience participation suggesting clinical severity scores, exam, etc. Also ask about what makes a patient high-risk? After receiving answers from audience, lead into next slide:* OK, so this is what we are going to discuss next.
Slide 9	The Pediatric Respiratory Assessment Measure (PRAM) score is one of the clinical tools used to assess asthma severity. First described by Ducharme in 2000 in the preschool population, then validated in the larger pediatric population in 2008. It utilizes physical exam findings and oxygen saturation to assess severity on a scale from 0 to 12 which includes the following categories: 0–3 is low risk (<10%), 4 to 7 is moderate risk (10–50%) and 8 to 12 is high risk (>50%) of hospital admission. (*can refer to the table that appears on second mouse click).*
Slide 10	The Pediatric Asthma Severity Score (PASS) is another clinical tool used to assess asthma severity. It is simpler than the PRAM score, utilizing three physical exam findings (notably, not oxygen saturation) to assess severity on a scale from 0 to 6. *Can refer to table that appears on second mouse click.*
Slide 11	The bottom line is that utilizing a severity score in your institution will normalize communication across the healthcare team (nursing, physician, respiratory therapist) and facilitate a quicker assessment and disposition of the patient presenting with an acute asthma exacerbation. However, it should be a score that is evidence-based.
Slide 12	So, going back to our case: how do you know who is high risk (in addition to calculating a clinical severity score)? What are some questions you ask about a patient’s history when you are assessing an acute asthma exacerbation? *Elicit suggestions from the audience; then discuss the list on the slide.*
Slide 13	So now we are going to move into therapies for acute asthma. Any questions regarding the pathophysiology, clinical severity scores, or risk factors before we move on?
Slide 14	We will start with the mainstay of asthma therapies, beta-agonists. Beta-agonists are smooth muscle relaxers, thereby reversing bronchoconstriction. We will review standard dosing here. Remember that a metered dose inhaler (MDI) should always be used with a spacer. If there is not a spacer available, you can easily make one with a plastic bottle (*refer to photo*).
Slide 15	Next, we’ll talk about anticholinergics. Anticholinergics are also smooth muscle relaxants and act to reverse bronchoconstriction. They are most commonly recommended to be utilized in moderate to severe asthma exacerbations. Combination Therapy with SABA has been shown to lower the risk of hospitalization in ANY pediatric asthma exacerbation, regardless of severity (relative risk [RR] 0.73, 95% CI 0.63–0.85).
Slide 16	Steroids, or glucocorticoids, are also a mainstay for moderate to severe asthma exacerbations. These reverse airway edema and inflammation through anti-inflammatory action; however, they TAKE TIME TO WORK (2–4 hrs). Because of this, they need to be GIVEN EARLY to prevent hospitalization. When they are given appropriately, you only need to treat 8 patients to have a significant reduction in admission rates AND time to clinical improvement and discharge.
Slide 17	Let’s review the modes and dosing administration of steroids. Note that inhaled steroids are NOT recommended for an acute exacerbation; those are considered controller or maintenance medications and don’t have a role in an acute exacerbation.
Slide 18	So those are the standard meds in your toolkit, but what about other adjuncts? Magnesium also works as a smooth muscle relaxant. It is utilized in severe exacerbations. *Review dosing and contraindications.* When utilized in combination with the other standard asthma tools (B-Agonist, Steroid, Anticholinergic) you only need to treat 4 patients to prevent hospitalization.
Slide 19	Epinephrine is a beta agonist as well; however, it is a parenteral agent rather than inhaled. When is this useful? When a patient is so bronchoconstricted, you can’t get a b-agonist into their lungs! Don’t be afraid to use this in severe exacerbations, it can literally be life or death. *Review dosing and side effects.*
Slide 20	Lastly, let’s discuss theophylline. Theophylline is an old drug that has both smooth muscle relaxation and anti-inflammatory properties. It isn’t utilized much in developed countries because of its narrow therapeutic window; however, in low-resource settings it may be one of the only agents available so we will briefly review it here. *Review dosing and side effects.*
Slide 21	Finally, let’s discuss our respiratory adjuncts. Noninvasive positive pressure ventilation (ie, continuous or bi-level) is typically reserved for severe exacerbations or children with impending respiratory arrest to rescue them from being intubated. What are some signs you can think of that would signal impending respiratory arrest?
Slide 22	Our last choice is to intubate these patients. Why? Because they are so difficult to manage on a ventilator. You have to carefully adjust the pressure and volume settings; they are prone to barotrauma and air trapping. If you are intubating an asthmatic, remember implement prolonged I:E ratio, low tidal volumes, permissive hypercapnia, and at times higher positive inspiratory pressure pressures (PIPs).
Slide 23	How do we put these scores and therapies together?
Slide 24	For pediatric patients, it is often recommended to create or adopt an institutional protocol so that communication is streamlined among departments and healthcare providers, and patients receive evidence-based standard of care. An example asthma protocol is shown here (*can discuss pathways).*
Slide 25	In general, standard admission criteria include a moderate/severe asthma severity score (PRAM listed here), persistent hypoxia, or other discharge concerns (eg, social context).
Slide 26	In general, standard discharge criteria include a low asthma severity score (PRAM listed here), no oxygen requirement, sustained response to the therapies received (eg, need to be observed for a few hours), and a good social situation. Asthma action plans/education should also be an integral part of the discharge process.
Slide 27	Thank you for your time. Any questions?

## Appendix K Pediatric Asthma Rapid Cycle Deliberate Practice (RCDP) Case

**Target Audience:**
Generalist healthcare providers in low- and middle-income countries
**Educational Methods:**
Simulation Rapid Cycle Deliberate Practice
**Recommended Number of Learners per Instructor:**

- One simulation instructor/debriefing facilitator per group of 3 to 6 learners
  ○ This person should be well-versed in the medical theory taught by the simulations presented
- One confederate/assistant per group of 3 to 6 learners

**Time required for implementation:**
~ 45 minutes for multiple rounds of RCDP
**Learning Objectives:**
By the end of the session, learners should be able to: Cognitive:

1. Recognize severe asthma exacerbation
2. Understand the need for rapid treatment and reassessment of asthma

Technical:

1. Perform a rapid initial assessment
2. Perform peripheral IV line or IO placement
3. Perform airway management if patient clinically deteriorates

Behavioral:

1. Communicate clear leadership roles with delegation of roles
2. Perform early interventions for severe asthma exacerbation
3. Administer appropriate steroids for a patient with acute asthma exacerbation
4. Appropriate ventilator management (if indicated) for the asthmatic patient

**Learner responsible content:**

- Appendix I. Asthma Lecture

**Abbreviations**

AED = automatic external defibrillator

BP = blood pressure

Bpm = beats per minute

BVM = bag valve mask

CAP=community acquired pneumonia

CPR = cardiopulmonary resuscitation

EKG = electrocardiogram

EMS = emergency medical services

ETT=endotracheal tube

FH=family history

GCS = Glasgow Coma Scale

HPI=history of present illness

HR = heart rate

Hx=history

IO = intraosseous

IV = intravenous

LR = Ringer’s lactate

NS = normal saline

O2 = oxygen

PEA = pulseless electrical activity

PIV=peripheral intravenous line

PO=per ora, by mouth

Pt = patient

RCDP = rapid cycle deliberate practice

ROS=review of symptoms

RR = respiratory rate

RSI=rapid sequence intubation

O_2_Sat = oxygen saturation

T = temperature

**Tips for successful implementation:**

- It is beneficial to have various disciplines represented in each group, rather than having the nurses be in one group and physicians in another. It can help lead to more robust discussion.
- In general, participants should perform tasks that are in line with their discipline (ie, nurses performing nursing tasks, physician performing physician tasks) within the group. However, sometimes there will be participants who would like to experience a role outside of their usual role. This is allowable with the group’s support, and the participants learn about an experience other than their own lived experience.
- Some participants may be very confident and talkative, but because this is meant to be a group exercise, it may be useful to initially have any member of the group participate in any role, but then to selectively encourage those who are more reserved to take a role that will require noticeable interaction.
- Essentials of Debriefing and its Characteristics for facilitators (Abulebda, Auerbach, Limaiem, 2020):
  ○ **1. Ensuring psychosocial safety**
    ■ “Behave or perform without fear of negative consequences to self-image, social standing, or career trajectory.”
    ■ Essential to optimize learning outcomes by providing a supportive climate.
    ■ Should be conducted during the pre-simulation briefing and then during the debriefing.
  ○ **2. Having a debriefing stance or “basic assumption”**
    ■ Predefined basic assumption statement, “We believe that everyone participating in this simulation is intelligent, capable, cares about doing their best, and wants to improve.”
  ○ **3. Establishing debriefing rules**
    ■ A basic set of rules for debriefing among participants
    ■ Confidential discussion
    ■ Encourage all learners to participate actively
  ○ **4. Establishing a shared mental model**
    ■ Review the event details with input from the facilitator
  ○ **5. Addressing Key Learning Objectives**
    ■ Incorporate and analyze clear learning objectives during debriefing
  ○ **6. Using open-ended questions**
    ■ Helps facilitate discussion and foster reflection
    ■ Avoid close-ended or (yes/no) questions
  ○ **7. Using silence**
    ■ A brief period of silence after a facilitator asks a question
    ■ Promote an internal process within the debriefing learner’s mind
    ■ Allows learners to formulate their thoughts and analyze their mental frames
- Rapid cycle deliberate practice (RCDP) explanation to learners:
  ○ Each group member should treat the case with fidelity to the best of your ability
  ○ The facilitator (and assistant) will provide any necessary information for the case
  ○ There will be frequent stops where the facilitator will assess progress and provide feedback
  ○ Sometimes the facilitator will have the group re-perform the round if he/she deems necessary
  ○ Members of the group can and should change roles in between rounds to increase participation and educational vantage points

**Case Title:** Pediatric Asthma RCDP Practice Case

**Case Description & Diagnosis (short synopsis):** Daisy is brought to the A&E bed. She has unstable vital signs. She requires rapid nebulization, steroids, and respiratory support and will deteriorate if not treated quickly. If appropriate and time sensitive therapy occurs, she will have improvement of her vital signs and general appearance. If inappropriate or delayed interventions, the patient will decompensate to Pulseless Electrical Activity (PEA) arrest due to hypoxic respiratory failure.

**Equipment or props needed:**

Setup for All Rounds:

- Room configuration: typical A&E bed/stretcher (or table)
- For each group of 3 to 5 learners, one equipment setup includes the following:
  ○ A low-fidelity full-body simulation mannequin. If available, higher-fidelity mannequins can be used (we used a MegaCode Kid, and Laerdal ALS Baby mannequins)
  ○ An intravenous arm task trainer (if the mannequin is not equipped)
  ○ Lower extremity capable of intraosseous (IO) insertion (if mannequin is not equipped)
  ○ A medical resuscitation setup including the standard resuscitation equipment available in the A&E department (intravenous line starter kits, intravenous fluids, IO drill, IO needles, medical tape, nebulizer, bag valve mask, mock medications, etc.)
- Personnel:
  ○ Simulation instructor/debriefer
  ○ Confederates: patient’s grandmother (Angela), inpatient pediatrician
  ○ Demonstration items needed for debriefing: same as equipment

**How the Scenario Unfolds:**

**Ideal Scenario Flow:**

Daisy is brought to the A&E bed. The learners are expected to obtain a full set of vital signs and recognize that her vital signs are unstable. They should also perform an initial survey. Participants should be concerned for impending respiratory failure, and they should give medications and steroids early along with supplemental O2 and obtaining vascular access for labs and other potential medication administration. The learners should give an appropriate weight-based dose of albuterol and steroids rapidly and initiate adjuvant asthma medications or interventions (eg, magnesium, non-invasive positive pressure ventilation [NIPPV]) early.

They should be concerned for community acquired pneumonia as the source of respiratory failure if there is no response or improvement to initial asthma treatment and obtain and interpret a chest x-ray. They will also need to interpret the laboratory studies that they obtained.

After their interventions, they should reassess the patient including a reassessment of vital signs. If interventions are not appropriate and/or timely, she will progress to respiratory failure with or without pulseless electrical activity (PEA) due to acidosis and hypoxic respiratory failure. The learners should support her airway to their best ability (ie, bag valve mask, endotracheal intubation, etc.) and initiate CPR and/or defibrillation if the patient arrests. They should also reassess the physical exam to rule out a pneumothorax as the cause of PEA if the patient is intubated or being supported by bag-valve mask.

The participants should end the case with a handoff to the inpatient physician – summarizing the case using proper medical terminology.

**Critical actions:**

Round 1:

1. Rapid assessment of Airway, Breathing & Circulation
2. Check vital signs
3. Obtain IV/IO access
4. Recognize abnormal vital signs
5. Take basic history from the patient

Round 2:

1. Recognize impending respiratory failure (and etiology)
2. Give supplemental O2
3. Give albuterol
4. Give steroids
5. Consider adjuncts (magnesium, epinephrine, NIPPV
6. Re-assess after breathing treatment and steroid administration

Round 3:

1. Recognize continued tachypnea and increased work of breathing
2. Obtain labs
3. Obtain a chest x-ray
4. Give epinephrine IV/IO (1:10,000), magnesium if not already given
5. Start NIPPV (if available)
6. Recognize need to admit to hospital

Round 4:

1. Recognize continued tachycardia and tachypnea then slowed breathing
2. Airway intervention
3. Administer Rapid Sequence Intubation (RSI) or bag-valve mask for airway support
4. Reassessment of breath sounds to rule-out pneumothorax
5. Admission to intensive care unit

**Expected Endpoint of the Scenario:** maximum 10-minute time limit per round, but facilitator can stop at any point during scenario

**Possible Distractors Within Scenario**: high acuity, family members want to take patient home to care for them, family does not believe hospital will help their child

**Optional Challenges for Higher Level Learners**:

- Management of ventilator for asthmatic patients (permissive hypercapnea, prolonged I:E ratio, peak pressure monitoring)
- Considering other types of shock

**Roles of Participants/Trainees:** usual roles within the A&E

**Roles of Confederates (if applicable):**

Grandmother (Angela): provides medical history, primary caretaker of patient

Inpatient Physician: take handoff for transition of care from learners

**Anticipated Management Errors:**

1. Failure to recognize the impending respiratory arrest patient: Because this is a low-fidelity simulation and a simulated cardiac monitor is not used, learners may not realize that the patient is transitioning into arrest with slowing down her respiratory rate and taking shallower breaths with a decline of her mental status, and they often do not realize the deterioration in the vital signs. It is useful for the confederate to clearly indicate that there is an acute change in vital signs and that the patient has deterioration of vital signs even if the provider does not check.

**Case Title:** Pediatric Asthma RCDP Practice Case

**Chief complaint:** “I can’t breathe”[Table t23-jetem-6-2-c73]

**Table t23-jetem-6-2-c73:**

**Initial Vitals for all rounds:** HR 130	BP 110/50	RR 40	Temp 37 °C
SpO2 88%	Weight 30kg		

**Initial Physical Exam for all rounds:**

**General appearance:** She appears fatigued. Dry mucous membranes. Increased work of breathing, anxious.

**Initial Assessment:**

- **Airway/Breathing:** Airway open. Speaks in short 1–2-word sentences. Lungs with minimal air movement. Tachypneic. Occasionally coughing.
- **Circulation**: Tachycardic. No murmur, normal central and peripheral pulses. Capillary refill 1–2 seconds, extremities warm. Dry mucous membranes.
- **Mental Status:** Alert

**Case Title:** Pediatric Asthma RCDP Practice Case

**ROUND 1: Initial assessment of patient**

**Objectives introduced this round**:

1. Perform a rapid initial assessment
2. Perform peripheral IV placement

**Prompt for Team:** “A 7-year-old girl is brought to the A&E because she been having difficulty breathing. She has had a ‘cold’ for the past week.”

**Pertinent history (if asked):**

- **History of present illness:** The child has been having cough, congestion and running nose for the past week. Everything has been getting better except the cough, which has persisted. Today, she started acting sicker and has been wheezing at home.
  Grandmother Angela gave her albuterol inhaler this morning but has now run out. Her breathing became more labored and she seemed more anxious so was brought to A&E.
  No known sick contacts.
  Grandmother gave albuterol this morning. No other medications tried at home.
  She does not have fever, chills, nausea or vomiting. No diarrhea.
  Last bowel movement was last night and described as “normal.”
  Last urination was 5 hours ago.
  Immunizations are up to date.
- **ROS:**
  ○ Positive: fatigue, shortness of breath, cough, wheeze
  ○ Negative: fever, night sweats, weight loss, lymphadenopathy, ecchymosis, rash, vision change, rhinorrhea, sinus pain, epistaxis, dental problems, weight change, palpitations, syncope, edema, cyanosis, orthopnea, hemoptysis, nausea, vomiting, diarrhea, blood in stool, appetite change, abdominal pain, dysuria, frequency, urgency, hematuria, joint pain or swelling, muscle pain, back pain, headache, weakness
- **Medications:** albuterol
- **Past medical & surgical history:** Asthma, eczema
- **Family History**: Mother has asthma. Siblings have asthma and eczema. Grandfather has coronary artery disease and hypertension.
- **Social Hx:** Lives at home with her mother, father, grandfather, and younger sisters. She is in second grade.[Table t24-jetem-6-2-c73]

**Table t24-jetem-6-2-c73:**

**Vital signs** (need to be obtained): HR 130	BP 110/50	RR 40	Temp 37 °C	SpO2 88%
Weight 30kg				

**Physical exam findings (if asked):**

**Initial Assessment:**

- **Airway/Breathing:** Airway open. Speaks in short 1–2-word sentences. Lungs with minimal air movement. Tachypneic. Occasionally coughing.
- **Circulation**: Tachycardic. No murmur, normal central and peripheral pulses. Capillary refill 1–2 seconds, extremities warm. Dry mucous membranes.
- **Mental Status:** Alert, anxious

*No scenario progression yet.*

**End Round 1.**

**Round 1 Debriefing and Evaluation:**

Review the initial exam, reinforce the importance of rapid initial assessment, checking vital signs and obtaining IV/IO access in a critically ill patient.

Refer to expected actions and teaching points below:[Table t25-jetem-6-2-c73]

**Table t25-jetem-6-2-c73:**

Expected Action	Teaching Point	Result
Rapid assessment of Airway, Breathing, and Circulation	When presented with ill patient, assess circulation, airway and breathing and immediately intervene if emergent situation found	Patient is in impending respiratory failure
Check vital signs	Document initial vitals	
Obtain IV/IO access	Try PIV first, particularly if patient awake. IO if critically ill, particularly if altered mental status/coma	If re-doing case, can make PIV unattainable
Recognize abnormal vitals	HR for age: greater than 110 bpm is abnormal BP: less than 80/45 is considered hypotensive for child’s age RR greater than 22 is tachypneic for child’s age	
Take basic history from the patient and grandmother	History is important to management. Focus should be on the past medical history, surgical history, and medications. This will guide management. Try to obtain information while performing other tasks. This can be achieved with appropriate role delegation.	

**END SCENARIO (1)**

**Case Title:** Pediatric Asthma RCDP Practice Case

**ROUND 2: Initial management of impending respiratory failure**

**Objectives introduced this round:**

1. Recognize impending respiratory failure (and etiology)
2. Give supplemental O2
3. Give albuterol
4. Give steroids
5. Consider adjuncts (magnesium, epinephrine, NIPPV)
6. Re-assess after breathing treatment and steroid administration

**Restart scenario from beginning.**

**Prompt for team:** “A 7-year-old girl is brought to the A&E because she has been having difficulty breathing. She has had a “cold” for the past week.”

**Pertinent history (if asked):**

- **History of present illness:** The child has been having cough, congestion and running nose for the past week. Everything has been getting better except the cough, which has persisted. Today, she started acting sicker and has been wheezing at home.
  Grandmother Angela gave her albuterol inhaler this morning but has now run out. Her breathing became more labored and she seemed more anxious so was brought to A&E.
  No known sick contacts.
  Grandmother gave albuterol this morning. No other medications tried at home.
  She does not have fever, chills, nausea or vomiting. No diarrhea.
  Last bowel movement was last night and described as “normal.”
  Last urination was 5 hours ago.
  Immunizations are up to date.
- **ROS:**
  ○ Positive: fatigue, shortness of breath, cough, wheeze
  ○ Negative: fever, night sweats, weight loss, lymphadenopathy, ecchymosis, rash, vision change, rhinorrhea, sinus pain, epistaxis, dental problems, weight change, palpitations, syncope, edema, cyanosis, orthopnea, hemoptysis, nausea, vomiting, diarrhea, blood in stool, appetite change, abdominal pain, dysuria, frequency, urgency, hematuria, joint pain or swelling, muscle pain, back pain, headache, weakness
- **Medications:** albuterol
- **Past medical & surgical history:** Asthma, eczema
- **Family History**: Mother has asthma. Siblings have asthma and eczema. Grandfather has coronary artery disease and hypertension.
- **Social Hx:** Lives at home with her mother, father, grandfather, and younger sisters. She is in second grade.[Table t26-jetem-6-2-c73]

**Table t26-jetem-6-2-c73:**

**Vital signs** (need to be obtained): HR 130	BP 110/50	RR 40	Temp 37 °C	SpO2 88%
Weight 30kg				

**Physical exam findings (if asked):**

**Initial Assessment:**

- **Airway/Breathing:** Airway open. Speaks in short 1–2-word sentences. Lungs with minimal air movement. Tachypneic. Occasionally coughing.
- **Circulation**: Tachycardic. No murmur, normal central and peripheral pulses. Capillary refill 1–2 seconds, extremities warm. Dry mucous membranes.
- **Mental Status:** Alert, anxious

***No scenario progression yet.***

End Round 2.

**Expected actions after verbal prompt:**

□ Rapid assessment of ABC
□ Check vital signs, and recognize abnormal vitals
□ Obtain peripheral IV, or IO if unable
□ Take basic history from patient
□ Recognize impending respiratory failure and etiology and verbalize
□ Give albuterol, steroids
□ Consider adjuncts including magnesium, epinephrine, NIPPV
□ Reassess patient after each intervention

*No scenario progression yet.*

**END Round 2.**

**Round 2 Debriefing and Evaluation:**

Review initial exam, reinforce importance of rapid initiation of interventions in severe asthma exacerbation (emphasis on albuterol AND steroids), appropriate medication dosing for pediatric patients (eg, how many mg of albuterol based on weight, not just using the same dose for all pediatric patients), and immediate reassessment after interventions. Discuss learners’ differential diagnosis and why frequent reassessment is important in an acutely ill pediatric patient (eg, if not responding to albuterol, consider pneumonia)

Refer to expected actions and teaching points below:[Table t27-jetem-6-2-c73]

**Table t27-jetem-6-2-c73:**

Expected Action	Teaching Point	Result
Recognize impending respiratory arrest (& etiology)	In a tachypneic patient and silent chest, consider impending respiratory arrest and treat accordingly	If they do not treat aggressively (eg, only give nebs), will develop respiratory arrest
Give nebulized albuterol & supplemental O2	A reasonable goal for SpO2 is >93% Titrate to SpO2	Improved SpO2 to 94%
Give steroids	2 mg/kg within 1 hour of presentation Improved RR to 35	
Reassess after nebs/steroids	Always recheck vitals, perfusion, and respiratory status, and mental status after intervention	Improved vital signs, if they do not recheck again, patient will have respiratory arrest requiring airway support

**END SCENARIO (2)**

**Case Title:** Pediatric Asthma RCDP Practice Case

**ROUND 3: Assessment and Treatment of Respiratory Failure due to Asthma**

**Objectives introduced this round:**

1. Recognize continued tachypnea and increased work of breathing
2. Obtain labs
3. Obtain a chest x-ray
4. Give epinephrine, magnesium if not already given
5. Start NIPPV (if available)
6. Recognize need to admit to hospital

**Restart from beginning of scenario.**

**Prompt for team:** “A 7-year-old girl is brought to the A&E because she been having difficulty breathing. She has had a ‘cold’ for the past week.”

**Pertinent history (if asked):**

- **History of present illness:** The child has been having cough, congestion and running nose for the past week. Everything has been getting better except the cough, which has persisted. Today, she started acting sicker and has been wheezing at home.
  Grandmother Angela gave her albuterol inhaler this morning but has now run out. Her breathing became more labored and she seemed more anxious so was brought to A&E.
  No known sick contacts.
  Grandmother gave albuterol this morning. No other medications tried at home.
  She does not have fever, chills, nausea or vomiting. No diarrhea.
  Last bowel movement was last night and described as “normal.”
  Last urination was 5 hours ago.
  Immunizations are up to date.
- **ROS:**
  ○ Positive: fatigue, shortness of breath, cough, wheeze
  ○ Negative: fever, night sweats, weight loss, lymphadenopathy, ecchymosis, rash, vision change, rhinorrhea, sinus pain, epistaxis, dental problems, weight change, palpitations, syncope, edema, cyanosis, orthopnea, hemoptysis, nausea, vomiting, diarrhea, blood in stool, appetite change, abdominal pain, dysuria, frequency, urgency, hematuria, joint pain or swelling, muscle pain, back pain, headache, weakness
- **Medications:** albuterol
- **Past medical & surgical history:** Asthma, eczema
- **Family History**: Mother has asthma. Siblings have asthma and eczema. Grandfather has coronary artery disease and hypertension.
- **Social Hx:** Lives at home with her mother, father, grandfather, and younger sisters. She is in second grade.[Table t28-jetem-6-2-c73]

**Table t28-jetem-6-2-c73:**

**Vital signs** (need to be obtained): HR 130	BP 110/50	RR 40	Temp 37 °C	SpO2 88%
Weight 30kg				

**Physical exam findings (if asked):**

**Initial Assessment:**

- **Airway/Breathing:** Airway open. Speaks in short 1–2-word sentences. Lungs with minimal air movement. Tachypneic. Occasionally coughing.
- **Circulation**: Tachycardic. No murmur, normal central and peripheral pulses. Capillary refill 1–2 seconds, extremities warm. Dry mucous membranes.
- **Mental Status:** Alert, anxious

**Expected actions after verbal prompt:**

□ Rapid assessment of ABC
□ Check vital signs, and recognize abnormal vitals
□ Obtain peripheral IV, or IO if unable
□ Take basic history from patient
□ Recognize impending respiratory failure and etiology and verbalize
□ Give albuterol, steroids
□ Consider adjuncts including magnesium, epinephrine, NIPPV
□ Reassess patient after each intervention

**Scenario progression #1:** *After the first round of albuterol and steroids, HR 150, BP 120/70, RR 35, sat 90% on facemask/nasal cannula (88% on RA). T 37 °C.*

*The patient continues to have increased work of breathing and tachypnea.*

*Updated physical exam:*

*AIRWAY/BREATHING: Airway open. Speaks in short 1–2-word sentences. Lungs with occasional expiratory wheeze, slight improvement in air movement. Tachypneic.*

*CIRCULATION: Normotensive. Tachycardic. Capillary refill 1s. Hands are warm.*

**Expected actions after scenario progression:**

□ Recognize continued tachypnea and increased work of breathing
□ Give adjuncts (magnesium, epinephrine, NIPPV) if not already given
□ Obtain lab work
□ Obtain chest x-ray

**END Round 3.**

**Round 3 Debriefing and Evaluation:**

Review ongoing management including how you assess severity of asthma exacerbations (clinician gestalt, scoring tools such as PRAM or PASS), adjunct therapies for severe asthma exacerbations (magnesium, epinephrine/terbutaline, theophylline, NIPPV). Discuss need for reassessment when patient does not respond as expected to initial round of treatment and expected actions: broadening differential to include pneumonia, pneumothorax so obtaining imaging and/or bloodwork, using adjuvant therapies to stave off impending respiratory arrest. Providers should be able to advocate for an appropriate level of care for a critically ill patient. Severe asthma exacerbations require early interventions with albuterol, steroids, adjuncts for best outcomes.

Refer to expected actions and teaching points below:[Table t29-jetem-6-2-c73]

**Table t29-jetem-6-2-c73:**

Expected Action	Teaching Point	Result
Recognize continued tachypnea and increased work of breathing	Recheck vitals after every critical intervention. Consider labs, CXR and begin asthma adjunct therapies.	
Obtain labs	CBC with mild leukocytosis.	
Obtain a chest x-ray	Patient sent to radiology, not yet obtained	

**END SCENARIO (3)**

**Case Title:** Pediatric Asthma RCDP Practice Case

**ROUND 4: Recognition & Intervention for Respiratory Failure**

**Objectives introduced this round:**

1. Recognize impending respiratory failure
2. Airway support
3. Admission to intensive care unit

**Restart from beginning of scenario.**

**Prompt for team:** “A 7-year-old girl is brought to the A&E because she been having difficulty breathing. She has had a ‘cold’ for the past week.”

**Pertinent history (if asked):**

- **History of present illness:** The child has been having cough, congestion and running nose for the past week. Everything has been getting better except the cough, which has persisted. Today, she started acting sicker and has been wheezing at home.
  Grandmother Angela gave her albuterol inhaler this morning but has now run out. Her breathing became more labored and she seemed more anxious so was brought to A&E.
  No known sick contacts.
  Grandmother gave albuterol this morning. No other medications tried at home.
  She does not have fever, chills, nausea or vomiting. No diarrhea.
  Last bowel movement was last night and described as “normal.”
  Last urination was 5 hours ago.
  Immunizations are up to date.
- **ROS:**
  ○ Positive: fatigue, shortness of breath, cough, wheeze
  ○ Negative: fever, night sweats, weight loss, lymphadenopathy, ecchymosis, rash, vision change, rhinorrhea, sinus pain, epistaxis, dental problems, weight change, palpitations, syncope, edema, cyanosis, orthopnea, hemoptysis, nausea, vomiting, diarrhea, blood in stool, appetite change, abdominal pain, dysuria, frequency, urgency, hematuria, joint pain or swelling, muscle pain, back pain, headache, weakness
- **Medications:** albuterol
- **Past medical & surgical history:** Asthma, eczema
- **Family History**: Mother has asthma. Siblings have asthma and eczema. Grandfather has coronary artery disease and hypertension.
- **Social Hx:** Lives at home with her mother, father, grandfather, and younger sisters. She is in second grade.[Table t30-jetem-6-2-c73]

**Table t30-jetem-6-2-c73:**

**Vital signs** (need to be obtained): HR 130	BP 110/50	RR 40	Temp 37 °C	SpO2 88%
Weight 30kg				

**Physical exam findings (if asked):**

**Initial Assessment:**

- **Airway/Breathing:** Airway open. Speaks in short 1–2-word sentences. Lungs with minimal air movement. Tachypneic. Occasionally coughing.
- **Circulation**: Tachycardic. No murmur, normal central and peripheral pulses. Capillary refill 1–2 seconds, extremities warm. Dry mucous membranes.
- **Mental Status:** Alert, anxious

**Expected actions after verbal prompt:**

□ Rapid assessment of ABC
□ Check vital signs, and recognize abnormal vitals
□ Obtain peripheral IV, or IO if unable
□ Take basic history from patient
□ Recognize impending respiratory failure and etiology and verbalize
□ Give albuterol, steroids
□ Consider adjuncts including magnesium, epinephrine, NIPPV
□ Reassess patient after each intervention

**Scenario progression #1:** *After the first round of albuterol and steroids, HR 150, BP 120/70, RR 35, sat 90% on facemask/nasal cannula (88% on RA). T 37 °C.*

*The patient continues to have increased work of breathing and tachypnea.*

*Updated physical exam:*

*AIRWAY/BREATHING: Airway open. Speaks in short 1–2-word sentences. Lungs with occasional expiratory wheeze, slight improvement in air movement. Tachypneic.*

*CIRCULATION: Normotensive. Tachycardic. Capillary refill 1s. Hands are warm.*

**Expected actions after scenario progression:**

□ Recognize continued tachypnea and increased work of breathing
□ Give adjuncts (magnesium, epinephrine, NIPPV) if not already given
□ Obtain lab work
□ Obtain chest x-ray

**Scenario progression #2:** *You are called to radiology where the patient is now becoming very sleepy with short, shallow breaths. When you bring her quickly back to A&E, she opens her eyes to voice and her oxygen saturation is 85% on room air, 88% on facemask.*

*Updated physical exam:*

*AIRWAY/BREATHING: No air movement, silent chest. Bradypnea.*

*CIRCULATION: Tachycardic, normotensive. Capillary refill <3s. Warm hands.*

*MENTAL STATUS: Only responds to voice.*

**Expected actions after scenario progression:**

- Providers who are not skilled in endotracheal intubation should provide respiratory support by demonstrating BVM
- Providers who are skilled in endotracheal intubation should:
  ○ Prepare for rapid sequence intubation
    ■ Name which medication to be used and reasoning
    ■ Name what equipment should be available (including endotracheal tube & laryngoscope size)
    ■ Discuss initial ventilator settings for a severe asthmatic
  ○ Intubate patient
  ○ Post-intubation
    ■ Endotracheal tube placement confirmation
- Admit patient to the pediatric intensive care unit

**END Round 4.**

**Round 4 Debriefing and Evaluation:**

Review ongoing management. Providers should be able to advocate for an appropriate level of care for a critically ill patient. Respiratory failure requires supporting the patient’s breathing which can be as minimal as bag/valve/mask or as invasive as intubation.

Refer to expected actions and teaching points below:[Table t31-jetem-6-2-c73]

**Table t31-jetem-6-2-c73:**

Expected Action	Teaching Point	Result
Recognize impending respiratory failure	Recheck vitals after every intervention. Bag mask ventilation must be started to address bradypnea and worsening oxygenation.	Improved oxygenation to 94% but continued bradypnea. If no bag mask ventilation done, patient can go into cardiac arrest and provider will have to provide CPR.
Bag valve mask	Practice correct BVM technique in patient with respiratory failure	Improved O2 sat, improved RR
Prepare for rapid sequence intubation	Be comfortable with medications used for rapid sequence intubation in pediatric patients.	
Endotracheal tube placement confirmation	There are several ways to confirm endotracheal tube placement. Provider should be able to list several.	If ETT placement not confirmed, patient should have persistent hypoxia and unequal breath sounds on exam (increased on the right for possible right mainstem intubation).
Admission to hospital	High risk patient intubated for respiratory failure needs to be admitted to the intensive care unit with continuous monitoring and antibiotics with respiratory support	

**END SCENARIO (4)**

**Case Title:** Pediatric Asthma RCDP Practice Case

**ROUND 5: Full scenario**

If time allows, restart from the beginning of the scenario and run the full scenario without interruption. If initial actions are performed well, can restart where needed.

**END OF RCDP Session.**

**Final debriefing and feedback**

- Praise learners for tasks accomplished well.
- Provide areas for continued improvement.

*Complete Blood Count* [Table t32-jetem-6-2-c73]

**Table t32-jetem-6-2-c73:**

White blood count (WBC)	11.7 × 1000/mm3
Hemoglobin (Hgb)	13 g/dL
Hematocrit (HCT)	38%
Platelets (Plt)	315 ×1000/mm3

**Table t33-jetem-6-2-c73:**

Segmented Neutrophils	55 K/μL
Bands	0.5%
Lymphocytes	35 K/μL

[Table t33-jetem-6-2-c73]

## Appendix L Airway and Intubation Lecture

Please see associated PowerPoint file

## Appendix M Airway and Intubation Lecture Synopsis

**Target Audience:**
Generalist healthcare providers in low- and middle-income countries
**Educational Methods:**
PowerPoint didactic lecture
**Time required for implementation:**
About 60 minutes
**Learning Objectives:**
The learner will be able to:

1. Identify indications for intubation.
2. Describe the differences of a pediatric airway compared to an adult airway.
3. Identify equipment used for airway support.
4. List the “P’s” of intubation.
5. Recognize complications associated with RSI and contraindications to common RSI medications.

**Equipment/Environment:**

- A large room (with a capacity of at least 50 people) with multiple tables and ample floor space, or multiple rooms if available
- A computer with PowerPoint capability and projector setup
- Personnel needed: one lecturer

**Recommended pre-reading for instructor:**

- Santillanes G, Gausche-Hill M. Pediatric Airway Management. *Emergency Medicine Clinics of North America*. 2008;26(4):961–975.[Table t34-jetem-6-2-c73]

### Lecture Script

**Table t34-jetem-6-2-c73:**

Slide 1	
Slide 2	This is an opportunity to discuss how a practitioner would approach airway management before being presented with the items in the lecture.
Slide 3	This is an opportunity to discuss how a practitioner would approach airway management before being presented with the items in the lecture.
Slide 4	
Slide 5	The pediatric tongue is large, can easily lose tone and cause upper airway obstruction. Adewale L. Anatomy and assessment of the pediatric airway. *Pediatric Anesthesia*. 2009;19:1–8.
Slide 6	The pediatric larynx is higher (more superior) and more anterior compared to an adult airway. With age, the larynx begins to resemble adult anatomy. - Infant larynx is closer to the C1 level. - At about 6 months, the larynx is closer to the C3 level. - The larynx is at about the level of C4–C6 in older children and adults. An adult airway is more shaped like a pipe, while a pediatric airway is more shaped like a cone. The narrowest point of the pediatric airway is at the cricoid cartilage (the subglottic area of the larynx). The narrowest part of the adult airway is at the vocal cords. Adewale L. Anatomy and assessment of the pediatric airway. *Pediatric Anesthesia*. 2009;19:1–8.
Slide 7	The pediatric epiglottis appears larger in size, more “omega (Ω)” in shape, and floppier (due to lack of cartilage) compared to an adult. Adewale L. Anatomy and assessment of the pediatric airway. *Pediatric Anesthesia*. 2009;19:1–8.
Slide 8	There are multiple interventions that can be made to assist a child’s respiratory status. Some interventions are more invasive and higher risk than other measures.
Slide 9	Positioning the child is a non-invasive measure that can support a child. Position of comfort means that the child should be left in whatever position that keeps the child from becoming more agitated (ie, in parent’s arms, left alone in bed, in child carrier, etc.). If the child will allow, consider: - The sniffing position in children older than age 2 - Placing a rolled towel under the back at the level of the shoulders - Chin lift (contraindicated if a possible c-spine injury) - Jaw thrust
Slide 10	Suctioning is a means to help a young child clear accumulating secretion. Infants do not have an ability to generate a strong cough. They also have smaller airways which makes removing tracheal secretions important to ensure that the airway stays patent. Infants are obligate nose breathers. Suctioning helps to clear the nose if there are copious secretions. Many an infant was saved from more aggressive airway interventions by simple suctioning.
Slide 11	Oxygen can be administered in different volumes and concentrations.
Slide 12	High flow nasal cannula is being used with more frequency in cases of respiratory distress in the pediatric emergency department. It is uniquely different from nasal cannula in that it utilizes warmed and humidified oxygen at higher flow rates and higher oxygen concentration rates. Slain KN, Shein SL, Rotta AT. The use of high-flow nasal cannula in the pediatric emergency department. *J Pediatr (Rio J)*. 2017.
Slide 13	Nasal airway (also called nasopharyngeal airway or nasal trumpet) Slips into the nose to the upper pharynx to assist in oxygenation.
Slide 14	Oropharyngeal airway (OPA) is used to help lift the tongue which can cause upper airway obstruction. This can help facilitate oxygenation and ventilation. The OPA can induce a gag reflex and should only be used if a patient is not conscious.
Slide 15	Bag-valve mask (BVM) ventilation is a means to augment respirations and oxygenation in a patient with respiratory failure. Ventilation can occur without using oxygen supplementation, but oxygenation requires at least 10 liters per minute of O2 to fill the oxygen reservoir (provides 100% O2).
Slide 16	Intubation is the only way to secure a patent airway and deliver 100% O2. However, intubation is one of the most invasive methods of airway management.
Slide 17	Intubation is a higher-risk procedure. The P’s of intubation offer a mnemonic to remember the many aspects to address having a successful intubation.
Slide 18	Plan and Prepare Suction - at least one working suction should be ready Oxygen - a non-rebreather facemask and BVM should be attached to 10–15L/min of O2 - nasal prongs may be attached at the same time for apneic oxygenation Airways - test balloon on endotracheal tube to ensure there is not a leak of air Stylet - placed inside ET tube for rigidity - ensure that the stylet does not extend past the distal opening of the ET tube Monitoring equipment/Medications - cardiac monitor, pulse ox, BP cuff opposite arm with IV medications drawn up and ready to be given
Slide 19	
Slide 20	If any suspicion of cervical spine injury, use measures to avoid manipulating the cervical spine: - Manual in-line stabilization: from the head of the bed, the hands should cradle the occiput and mastoid processes - Manual in-line stabilization: from the side of the bed to allow intervention from the head of the bed - Spine board + sandbags + tape - Semirigid collars
Slide 21	Position the child in a way that will optimize the airway being open or keep the child from becoming increasingly agitated. Covered more in detail in Slide 10
Slide 22	Pre-oxygenate - non-rebreather facemask at 15 L/min - nasal cannula can provide passive oxygenation without ventilation; avoid using BVM unless the patient has severely depressed respiratory rate or poor inspiratory effort (BVM fills the stomach with air and increases risk for aspiration)
Slide 23	In adult populations, there has been increased use of “apneic oxygenation” using nasal cannula at higher flow rates to passively deliver oxygen. Brainard A, Chuang D, Zeng I, Larkin GL. A Randomized Trial on Subject Tolerance and the Adverse Effects Associated with Higher- versus Lower-Flow Oxygen Through a Standard Nasal Cannula. *Annals of Emergency Medicine*. 2015;65(4):356–361. - adults have been shown to tolerate passive oxygenation well in the short term Vourc’h M, Asfar P, Volteau C, et al. High-flow nasal cannula oxygen during endotracheal intubation in hypoxemic patients: a randomized controlled clinical trial. *Intensive Care Medicine*. 2015;41(9):1538–1548. - HFNC did not reduce the lowest level of desaturation - used HFNC versus facemask Aziz M. Faculty of 1000 evaluation for Transnasal Humidified Rapid-Insufflation Ventilatory Exchange (THRIVE): a physiological method of increasing apnoea time in patients with difficult airways. *F1000 - Post-publication peer review of the biomedical literature*. 2017. - increased apnea times without desaturation (<90%) used HFNC with jaw thrust - A case series of 25 patients with difficult airways undergoing general anesthesia for hypopharyngeal or laryngotracheal surgery had mean apnea times of 14 minutes without desaturation (ie, SaO2 >90%) - Studies showing efficacy in children are still needed
Slide 24	
Slide 25	Pretreatment is sometimes performed in preparation for optimizing RSI. The listed agents are the most commonly administered pretreatment medications. They can be used individually or in combination.
Slide 26	Atropine traditionally is the most commonly administered pediatric pretreatment medication, especially for young children. There has been newer literature to suggest that atropine may no longer be necessary as a routine pretreatment medication. Bean A, Jones J. Atropine: Re-evaluating its use during paediatric RSI. *Emergency Medicine Journal*. 2007;24(5):361–362.
Slide 27	Lidocaine is often given as a pretreatment to blunt the increase in intracranial pressure. Robinson N. In patients with head injury undergoing rapid sequence intubation, does pretreatment with intravenous lignocaine/lidocaine lead to an improved neurological outcome? A review of the literature. *Emergency Medicine Journal*. 2001;18(6):453–457.
Slide 28	The basic equation to understand RSI (rapid anesthesia usually performed in the acute care setting) is medications to achieve sedation and paralysis.
Slide 29	The medications listed are often used to achieve the sedation portion of RSI.
Slide 30	Ketamine is a dissociative medication that is controversial because traditionally it has been thought to cause increased intracranial pressure (ICP) and intraocular pressure (IOP). However, though there may be some increases in the ICP and IOP measured values, negative effects on morbidity and mortality are not routinely documented.
Slide 31	Paralysis is achieved by neuromuscular blockade. Typical medications are succinylcholine, rocuronium, and vecuronium. Vecuronium has a significantly longer half-life and so its use is less frequent in the acute care setting.
Slide 32	
Slide 33	The two most common laryngoscope blades used in the acute care setting are the Macintosh and the Miller blades. There are subtle differences in the technique used depending on the laryngoscope blade.
Slide 34	This slide gives a cursory overview of the intubation procedure.
Slide 35	Provided is a simple equation to estimate the size of endotracheal tube to use based on a child’s age (if older than 2 years). The other equations listed can help determine the sizing of other materials used on a pediatric patient based on age.
Slide 36	After intubation, a practitioner must ensure that the placement was appropriate. There are multiple ways to confirm placement, but the best methods would be to obtain continuous capnometry and evaluate a chest x-ray.
Slide 37	
Slide 38	This is an opportunity to discuss how a practitioner would approach airway management after the items presented in the lecture.
Slide 39	This is an opportunity to discuss how a practitioner would approach airway management after the items presented in the lecture.
Slide 40	End

## Appendix N Airway Lab

**Target Audience:**
Generalist healthcare providers in low- and middle-income countries
**Educational Methods:**
Procedural medical simulation
**Time required for implementation:**
About 60 minutes
**Learning Objectives:**
The learner will be able to:

1. Implement initial maneuvers to manage a compromised airway.
2. Demonstrate knowledge of indications and contraindications for basic airway management.
3. Demonstrate effective use of appropriate equipment needed to effectively manage a basic airway emergency.

**Equipment/Environment/Personnel:**

- A large room (with a capacity of at least 50 people) with multiple tables and ample floor space, or multiple rooms if available
- 3–5 learners per station:
  ○ 1 full medical simulation mannequin or bust capable of airway manipulation
    ■ Try to have multiple sizes of mannequins available for the lab (ie, infant, child, adolescent)
  ○ Oral pharyngeal airways of multiple sizes
    ■ 3–5 airways of different sizes per station
  ○ Nasopharyngeal airways of multiple sizes
    ■ 3–5 airways of different sizes per station
  ○ Bag valve mask
    ■ 2–3 bag valve masks of different sizes per station
  ○ Lubricant (must be safe for use with mannequins)
- One facilitator per station of 3 to 5 learners

**Recommended pre-reading for instructor:**

- Santillanes G, Gausche-Hill M. Pediatric Airway Management. *Emergency Medicine Clinics of North America*. 2008;26(4):961–975.

**Abbreviations:**

HR – Heart Rate

RR – Respiratory Rate

BP – Blood Pressure

T – Temperature

O2 Sat – Oxygen Saturation

IV - intravenous

**Tips for successful implementation:**

- The facilitator should guide the learners through each of the procedures listed (**in bold**).
- The learners should take turns demonstrating each of the procedures listed.
- The learners should be able to explain the indications and contraindications of the procedures listed.
- The learners should be coached in how to troubleshoot difficulty with any of the procedures listed.
- It is beneficial to have various disciplines represented in each group, rather than having the nurses be in one group and physicians in another. It can help lead to more robust discussion and feedback.

### Airway Positioning

#### Head-Tilt, Chin-Lift

- Use if upper airway obstruction due to collapsed soft tissue structures is suspected
- Use fingers to lift the chin up and backward lifting at the bony surface of the jaw
- Do not use this maneuver if c-spine injury is suspected

#### Jaw Thrust

- Use if upper airway obstruction due to collapsed soft tissue structures is suspected
- Use the index and middle fingers to push the lower jaw anteriorly at the angle of the mandible while bracing the face with the thumbs on the face

### Airway Adjuncts

#### Nasopharyngeal Airway (NPA, “Nasal Trumpet”)

Device that provides a patent airway from the nares to the pharynx.

Learner should be able to state indications

- The soft tissue structures of the upper airway are causing obstruction at the upper airway
- Difficulty oxygenating or ventilating a patient

Learner should be able to state contraindications

- An NPA should not be used in patients with significant facial trauma (ie, obvious facial fractures)
- Do not use an NPA if it requires any force

Learner should be able to determine an appropriate-sized NPA

- Measure from the tip of the patient’s earlobe to the tip of the patient’s nose.

Learner should be able to demonstrate appropriate placement of the NPA

- Lubricate the NPA
- Keep the patient’s head in a neutral position. Insert the airway, bevel toward the nasal septum straight into the face (not angling cranially). The end of the NPA should rest against the opening of the nare.

#### Oropharyngeal Airway (OPA)

Device fits over the tongue to hold the soft structures of the upper airway away from the posterior pharyngeal wall.

Learner should be able to state indications

- The soft tissue structures of the upper airway are causing obstruction at the upper airway
- Difficulty oxygenating or ventilating a patient

Learner should be able to state contraindications

- An OPA should not be used in conscious individuals (ie, those who still have a cough or gag reflex). Otherwise it can stimulate vomiting, laryngeal spasm, or aspiration.

Learner should be able to determine an appropriate-sized OPA

- Measure from the corner of the mouth to the earlobe
- An OPA that is too large can damage the throat. An OPA that is too small can potentially act as a foreign body obstruction.

Learner should be able to demonstrate appropriate placement of the OPA

- Open the mouth (consider the “scissors” finger technique)
- Insert the OPA without pushing the tongue back
  ○ Clear the mouth of blood/secretions
  ○ One can use a tongue depressor to move the tongue from the way
  ○ One can insert the OPA upside down (tip facing the roof of the patient mouth), then as the airway is inserted, rotate 180 degrees to proper position

### Bag Valve Mask

Bag Valve Mask (BVM) devices are used when there is respiratory failure. Effective BVM use can deliver effective ventilation and can provide up to 100% oxygenation.

Learner should be able to state indications

- When there is respiratory failure
- As a bridge to an advanced airway device

Learner should be able to determine an appropriate-sized BVM

- The mask should cover both the mouth and the nose, but should not extend beyond the chin
- The mask should not significantly encroach on the eyes
- When ventilating with the BVM, there should not be a loud air leak and there should be a significant chest rise

Learner should be able to demonstrate appropriate use of a BVM

- Position the patient in the “sniffing” position, to open the airway
- Practice using with an OPA or an NPA
- The face should be drawn up to the mask, rather than pushing the mask into the face
- One-Person
  ○ The nondominant hand should be used to hold the mask onto the face using the “E-C” technique
    ■ Care must be taken particularly with pediatric patients to not compress the soft tissue under the jaw
- Two-Person
  ○ One person will hold the mask to the face using two hands. Each thumb and index finger should create a “C” shape with the other fingers pulling the jaw toward the mask at the rim of the mandible
- Each squeeze of the bag should be delivered over about 1–2 seconds
- The air in the bag does not need to be emptied
- Check for effective ventilations
  ○ Observe for adequate rise and fall of the patient’s chest
  ○ Observe for improvement in the patient’s color and oxygen saturation
  ○ Listen and feel for an air leak round the mask

## Appendix O Post-Test Questions

1. Which is true regarding the use of ipratropium in asthma management?
  - If a patient responded well to ipratropium for status asthmaticus, he should be discharged to home with a prescription for ipratropium.
  - Ipratropium has never been proven to be of benefit in patients with acute asthma exacerbations
  - Ipratropium is most useful as an adjunct for patients with severe asthma exacerbations
  - Ipratropium is useful by itself as a bronchodilator in the treatment of acute asthma exacerbations
  - The main benefit of ipratropium instead of other anticholinergic drugs is its rapid onset of action
2. What size cuffed endotracheal tube would you request for a 4-year-old child?
  - 3.5
  - 4
  - 4.5
  - 5
  - 6
3. A 4-month-old female with a history of prematurity presents to the ED with cough, nasal congestion, and fever of the past 3 days. The infant’s respiratory rate is 80, severe chest indrawing (retractions) and diffuse crackles on lung exam. She is otherwise ill in appearance.
  What should be the providers’ first step?
  - Albuterol inhaler
  - Oral corticosteroids
  - Perform rapid sequence intubation
  - Supplemental oxygen
  - Epinephrine injection
4. Which of the following statements regarding pneumonia in children is true?
  - Blood cultures frequently reveal the cause of pneumonia in children
  - Cough is the best single predictor of pneumonia
  - Cough is the most prominent symptom in neonates with pneumonia
  - Dehydration is the most common systemic complication
  - Mycoplasma pneumoniae is the most common cause of bacterial pneumonia
5. Regarding the use of corticosteroids in asthma management, which statement is true?
  - IV corticosteroids have been proven more effective than oral corticosteroids
  - Inhaled corticosteroids are not useful for long-term asthma management
  - Long-term systemic corticosteroid use may be complicated by weight gain, aseptic necrosis of long bones, and peptic ulcer disease (PUD)
  - Patients who require steroids upon discharge to home require tapered oral corticosteroids for 10 or more days
  - The onset of action for IV corticosteroids is within 1 hour
6. Which of the following statements regarding the pediatric airway as compared with the adult airway is true?
  - A child has a smaller tongue relative to the size of the oral cavity
  - An infant’s epiglottis is relatively short and thicker
  - In children younger than 10 years, the narrowest portion of the airway is below the vocal cords
  - The vocal cords in infants are omega-shaped
7. A 10-year-old girl presents with altered mental status. GCS is 8 on initial evaluation in the A&E. Please match the RSI (rapid sequence intubation) pretreatment with the correct indication for administration.
  - Atropine – to prevent bradycardia
  - Etomidate – to promote adrenal stimulation
  - Fentanyl – to decrease secretions
  - Lidocaine – to cause dissociation
8. Aminophylline (theophylline) is prescribed for a patient with asthma. A nurse administers the medication, knowing that the primary action of this medication is to:
  - Improve PEEP (positive end-expiratory pressure) to the lungs
  - Prevent infection
  - Promote expectoration
  - Reduce pain associated with cough
  - Relax smooth muscles
9. A previously healthy 3-year-old male presents to the A&E for evaluation of fever and cough for 3 days. He has a decreased appetite but taking fluids and does not appear dehydrated. In triage, his T 39.5 °C, HR 120, RR 36, SpO2 96% on room air. On exam, he is alert and ambulatory with no distress in his breathing. Diminished breath sounds are noted in the right base.
  The next appropriate step would be:
  - A call to the pediatrician for admission
  - Immediate CBC (complete blood count), blood culture, and ABG (arterial blood gas)
  - Immediate IV placement for fluids and antibiotics
  - Inspiratory/expiratory radiographs
  - Oral antibiotics and discharge home
10. Which choice would be the most appropriate antibiotic(s) to start for the above case?
  - Amoxicillin
  - Azithromycin
  - Ceftriaxone
  - Oseltamivir
  - Trimethoprim-sulfamethoxazole
11. An 8-month-old infant born at term is brought in for cough, rhinorrhea, and congestion for three days. Temperature at home was 101 F. Her past medical history and birth history are unremarkable. Vital signs are T 100.1 °F, HR 129, RR 42, BP 90/60, and SpO2 98% on room air. The patient is smiling and active and tachypneic. There is scant wheezing and mild retractions.
  What is true regarding this patient’s condition?
  - Albuterol therapy has been shown to reduce 30-day mortality
  - Corticosteroids reduce the hospitalization rates
  - Respiratory syncytial virus (RSV) is the most common cause
  - Ribavirin is indicated
  - The patient has a 75% chance of developing asthma as a child
12. Which of the following is true regarding selection of agents for rapid sequence intubation (RSI)?
  - Ketamine is known to be safely used in a patient with a ruptured globe.
  - Neuromuscular blocking agents are contraindicated in patients who are having seizures.
  - Rocuronium at a dose of 1–1.2 mg/kg achieves similar intubating conditions as succinylcholine 1.5–2mg/kg for emergent RSI in children.
  - Succinylcholine would be recommended for use in a child with glomerulonephritis and the following labs (mEq/L): Na 138, K 5.8, Cl 101, HCO3 17.
13. A 5-year-old girl presents for fever for the past 2 days and now is lethargic and has BP 70/50 and HR 150 at triage. Which may improve perfusion in a patient receiving sedation during RSI for septic shock?
  - Etomidate
  - Ketamine
  - Midazolam
  - Thiopental
14. A 3-month-old male (born at 38 weeks gestational age) comes to the emergency room with a history of upper respiratory symptoms for 3 days, fever, decreased appetite and increased work of breathing. On physical exam his vitals are T 99 °F, RR 55, HR 185, BP 90/65, and Sat 96% on room air; he has nasal congestion, mild retractions, and coarse breath sounds. He has an older sister in primary school.
  Of the following, the MOST appropriate next step in management is:
  - Give oral corticosteroid
  - Prescription for nasal decongestants
  - Start albuterol
  - Suction
  - Supplementary oxygen
15. A mother brings her 6-month-old male infant to your office. She reports that her son has been breathing faster than usual for the past 2 days, and she has noted occasional wheezing. She states that prior to the difficulty breathing, she noticed some clear nasal discharge for several days. The infant was born full-term, with no complications, and no significant medical history. Vital signs are T 38 °C, BP 100/60 mmHg, HR 120 bpm, RR 40 rpm, SpO2 95%. Physical exam reveals expiratory wheezing and crackles, and intercostal retractions are noted. Which of the following is the most appropriate next step in management?
  - CT scan of the chest
  - Inhaled fluticasone
  - Intubation
  - Non-invasive supportive care
  - Oral amoxicillin
16. All the following patients are at high risk for morbidity and mortality from RSV infection EXCEPT:
  - A 1-month old child with an unrepaired congenital heart disease
  - A 2-month old with cerebral palsy
  - A 4-month old who was born at 32 weeks gestational age with bronchopulmonary dysplasia (BPD)
  - A full-term infant with hyperbilirubinemia
  - A premature infant
17. Which of the following medications is the most effective in the immediate treatment of an acute asthma exacerbation?
  - Inhaled corticosteroids
  - IV ketamine
  - IV magnesium
  - Nebulized albuterol or salbutamol
  - Nebulized saline
18. A previously healthy 4-year-old girl is brought into the A&E for concern for fever. After taking a complete history and performing a physical exam, you have diagnosed her with community acquired pneumonia. Which of the following does NOT necessitate inpatient admission?
  - Decreased oral intake
  - Incomplete immunization profile
  - Lethargy
  - Oxygen requirement
  - Persistent tachypnea
19. A 12-year-old boy presents with an asthma exacerbation. Over several hours, he is given oral steroids and several nebulized albuterol and ipratropium bromide treatments. Vital signs are P 110, RR 20, O2 saturation 95%, PEFR (peak expiratory flow rate) 250L/min (40% of his normal). He can speak in full sentences but is still wheezing. What should be the next step in his management?
  - Continue albuterol and consider admission
  - Discharge on albuterol and prescription for corticosteroids
  - Give subcutaneous epinephrine and reassess
  - Give subcutaneous terbutaline and admit to the ICU
  - Initiate noninvasive positive pressure
20. Which of the following is most true regarding pediatric community-acquired pneumonia (CAP)?
  - It is easier to differentiate between typical and atypical pneumonia in pediatric patients
  - Streptococcus pneumoniae is the most commonly isolated organism in children aged 5 years to 15 years
  - The incidence of CAP in children younger than 5 years old is higher than in middle-aged adult smokers
  - The most common cause of pneumonia in the neonate is Mycoplasma pneumoniae

## Appendix P Post-Test Answers

1. Which is true regarding the use of ipratropium in asthma management?
  - If a patient responded well to ipratropium for status asthmaticus, he should be discharged to home with a prescription for ipratropium.
  - Ipratropium has never been proven to be of benefit in patients with acute asthma exacerbations
  - **Ipratropium is most useful as an adjunct for patients with severe asthma exacerbations**
  - Ipratropium is useful by itself as a bronchodilator in the treatment of acute asthma exacerbations
  - The main benefit of ipratropium instead of other anticholinergic drugs is its rapid onset of action
2. What size cuffed endotracheal tube would you request for a 4-year-old child?
  - 3.5
  - 4
  - 4.5
  - **5**
  - 6
3. A 4-month-old female with a history of prematurity presents to the ED with cough, nasal congestion, and fever of the past 3 days. The infant’s respiratory rate is 80, severe chest indrawing (retractions) and diffuse crackles on lung exam. She is otherwise ill in appearance.
  What should be the providers’ first step?
  - Albuterol inhaler
  - Oral corticosteroids
  - Perform rapid sequence intubation
  - **Supplemental oxygen**
  - Epinephrine injection
4. Which of the following statements regarding pneumonia in children is true?
  - Blood cultures frequently reveal the cause of pneumonia in children
  - Cough is the best single predictor of pneumonia
  - Cough is the most prominent symptom in neonates with pneumonia
  - **Dehydration is the most common systemic complication**
  - Mycoplasma pneumoniae is the most common cause of bacterial pneumonia
5. Regarding the use of corticosteroids in asthma management, which statement is true?
  - IV corticosteroids have been proven more effective than oral corticosteroids
  - Inhaled corticosteroids are not useful for long-term asthma management
  - **Long-term systemic corticosteroid use may be complicated by weight gain, aseptic necrosis of long bones, and peptic ulcer disease (PUD)**
  - Patients who require steroids upon discharge to home require tapered oral corticosteroids for 10 or more days
  - The onset of action for IV corticosteroids is within 1 hour
6. Which of the following statements regarding the pediatric airway as compared with the adult airway is true?
  - A child has a smaller tongue relative to the size of the oral cavity
  - An infant’s epiglottis is relatively short and thicker
  - **In children younger than 10 years, the narrowest portion of the airway is below the vocal cords**
  - The vocal cords in infants are omega-shaped
7. A 10-year-old girl presents with altered mental status. GCS is 8 on initial evaluation in the A&E. Please match the RSI (rapid sequence intubation) pretreatment with the correct indication for administration.
  - **Atropine – to prevent bradycardia**
  - Etomidate – to promote adrenal stimulation
  - Fentanyl – to decrease secretions
  - Lidocaine – to cause dissociation
8. Aminophylline (theophylline) is prescribed for a patient with asthma. A nurse administers the medication, knowing that the primary action of this medication is to:
  - Improve PEEP (positive end-expiratory pressure) to the lungs
  - Prevent infection
  - Promote expectoration
  - Reduce pain associated with cough
  - **Relax smooth muscles**
9. A previously healthy 3-year-old male presents to the A&E for evaluation of fever and cough for 3 days. He has a decreased appetite but taking fluids and does not appear dehydrated. In triage, his T 39.5 °C, HR 120, RR 36, SpO2 96% on room air. On exam, he is alert and ambulatory with no distress in his breathing. Diminished breath sounds are noted in the right base.
  The next appropriate step would be:
  - A call to the pediatrician for admission
  - Immediate CBC (complete blood count), blood culture, and ABG (arterial blood gas)
  - Immediate IV placement for fluids and antibiotics
  - Inspiratory/expiratory radiographs
  - **Oral antibiotics and discharge home**
10. Which choice would be the most appropriate antibiotic(s) to start for the above case?
  - **Amoxicillin**
  - Azithromycin
  - Ceftriaxone
  - Oseltamivir
  - Trimethoprim-sulfamethoxazole
11. An 8-month-old infant born at term is brought in for cough, rhinorrhea, and congestion for three days. Temperature at home was 101 F. Her past medical history and birth history are unremarkable. Vital signs are T 100.1 °F, HR 129, RR 42, BP 90/60, and SpO2 98% on room air. The patient is smiling and active and tachypneic. There is scant wheezing and mild retractions.
  What is true regarding this patient’s condition?
  - Albuterol therapy has been shown to reduce 30-day mortality
  - Corticosteroids reduce the hospitalization rates
  - **Respiratory syncytial virus (RSV) is the most common cause**
  - Ribavirin is indicated
  - The patient has a 75% chance of developing asthma as a child
12. Which of the following is true regarding selection of agents for rapid sequence intubation (RSI)?
  - Ketamine is known to be safely used in a patient with a ruptured globe.
  - Neuromuscular blocking agents are contraindicated in patients who are having seizures.
  - **Rocuronium at a dose of 1–1.2 mg/kg achieves similar intubating conditions as succinylcholine 1.5–2mg/kg for emergent RSI in children**.
  - Succinylcholine would be recommended for use in a child with glomerulonephritis and the following labs (mEq/L): Na 138, K 5.8, Cl 101, HCO3 17.
13. A 5-year-old girl presents for fever for the past 2 days and now is lethargic and has BP 70/50 and HR 150 at triage. Which may improve perfusion in a patient receiving sedation during RSI for septic shock?
  - Etomidate
  - **Ketamine**
  - Midazolam
  - Thiopental
14. A 3-month-old male (born at 38 weeks gestational age) comes to the emergency room with a history of upper respiratory symptoms for 3 days, fever, decreased appetite and increased work of breathing. On physical exam his vitals are T 99 °F, RR 55, HR 185, BP 90/65, and Sat 96% on room air; he has nasal congestion, mild retractions, and coarse breath sounds. He has an older sister in primary school.
  Of the following, the MOST appropriate next step in management is:
  - Give oral corticosteroid
  - Prescription for nasal decongestants
  - Start albuterol
  - **Suction**
  - Supplementary oxygen
15. A mother brings her 6-month-old male infant to your office. She reports that her son has been breathing faster than usual for the past 2 days, and she has noted occasional wheezing. She states that prior to the difficulty breathing, she noticed some clear nasal discharge for several days. The infant was born full-term, with no complications, and no significant medical history. Vital signs are T 38 °C, BP 100/60 mmHg, HR 120 bpm, RR 40 rpm, SpO2 95%. Physical exam reveals expiratory wheezing and crackles, and intercostal retractions are noted. Which of the following is the most appropriate next step in management?
  - CT scan of the chest
  - Inhaled fluticasone
  - Intubation
  - **Non-invasive supportive care**
  - Oral amoxicillin
16. All the following patients are at high risk for morbidity and mortality from RSV infection EXCEPT:
  - A 1-month old child with an unrepaired congenital heart disease
  - A 2-month old with cerebral palsy
  - A 4-month old who was born at 32 weeks gestational age with bronchopulmonary dysplasia (BPD)
  - **A full-term infant with hyperbilirubinemia**
  - A premature infant
17. Which of the following medications is the most effective in the immediate treatment of an acute asthma exacerbation?
  - Inhaled corticosteroids
  - IV ketamine
  - IV magnesium
  - **Nebulized albuterol or salbutamol**
  - Nebulized saline
18. A previously healthy 4-year-old girl is brought into the A&E for concern for fever. After taking a complete history and performing a physical exam, you have diagnosed her with community acquired pneumonia. Which of the following does NOT necessitate inpatient admission?
  - Decreased oral intake
  - **Incomplete immunization profile**
  - Lethargy
  - Oxygen requirement
  - Persistent tachypnea
19. A 12-year-old boy presents with an asthma exacerbation. Over several hours, he is given oral steroids and several nebulized albuterol and ipratropium bromide treatments. Vital signs are P 110, RR 20, O2 saturation 95%, PEFR (peak expiratory flow rate) 250L/min (40% of his normal). He can speak in full sentences but is still wheezing. What should be the next step in his management?
  - **Continue albuterol and consider admission**
  - Discharge on albuterol and prescription for corticosteroids
  - Give subcutaneous epinephrine and reassess
  - Give subcutaneous terbutaline and admit to the ICU
  - Initiate noninvasive positive pressure
20. Which of the following is most true regarding pediatric community-acquired pneumonia (CAP)?
  - It is easier to differentiate between typical and atypical pneumonia in pediatric patients
  - **Streptococcus pneumoniae is the most commonly isolated organism in children aged 5 years to 15 years**
  - The incidence of CAP in children younger than 5 years old is higher than in middle-aged adult smokers
  - The most common cause of pneumonia in the neonate is Mycoplasma pneumoniae

## Appendix Q Debriefing Techniques

**Table t35-jetem-6-2-c73:**

Directive Feedback	Learner self-assessment (plus/delta)	Advocacy-Inquiry
Immediate instructor-driven feedback on performance gaps and learning objectives	Exploration of learner-centric performance gaps and learning objectives	Learner focused facilitationrelated to instructor-driven performance gaps and learning objectives
*“I noticed you did (actual action)*. *Next time you may want to try doing (desired action) because (rationale).”* *“I liked how you did (praise point)*. *Next time continue to do that because (rationale).”*	*“What aspects of (performance) do you think you’d like to do differently?”* *What aspects of (performance) do you think you managed well?”*	*“I noticed (actual action). I was concerned because (rationale)*. *How do you see it?”* *I saw how you (desired action). I liked that because (appreciation)*. *What was your take on it?”*

[Table t35-jetem-6-2-c73]

## Appendix R Example Itinerary

Please see associated PowerPoint file
