# Supplementary material for: A Pediatric Emergency Medicine Refresher Course for Generalist Healthcare Providers in Belize: Respiratory Emergencies
Source: J Educ Teach Emerg Med. 2021 Apr 19;6(2):C73–C188. doi: 10.21980/J84063 (PMC10332788; doi:10.21980/J84063)
Supplement: Supplementary file 1 — Please see associated PowerPoint file [file jetem-6-2-c73-AppendixC.pptx]

## Slide 1
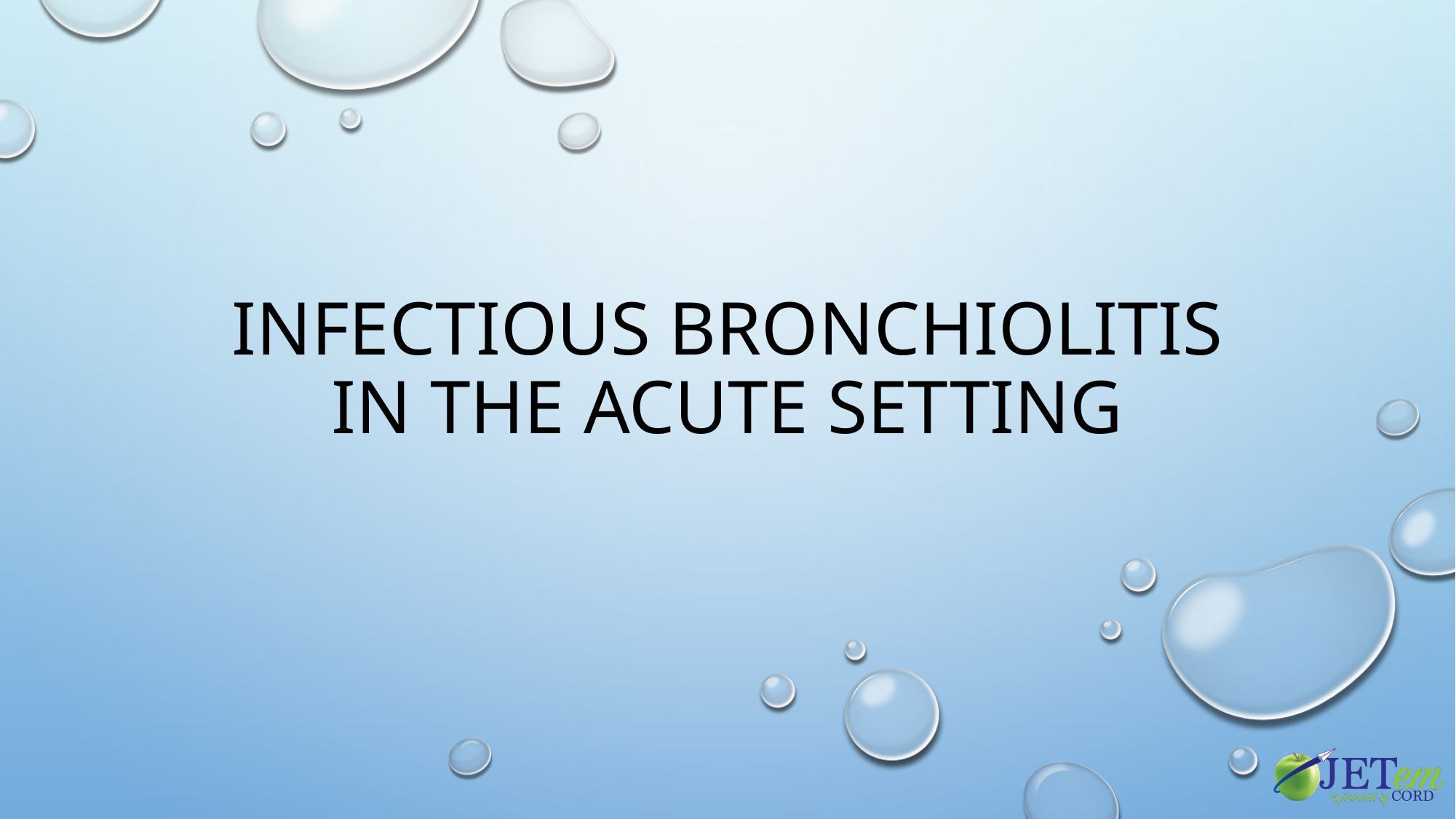

# infectious Bronchiolitis in the acute setting

## Slide 2
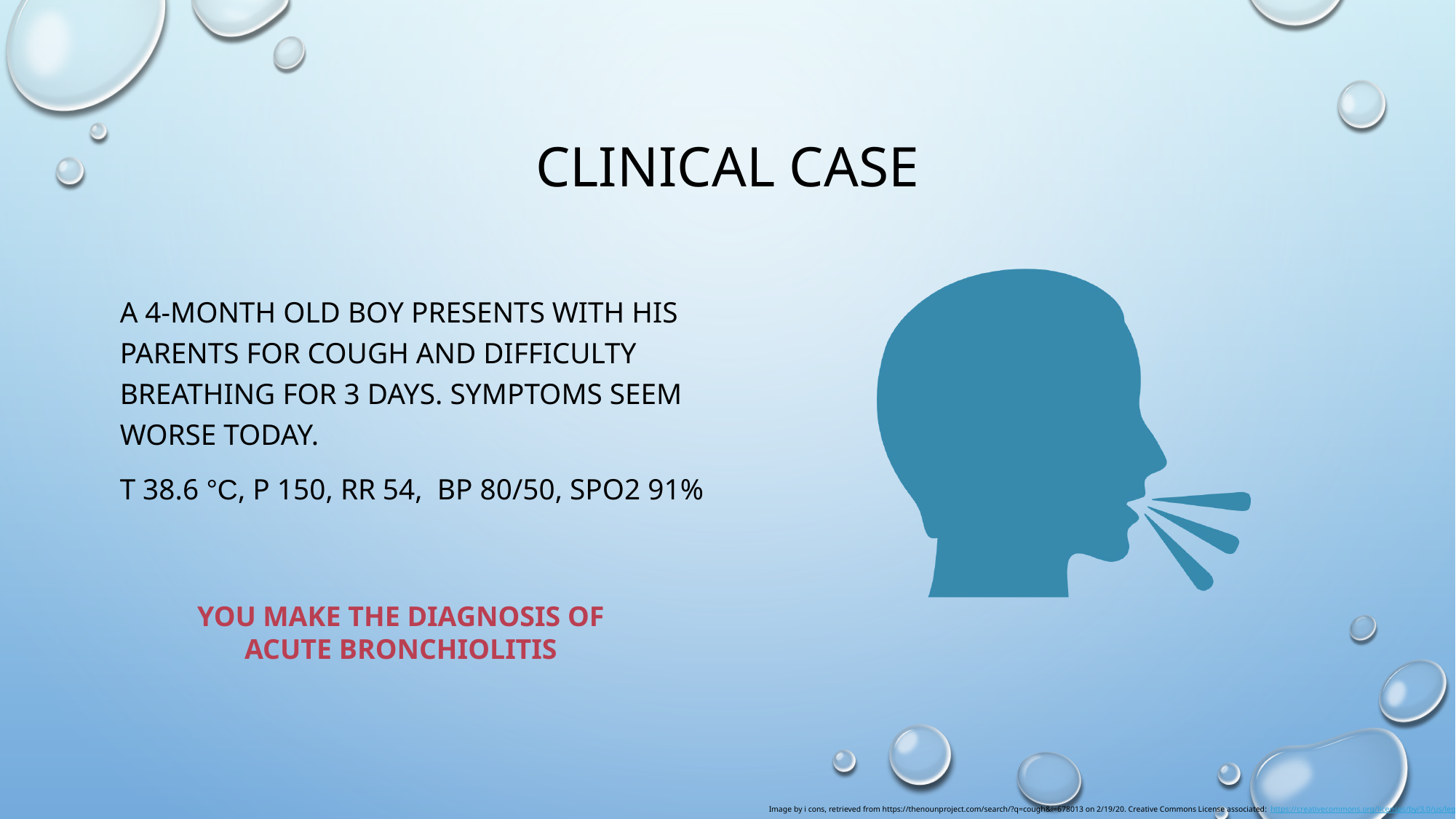

# Clinical Case
A 4-month old boy presents with his parents for cough and difficulty breathing for 3 days. Symptoms seem worse today.
T 38.6 °C, P 150, RR 54, BP 80/50, SPO2 91%
YOU MAKE THE DIAGNOSIS OF ACUTE BRONCHIOLITIS
Image by i cons, retrieved from https://thenounproject.com/search/?q=cough&i=678013 on 2/19/20. Creative Commons License associated: https://creativecommons.org/licenses/by/3.0/us/legalcode.

## Slide 3
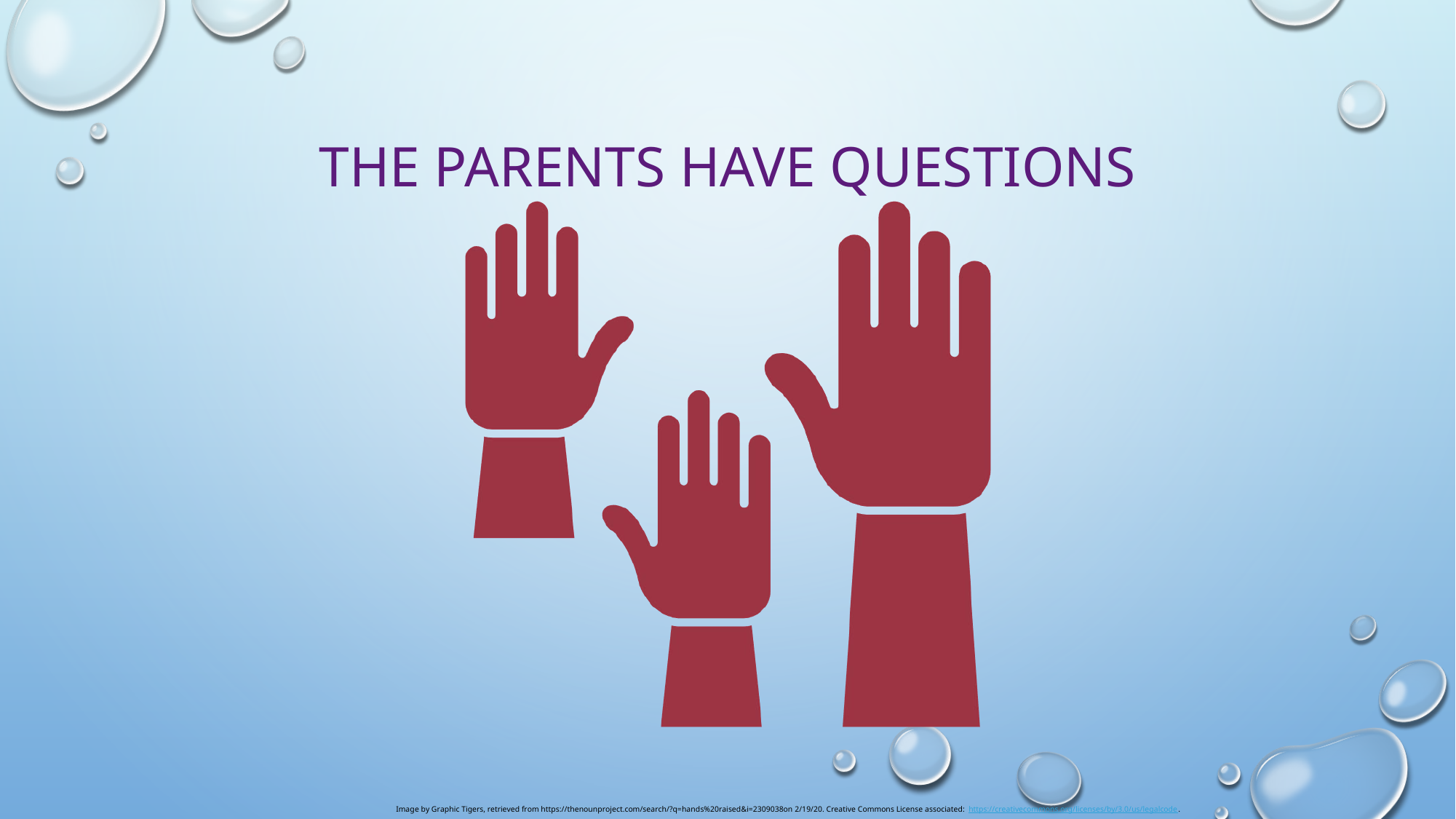

# The parents have questions
Image by Graphic Tigers, retrieved from https://thenounproject.com/search/?q=hands%20raised&i=2309038on 2/19/20. Creative Commons License associated: https://creativecommons.org/licenses/by/3.0/us/legalcode.

## Slide 4
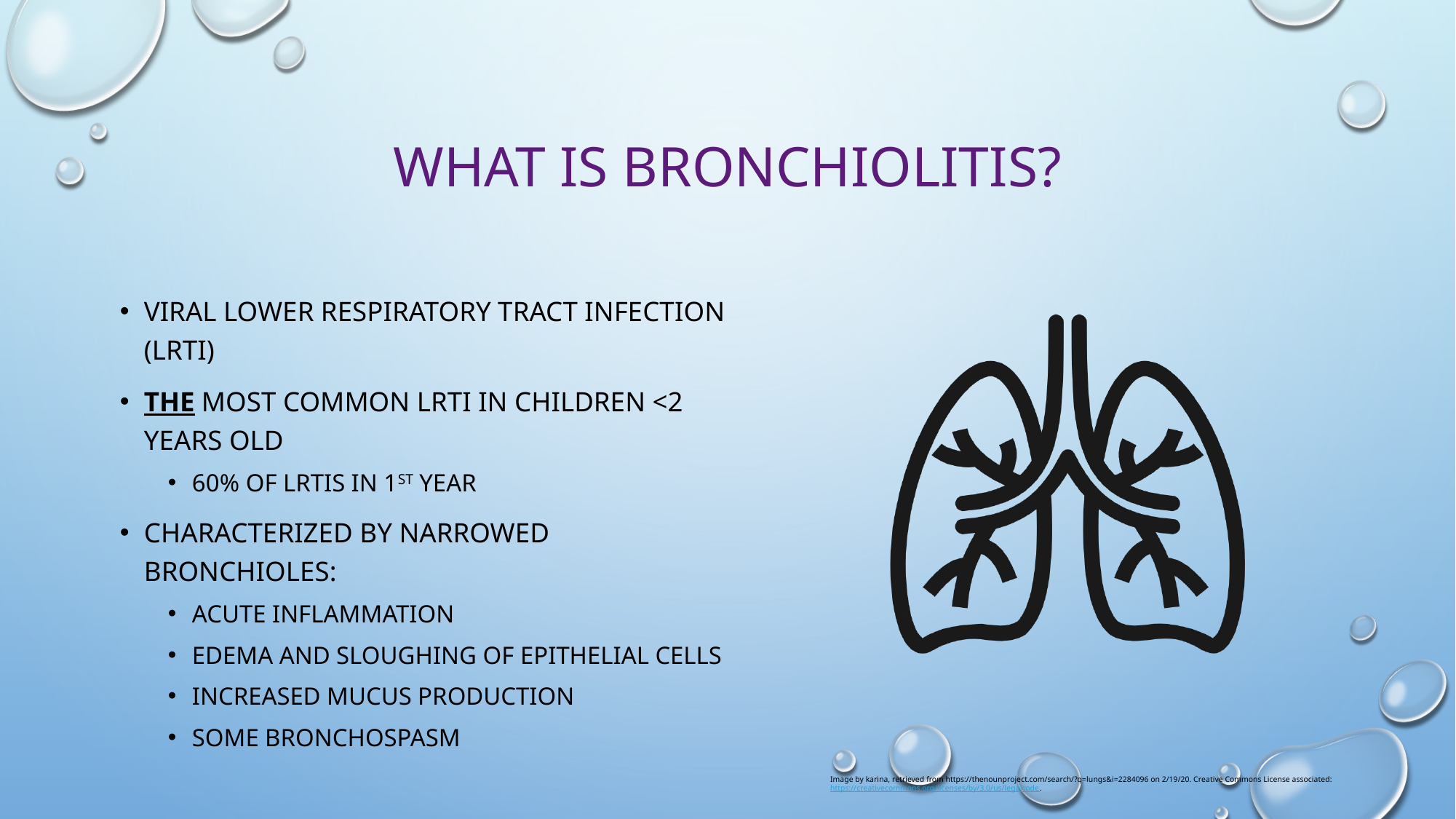

# What is Bronchiolitis?
viral lower respiratory tract infection (LRTI)
THE most common LRTI in children <2 years old
60% of LRTIs in 1st year
characterized by narrowed Bronchioles:
acute inflammation
edema and sloughing of epithelial cells
increased mucus production
some bronchospasm
Image by karina, retrieved from https://thenounproject.com/search/?q=lungs&i=2284096 on 2/19/20. Creative Commons License associated: https://creativecommons.org/licenses/by/3.0/us/legalcode.

## Slide 5
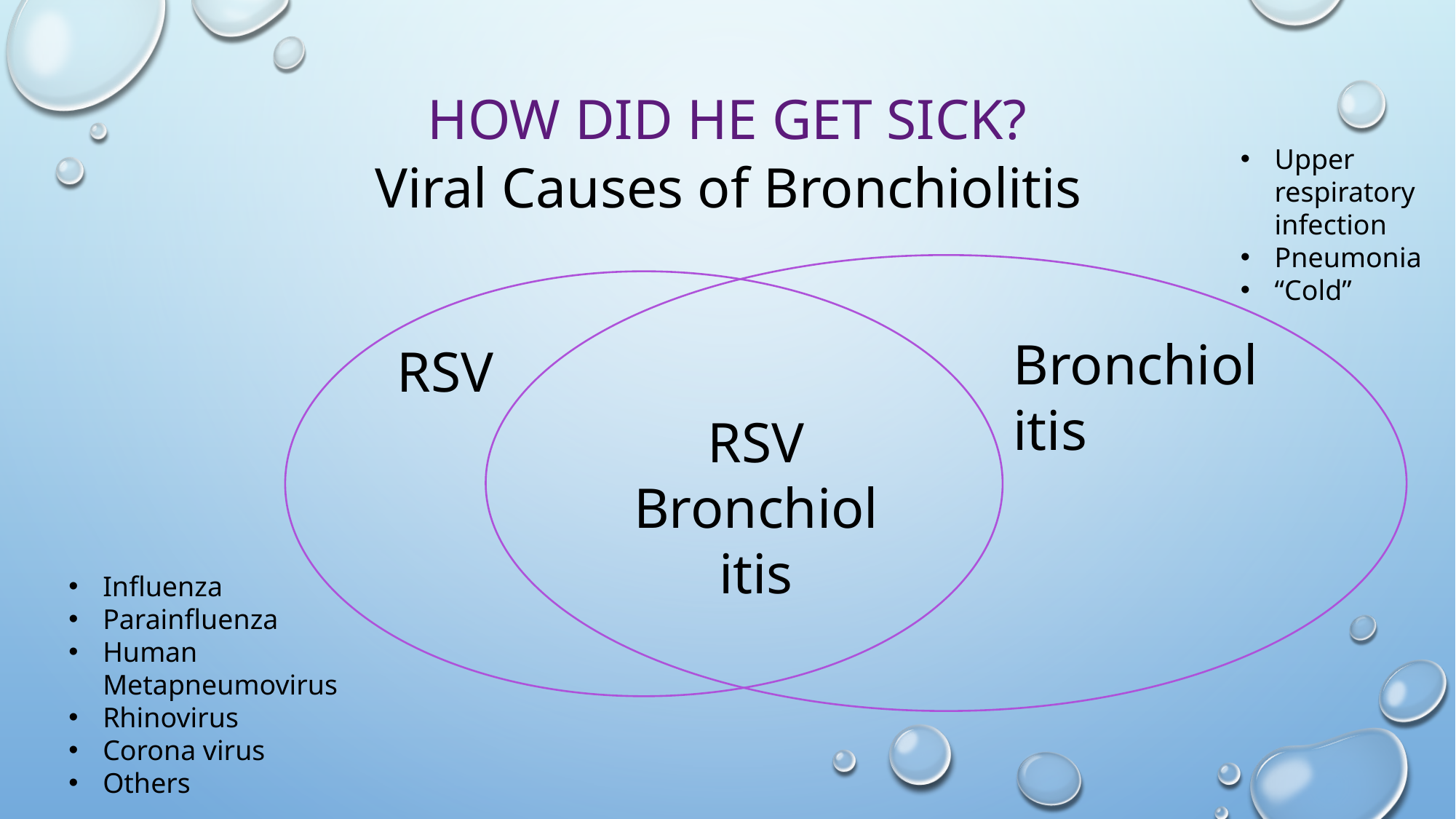

# How did He get sick?
Upper respiratory infection
Pneumonia
“Cold”
Viral Causes of Bronchiolitis
Bronchiolitis
RSV
RSV
Bronchiolitis
Influenza
Parainfluenza
Human Metapneumovirus
Rhinovirus
Corona virus
Others

## Slide 6
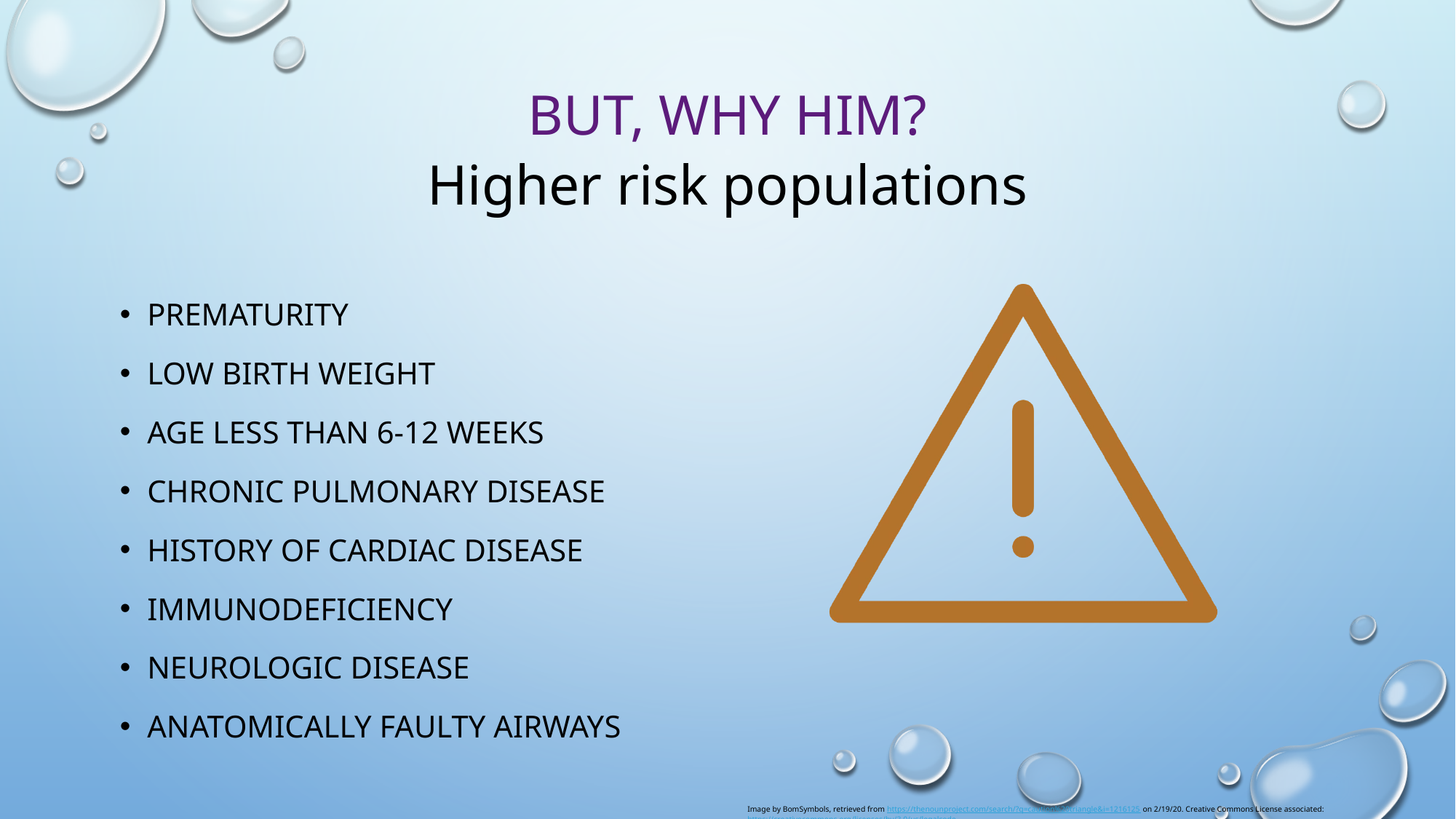

# But, why him?
Higher risk populations
prematurity
low birth weight
age less than 6-12 weeks
chronic pulmonary disease
history of cardiac disease
immunodeficiency
neurologic disease
anatomically faulty airways
Image by BomSymbols, retrieved from https://thenounproject.com/search/?q=caution%20triangle&i=1216125 on 2/19/20. Creative Commons License associated: https://creativecommons.org/licenses/by/3.0/us/legalcode.

## Slide 7
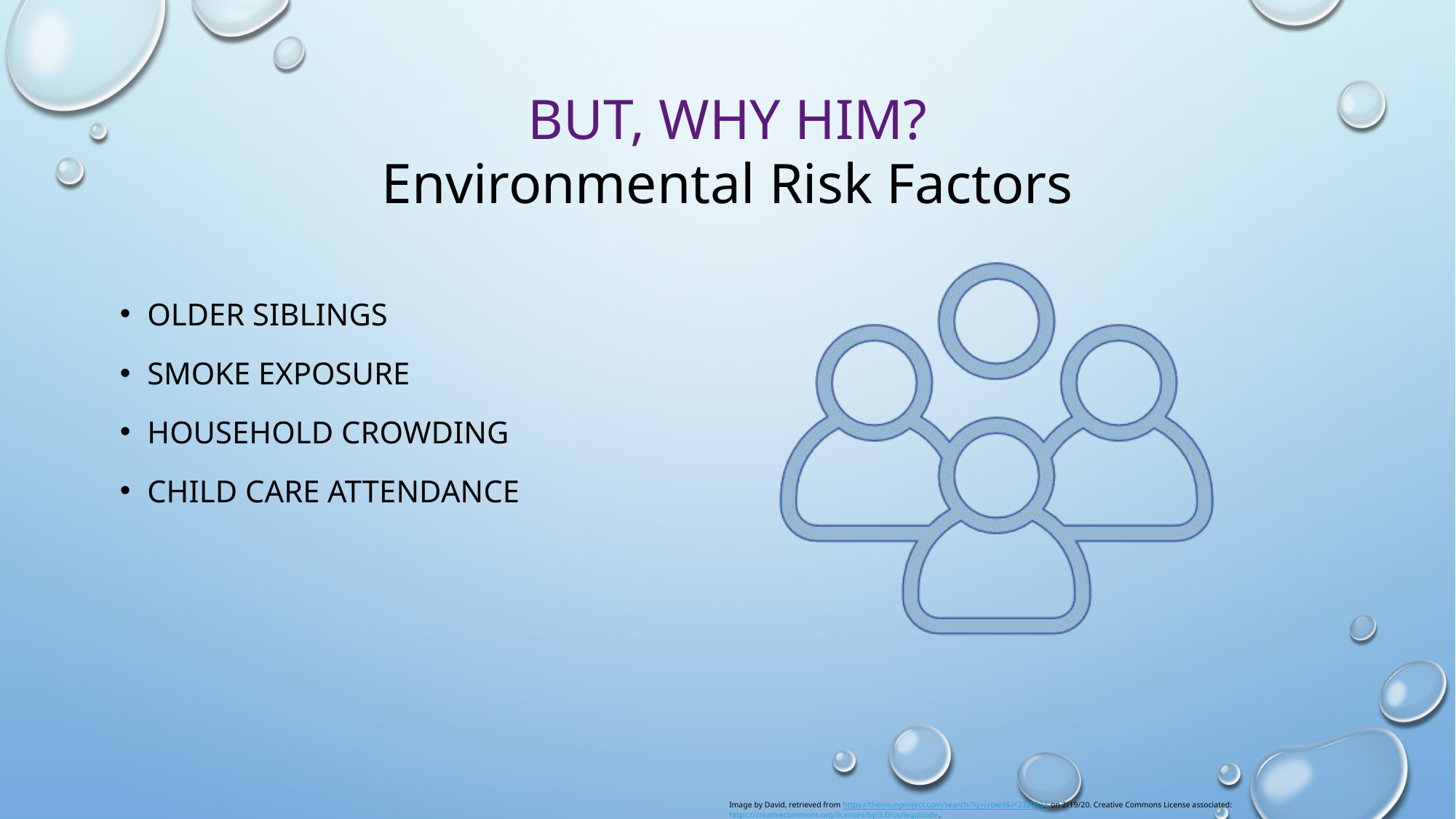

# But, why him?
Environmental Risk Factors
older siblings
smoke exposure
household crowding
child care attendance
Image by David, retrieved from https://thenounproject.com/search/?q=crowd&i=2320534 on 2/19/20. Creative Commons License associated: https://creativecommons.org/licenses/by/3.0/us/legalcode.

## Slide 8
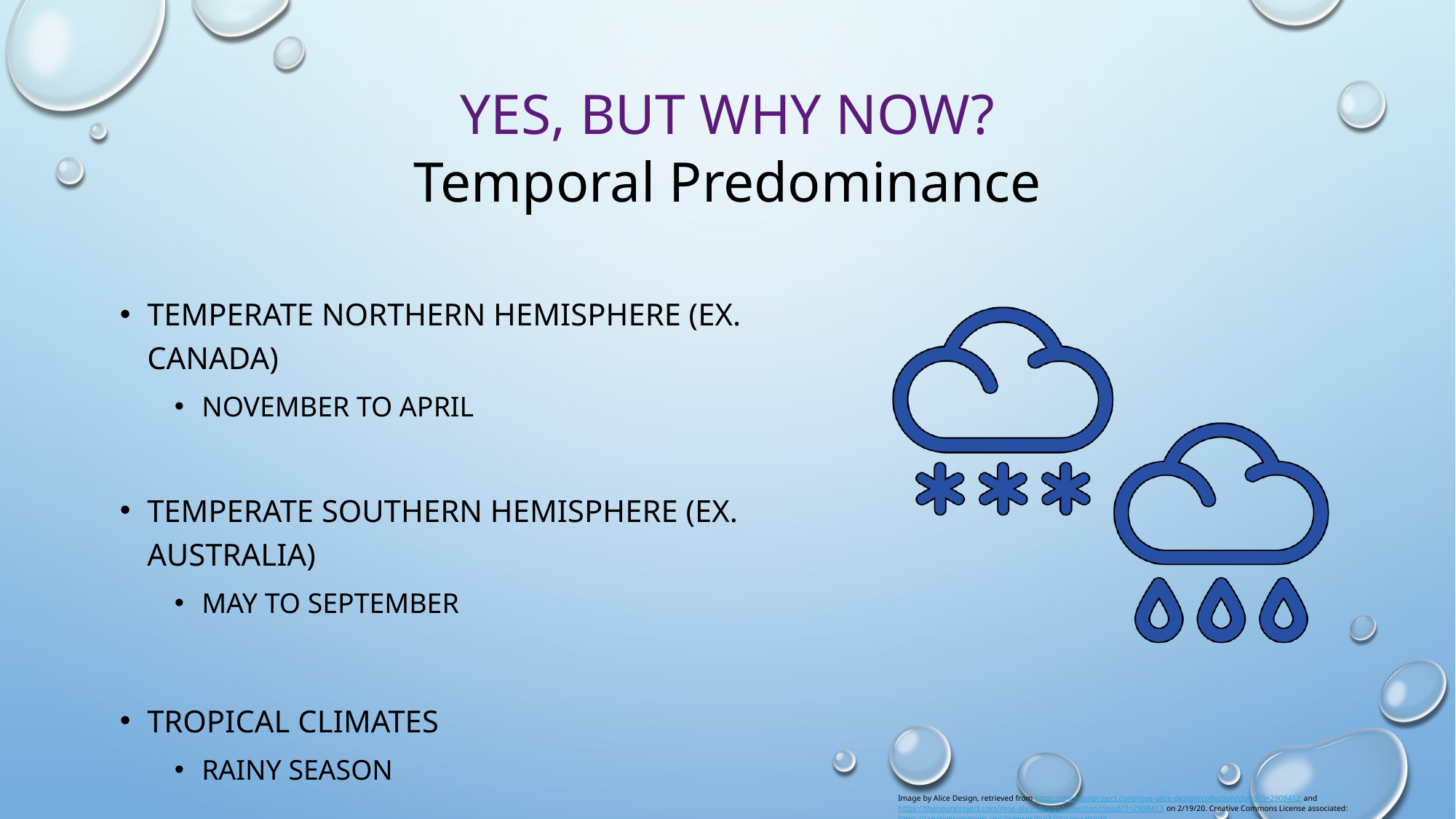

# Yes, but why now?
Temporal Predominance
Temperate Northern Hemisphere (ex. Canada)
November to April
Temperate Southern Hemisphere (ex. Australia)
May to September
Tropical Climates
Rainy Season
Image by Alice Design, retrieved from https://thenounproject.com/rose-alice-design/collection/cloud/?i=2908412 and https://thenounproject.com/rose-alice-design/collection/cloud/?i=2908413 on 2/19/20. Creative Commons License associated: https://creativecommons.org/licenses/by/3.0/us/legalcode.

## Slide 9
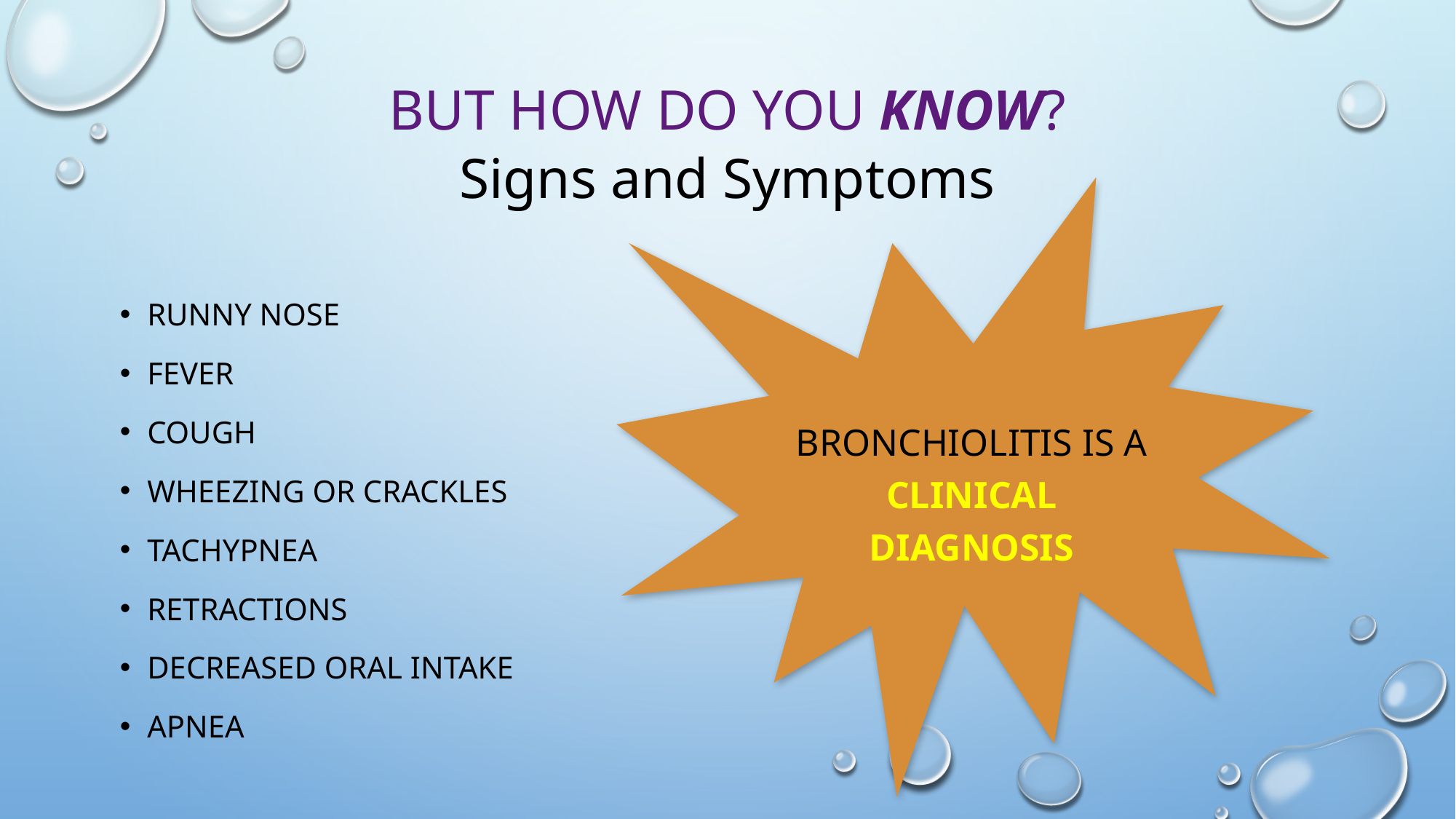

# But how do you know?
Signs and Symptoms
runny nose
fever
cough
wheezing or crackles
tachypnea
retractions
decreased oral intake
Apnea
Bronchiolitis is a clinical diagnosis

## Slide 10
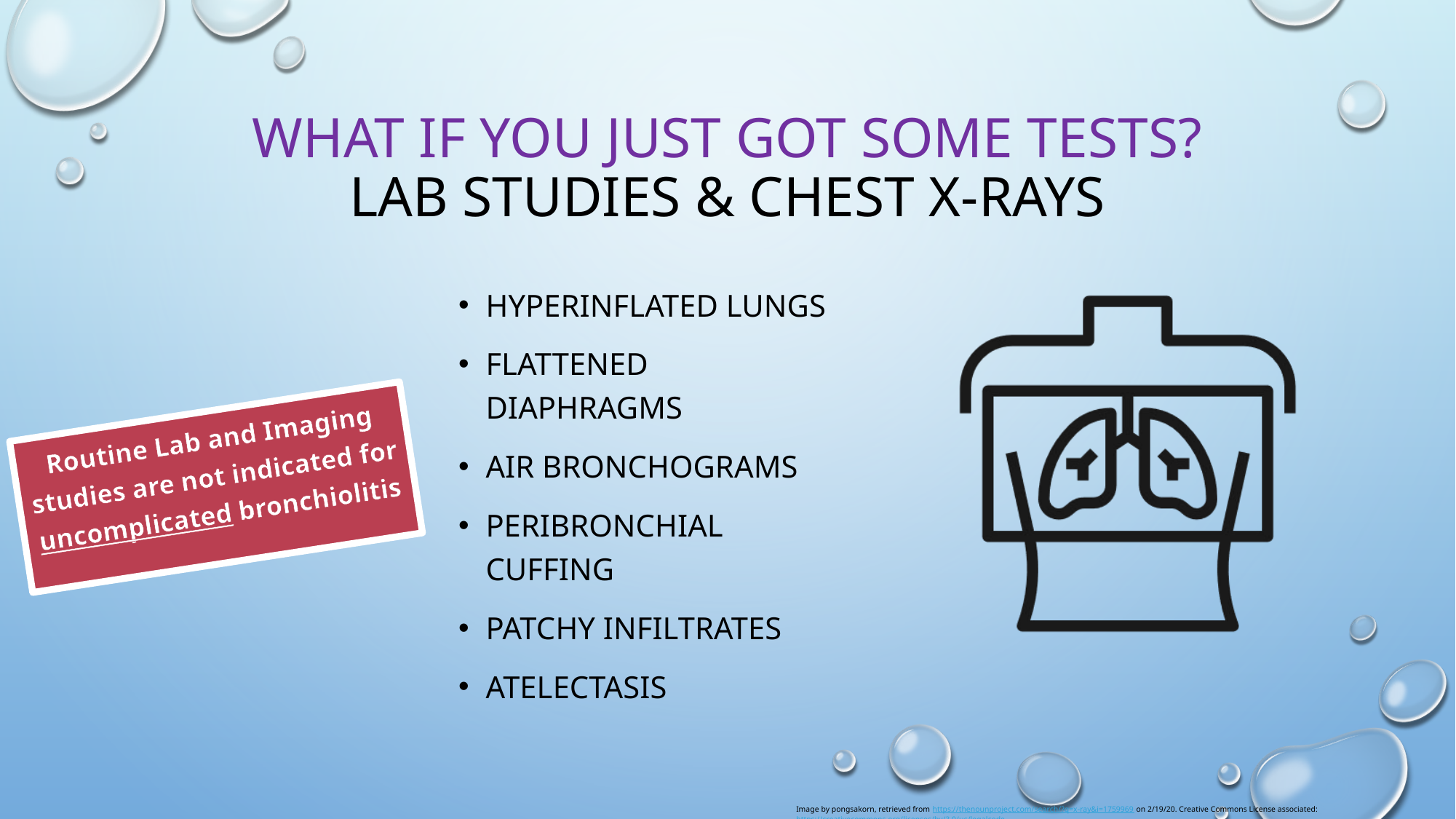

# What if you just got Some tests? Lab studies & Chest X-rays
hyperinflated lungs
flattened diaphragms
air bronchograms
peribronchial cuffing
patchy infiltrates
atelectasis
Routine Lab and Imaging studies are not indicated for uncomplicated bronchiolitis
Image by pongsakorn, retrieved from https://thenounproject.com/search/?q=x-ray&i=1759969 on 2/19/20. Creative Commons License associated: https://creativecommons.org/licenses/by/3.0/us/legalcode.

## Slide 11
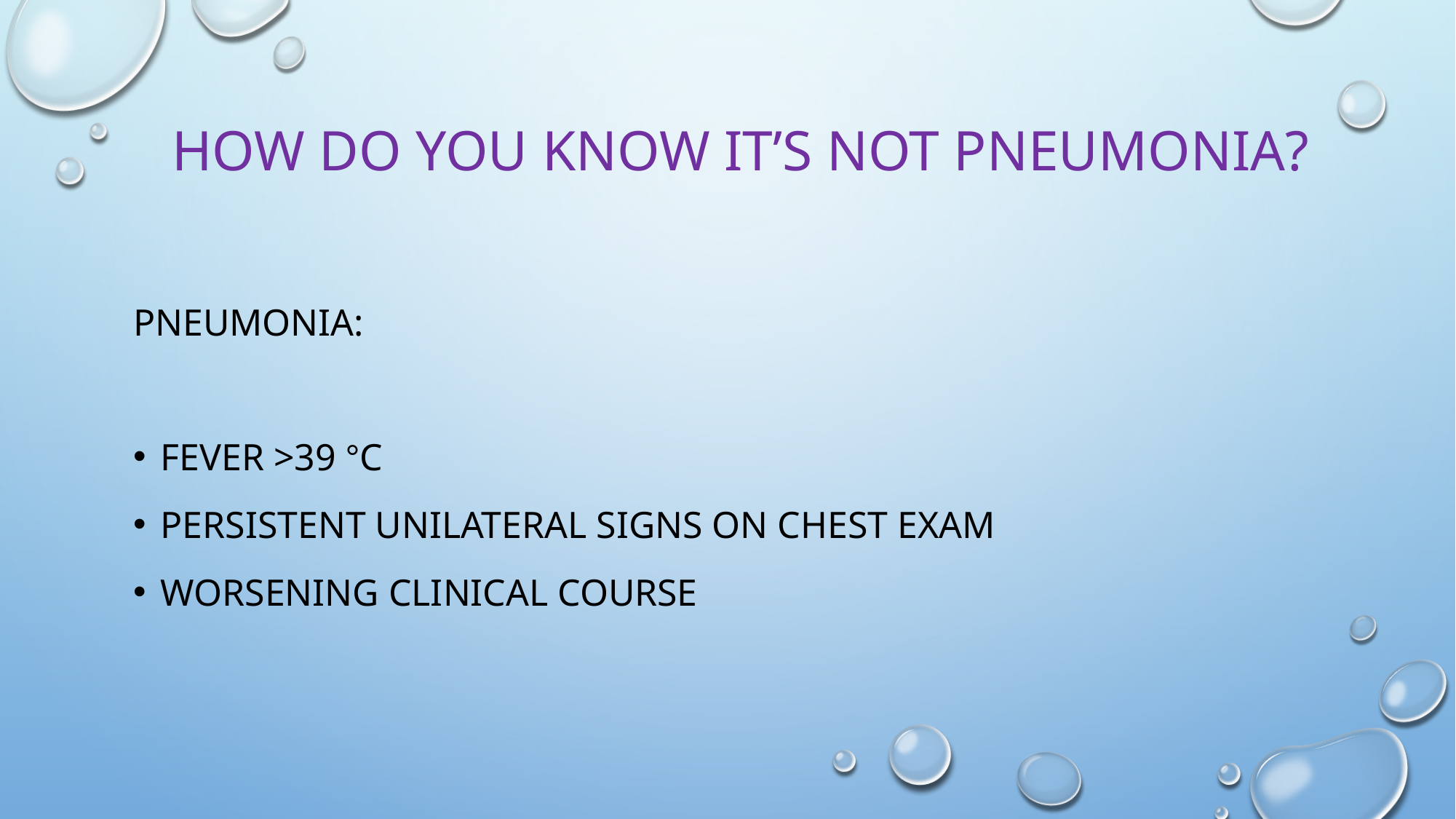

# How do you know it’s not pneumonia?
Pneumonia:
Fever >39 °C
Persistent unilateral signs on chest exam
worsening clinical course

## Slide 12
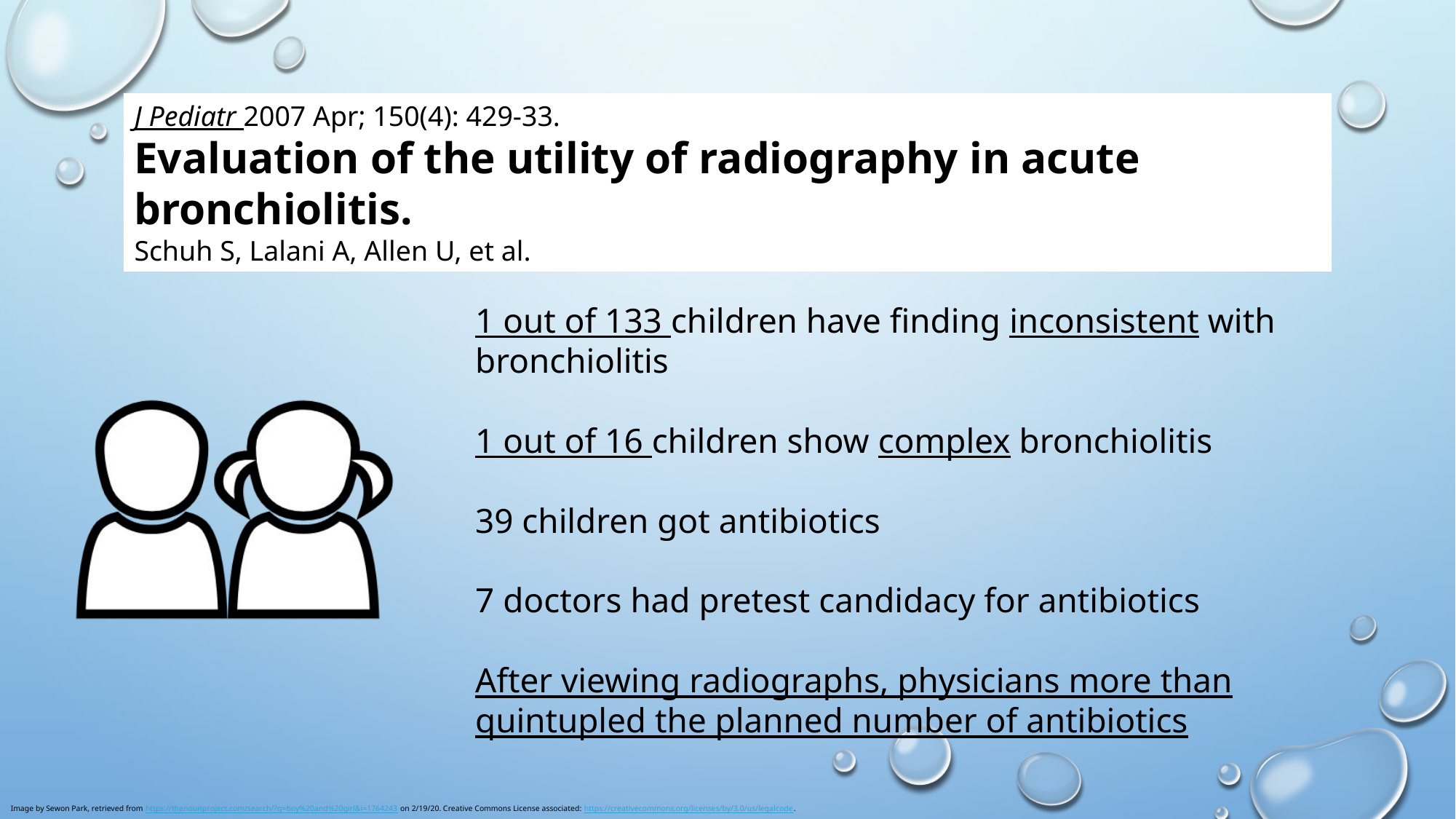

J Pediatr 2007 Apr; 150(4): 429-33.
Evaluation of the utility of radiography in acute bronchiolitis.
Schuh S, Lalani A, Allen U, et al.
1 out of 133 children have finding inconsistent with bronchiolitis
1 out of 16 children show complex bronchiolitis
39 children got antibiotics
7 doctors had pretest candidacy for antibiotics
After viewing radiographs, physicians more than quintupled the planned number of antibiotics
Image by Sewon Park, retrieved from https://thenounproject.com/search/?q=boy%20and%20girl&i=1764243 on 2/19/20. Creative Commons License associated: https://creativecommons.org/licenses/by/3.0/us/legalcode.

## Slide 13
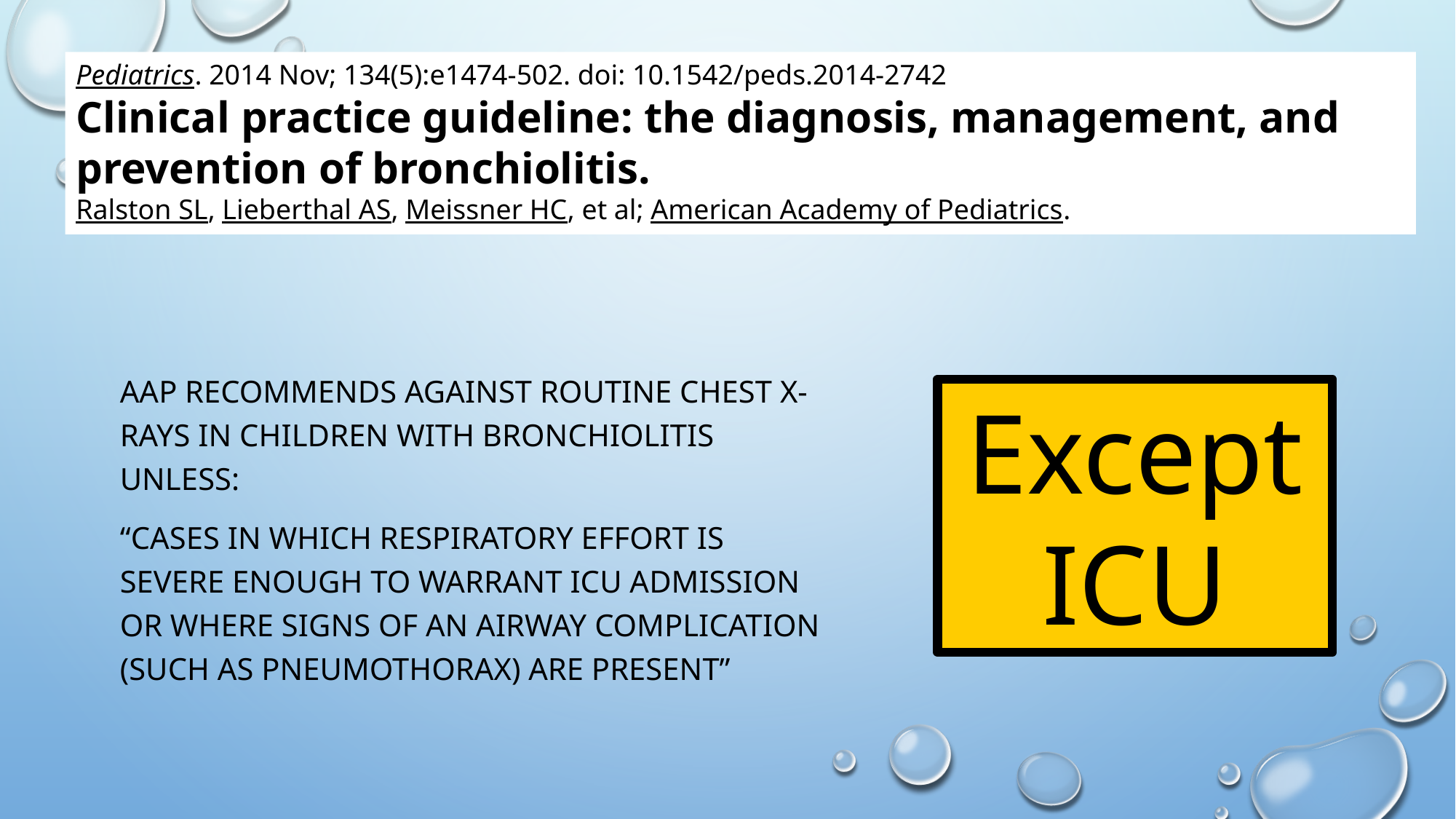

Pediatrics. 2014 Nov; 134(5):e1474-502. doi: 10.1542/peds.2014-2742
Clinical practice guideline: the diagnosis, management, and prevention of bronchiolitis.
Ralston SL, Lieberthal AS, Meissner HC, et al; American Academy of Pediatrics.
AAP recommends against routine chest x-rays in children with bronchiolitis unless:
“cases in which respiratory effort is severe enough to warrant ICU admission or where signs of an airway complication (such as pneumothorax) are present”
Except
ICU

## Slide 14
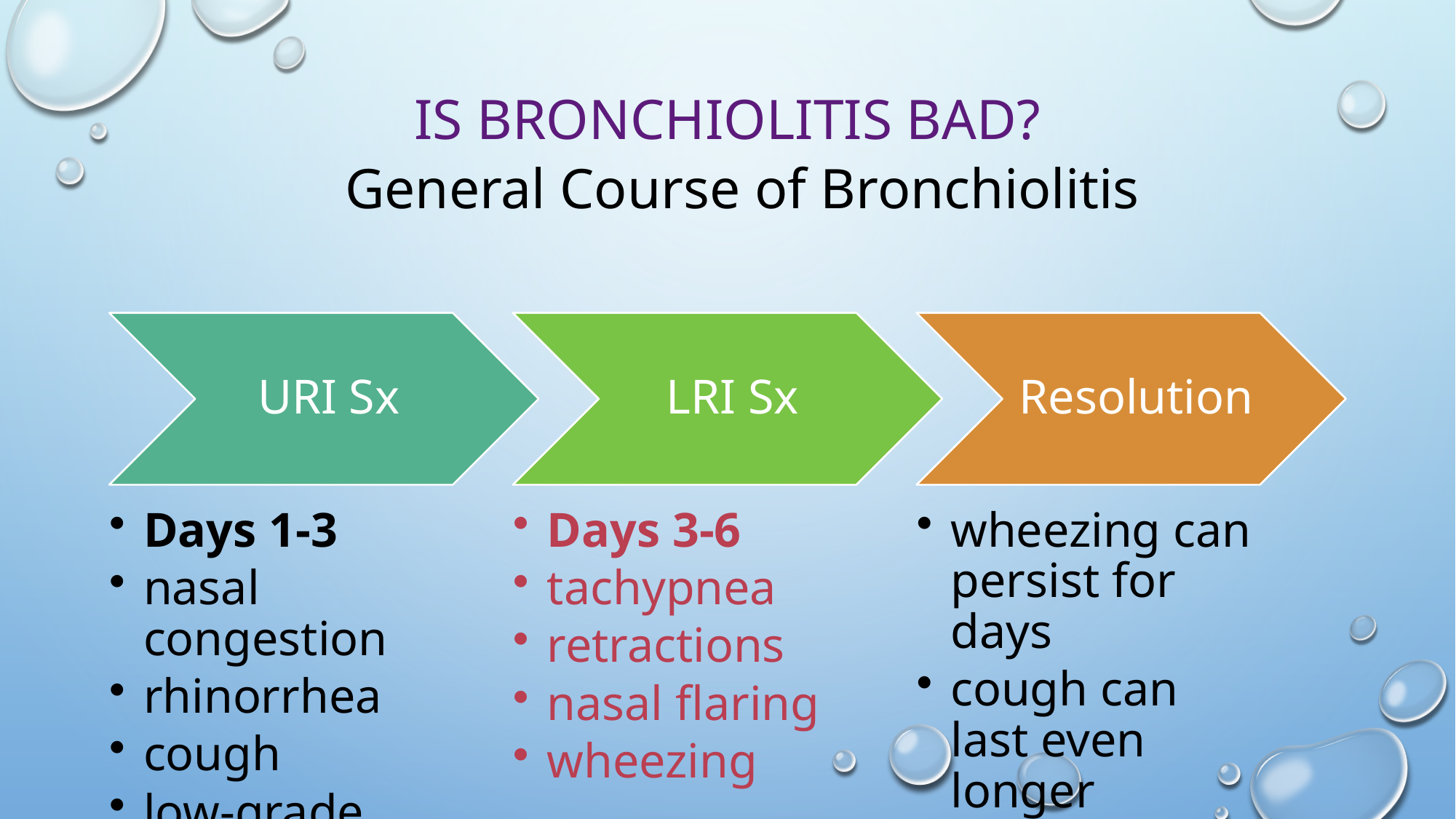

# Is Bronchiolitis Bad?
General Course of Bronchiolitis

## Slide 15
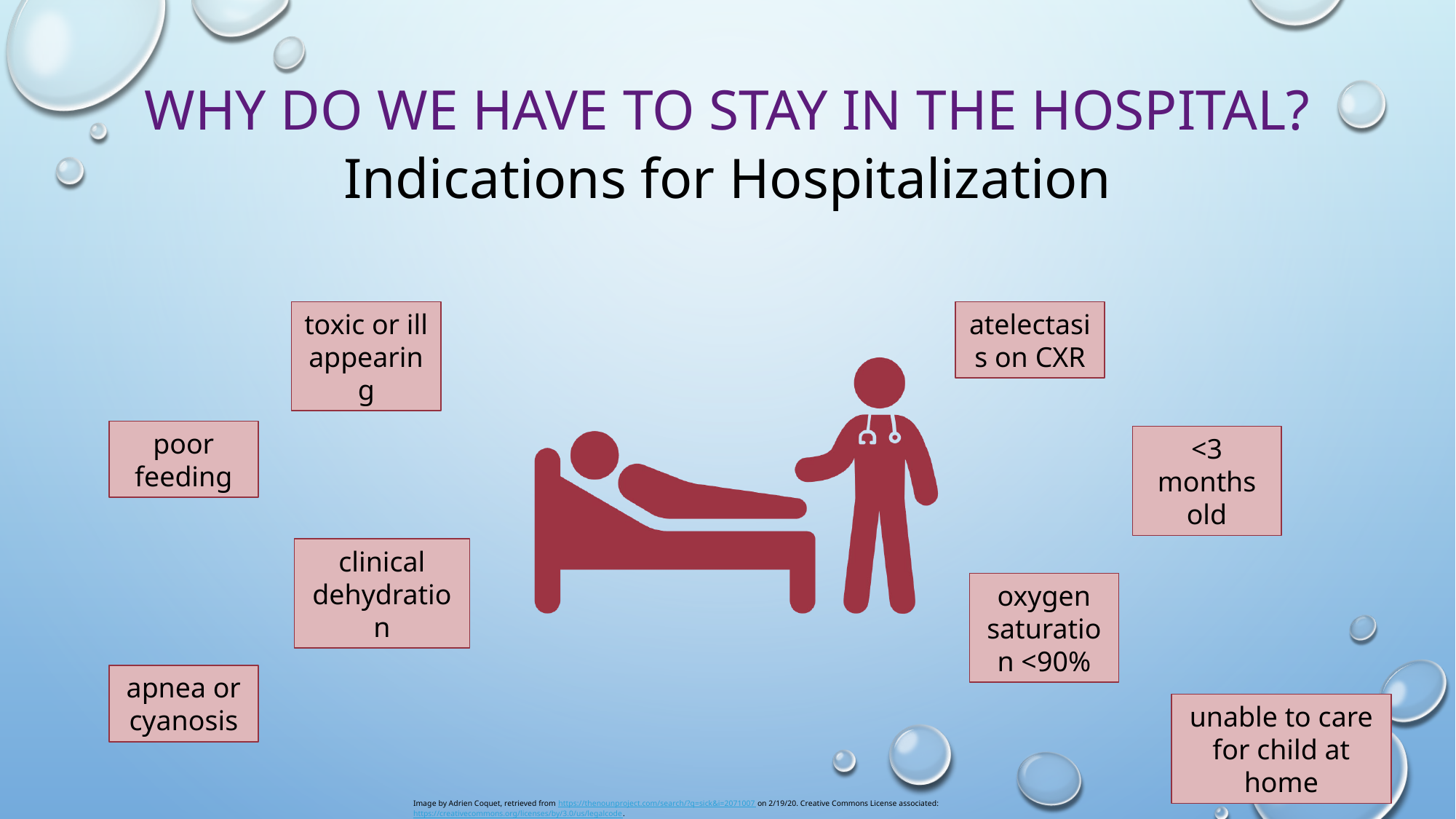

# Why do we Have to Stay in the Hospital?
Indications for Hospitalization
toxic or ill appearing
atelectasis on CXR
poor feeding
<3 months old
clinical dehydration
oxygen saturation <90%
apnea or cyanosis
unable to care for child at home
Image by Adrien Coquet, retrieved from https://thenounproject.com/search/?q=sick&i=2071007 on 2/19/20. Creative Commons License associated: https://creativecommons.org/licenses/by/3.0/us/legalcode.

## Slide 16
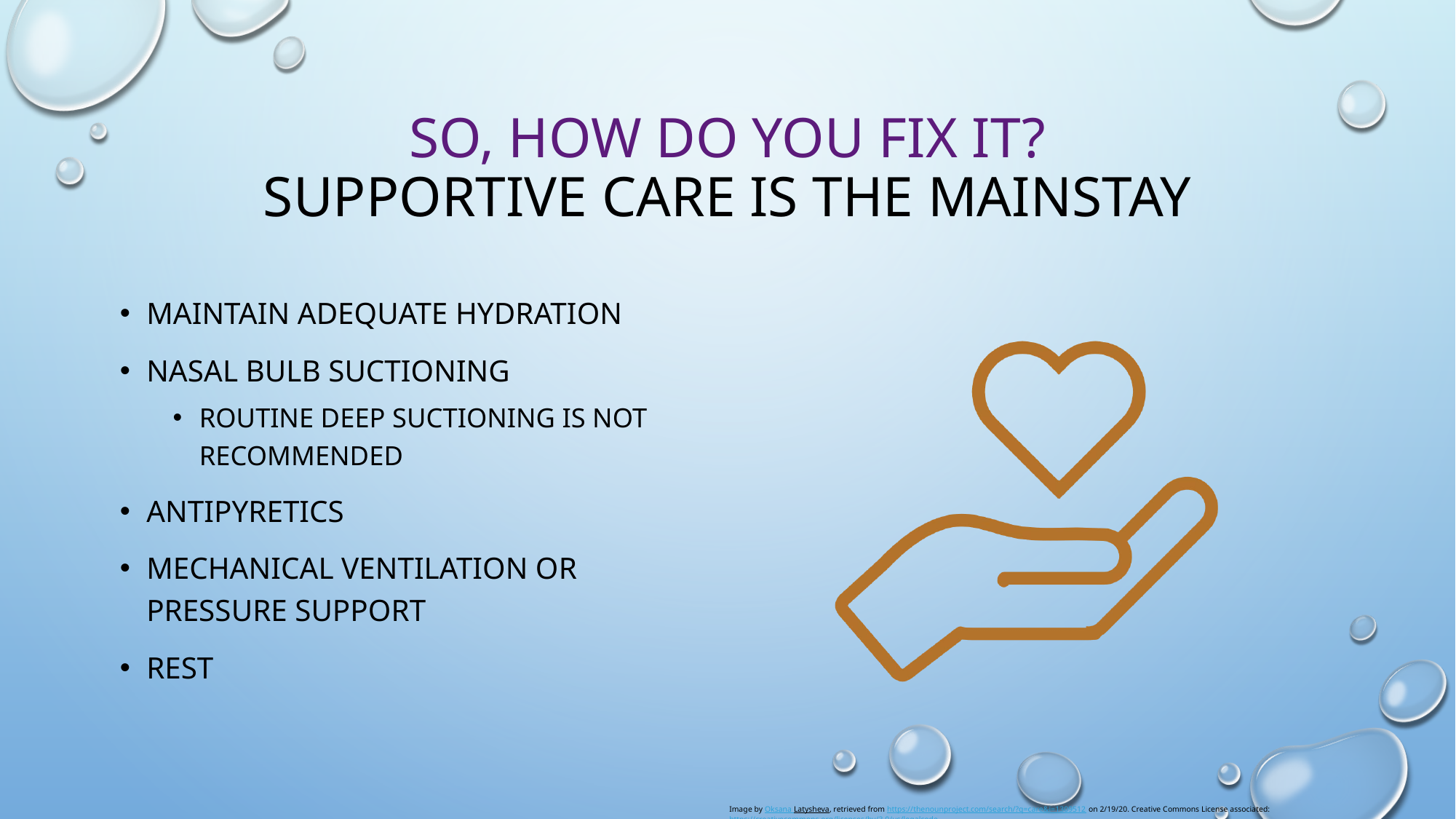

# So, how do you fix it? Supportive Care is the Mainstay
maintain adequate Hydration
nasal bulb suctioning
routine deep suctioning is not recommended
Antipyretics
Mechanical ventilation or pressure support
rest
Image by Oksana Latysheva, retrieved from https://thenounproject.com/search/?q=care&i=1309512 on 2/19/20. Creative Commons License associated: https://creativecommons.org/licenses/by/3.0/us/legalcode.

## Slide 17
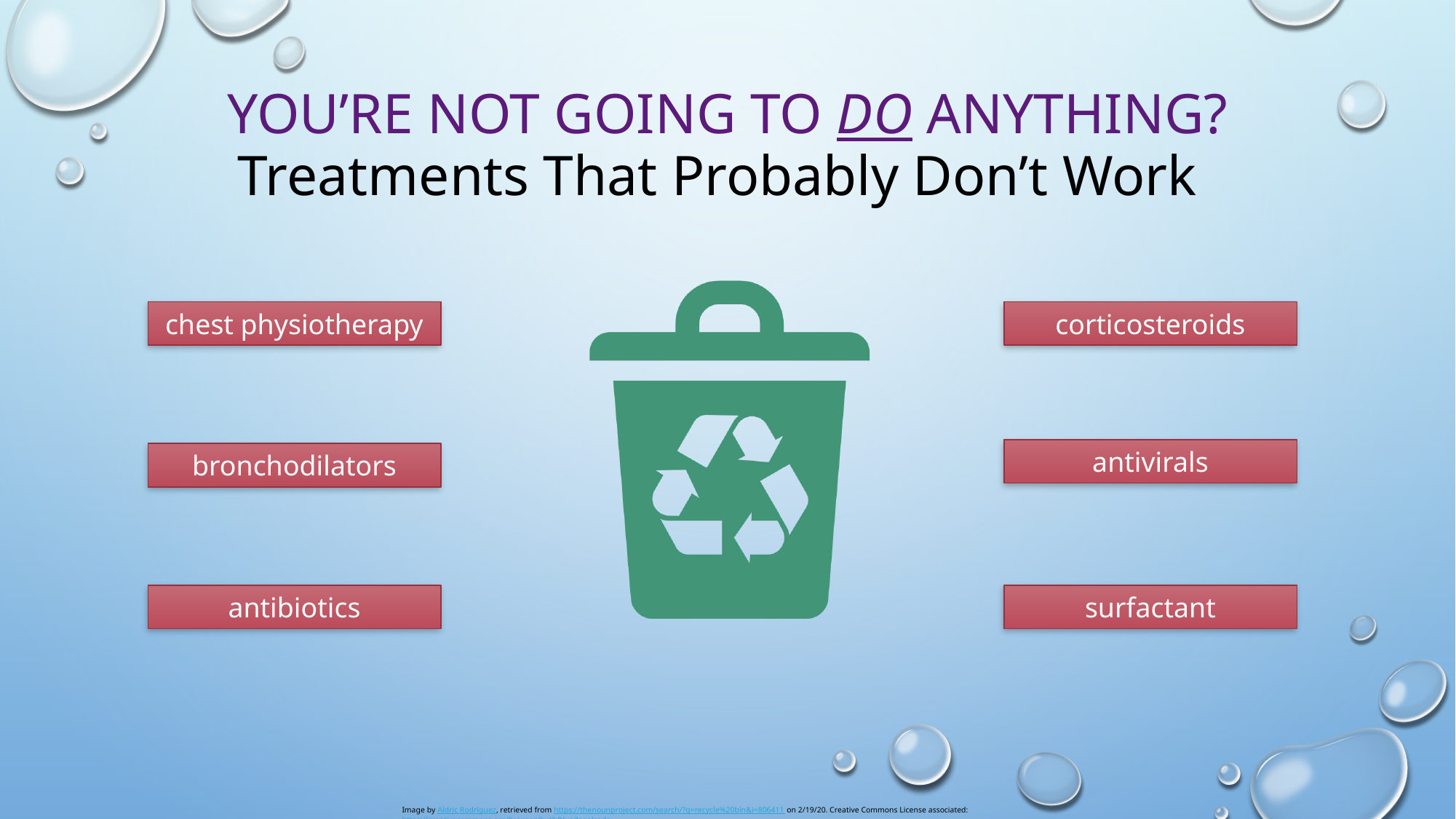

# You’re not going to Do Anything?
Treatments That Probably Don’t Work
chest physiotherapy
corticosteroids
antivirals
bronchodilators
antibiotics
surfactant
Image by Aldric Rodríguez, retrieved from https://thenounproject.com/search/?q=recycle%20bin&i=806411 on 2/19/20. Creative Commons License associated: https://creativecommons.org/licenses/by/3.0/us/legalcode.

## Slide 18
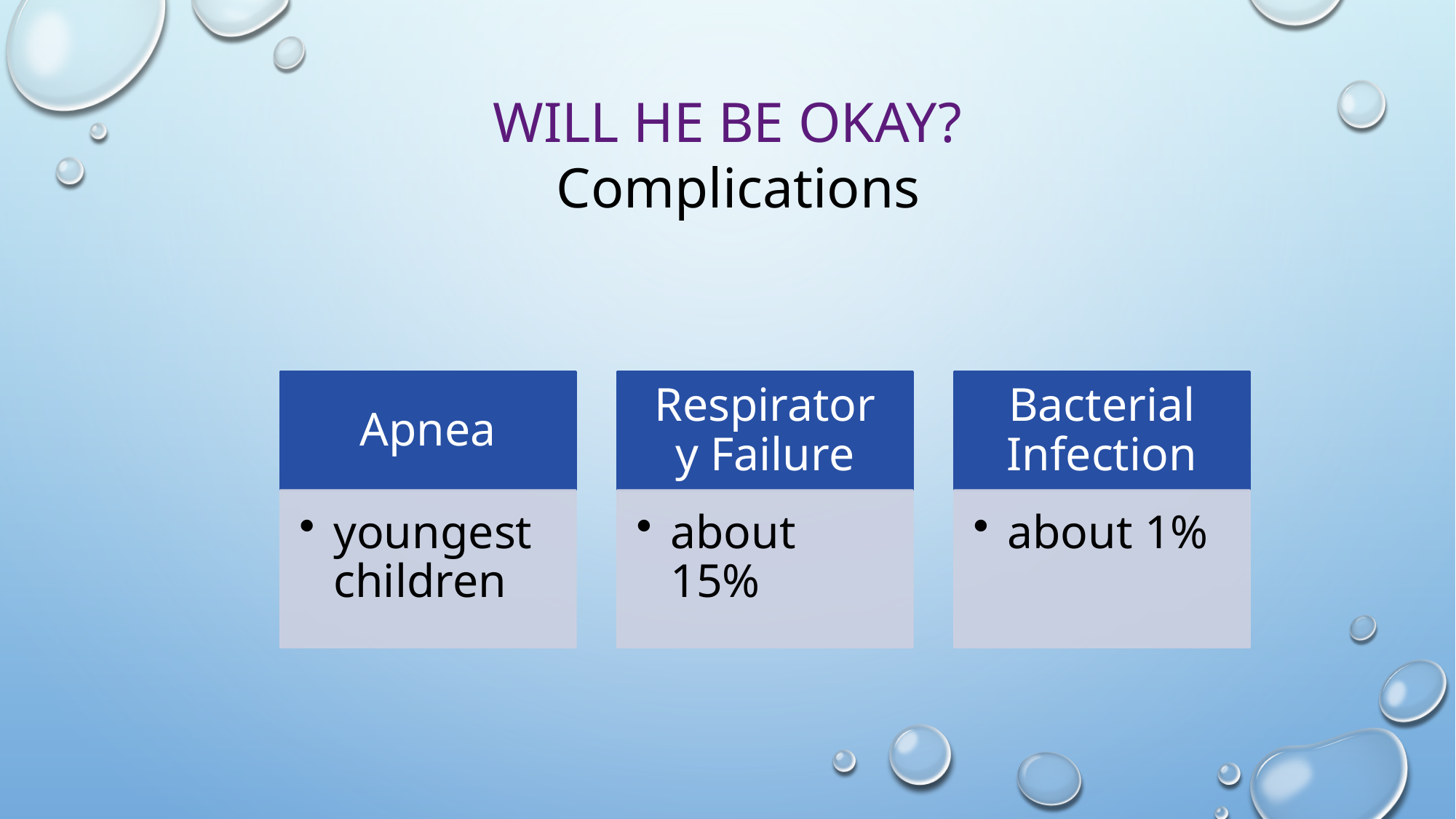

# Will He be okay?
Complications

## Slide 19
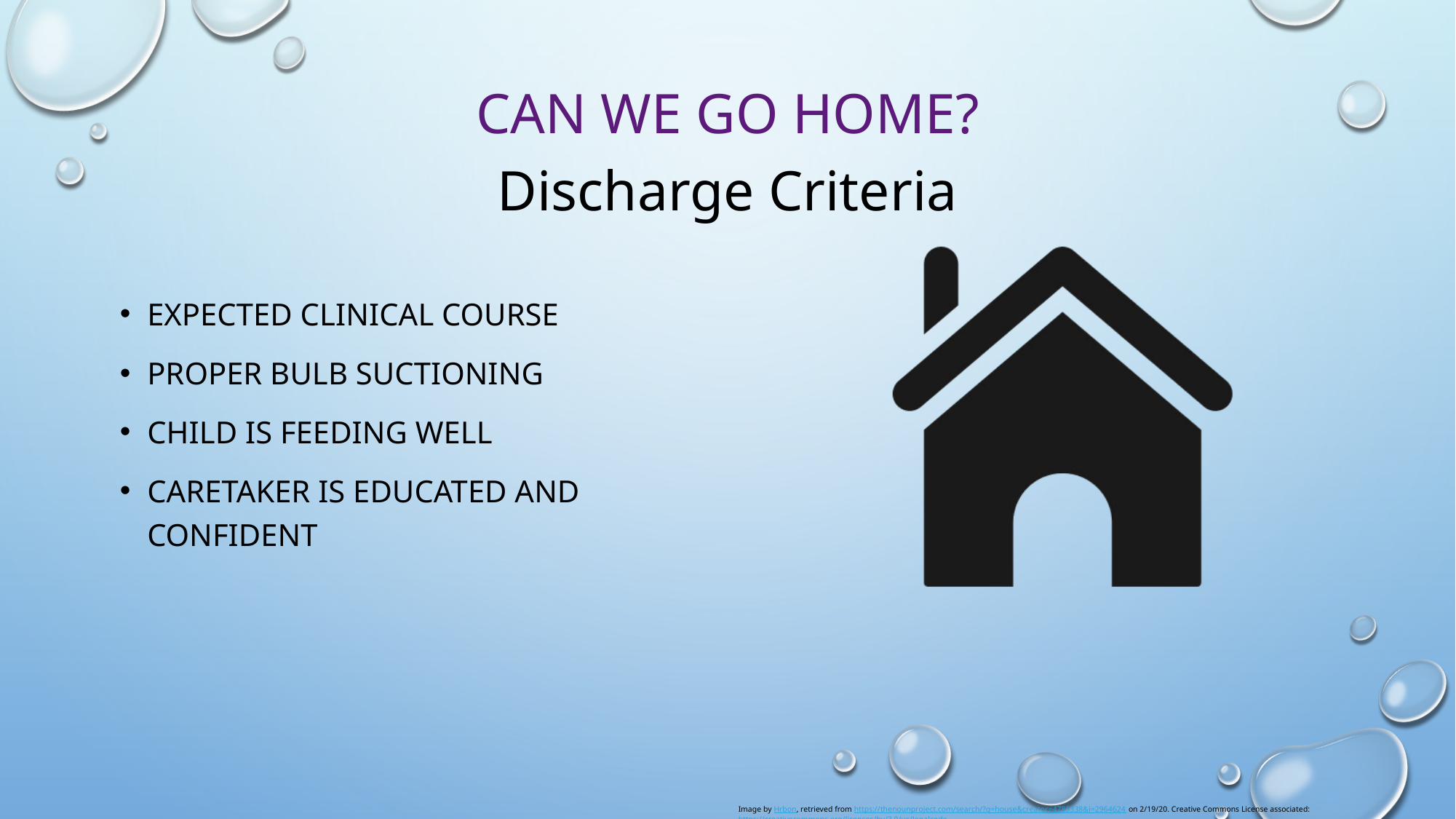

# Can we go home?
Discharge Criteria
Expected clinical course
proper bulb suctioning
child is feeding well
Caretaker is educated and confident
Image by Hrbon, retrieved from https://thenounproject.com/search/?q=house&creator=4785338&i=2964624 on 2/19/20. Creative Commons License associated: https://creativecommons.org/licenses/by/3.0/us/legalcode.

## Slide 20
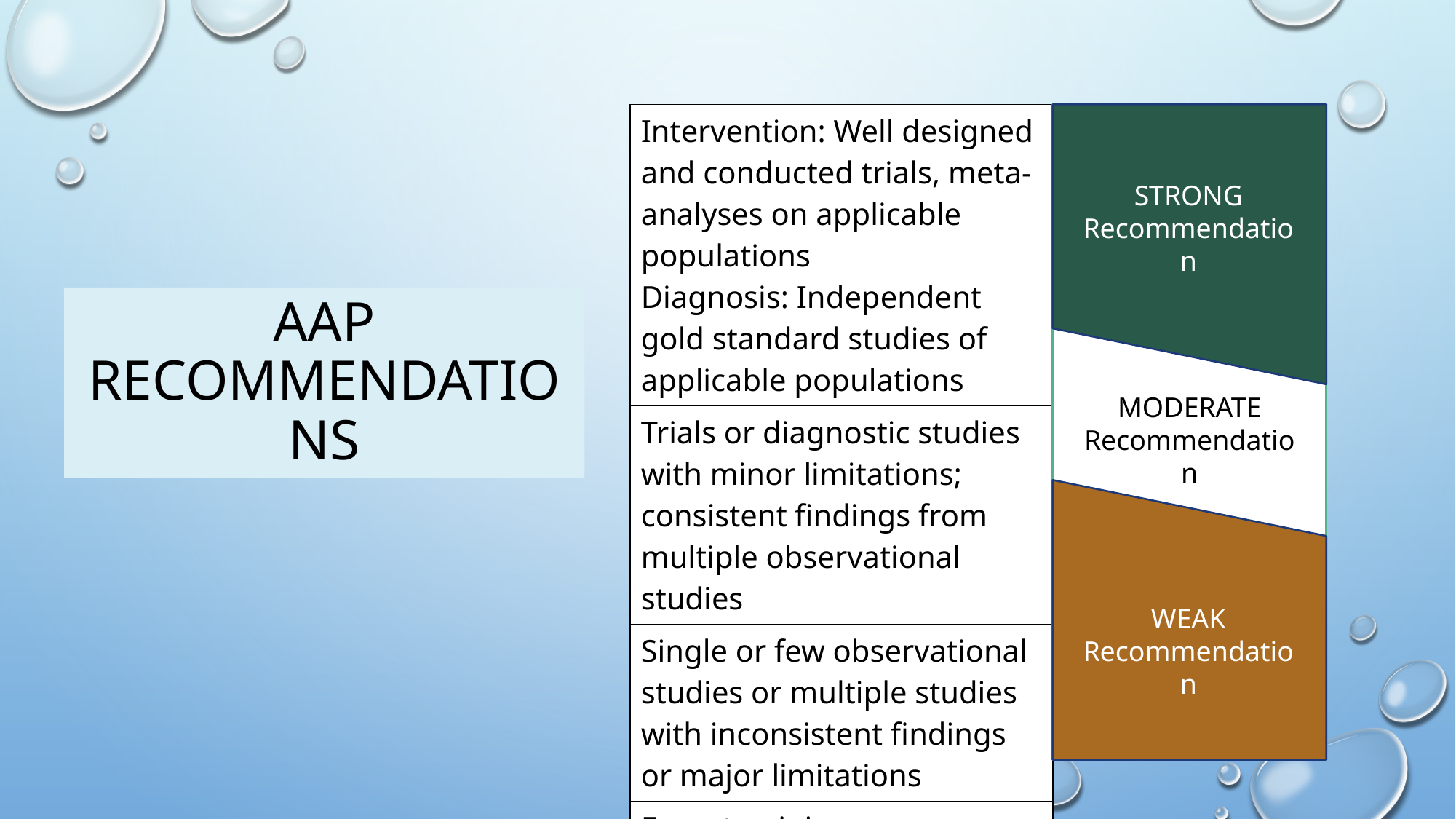

| Intervention: Well designed and conducted trials, meta-analyses on applicable populations Diagnosis: Independent gold standard studies of applicable populations |
| --- |
| Trials or diagnostic studies with minor limitations; consistent findings from multiple observational studies |
| Single or few observational studies or multiple studies with inconsistent findings or major limitations |
| Expert opinion, case reports, reasoning from first principles |
STRONG Recommendation
MODERATE Recommendation
WEAK Recommendation
# AAP Recommendations

## Slide 21
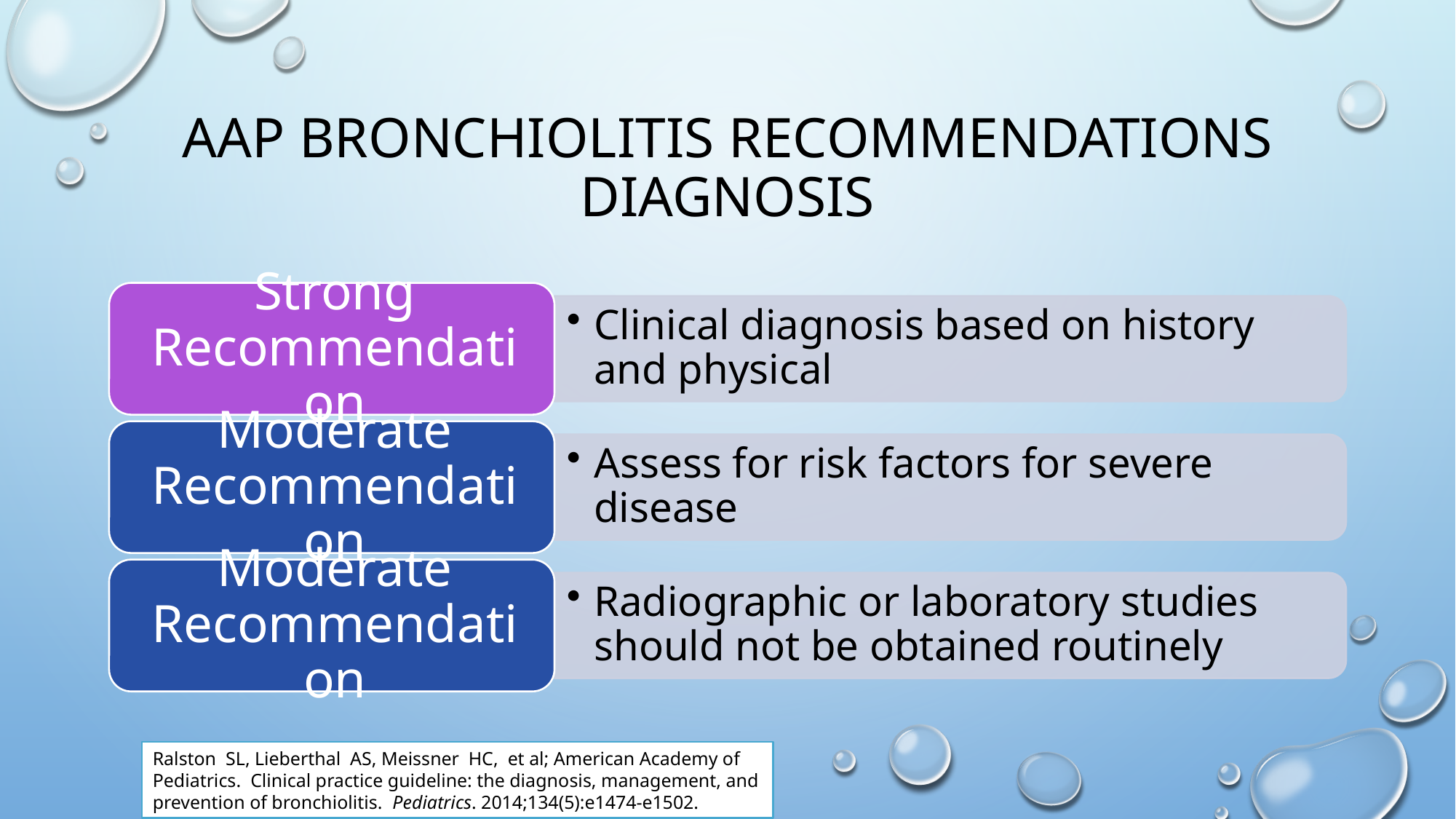

# AAP Bronchiolitis Recommendations Diagnosis
Ralston  SL, Lieberthal  AS, Meissner  HC,  et al; American Academy of Pediatrics.  Clinical practice guideline: the diagnosis, management, and prevention of bronchiolitis.  Pediatrics. 2014;134(5):e1474-e1502.

## Slide 22
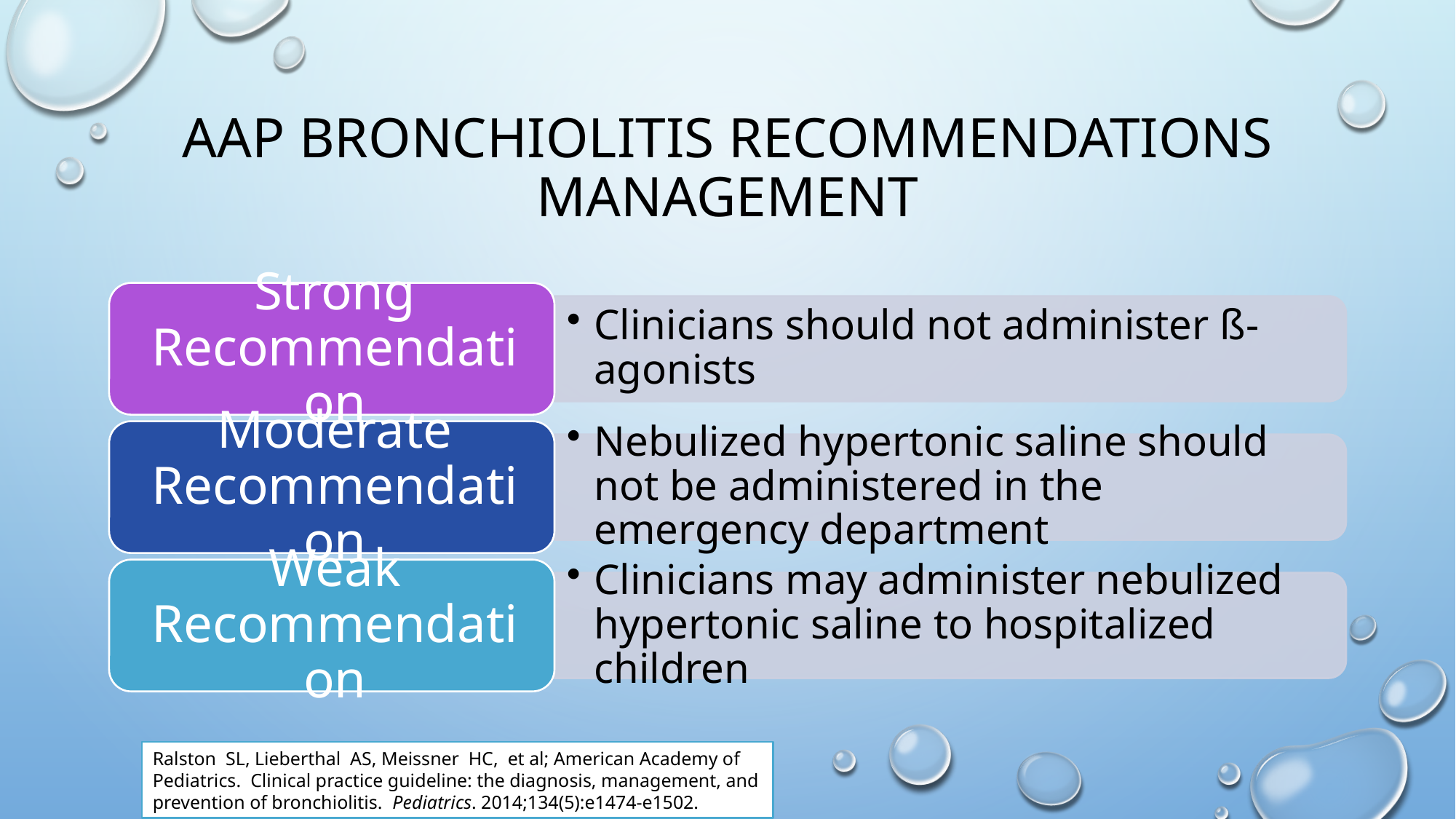

# AAP Bronchiolitis Recommendations Management
Ralston  SL, Lieberthal  AS, Meissner  HC,  et al; American Academy of Pediatrics.  Clinical practice guideline: the diagnosis, management, and prevention of bronchiolitis.  Pediatrics. 2014;134(5):e1474-e1502.

## Slide 23
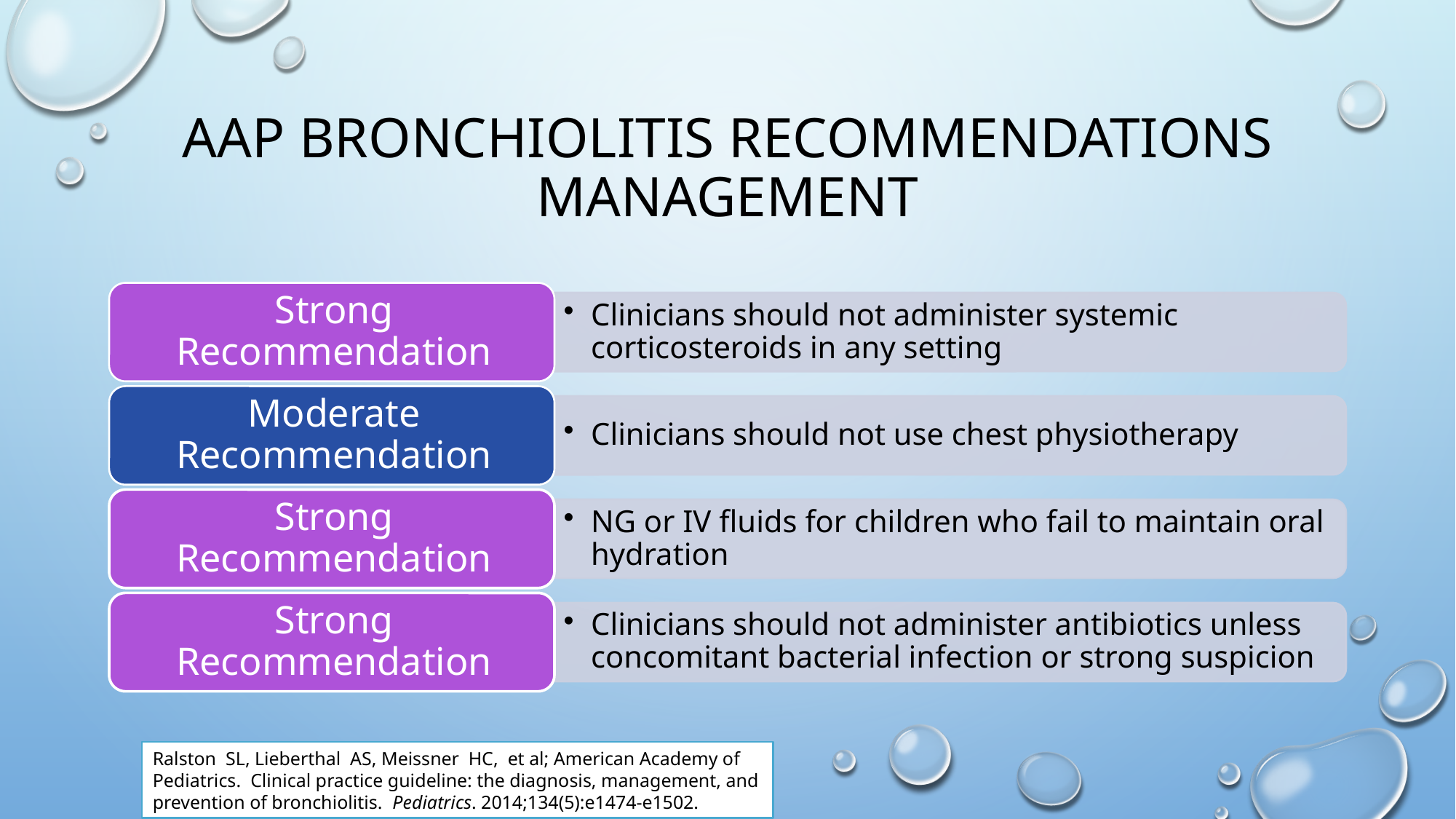

# AAP Bronchiolitis Recommendations Management
Ralston  SL, Lieberthal  AS, Meissner  HC,  et al; American Academy of Pediatrics.  Clinical practice guideline: the diagnosis, management, and prevention of bronchiolitis.  Pediatrics. 2014;134(5):e1474-e1502.
