# Supplementary material for: A Pediatric Emergency Medicine Refresher Course for Generalist Healthcare Providers in Belize: Respiratory Emergencies
Source: J Educ Teach Emerg Med. 2021 Apr 19;6(2):C73–C188. doi: 10.21980/J84063 (PMC10332788; doi:10.21980/J84063)
Supplement: Supplementary file 2 — Please see associated PowerPoint file [file jetem-6-2-c73-AppendixF.pptx]

## Slide 1
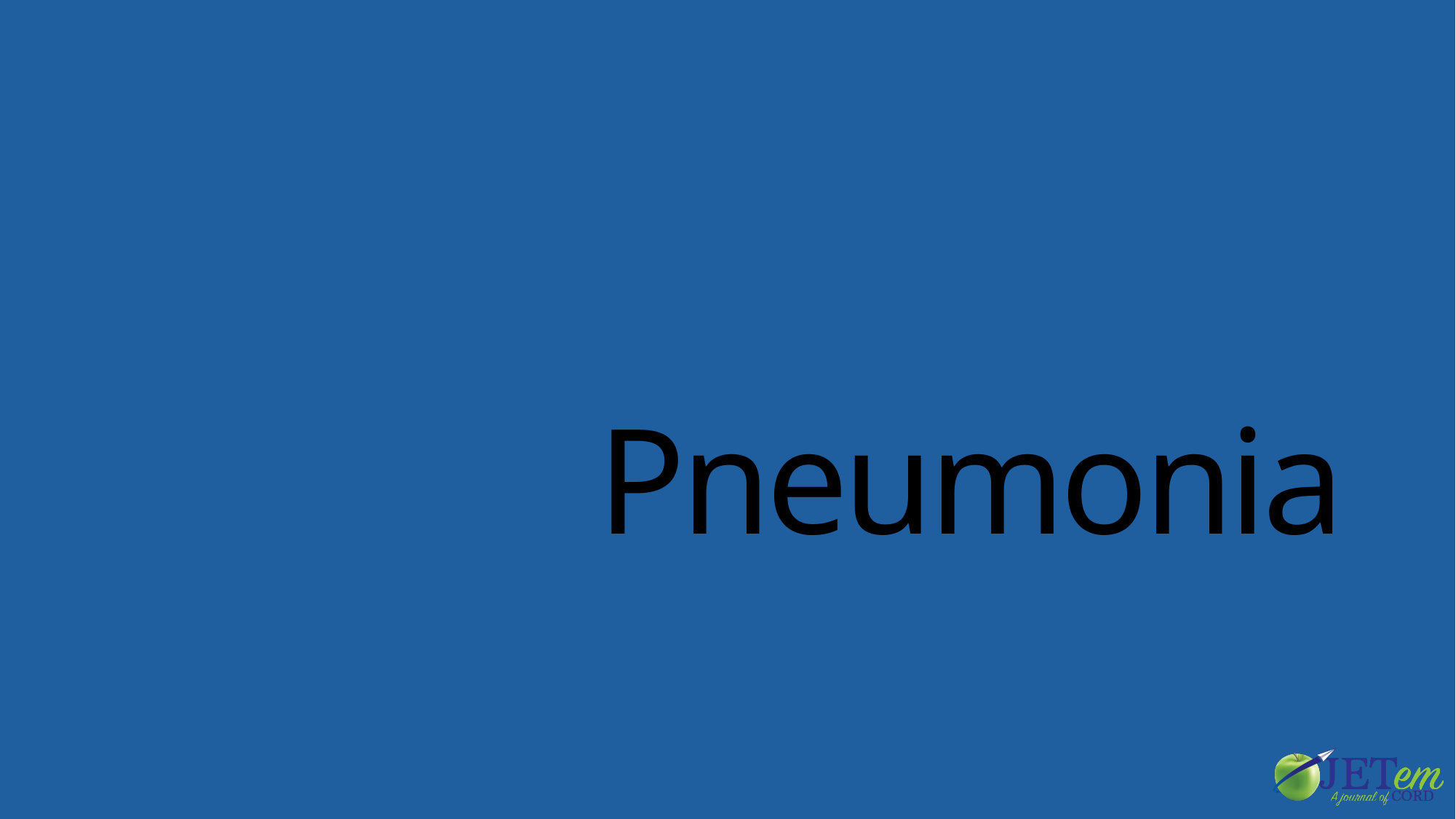

# Pneumonia

## Slide 2
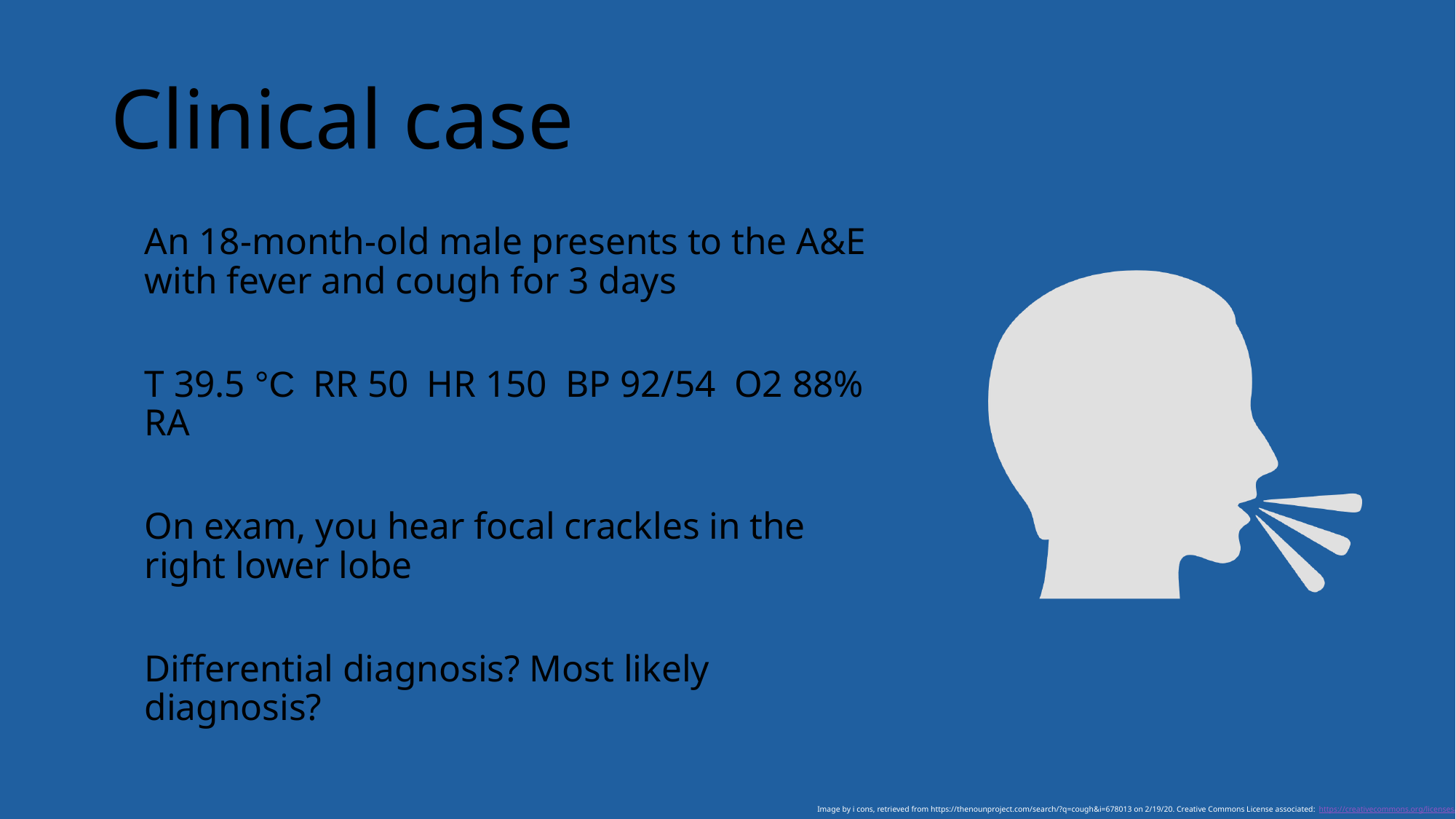

# Clinical case
An 18-month-old male presents to the A&E with fever and cough for 3 days
T 39.5 °C RR 50 HR 150 BP 92/54 O2 88% RA
On exam, you hear focal crackles in the right lower lobe
Differential diagnosis? Most likely diagnosis?
Image by i cons, retrieved from https://thenounproject.com/search/?q=cough&i=678013 on 2/19/20. Creative Commons License associated: https://creativecommons.org/licenses/by/3.0/us/legalcode.

## Slide 3
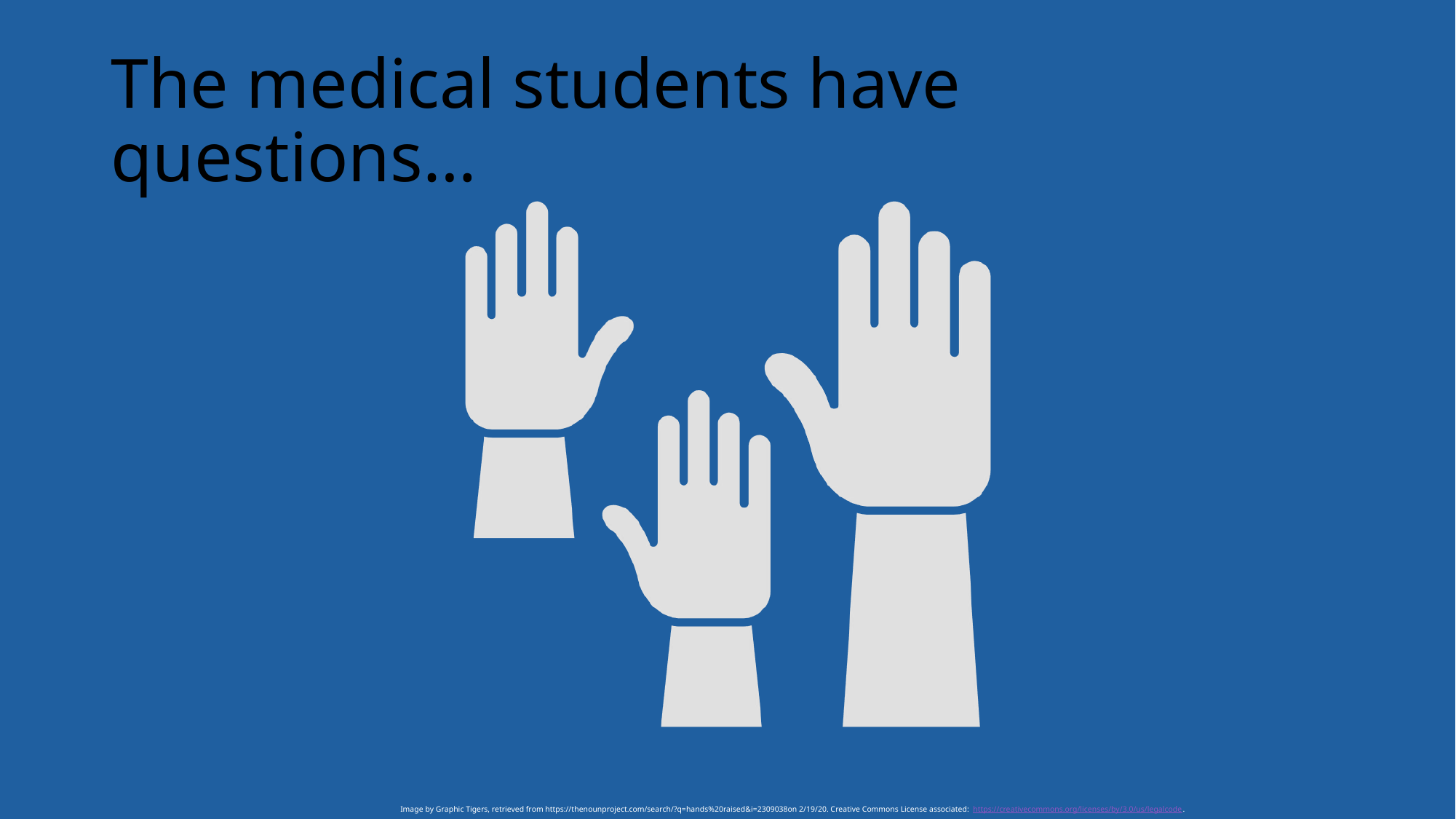

# The medical students have questions…
Image by Graphic Tigers, retrieved from https://thenounproject.com/search/?q=hands%20raised&i=2309038on 2/19/20. Creative Commons License associated: https://creativecommons.org/licenses/by/3.0/us/legalcode.

## Slide 4
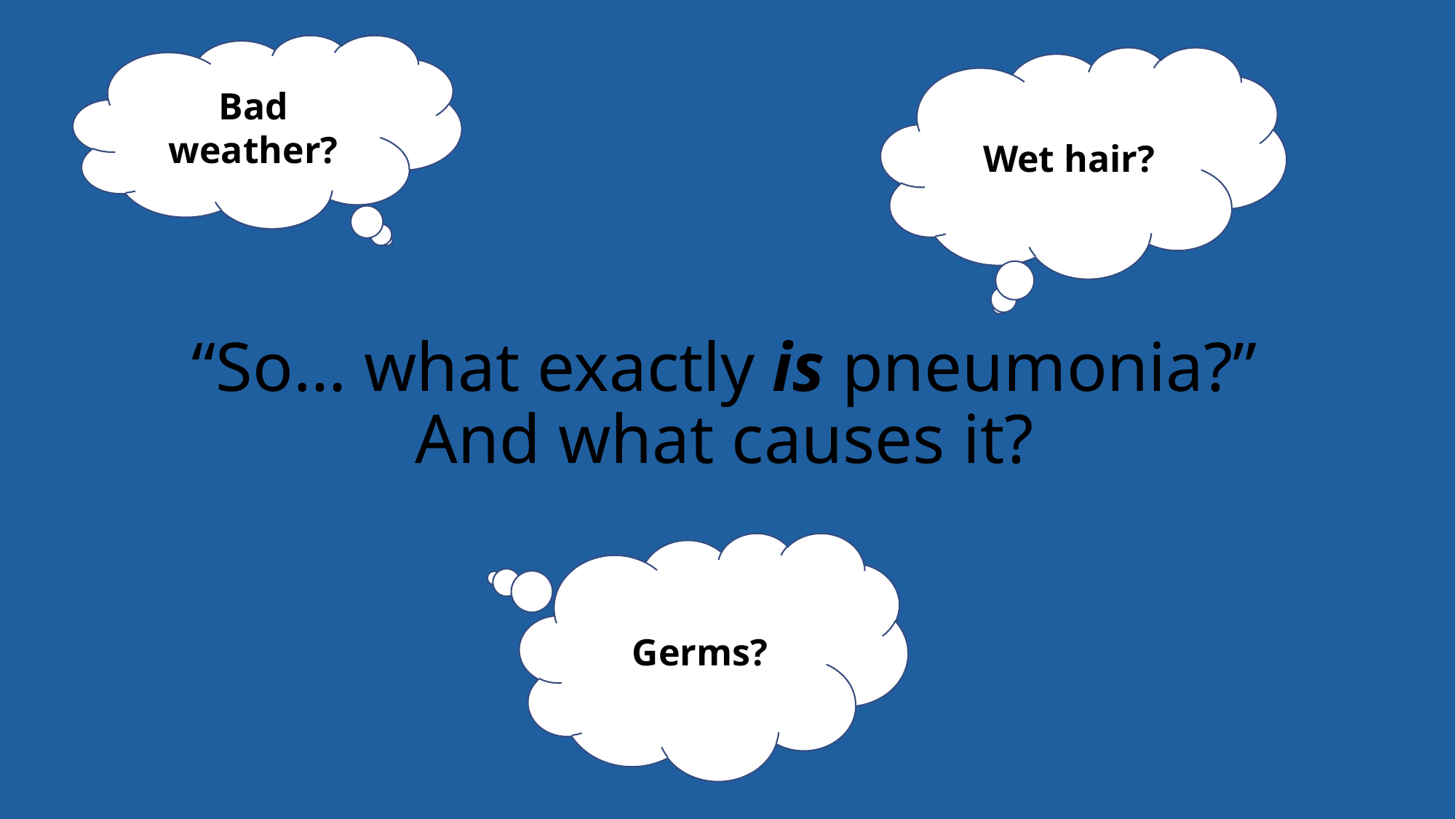

Bad weather?
Wet hair?
# “So… what exactly is pneumonia?” And what causes it?
Germs?

## Slide 5
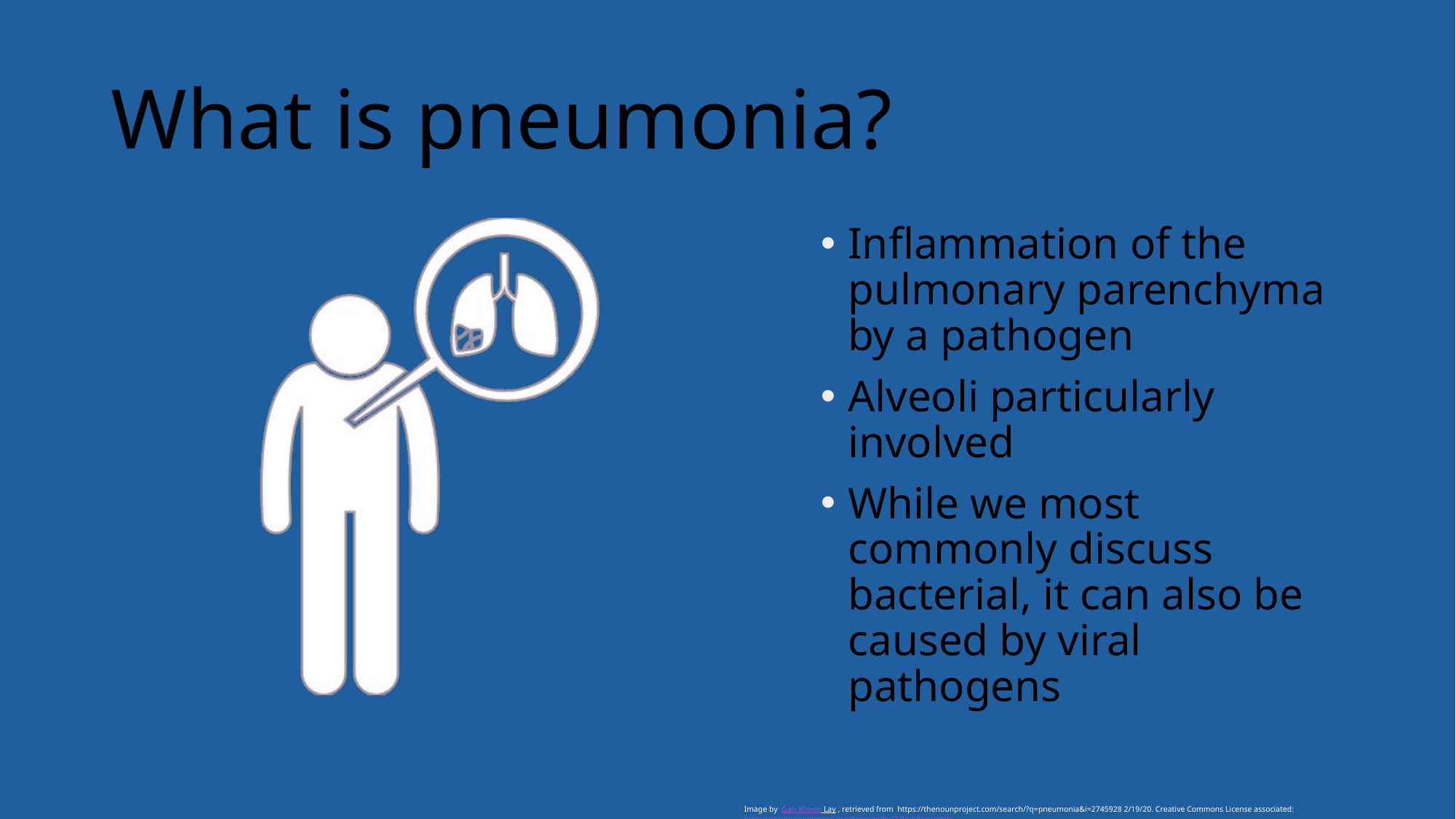

# What is pneumonia?
Inflammation of the pulmonary parenchyma by a pathogen
Alveoli particularly involved
While we most commonly discuss bacterial, it can also be caused by viral pathogens
Image by Gan Khoon Lay , retrieved from  https://thenounproject.com/search/?q=pneumonia&i=2745928 2/19/20. Creative Commons License associated: https://creativecommons.org/licenses/by/3.0/us/legalcode.

## Slide 6
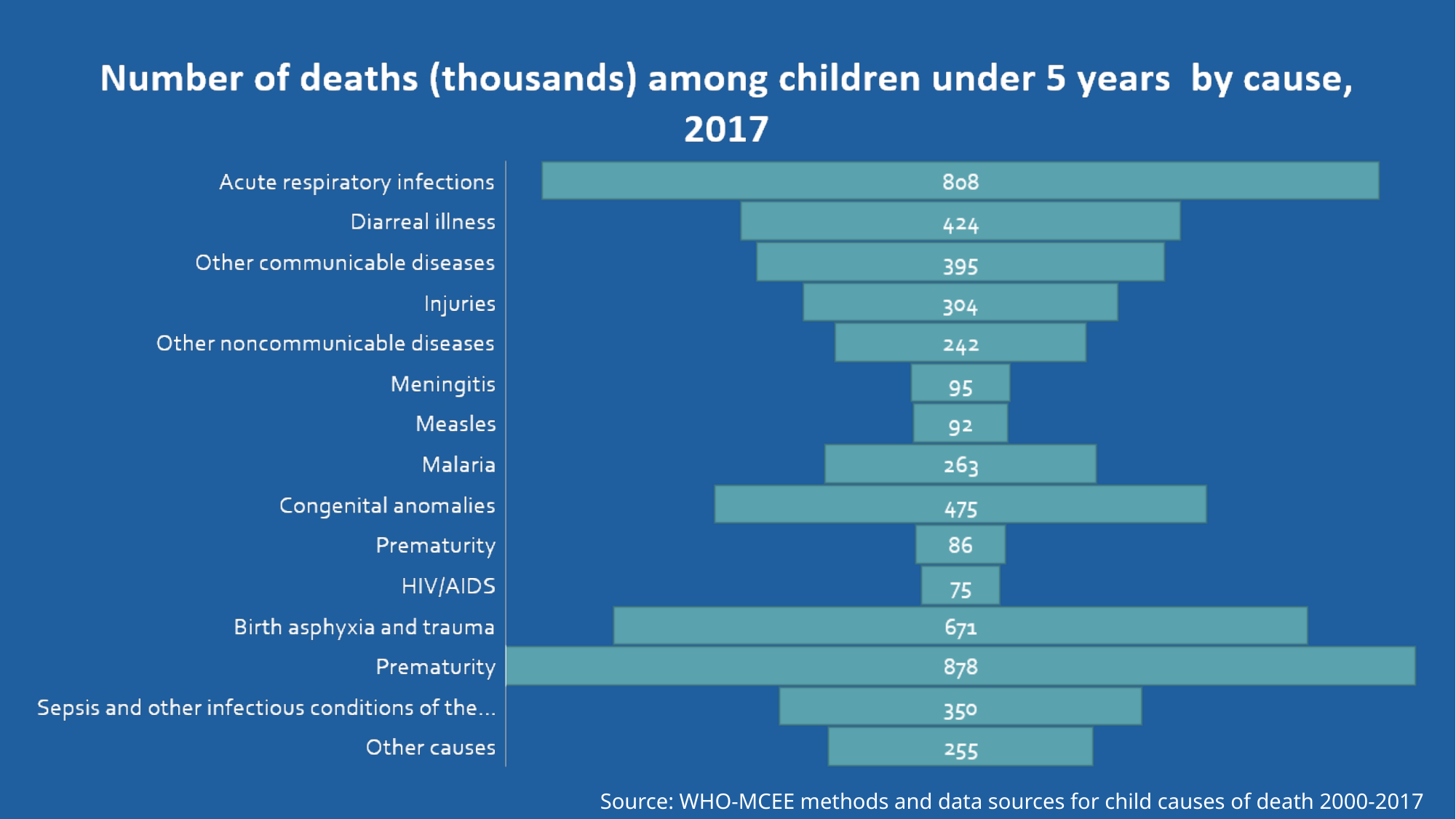

Source: WHO-MCEE methods and data sources for child causes of death 2000-2017

## Slide 7
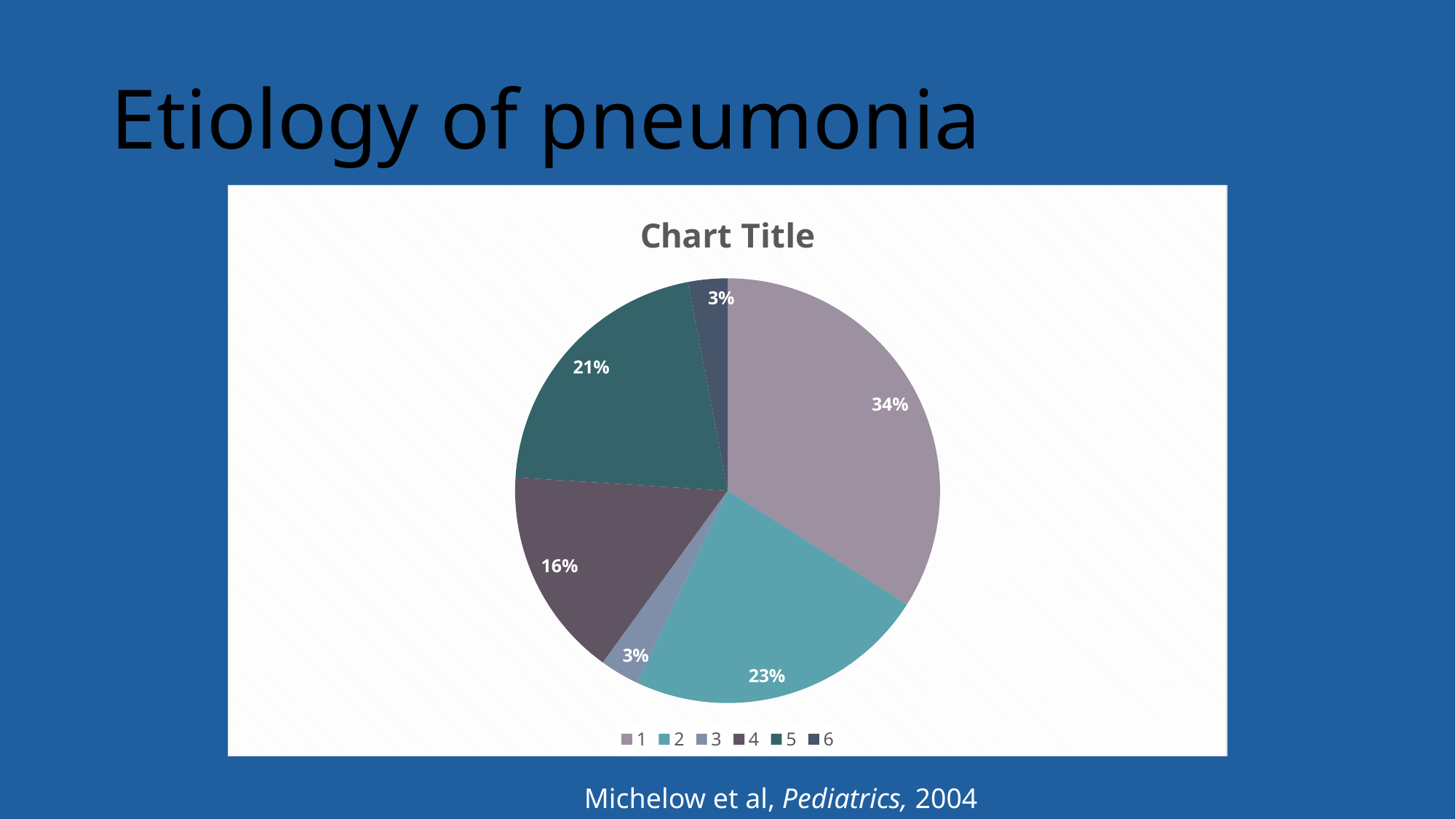

# Etiology of pneumonia
### Chart:
| Category | |
|---|---|Michelow et al, Pediatrics, 2004

## Slide 8
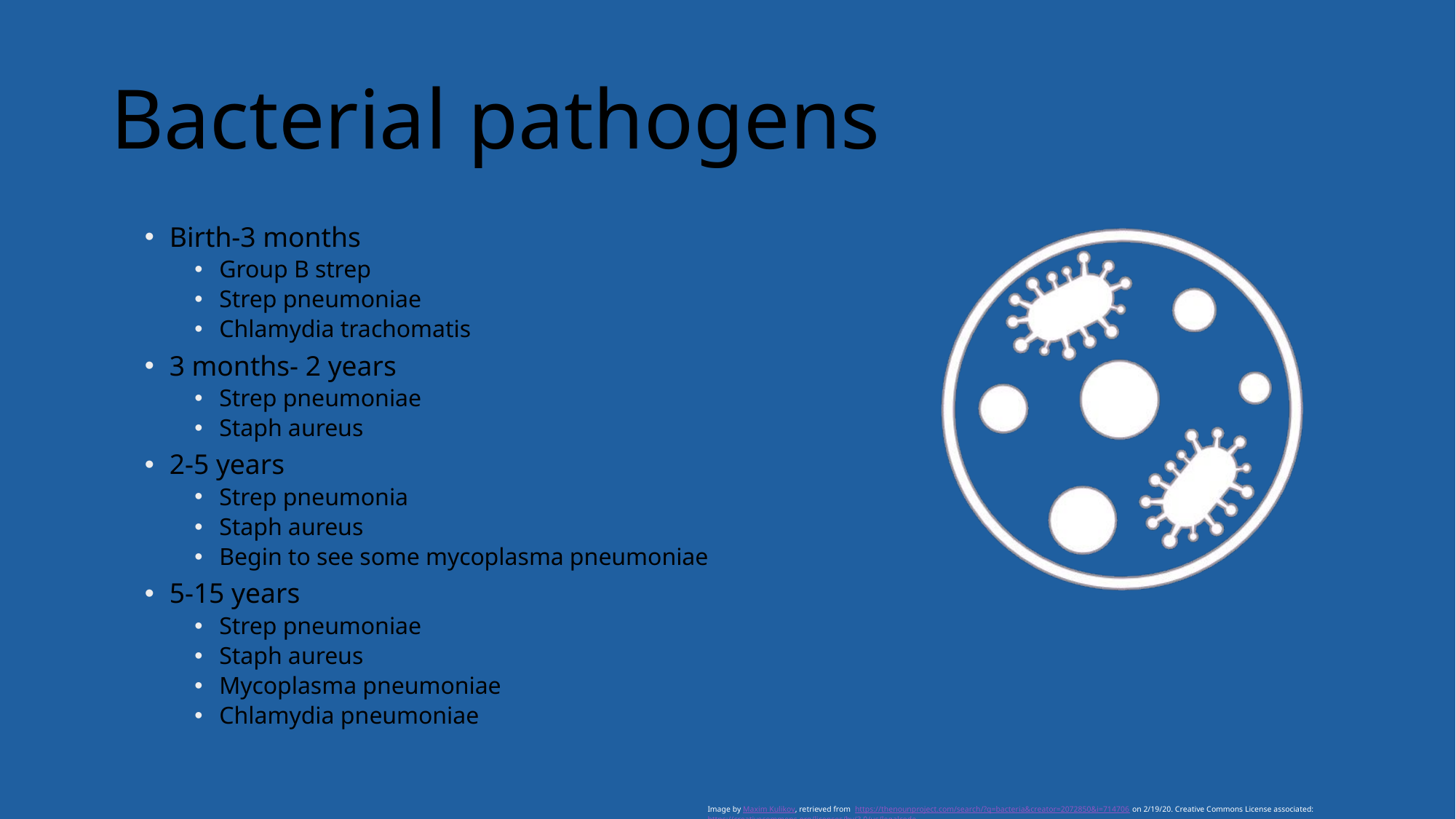

# Bacterial pathogens
Birth-3 months
Group B strep
Strep pneumoniae
Chlamydia trachomatis
3 months- 2 years
Strep pneumoniae
Staph aureus
2-5 years
Strep pneumonia
Staph aureus
Begin to see some mycoplasma pneumoniae
5-15 years
Strep pneumoniae
Staph aureus
Mycoplasma pneumoniae
Chlamydia pneumoniae
Image by Maxim Kulikov, retrieved from  https://thenounproject.com/search/?q=bacteria&creator=2072850&i=714706 on 2/19/20. Creative Commons License associated: https://creativecommons.org/licenses/by/3.0/us/legalcode.

## Slide 9
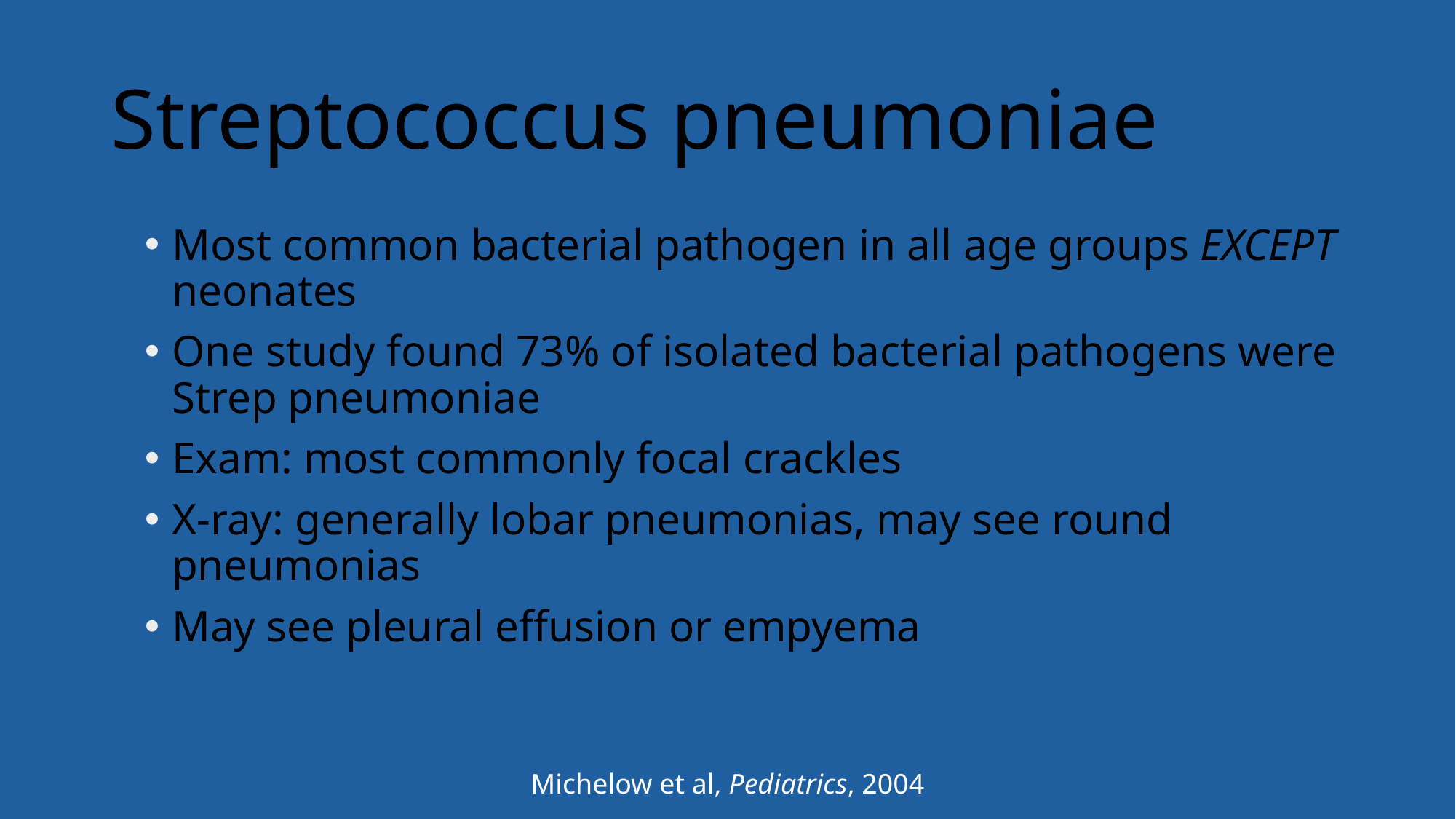

# Streptococcus pneumoniae
Most common bacterial pathogen in all age groups EXCEPT neonates
One study found 73% of isolated bacterial pathogens were Strep pneumoniae
Exam: most commonly focal crackles
X-ray: generally lobar pneumonias, may see round pneumonias
May see pleural effusion or empyema
Michelow et al, Pediatrics, 2004

## Slide 10
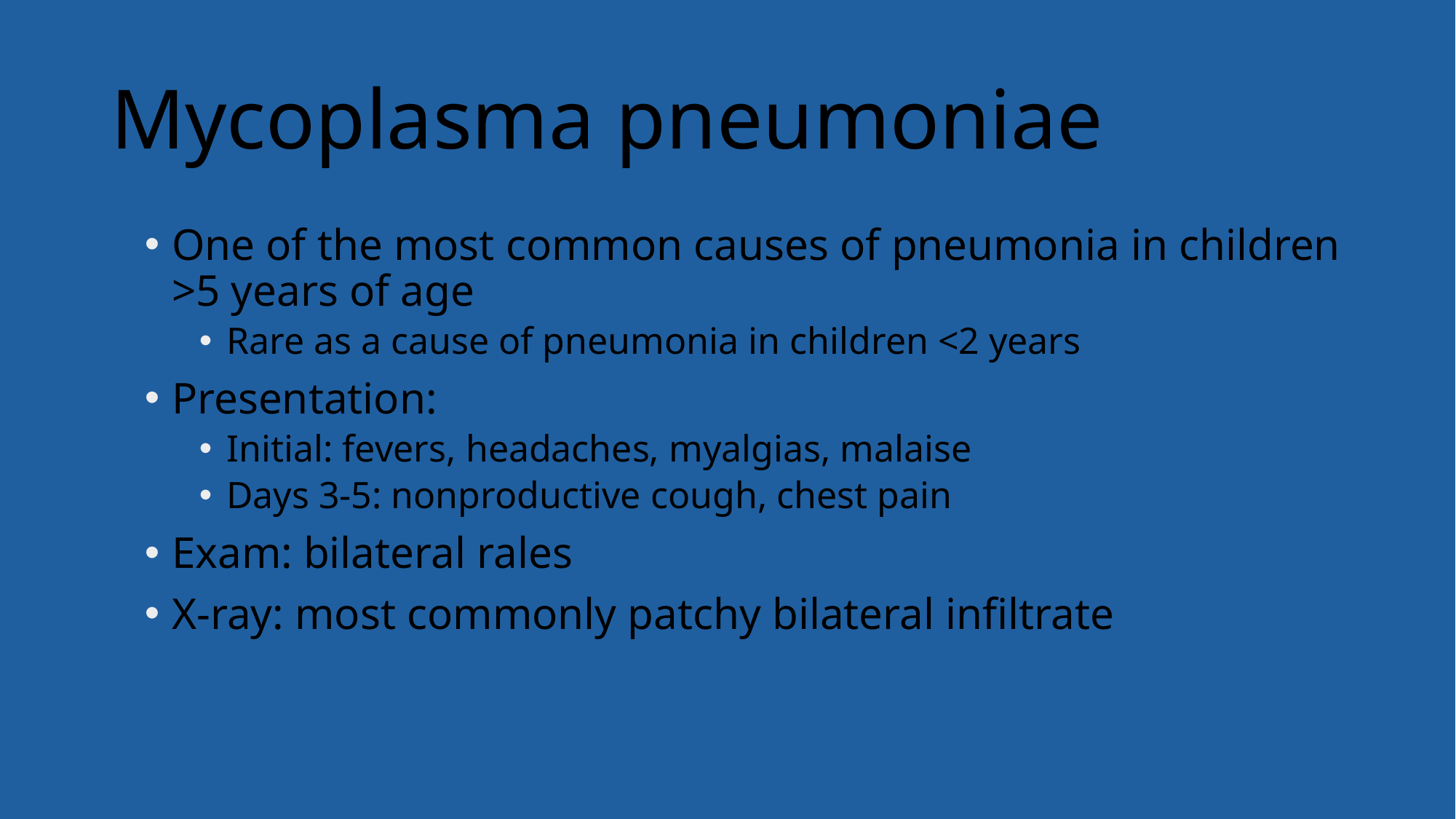

# Mycoplasma pneumoniae
One of the most common causes of pneumonia in children >5 years of age
Rare as a cause of pneumonia in children <2 years
Presentation:
Initial: fevers, headaches, myalgias, malaise
Days 3-5: nonproductive cough, chest pain
Exam: bilateral rales
X-ray: most commonly patchy bilateral infiltrate

## Slide 11
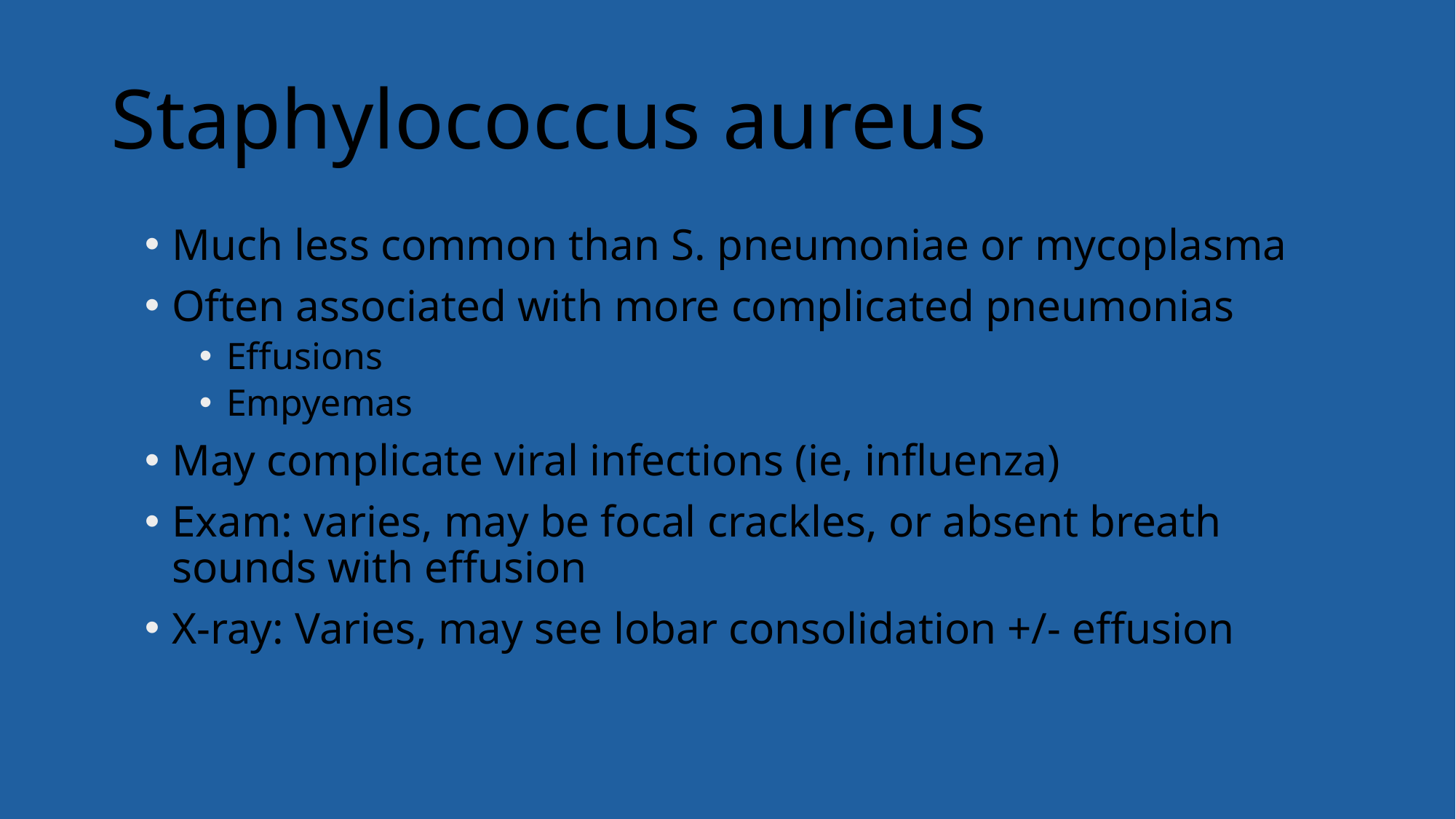

# Staphylococcus aureus
Much less common than S. pneumoniae or mycoplasma
Often associated with more complicated pneumonias
Effusions
Empyemas
May complicate viral infections (ie, influenza)
Exam: varies, may be focal crackles, or absent breath sounds with effusion
X-ray: Varies, may see lobar consolidation +/- effusion

## Slide 12
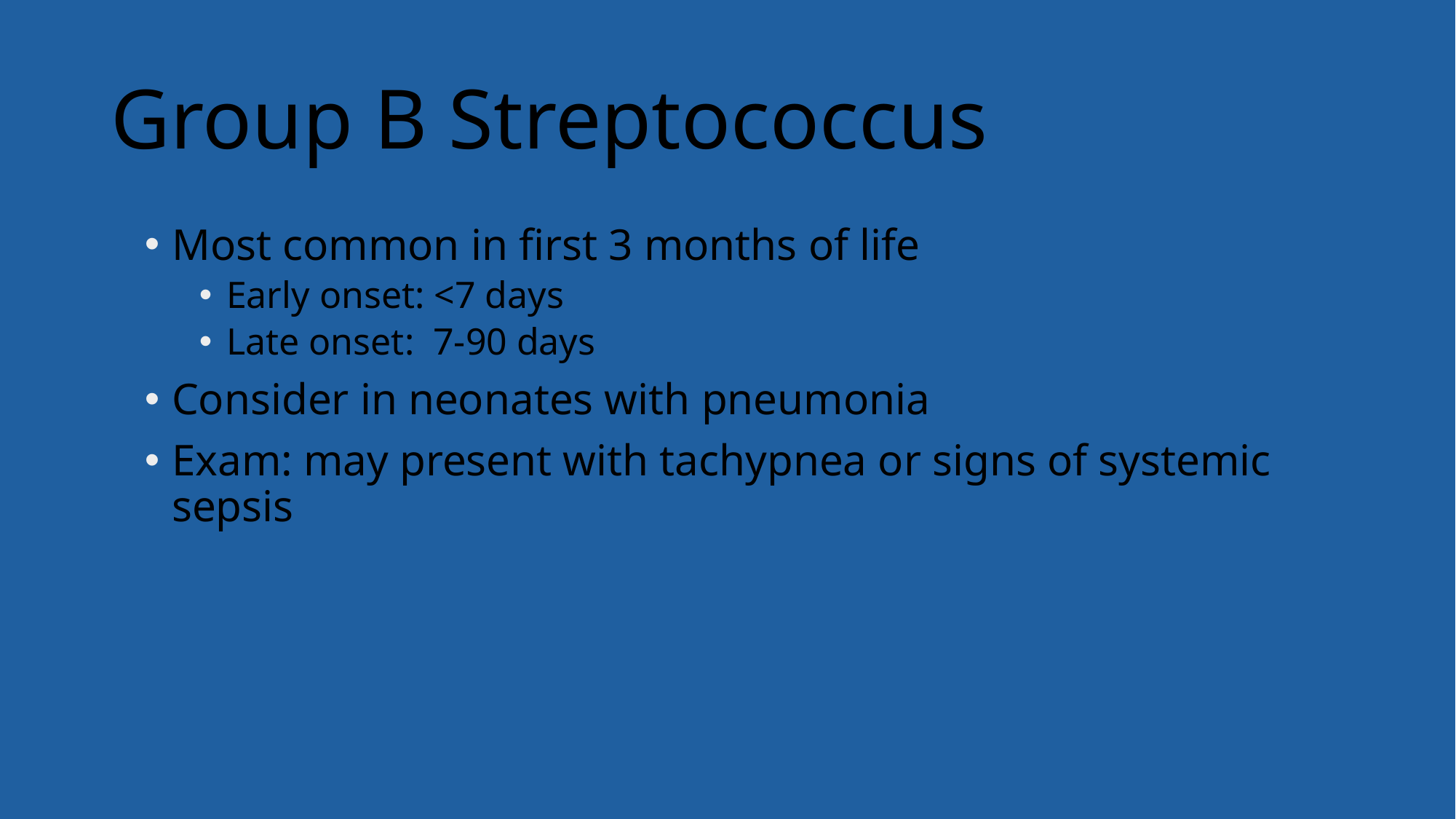

# Group B Streptococcus
Most common in first 3 months of life
Early onset: <7 days
Late onset: 7-90 days
Consider in neonates with pneumonia
Exam: may present with tachypnea or signs of systemic sepsis

## Slide 13
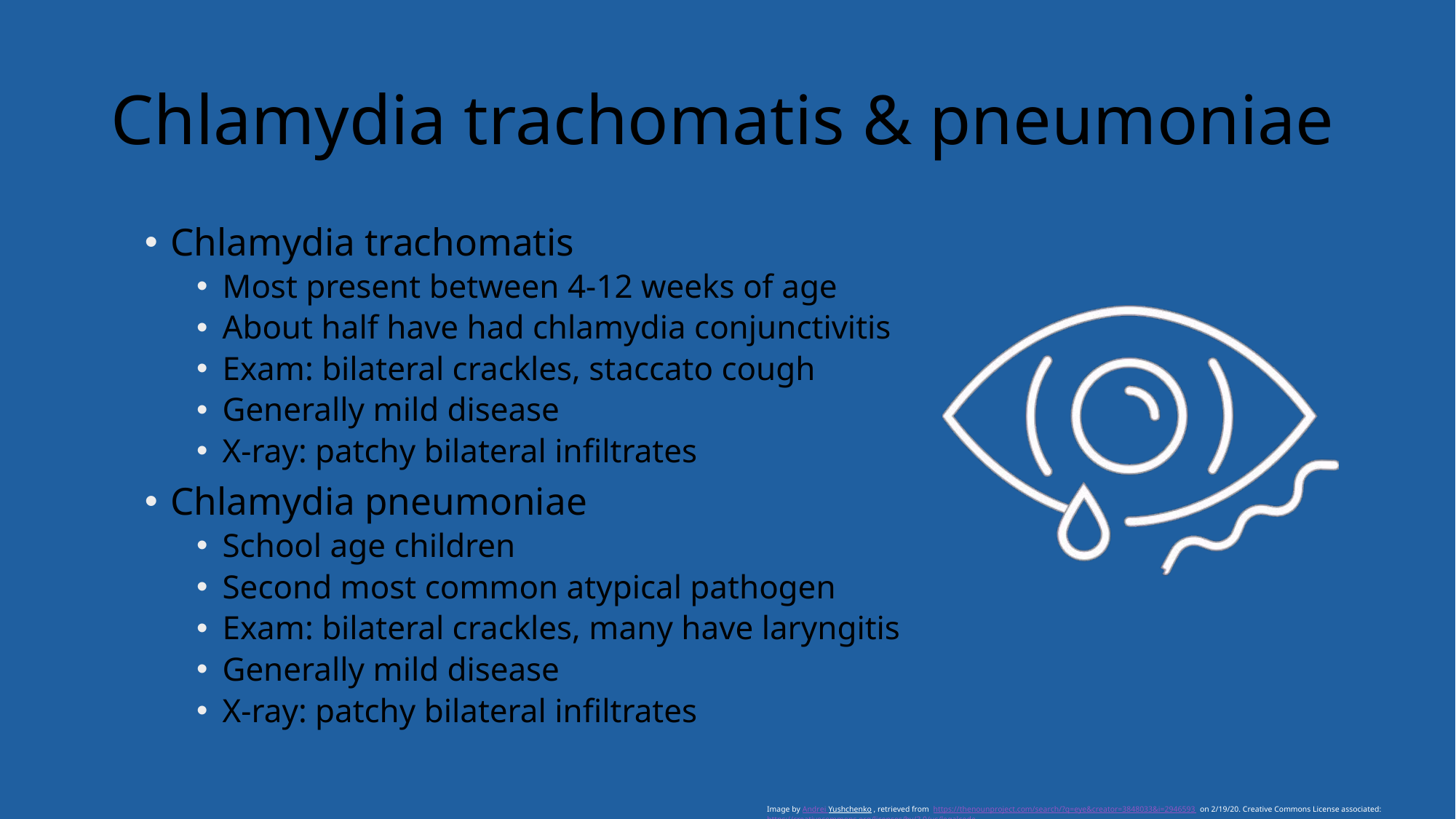

# Chlamydia trachomatis & pneumoniae
Chlamydia trachomatis
Most present between 4-12 weeks of age
About half have had chlamydia conjunctivitis
Exam: bilateral crackles, staccato cough
Generally mild disease
X-ray: patchy bilateral infiltrates
Chlamydia pneumoniae
School age children
Second most common atypical pathogen
Exam: bilateral crackles, many have laryngitis
Generally mild disease
X-ray: patchy bilateral infiltrates
Image by Andrei Yushchenko , retrieved from  https://thenounproject.com/search/?q=eye&creator=3848033&i=2946593 on 2/19/20. Creative Commons License associated: https://creativecommons.org/licenses/by/3.0/us/legalcode.

## Slide 14
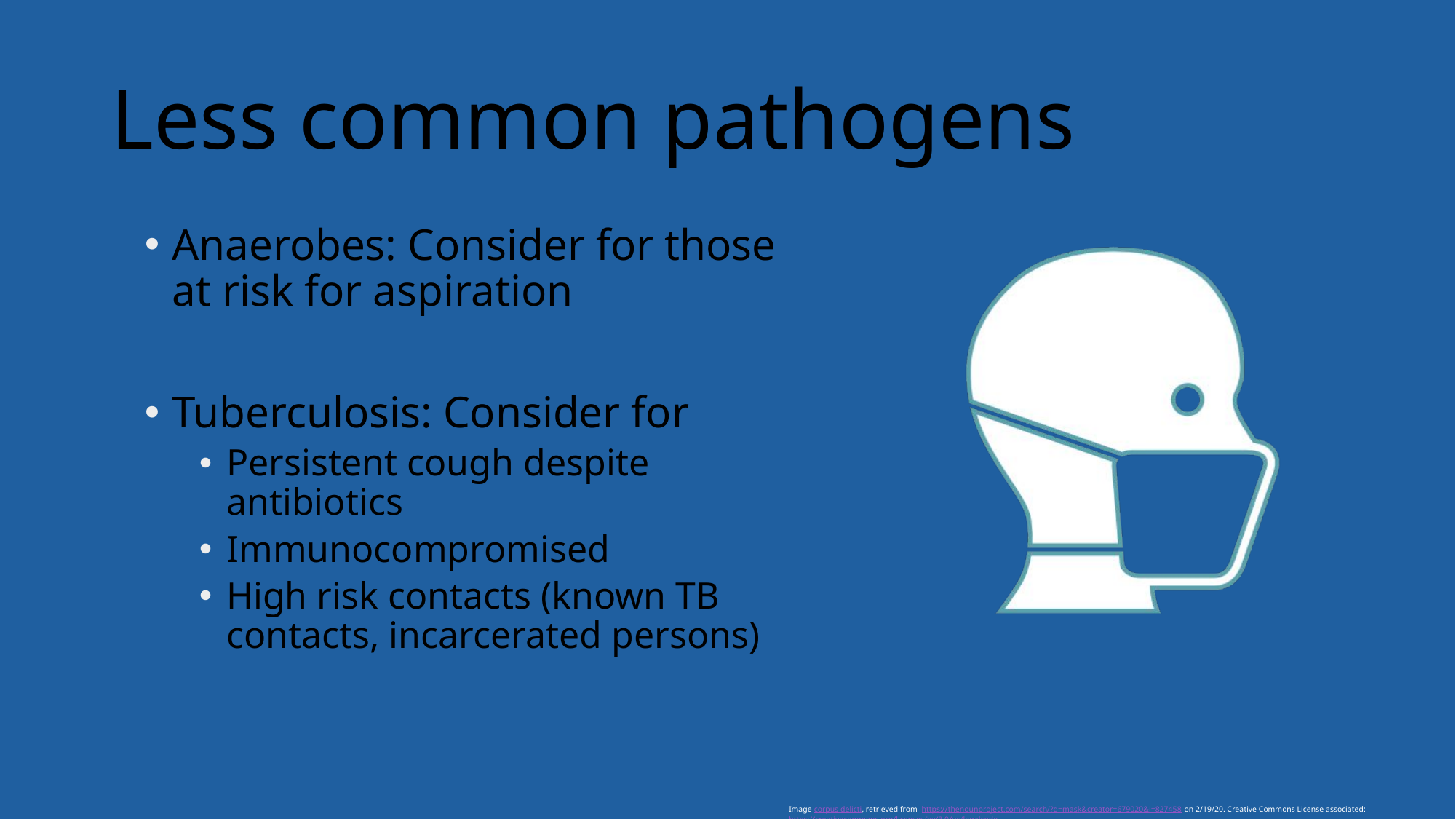

# Less common pathogens
Anaerobes: Consider for those at risk for aspiration
Tuberculosis: Consider for
Persistent cough despite antibiotics
Immunocompromised
High risk contacts (known TB contacts, incarcerated persons)
Image corpus delicti, retrieved from  https://thenounproject.com/search/?q=mask&creator=679020&i=827458 on 2/19/20. Creative Commons License associated: https://creativecommons.org/licenses/by/3.0/us/legalcode.

## Slide 15
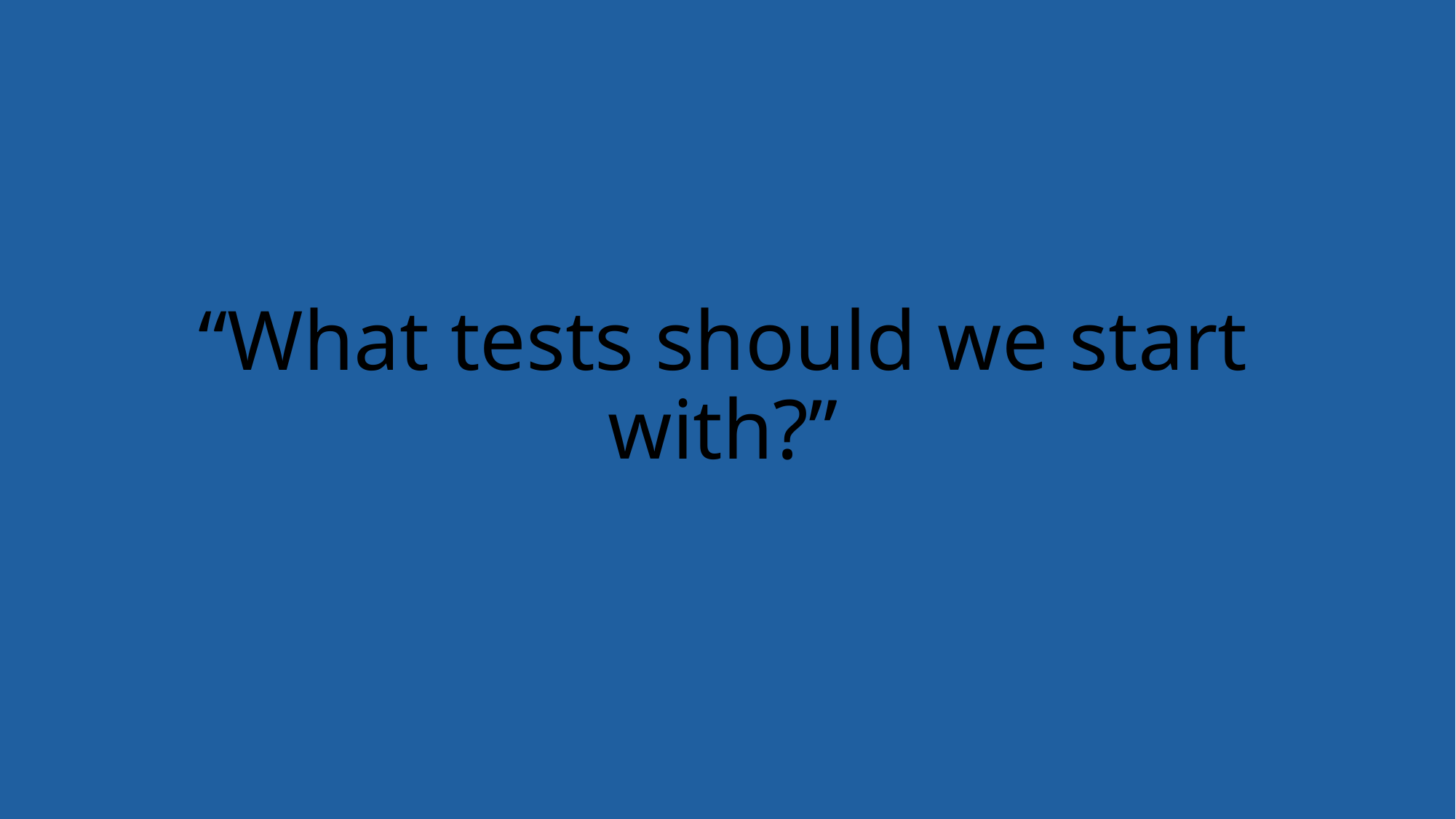

# “What tests should we start with?”

## Slide 16
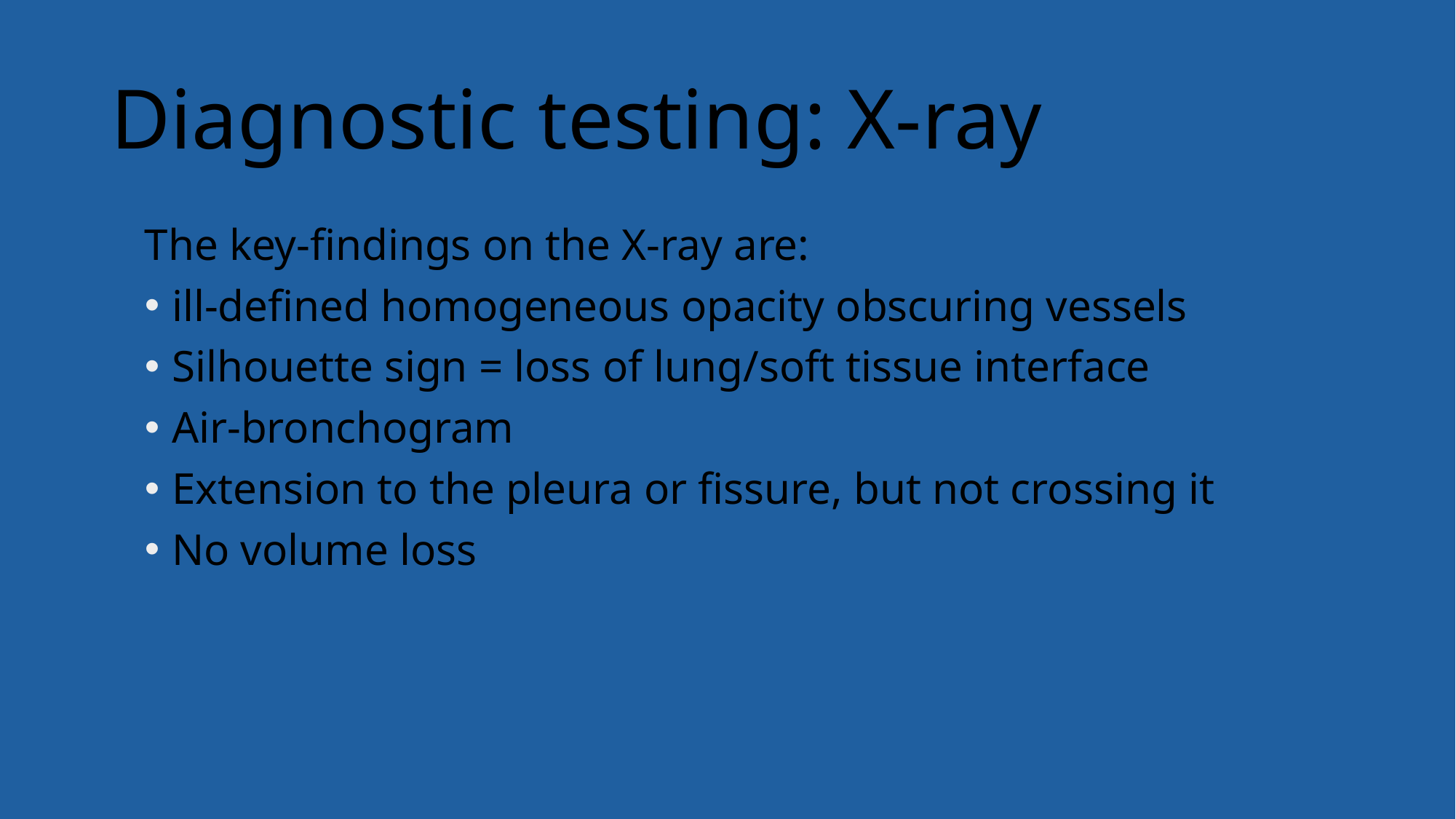

# Diagnostic testing: X-ray
The key-findings on the X-ray are:
ill-defined homogeneous opacity obscuring vessels
Silhouette sign = loss of lung/soft tissue interface
Air-bronchogram
Extension to the pleura or fissure, but not crossing it
No volume loss

## Slide 17
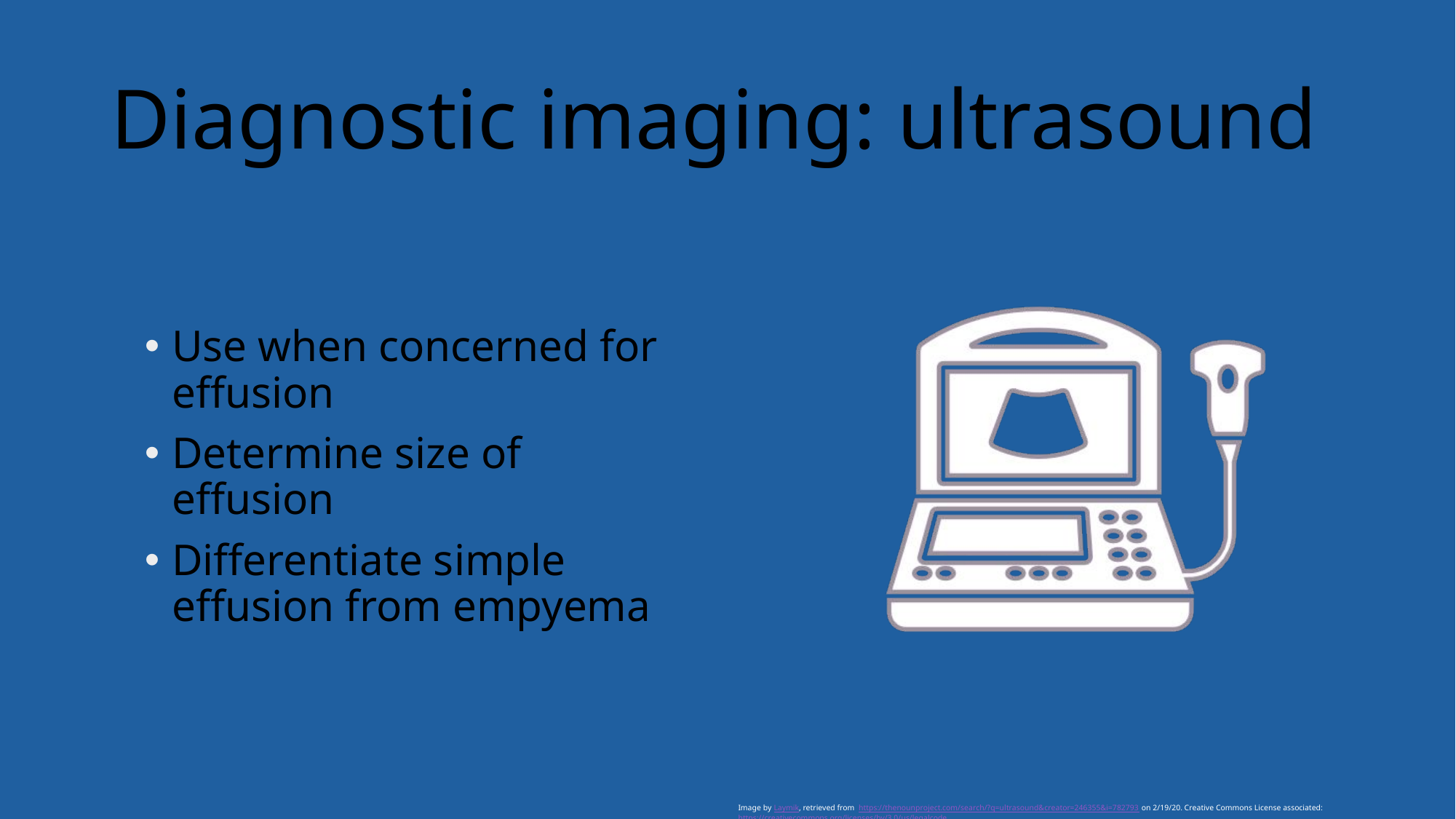

# Diagnostic imaging: ultrasound
Use when concerned for effusion
Determine size of effusion
Differentiate simple effusion from empyema
Image by Laymik, retrieved from  https://thenounproject.com/search/?q=ultrasound&creator=246355&i=782793 on 2/19/20. Creative Commons License associated: https://creativecommons.org/licenses/by/3.0/us/legalcode.

## Slide 18
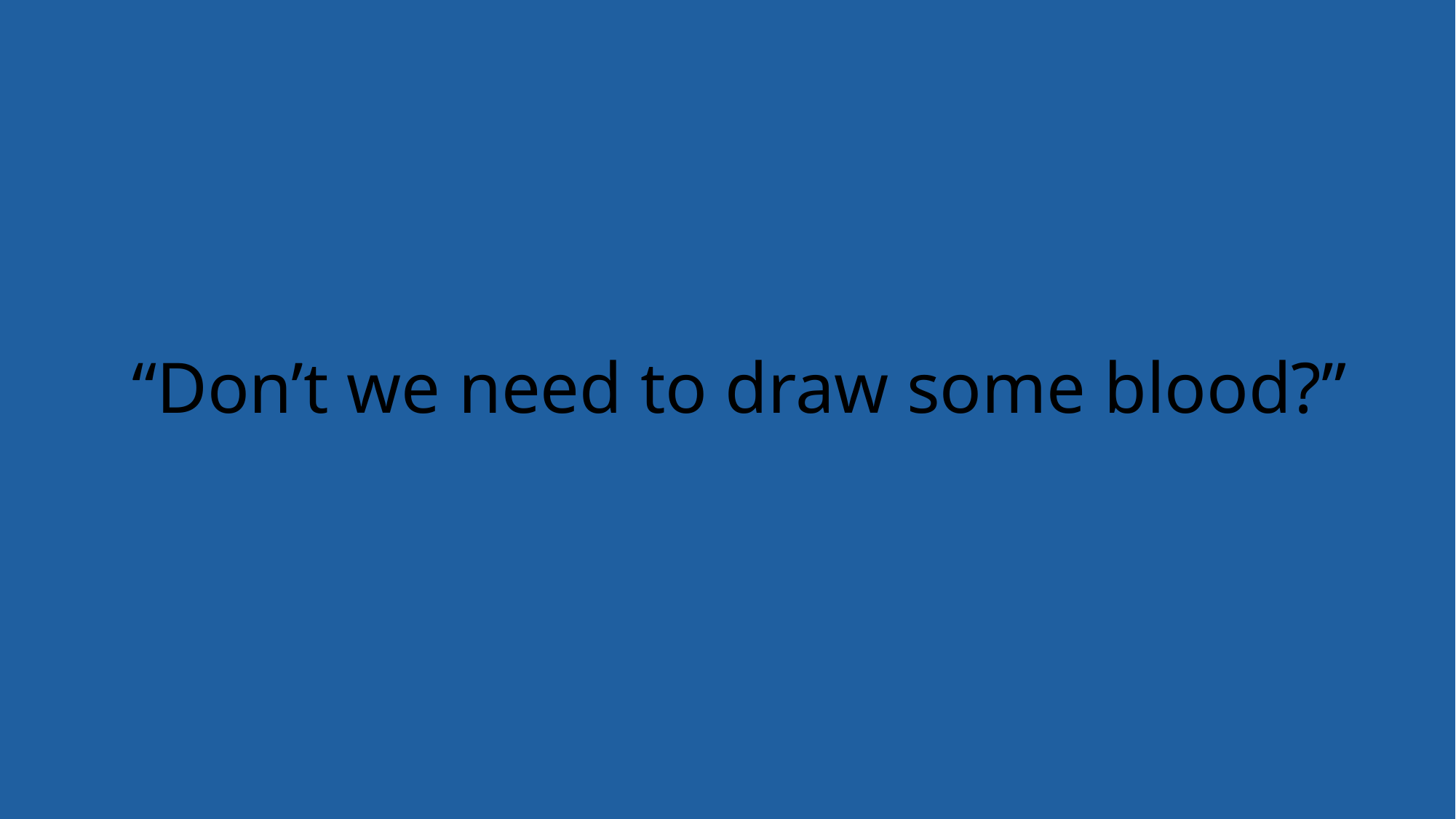

# “Don’t we need to draw some blood?”

## Slide 19
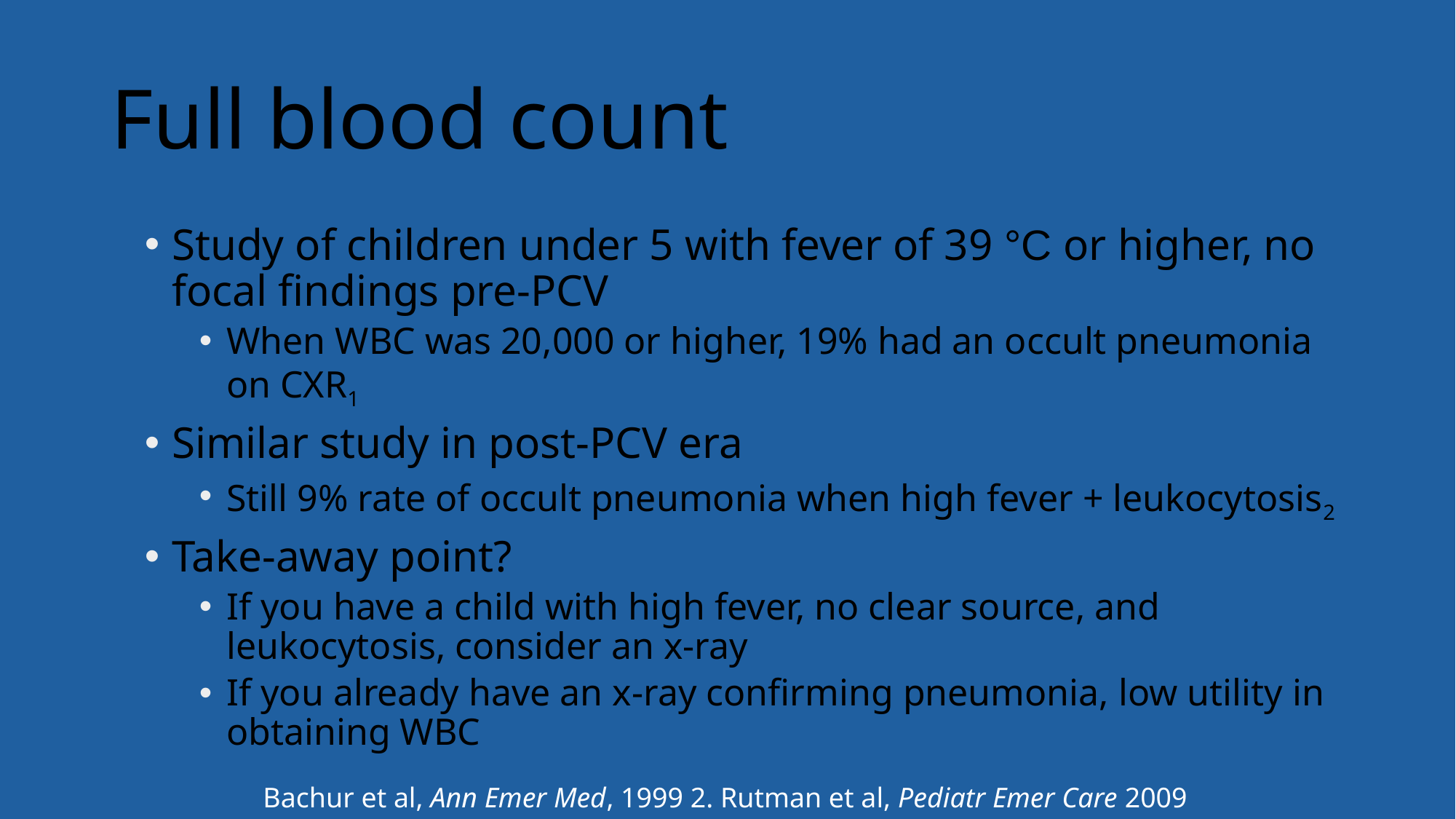

# Full blood count
Study of children under 5 with fever of 39 °C or higher, no focal findings pre-PCV
When WBC was 20,000 or higher, 19% had an occult pneumonia on CXR1
Similar study in post-PCV era
Still 9% rate of occult pneumonia when high fever + leukocytosis2
Take-away point?
If you have a child with high fever, no clear source, and leukocytosis, consider an x-ray
If you already have an x-ray confirming pneumonia, low utility in obtaining WBC
Bachur et al, Ann Emer Med, 1999 2. Rutman et al, Pediatr Emer Care 2009

## Slide 20
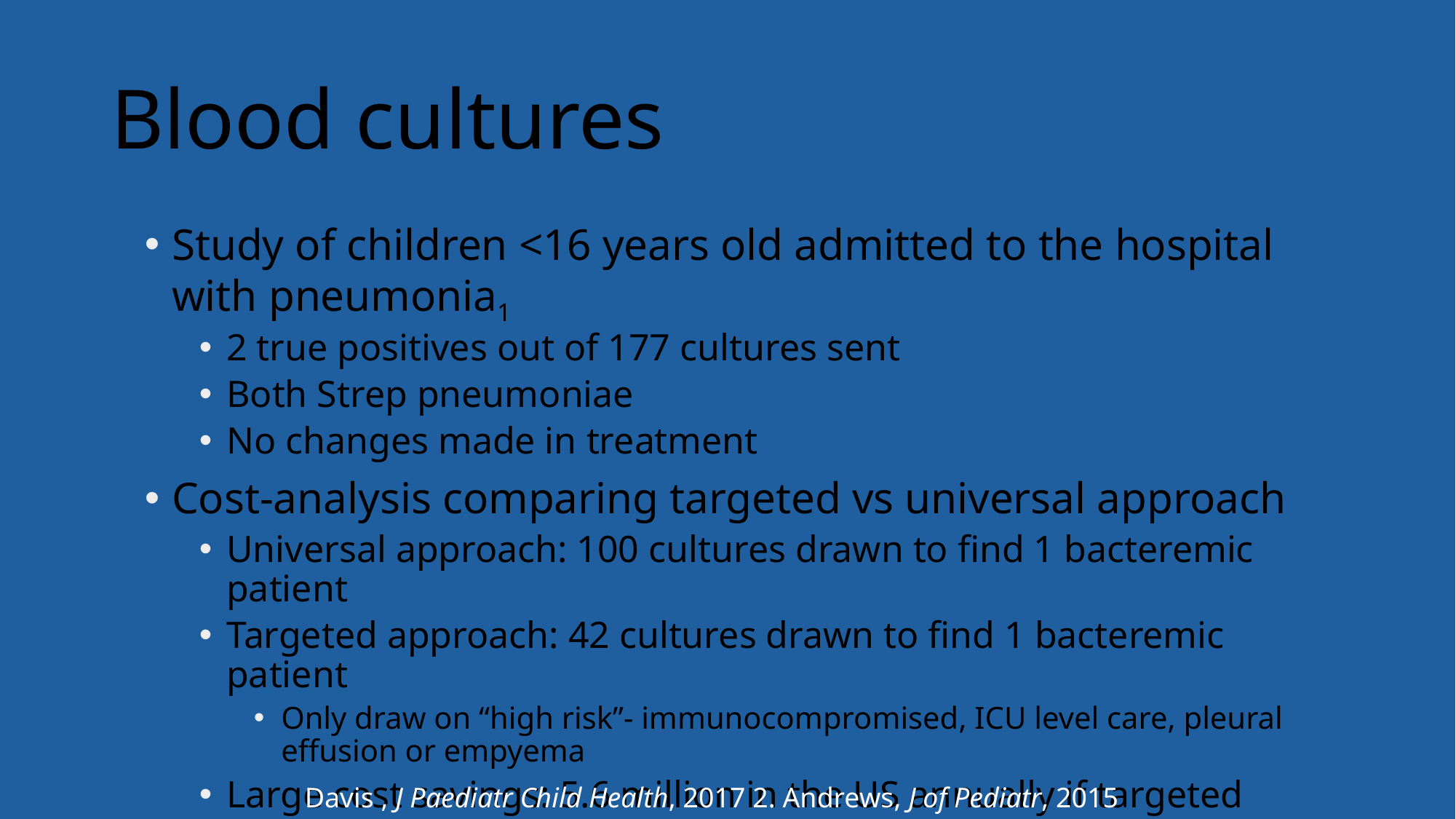

# Blood cultures
Study of children <16 years old admitted to the hospital with pneumonia1
2 true positives out of 177 cultures sent
Both Strep pneumoniae
No changes made in treatment
Cost-analysis comparing targeted vs universal approach
Universal approach: 100 cultures drawn to find 1 bacteremic patient
Targeted approach: 42 cultures drawn to find 1 bacteremic patient
Only draw on “high risk”- immunocompromised, ICU level care, pleural effusion or empyema
Large cost savings: 5.6 million in the US annually if targeted approach used
Davis , J Paediatr Child Health, 2017 2. Andrews, J of Pediatr, 2015

## Slide 21
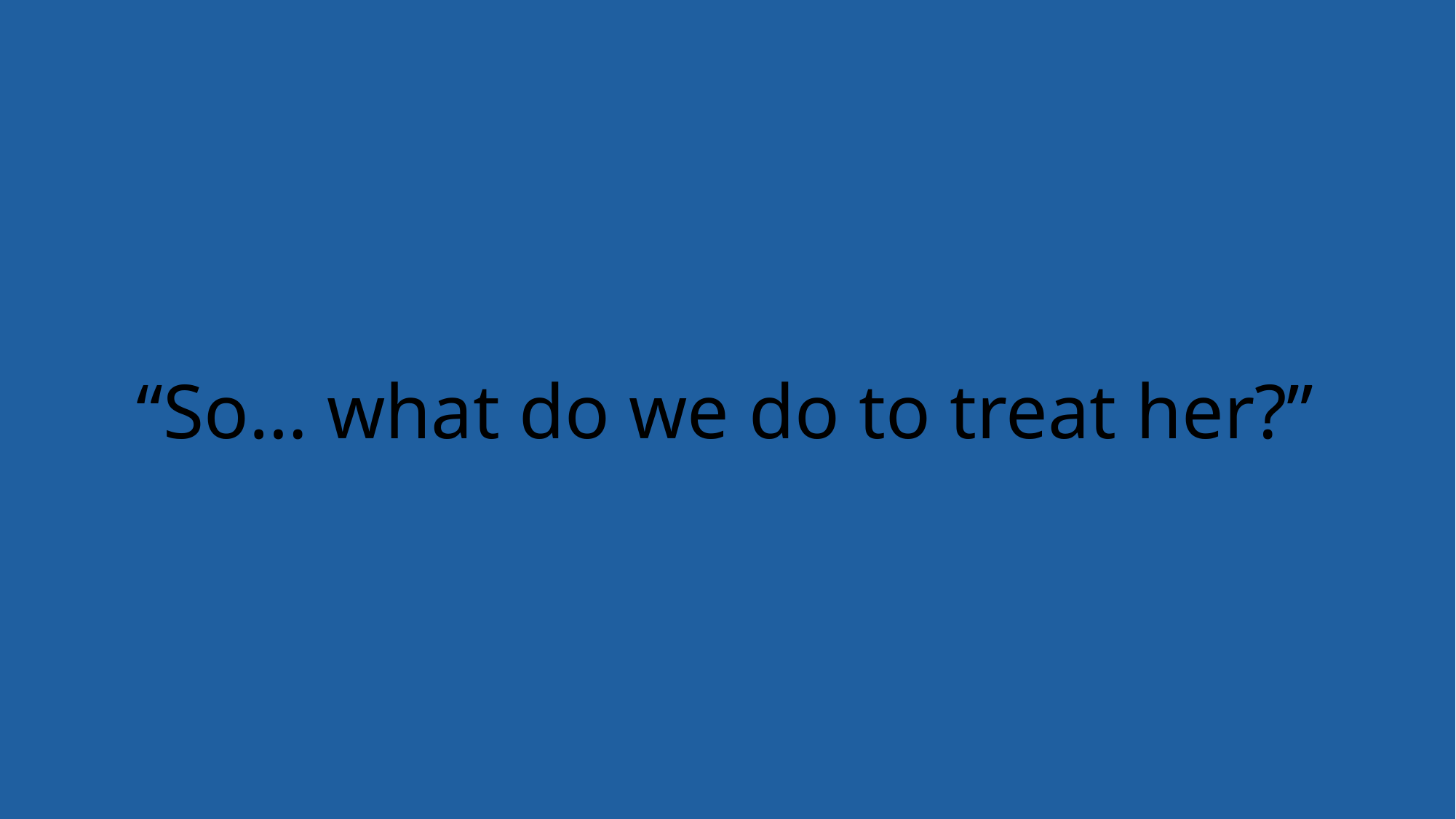

# “So… what do we do to treat her?”

## Slide 22
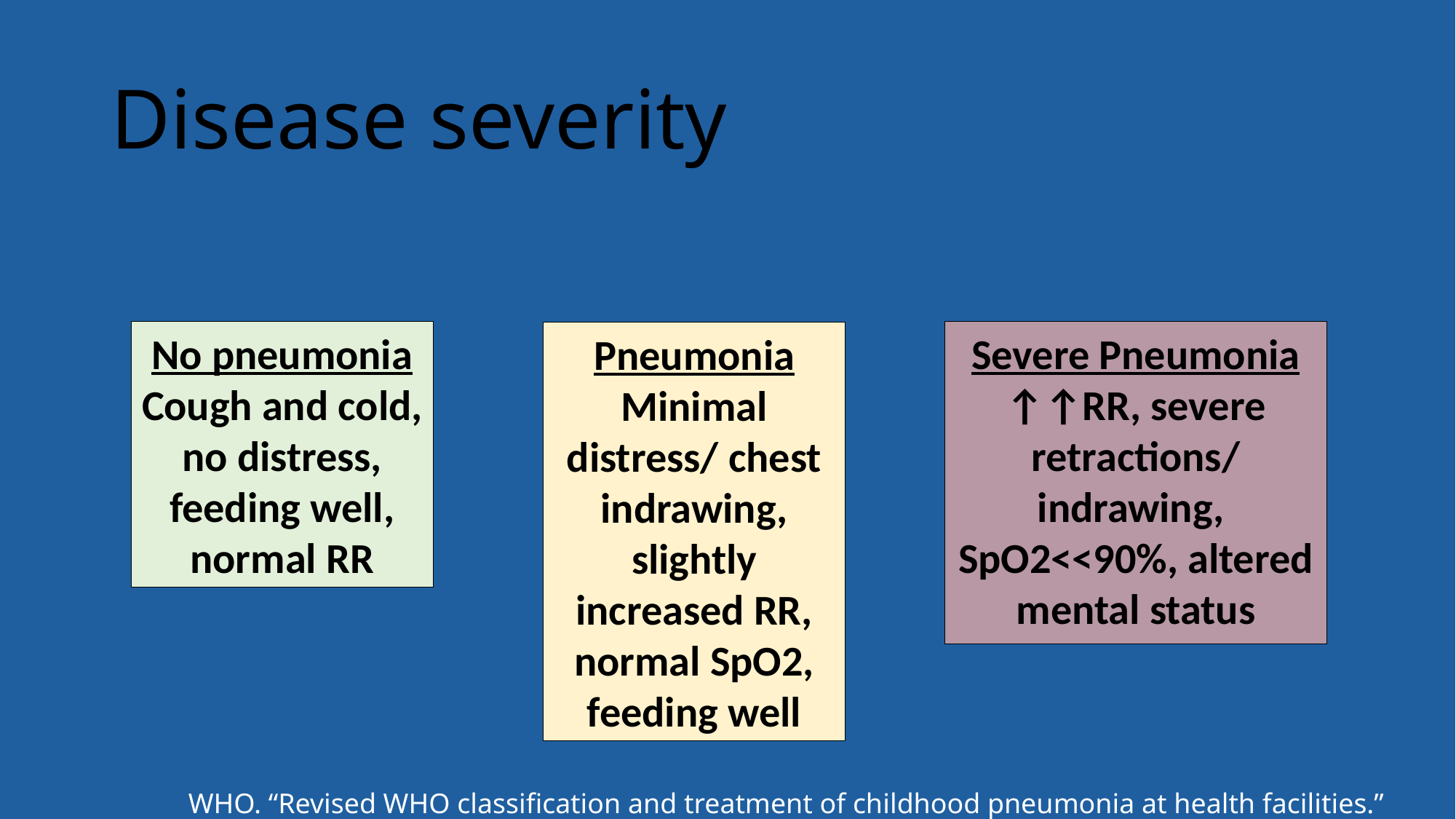

# Disease severity
No pneumonia
Cough and cold, no distress, feeding well, normal RR
Severe Pneumonia
↑↑RR, severe retractions/ indrawing, SpO2<<90%, altered mental status
Pneumonia
Minimal distress/ chest indrawing, slightly increased RR, normal SpO2, feeding well
WHO. “Revised WHO classification and treatment of childhood pneumonia at health facilities.” 2014.

## Slide 23
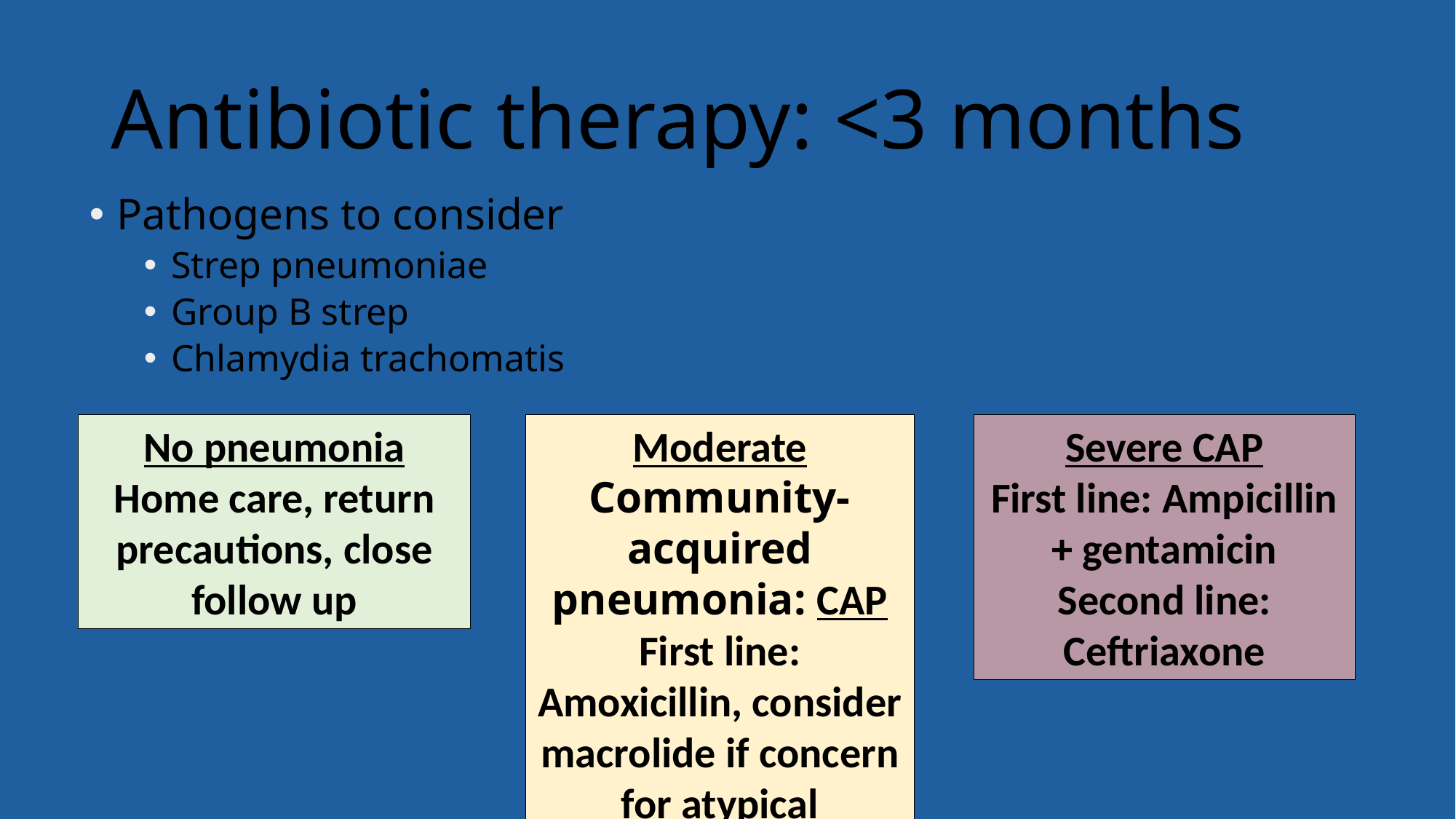

# Antibiotic therapy: <3 months
Pathogens to consider
Strep pneumoniae
Group B strep
Chlamydia trachomatis
No pneumonia
Home care, return precautions, close follow up
Moderate Community-acquired pneumonia: CAP
First line: Amoxicillin, consider macrolide if concern for atypical pathogens
Severe CAP
First line: Ampicillin + gentamicin
Second line: Ceftriaxone

## Slide 24
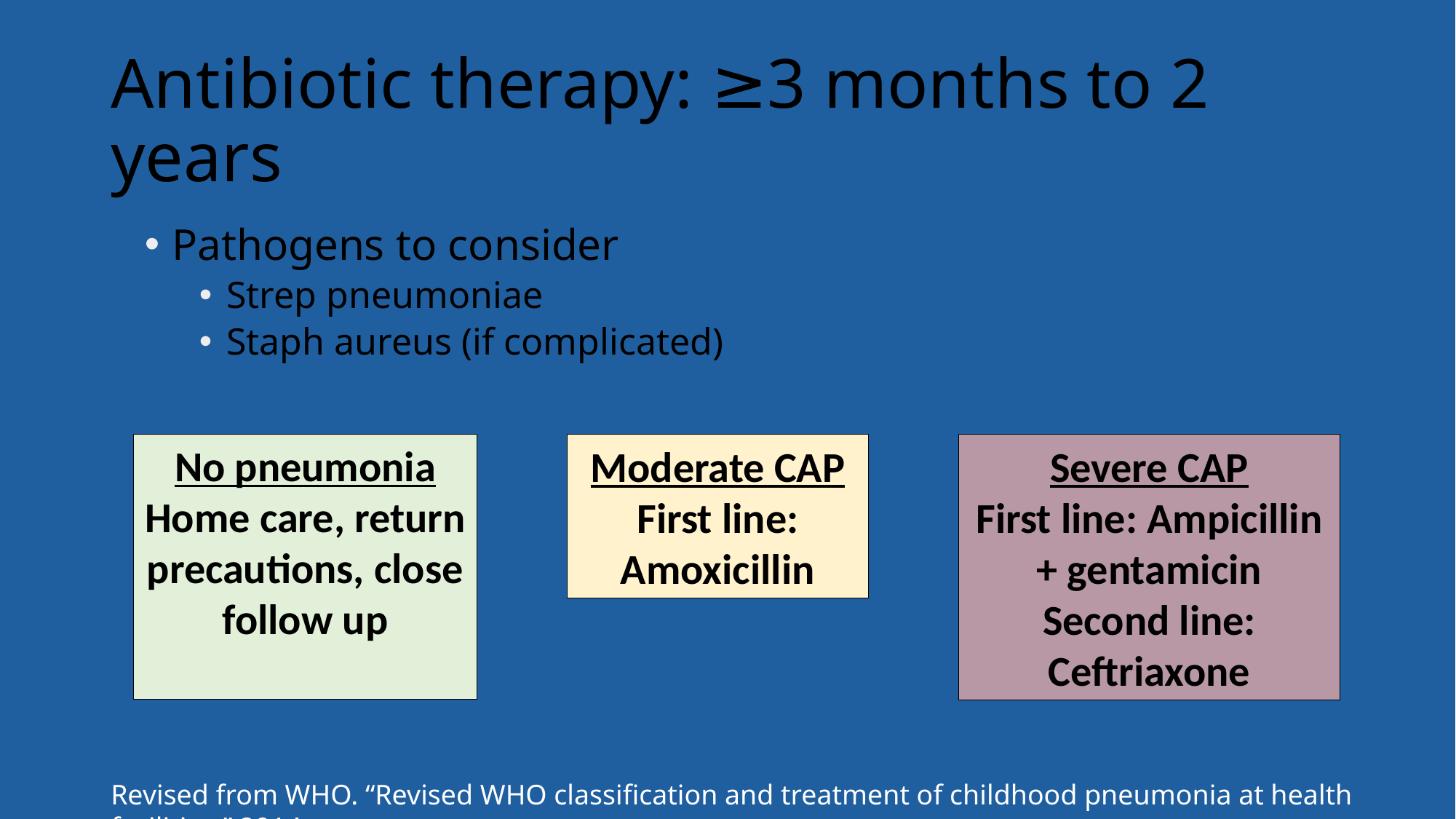

# Antibiotic therapy: ≥3 months to 2 years
Pathogens to consider
Strep pneumoniae
Staph aureus (if complicated)
No pneumonia
Home care, return precautions, close follow up
Severe CAP
First line: Ampicillin + gentamicin
Second line: Ceftriaxone
Moderate CAP
First line: Amoxicillin
Revised from WHO. “Revised WHO classification and treatment of childhood pneumonia at health facilities.” 2014

## Slide 25
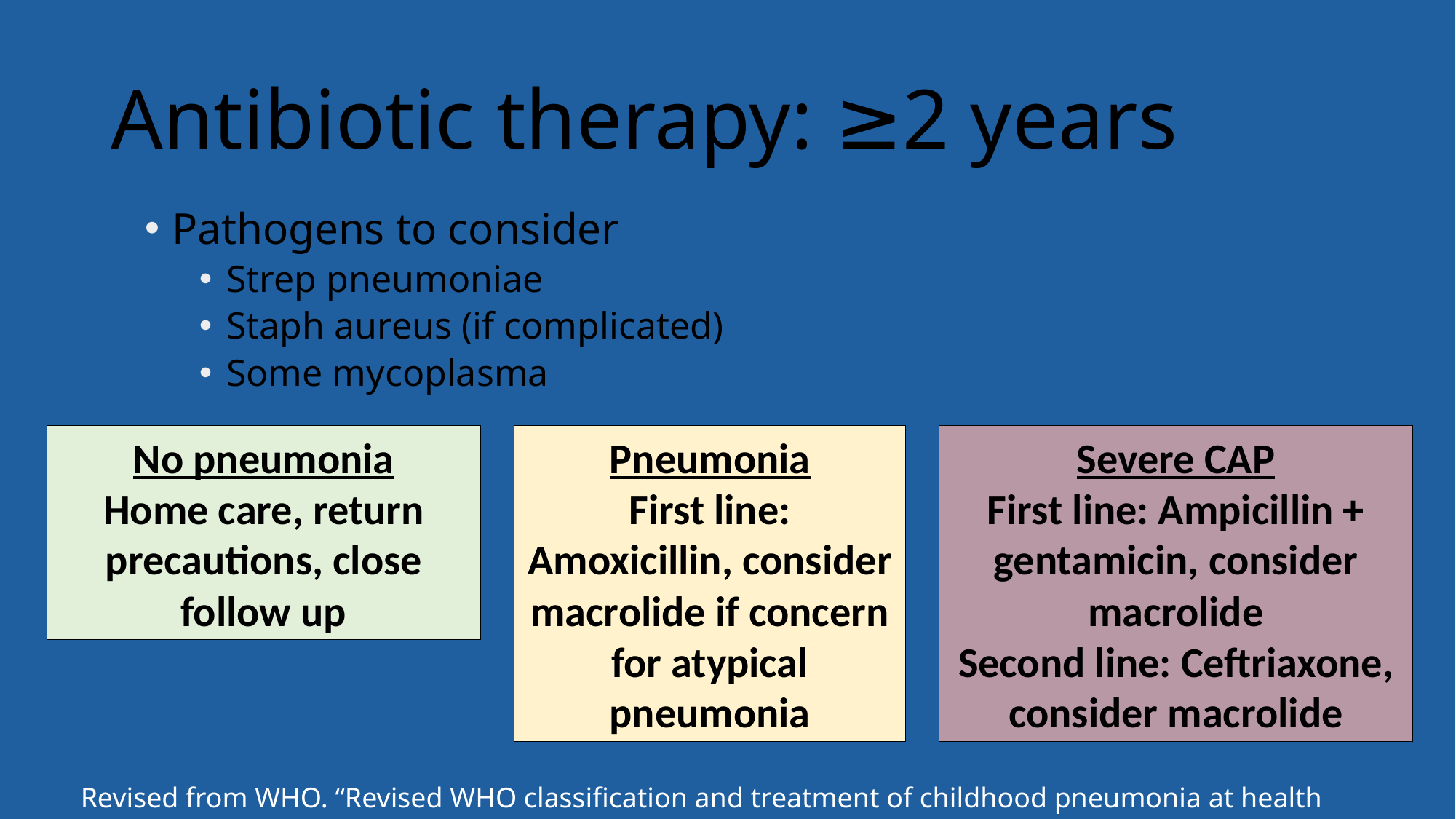

# Antibiotic therapy: ≥2 years
Pathogens to consider
Strep pneumoniae
Staph aureus (if complicated)
Some mycoplasma
No pneumonia
Home care, return precautions, close follow up
Pneumonia
First line: Amoxicillin, consider macrolide if concern for atypical pneumonia
Severe CAP
First line: Ampicillin + gentamicin, consider macrolide
Second line: Ceftriaxone, consider macrolide
Revised from WHO. “Revised WHO classification and treatment of childhood pneumonia at health facilities.” 2014

## Slide 26
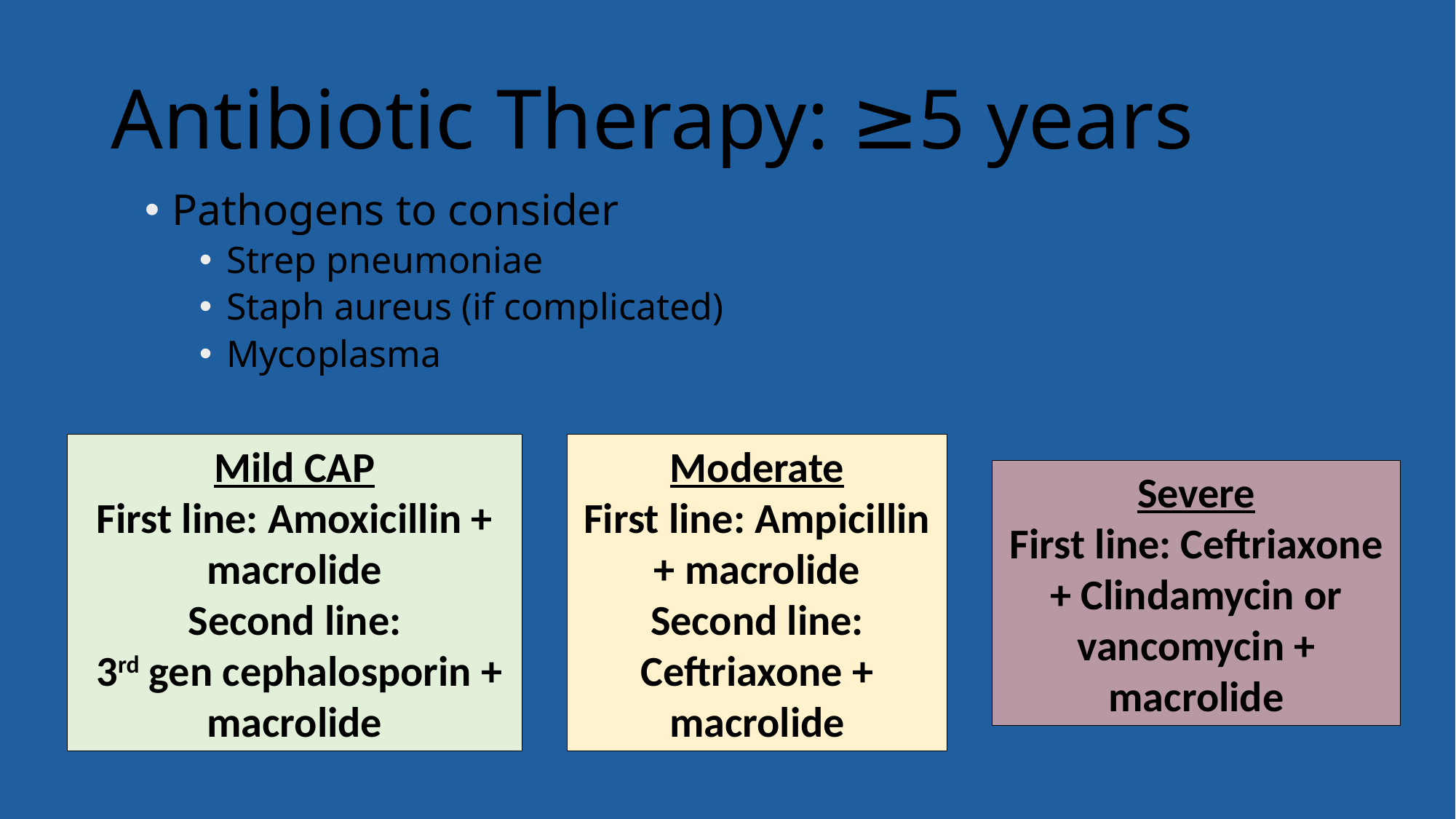

# Antibiotic Therapy: ≥5 years
Pathogens to consider
Strep pneumoniae
Staph aureus (if complicated)
Mycoplasma
Mild CAP
First line: Amoxicillin + macrolide
Second line:
 3rd gen cephalosporin + macrolide
Moderate
First line: Ampicillin + macrolide
Second line: Ceftriaxone + macrolide
Severe
First line: Ceftriaxone + Clindamycin or vancomycin + macrolide

## Slide 27
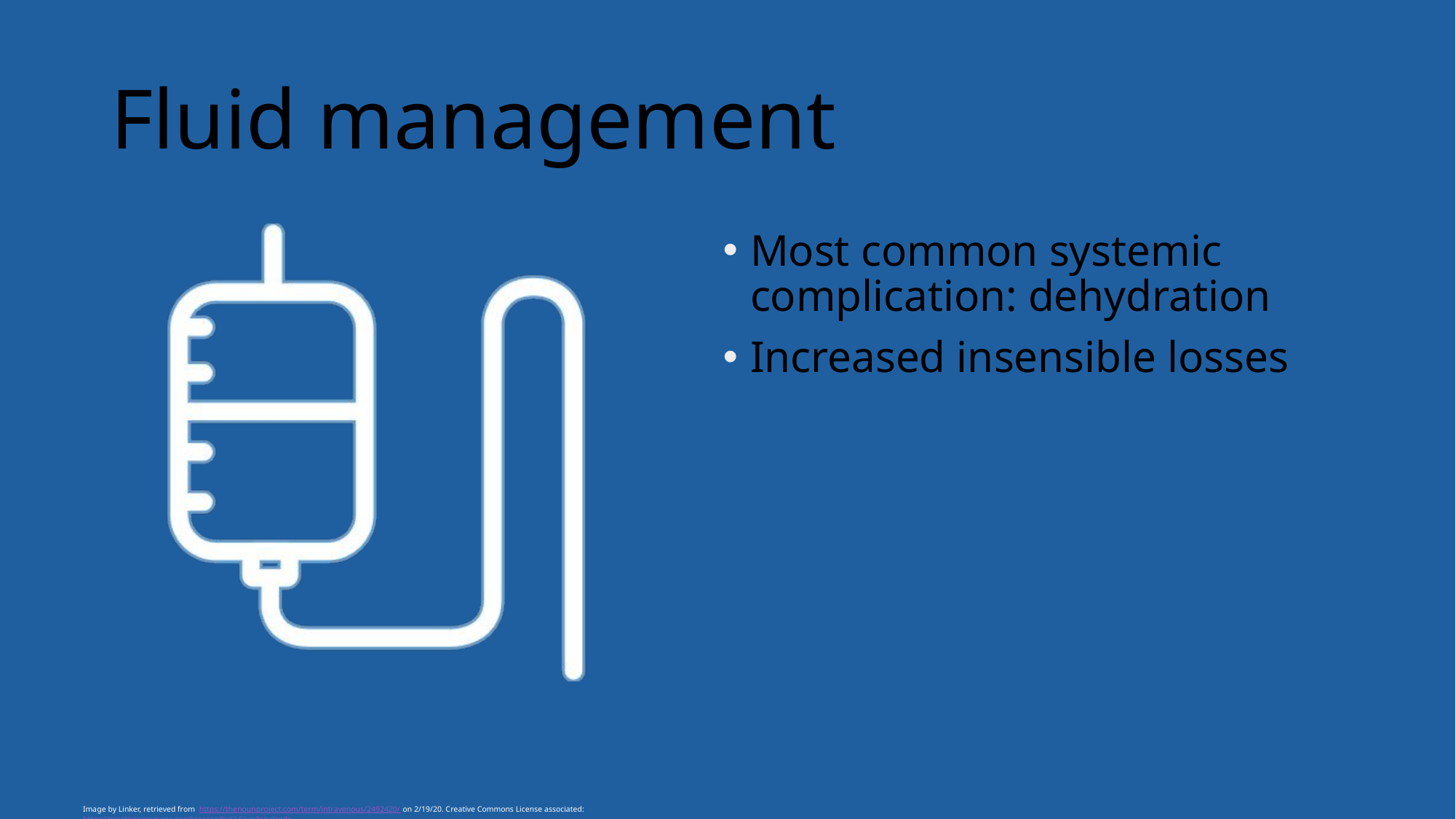

# Fluid management
Most common systemic complication: dehydration
Increased insensible losses
Image by Linker, retrieved from  https://thenounproject.com/term/intravenous/2492420/ on 2/19/20. Creative Commons License associated: https://creativecommons.org/licenses/by/3.0/us/legalcode.

## Slide 28
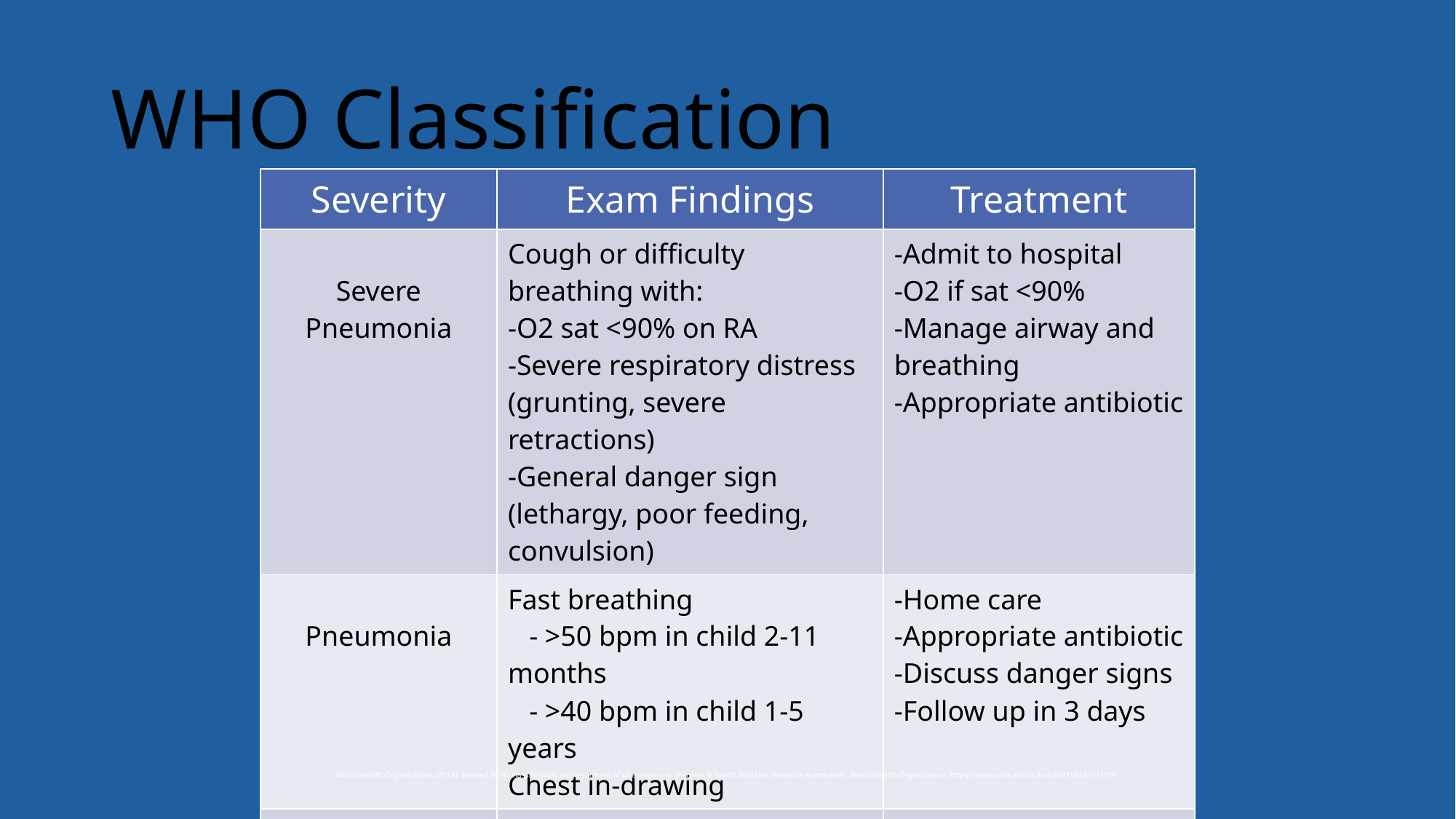

# WHO Classification
| Severity | Exam Findings | Treatment |
| --- | --- | --- |
| Severe Pneumonia | Cough or difficulty breathing with: -O2 sat <90% on RA -Severe respiratory distress (grunting, severe retractions) -General danger sign (lethargy, poor feeding, convulsion) | -Admit to hospital -O2 if sat <90% -Manage airway and breathing -Appropriate antibiotic |
| Pneumonia | Fast breathing - >50 bpm in child 2-11 months - >40 bpm in child 1-5 years Chest in-drawing | -Home care -Appropriate antibiotic -Discuss danger signs -Follow up in 3 days |
| No pneumonia, cough or cold | None of above signs | -Home care -Discuss danger signs -Follow up 5 days if not improving |
World Health Organization. (‎2014)‎. Revised WHO classification and treatment of pneumonia in children at health facilities: evidence summaries. World Health Organization. https://apps.who.int/iris/handle/10665/137319

## Slide 29
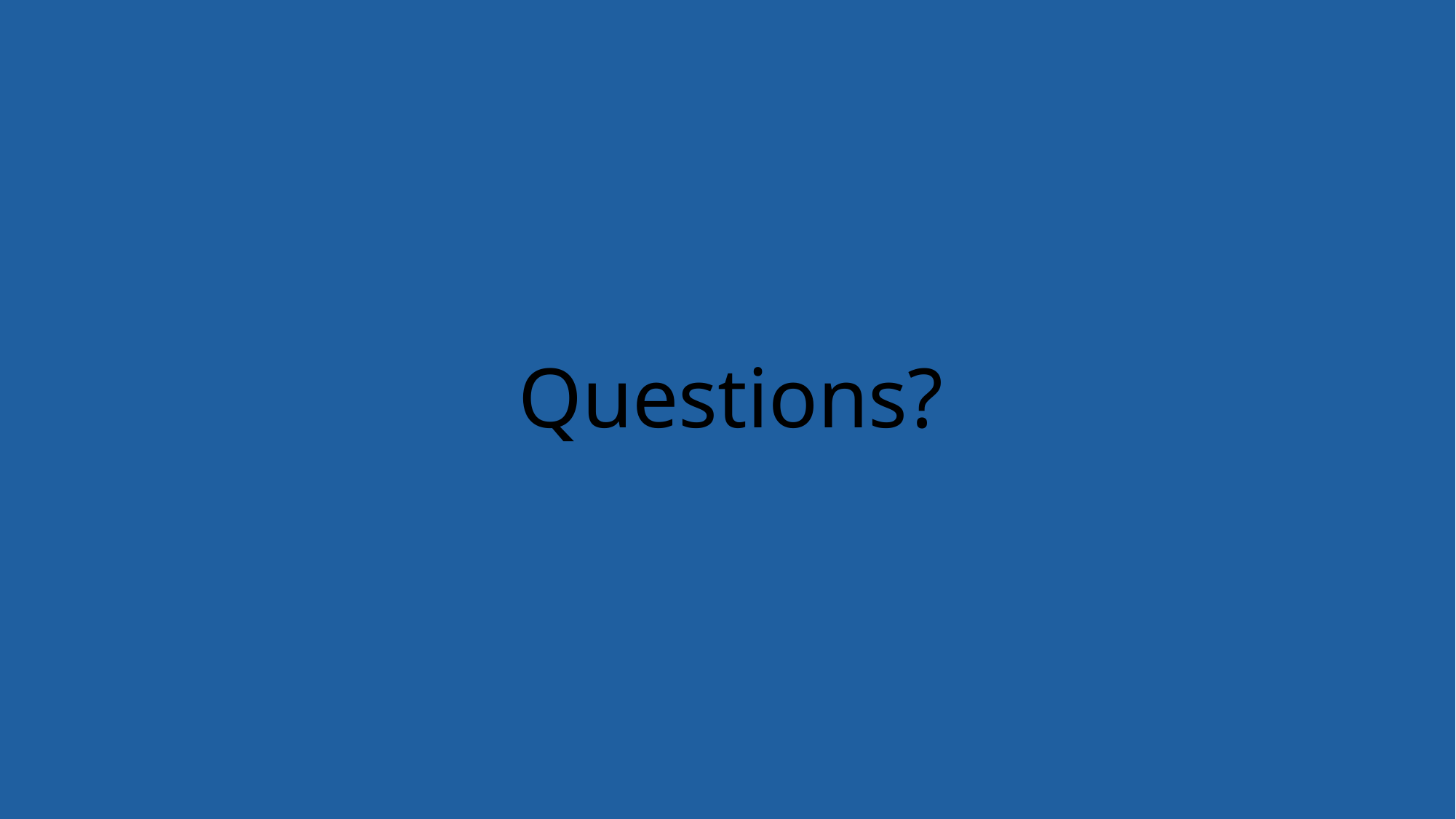

# Questions?
