# Supplementary material for: A Pediatric Emergency Medicine Refresher Course for Generalist Healthcare Providers in Belize: Respiratory Emergencies
Source: J Educ Teach Emerg Med. 2021 Apr 19;6(2):C73–C188. doi: 10.21980/J84063 (PMC10332788; doi:10.21980/J84063)
Supplement: Supplementary file 3 — Please see associated PowerPoint file [file jetem-6-2-c73-AppendixI.pptx]

## Slide 1
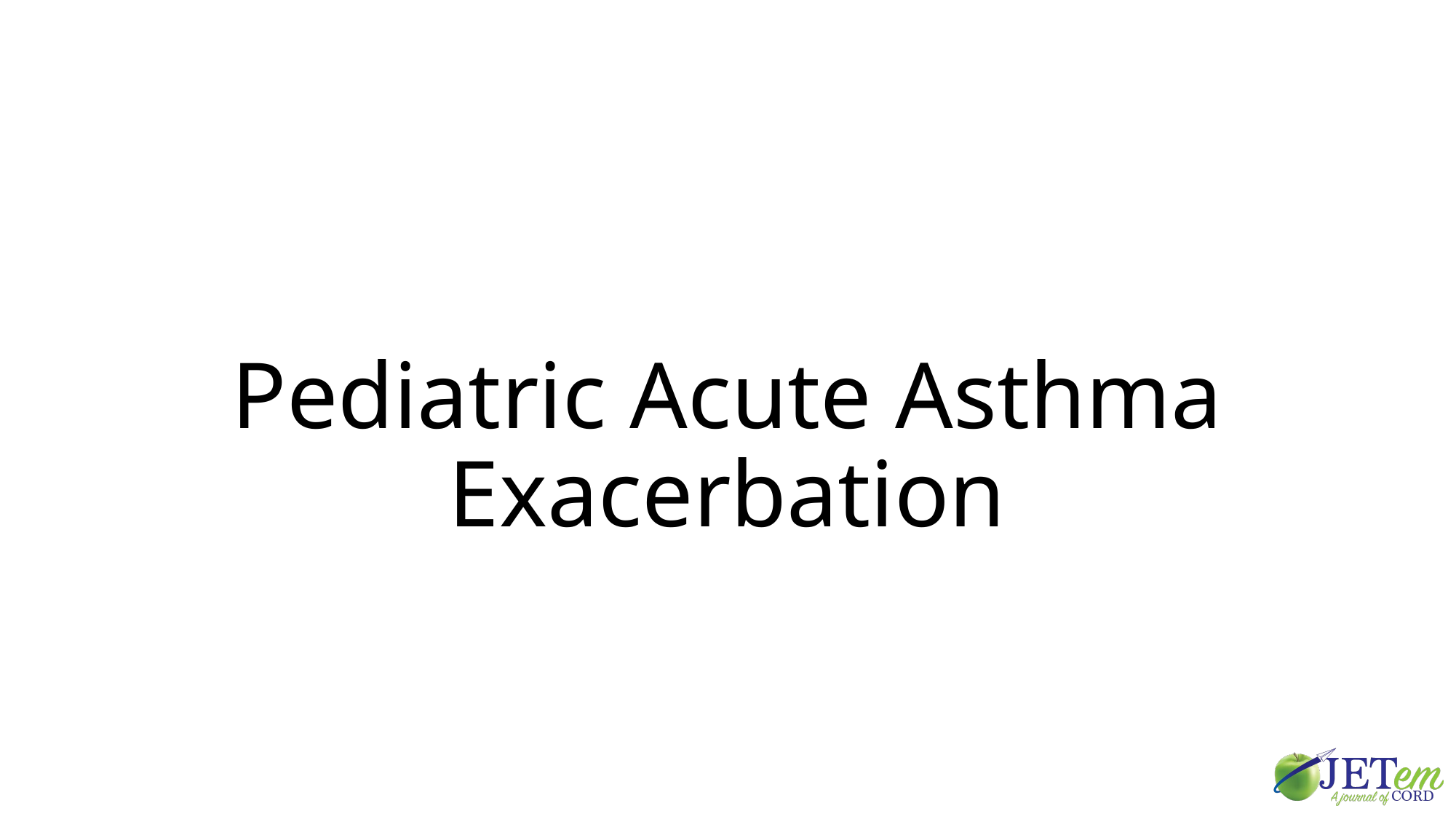

# Pediatric Acute Asthma Exacerbation

## Slide 2
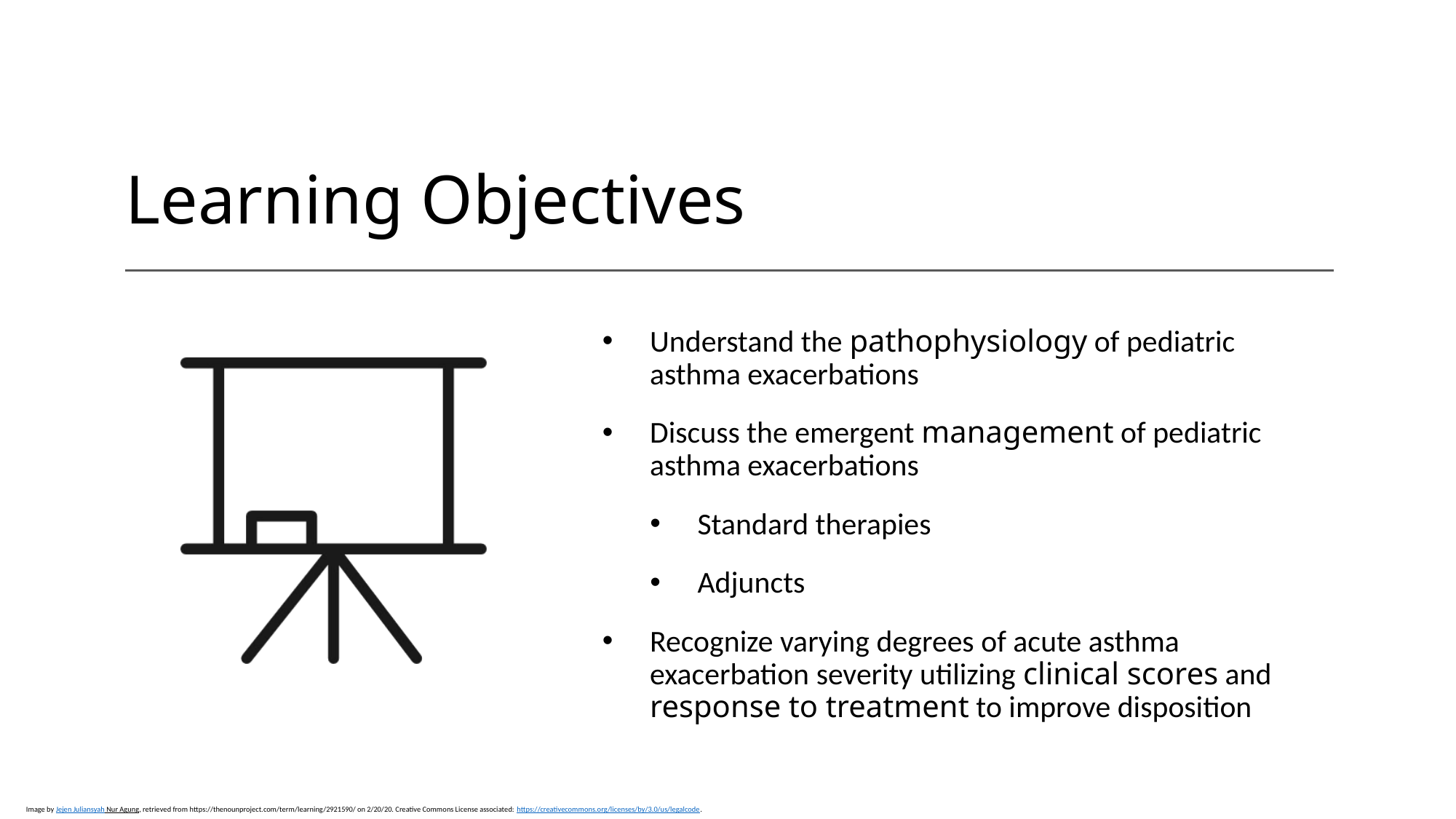

# Learning Objectives
Understand the pathophysiology of pediatric asthma exacerbations
Discuss the emergent management of pediatric asthma exacerbations
Standard therapies
Adjuncts
Recognize varying degrees of acute asthma exacerbation severity utilizing clinical scores and response to treatment to improve disposition
Image by Jejen Juliansyah Nur Agung, retrieved from https://thenounproject.com/term/learning/2921590/ on 2/20/20. Creative Commons License associated: https://creativecommons.org/licenses/by/3.0/us/legalcode.

## Slide 3
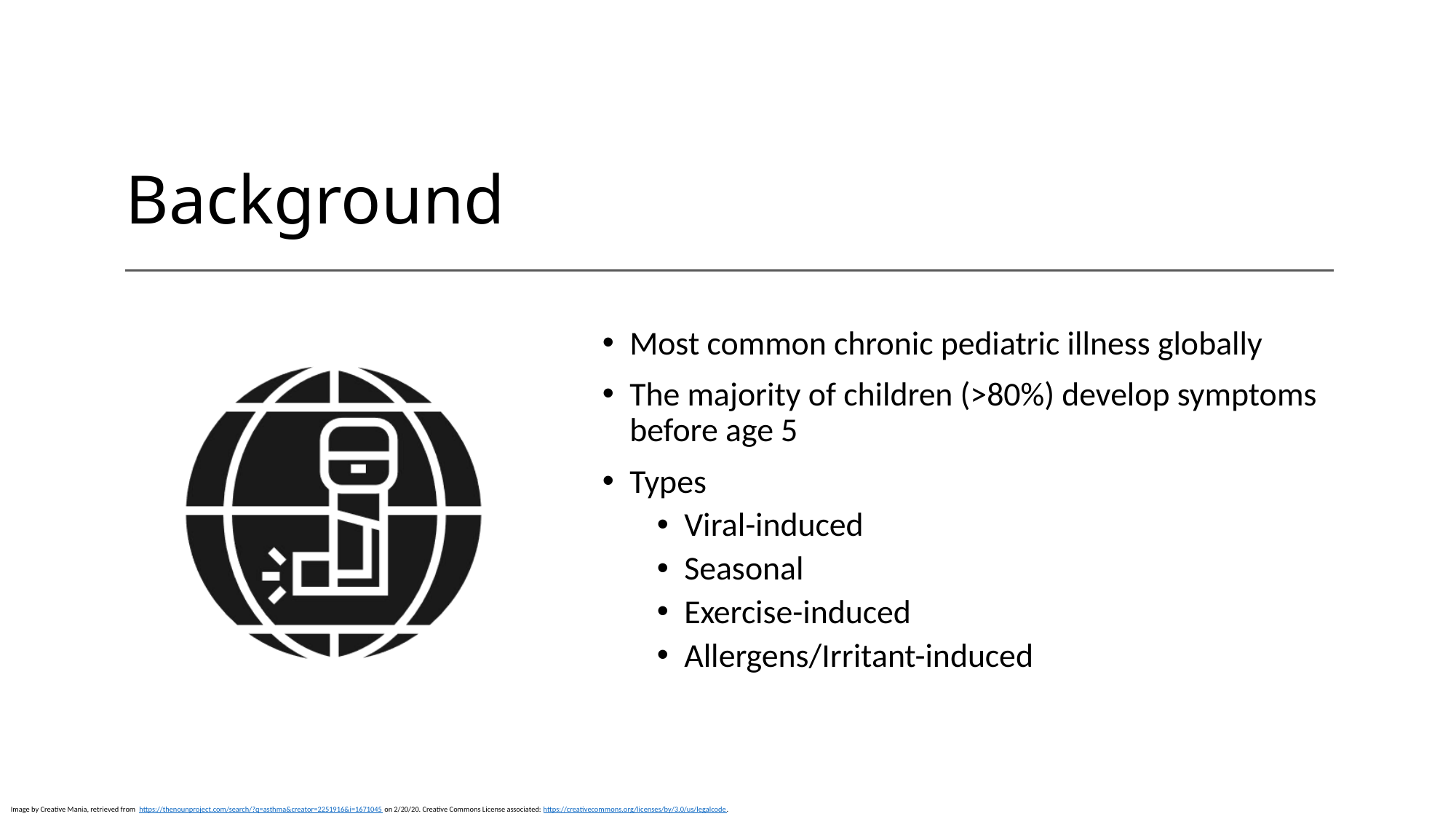

# Background
Most common chronic pediatric illness globally
The majority of children (>80%) develop symptoms before age 5
Types
Viral-induced
Seasonal
Exercise-induced
Allergens/Irritant-induced
Image by Creative Mania, retrieved from  https://thenounproject.com/search/?q=asthma&creator=2251916&i=1671045 on 2/20/20. Creative Commons License associated: https://creativecommons.org/licenses/by/3.0/us/legalcode.

## Slide 4
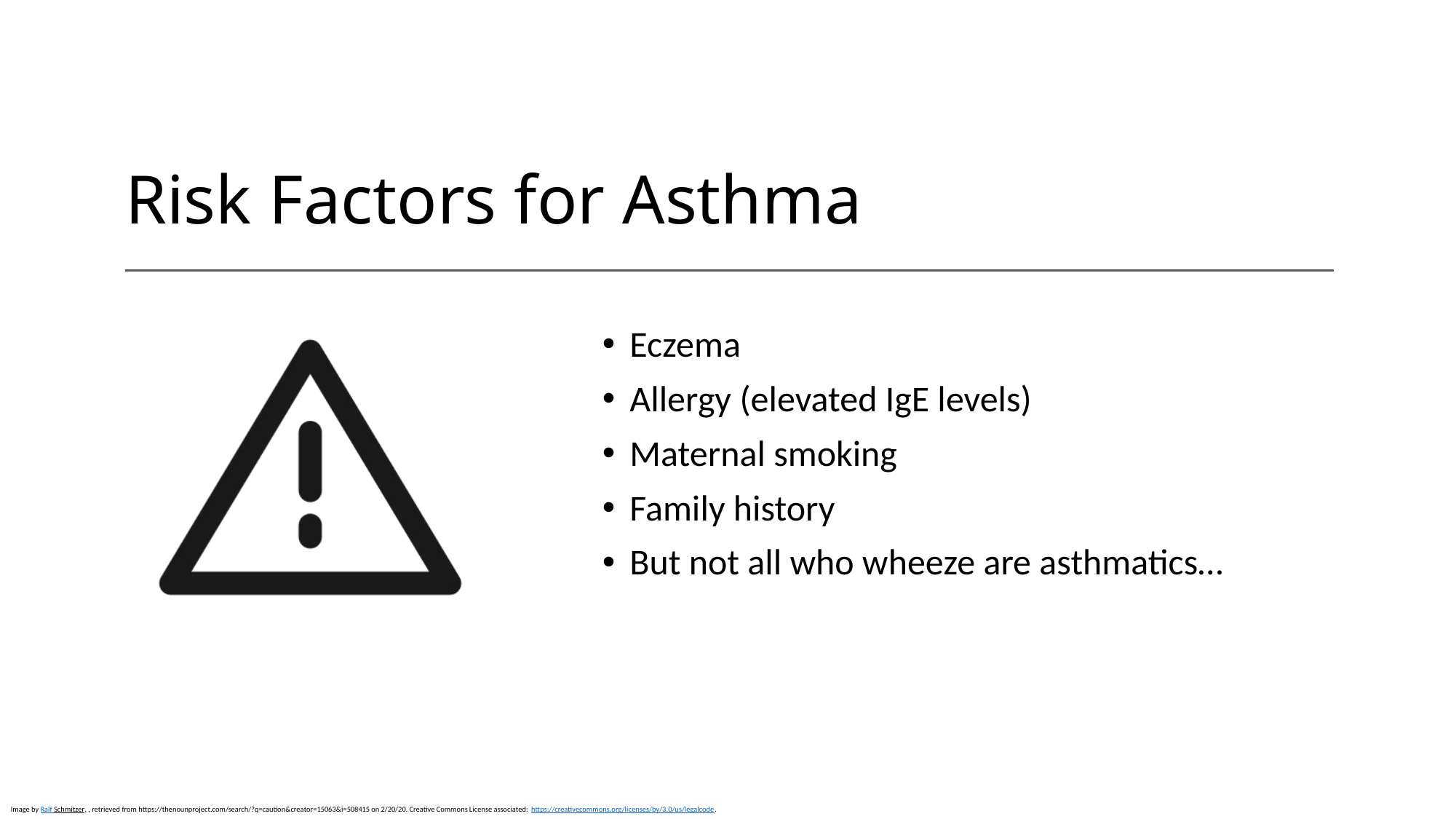

# Risk Factors for Asthma
Eczema
Allergy (elevated IgE levels)
Maternal smoking
Family history
But not all who wheeze are asthmatics…
Image by Ralf Schmitzer, , retrieved from https://thenounproject.com/search/?q=caution&creator=15063&i=508415 on 2/20/20. Creative Commons License associated: https://creativecommons.org/licenses/by/3.0/us/legalcode.

## Slide 5
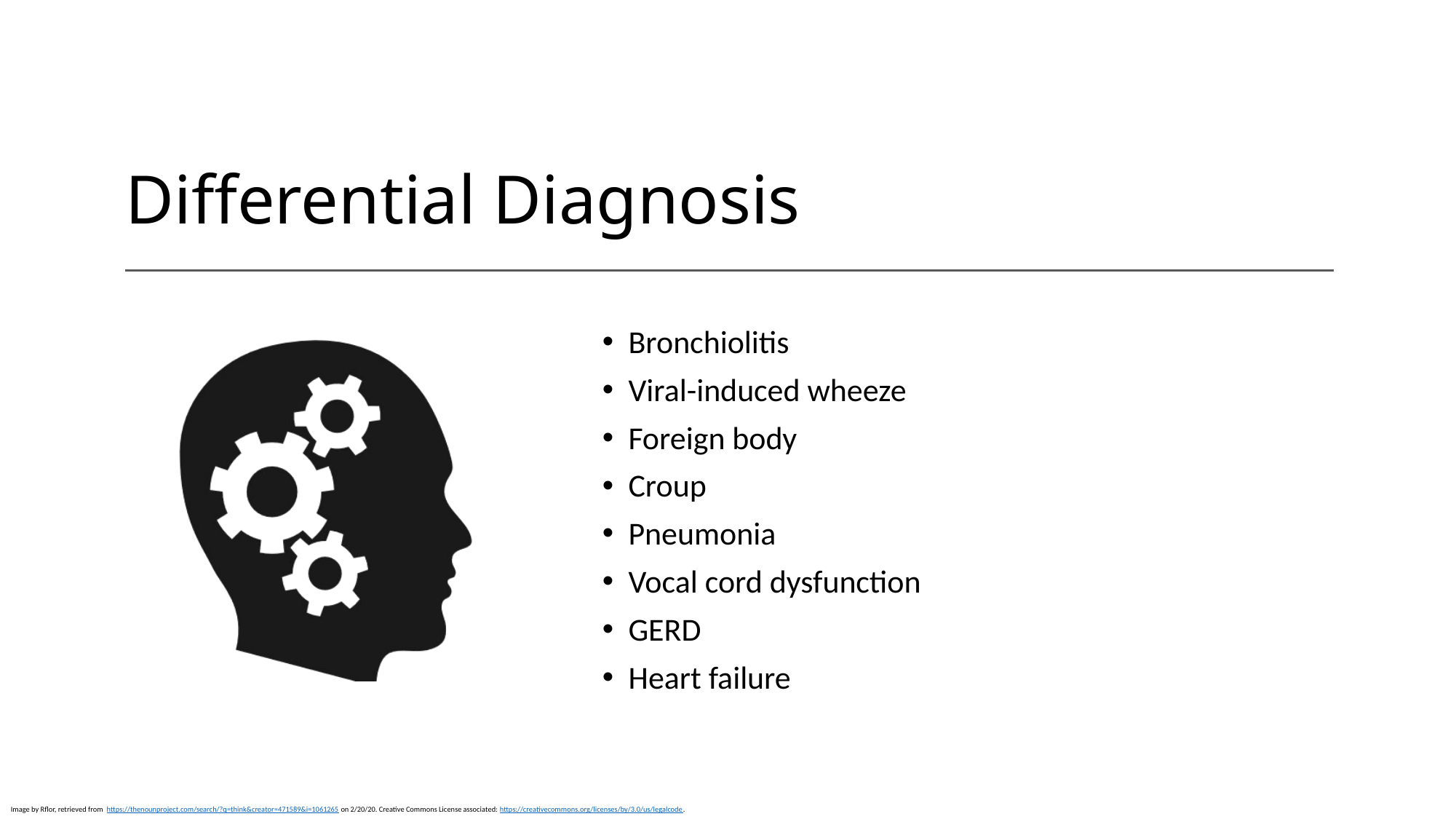

# Differential Diagnosis
Bronchiolitis
Viral-induced wheeze
Foreign body
Croup
Pneumonia
Vocal cord dysfunction
GERD
Heart failure
Image by Rflor, retrieved from  https://thenounproject.com/search/?q=think&creator=471589&i=1061265 on 2/20/20. Creative Commons License associated: https://creativecommons.org/licenses/by/3.0/us/legalcode.

## Slide 6
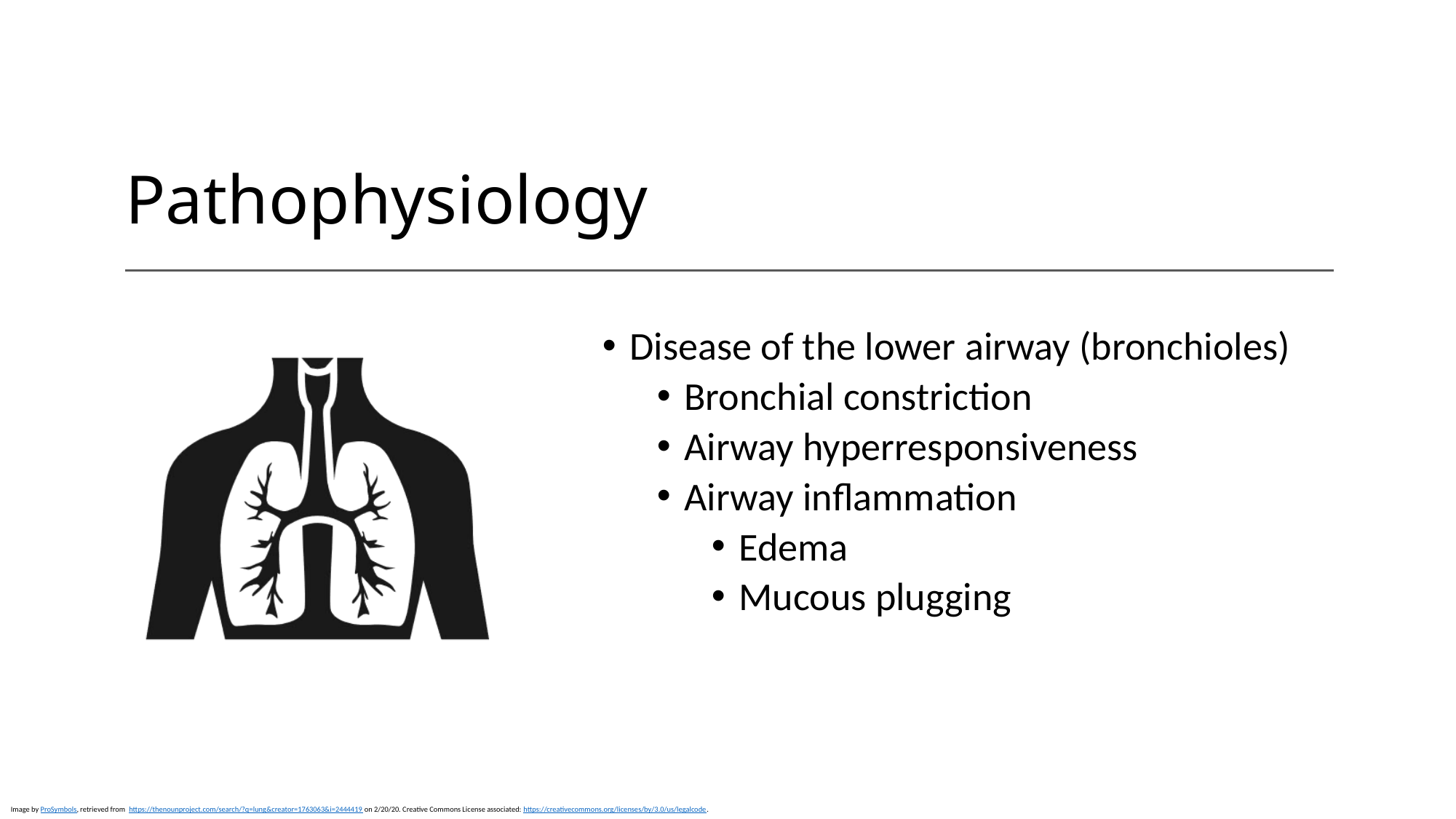

# Pathophysiology
Disease of the lower airway (bronchioles)
Bronchial constriction
Airway hyperresponsiveness
Airway inflammation
Edema
Mucous plugging
Image by ProSymbols, retrieved from  https://thenounproject.com/search/?q=lung&creator=1763063&i=2444419 on 2/20/20. Creative Commons License associated: https://creativecommons.org/licenses/by/3.0/us/legalcode.

## Slide 7
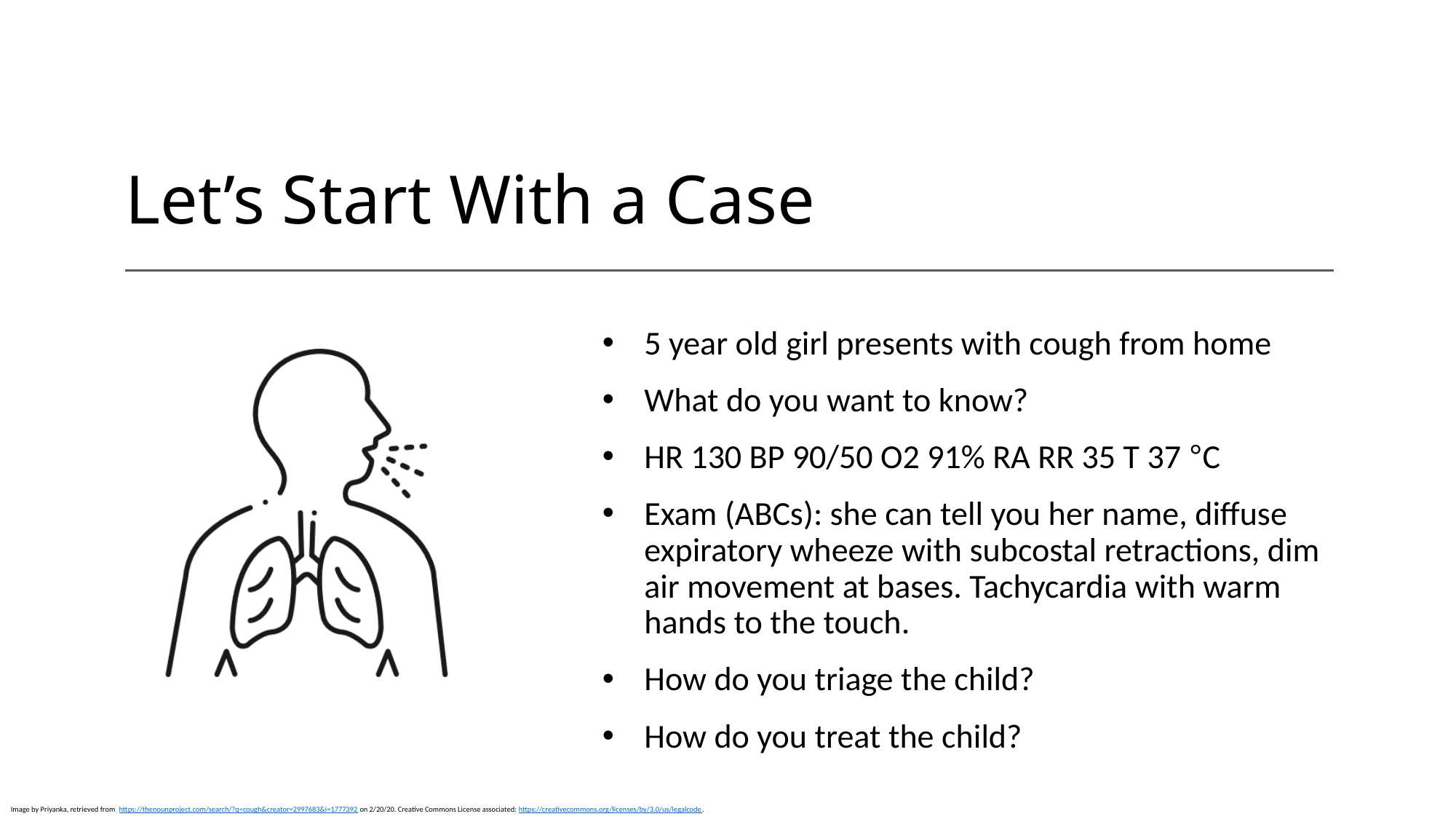

# Let’s Start With a Case
5 year old girl presents with cough from home
What do you want to know?
HR 130 BP 90/50 O2 91% RA RR 35 T 37 °C
Exam (ABCs): she can tell you her name, diffuse expiratory wheeze with subcostal retractions, dim air movement at bases. Tachycardia with warm hands to the touch.
How do you triage the child?
How do you treat the child?
Image by Priyanka, retrieved from  https://thenounproject.com/search/?q=cough&creator=2997683&i=1777392 on 2/20/20. Creative Commons License associated: https://creativecommons.org/licenses/by/3.0/us/legalcode.

## Slide 8
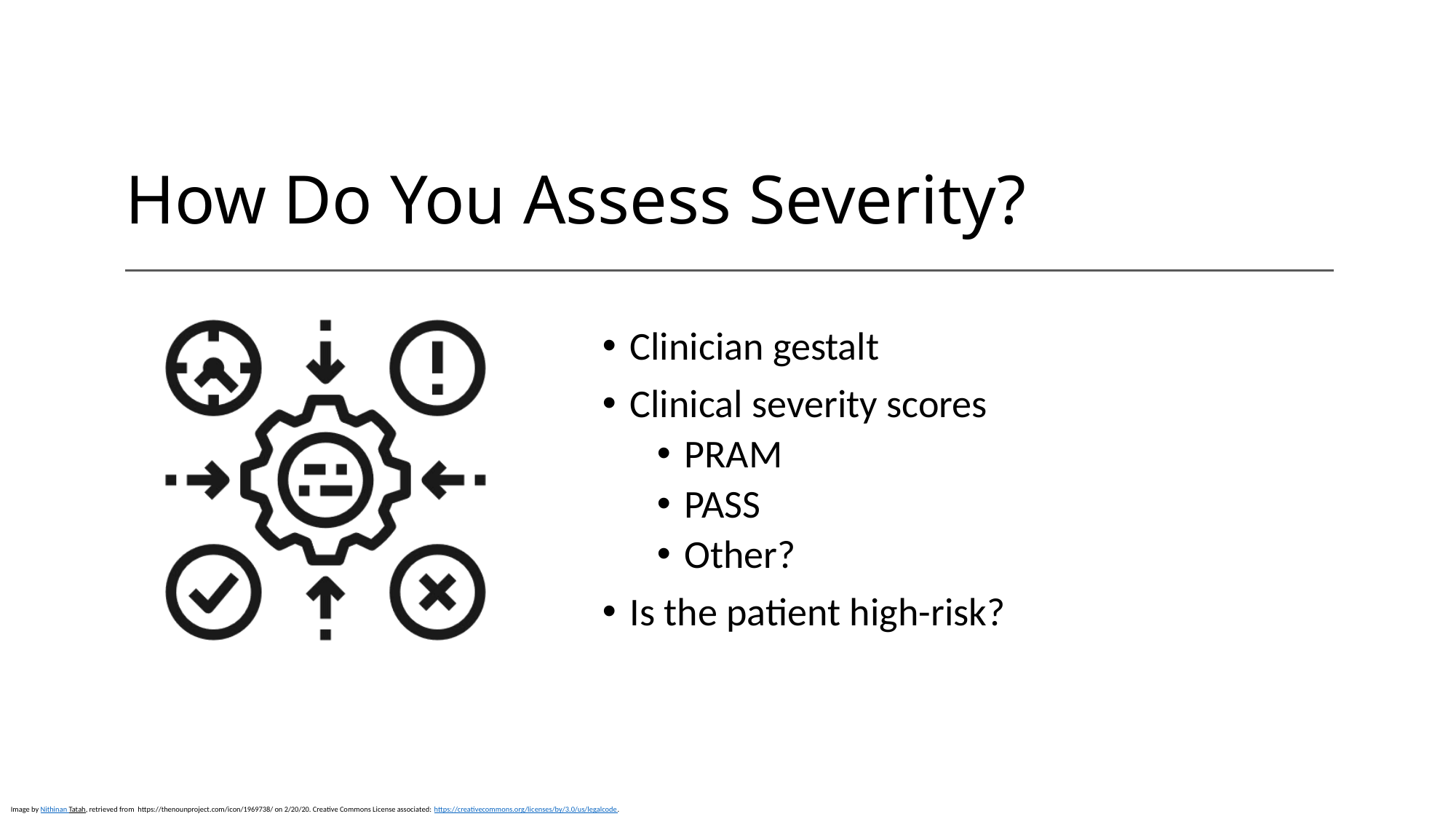

# How Do You Assess Severity?
Clinician gestalt
Clinical severity scores
PRAM
PASS
Other?
Is the patient high-risk?
Image by Nithinan Tatah, retrieved from  https://thenounproject.com/icon/1969738/ on 2/20/20. Creative Commons License associated: https://creativecommons.org/licenses/by/3.0/us/legalcode.

## Slide 9
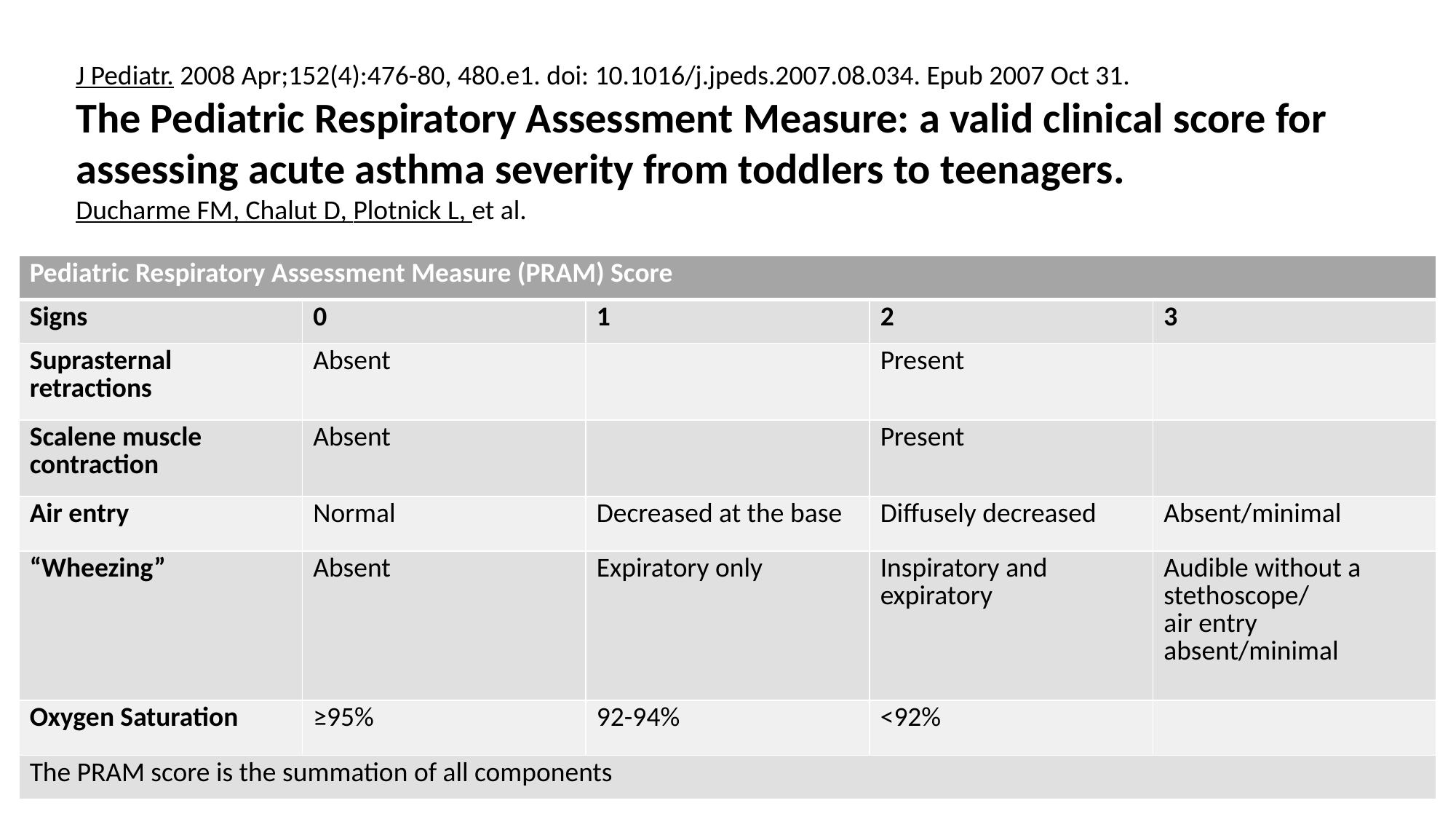

J Pediatr. 2008 Apr;152(4):476-80, 480.e1. doi: 10.1016/j.jpeds.2007.08.034. Epub 2007 Oct 31.
The Pediatric Respiratory Assessment Measure: a valid clinical score for assessing acute asthma severity from toddlers to teenagers.
Ducharme FM, Chalut D, Plotnick L, et al.
# PRAM Score
| Pediatric Respiratory Assessment Measure (PRAM) Score | | | | |
| --- | --- | --- | --- | --- |
| Signs | 0 | 1 | 2 | 3 |
| Suprasternal retractions | Absent | | Present | |
| Scalene muscle contraction | Absent | | Present | |
| Air entry | Normal | Decreased at the base | Diffusely decreased | Absent/minimal |
| “Wheezing” | Absent | Expiratory only | Inspiratory and expiratory | Audible without a stethoscope/ air entry absent/minimal |
| Oxygen Saturation | ≥95% | 92-94% | <92% | |
| The PRAM score is the summation of all components | | | | |
Initially developed for preschoolers (<5 years old) in 2000
Since then, validated in all pediatric ages
Utilizes 5-point system with scale of 0-3
Air Entry
Subcostal retractions
Scalene contractions
Wheeze
Oxygen Saturation
Add all the individual components for total score (min 0, max 15)

## Slide 10
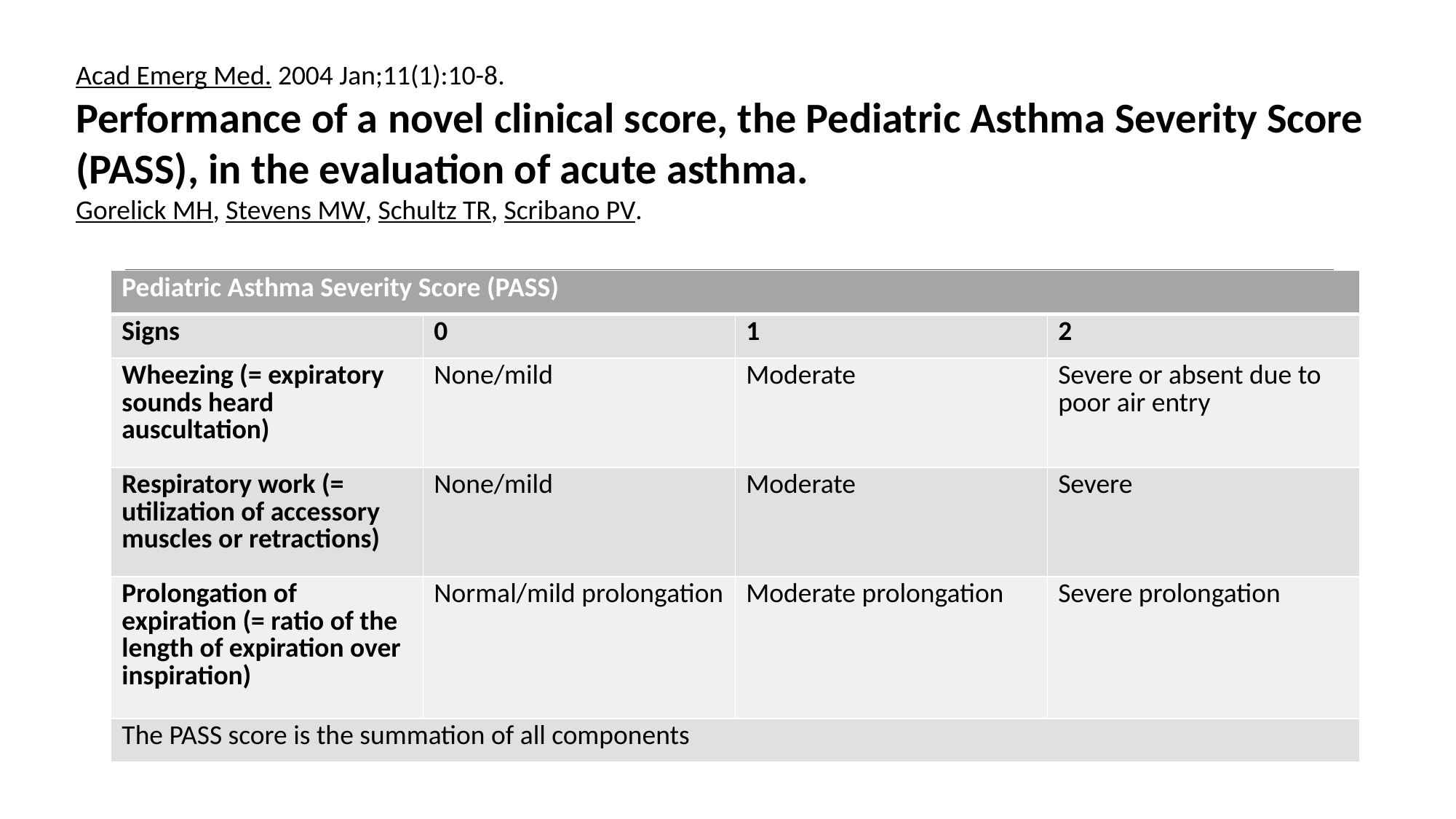

Acad Emerg Med. 2004 Jan;11(1):10-8.
Performance of a novel clinical score, the Pediatric Asthma Severity Score (PASS), in the evaluation of acute asthma.
Gorelick MH, Stevens MW, Schultz TR, Scribano PV.
# PASS Score
| Pediatric Asthma Severity Score (PASS) | | | |
| --- | --- | --- | --- |
| Signs | 0 | 1 | 2 |
| Wheezing (= expiratory sounds heard auscultation) | None/mild | Moderate | Severe or absent due to poor air entry |
| Respiratory work (= utilization of accessory muscles or retractions) | None/mild | Moderate | Severe |
| Prolongation of expiration (= ratio of the length of expiration over inspiration) | Normal/mild prolongation | Moderate prolongation | Severe prolongation |
| The PASS score is the summation of all components | | | |
Developed for all pediatric ages in 2008 as derivation of the pulmonary index (PI)
Utilizes 3-point system with scale of 0-2

## Slide 11
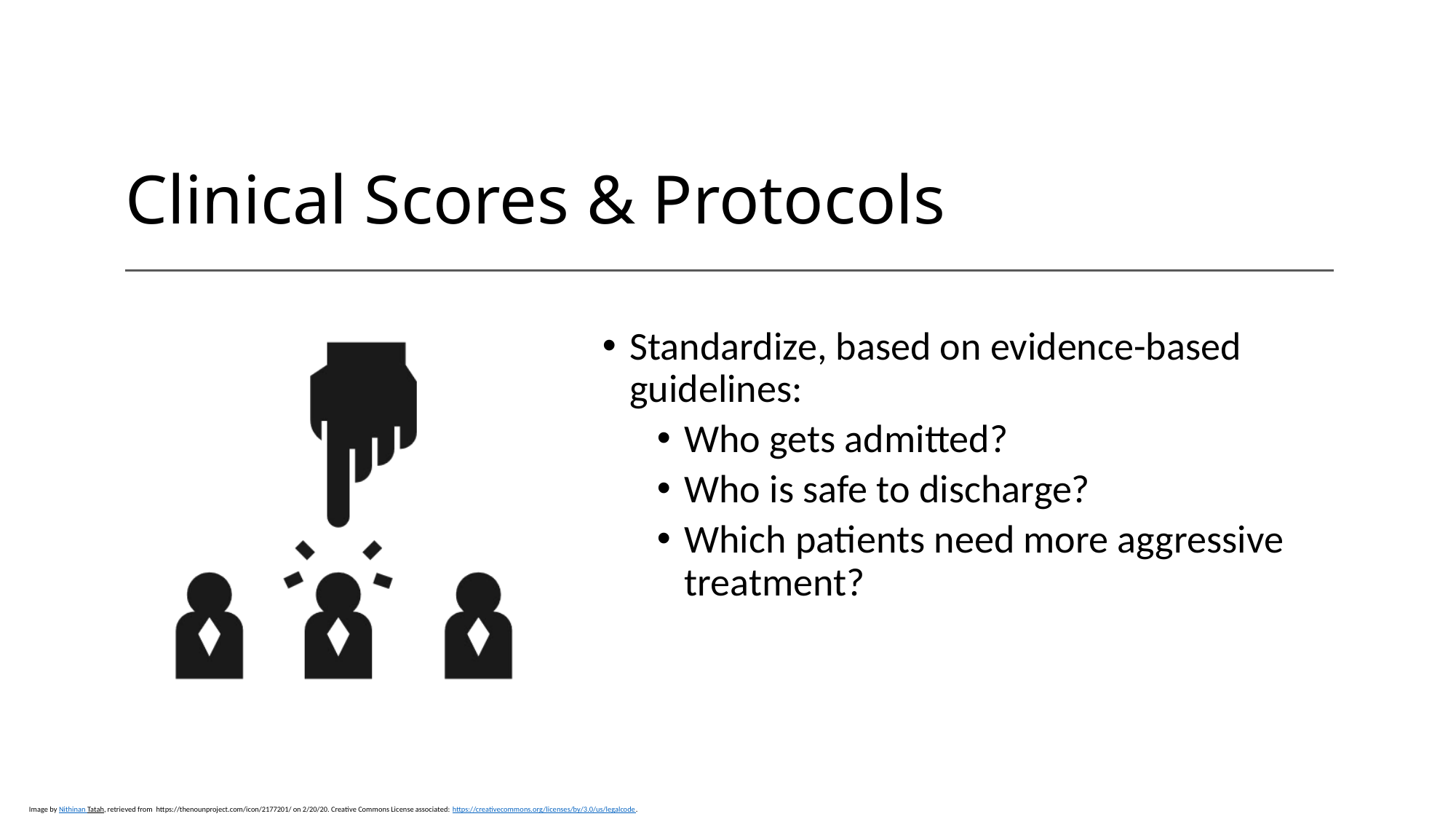

# Clinical Scores & Protocols
Standardize, based on evidence-based guidelines:
Who gets admitted?
Who is safe to discharge?
Which patients need more aggressive treatment?
Image by Nithinan Tatah, retrieved from  https://thenounproject.com/icon/2177201/ on 2/20/20. Creative Commons License associated: https://creativecommons.org/licenses/by/3.0/us/legalcode.

## Slide 12
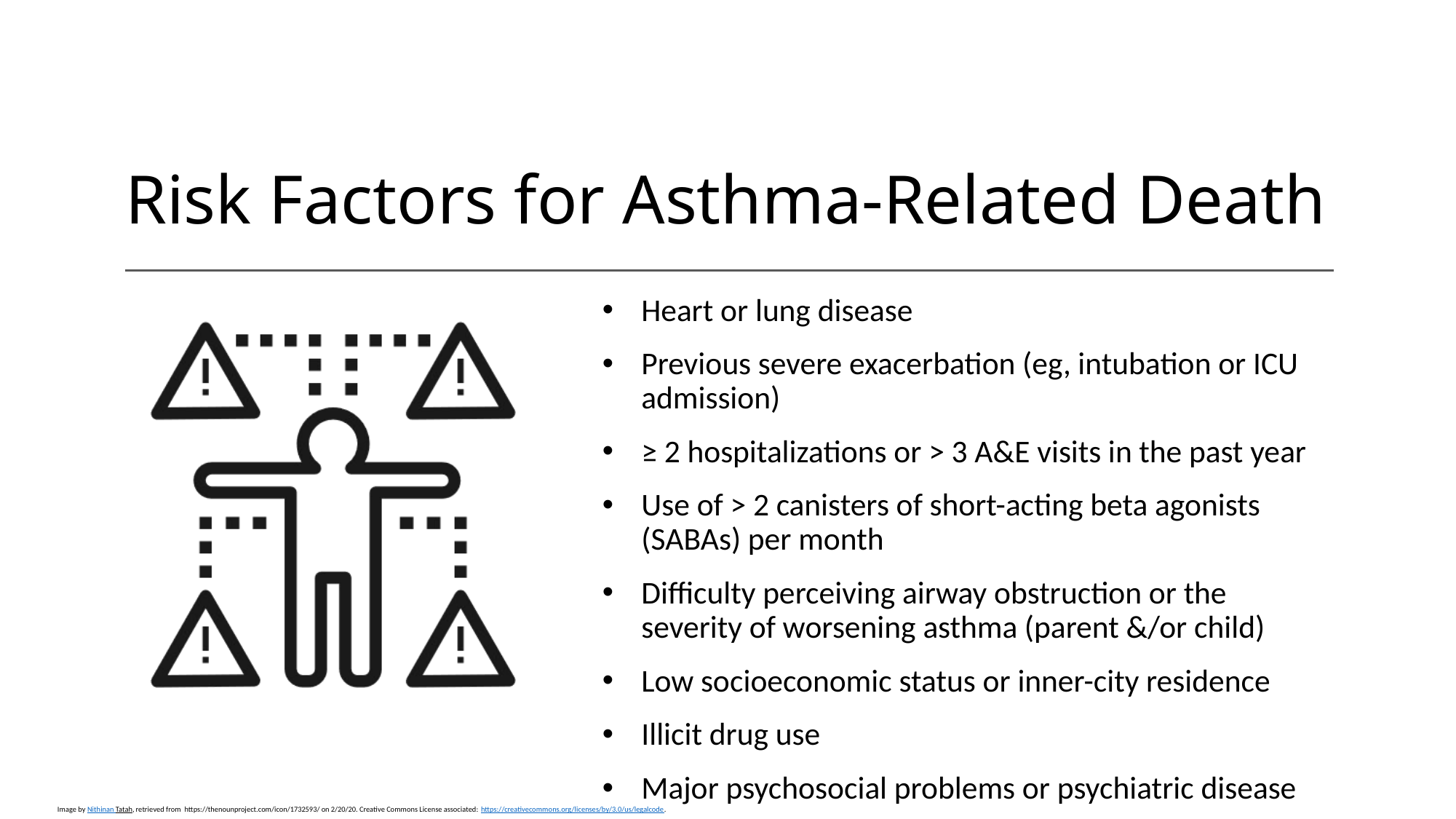

# Risk Factors for Asthma-Related Death
Heart or lung disease
Previous severe exacerbation (eg, intubation or ICU admission)
≥ 2 hospitalizations or > 3 A&E visits in the past year
Use of > 2 canisters of short-acting beta agonists (SABAs) per month
Difficulty perceiving airway obstruction or the severity of worsening asthma (parent &/or child)
Low socioeconomic status or inner-city residence
Illicit drug use
Major psychosocial problems or psychiatric disease
Image by Nithinan Tatah, retrieved from  https://thenounproject.com/icon/1732593/ on 2/20/20. Creative Commons License associated: https://creativecommons.org/licenses/by/3.0/us/legalcode.

## Slide 13
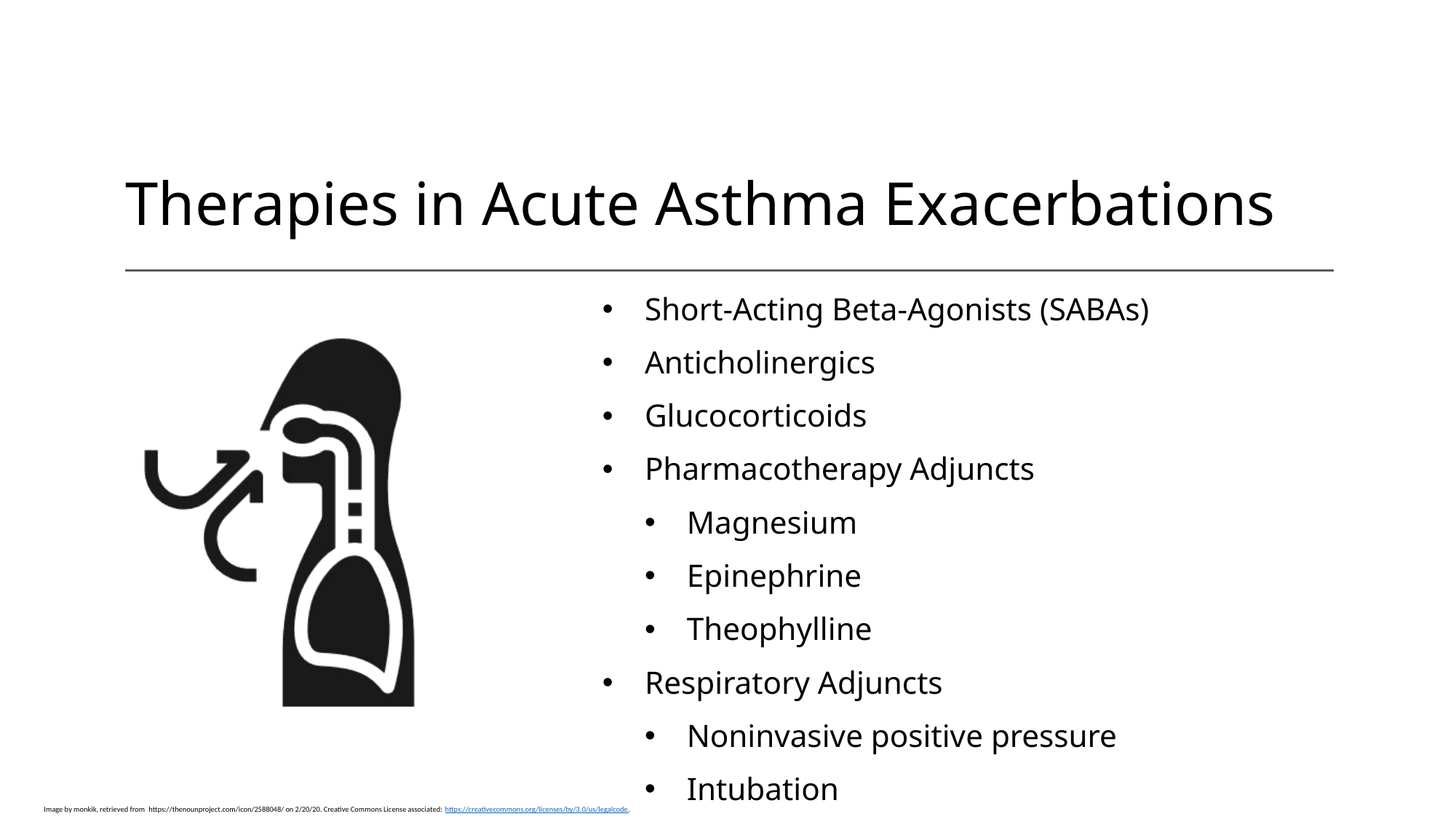

# Therapies in Acute Asthma Exacerbations
Short-Acting Beta-Agonists (SABAs)
Anticholinergics
Glucocorticoids
Pharmacotherapy Adjuncts
Magnesium
Epinephrine
Theophylline
Respiratory Adjuncts
Noninvasive positive pressure
Intubation
Image by monkik, retrieved from  https://thenounproject.com/icon/2588048/ on 2/20/20. Creative Commons License associated: https://creativecommons.org/licenses/by/3.0/us/legalcode.

## Slide 14
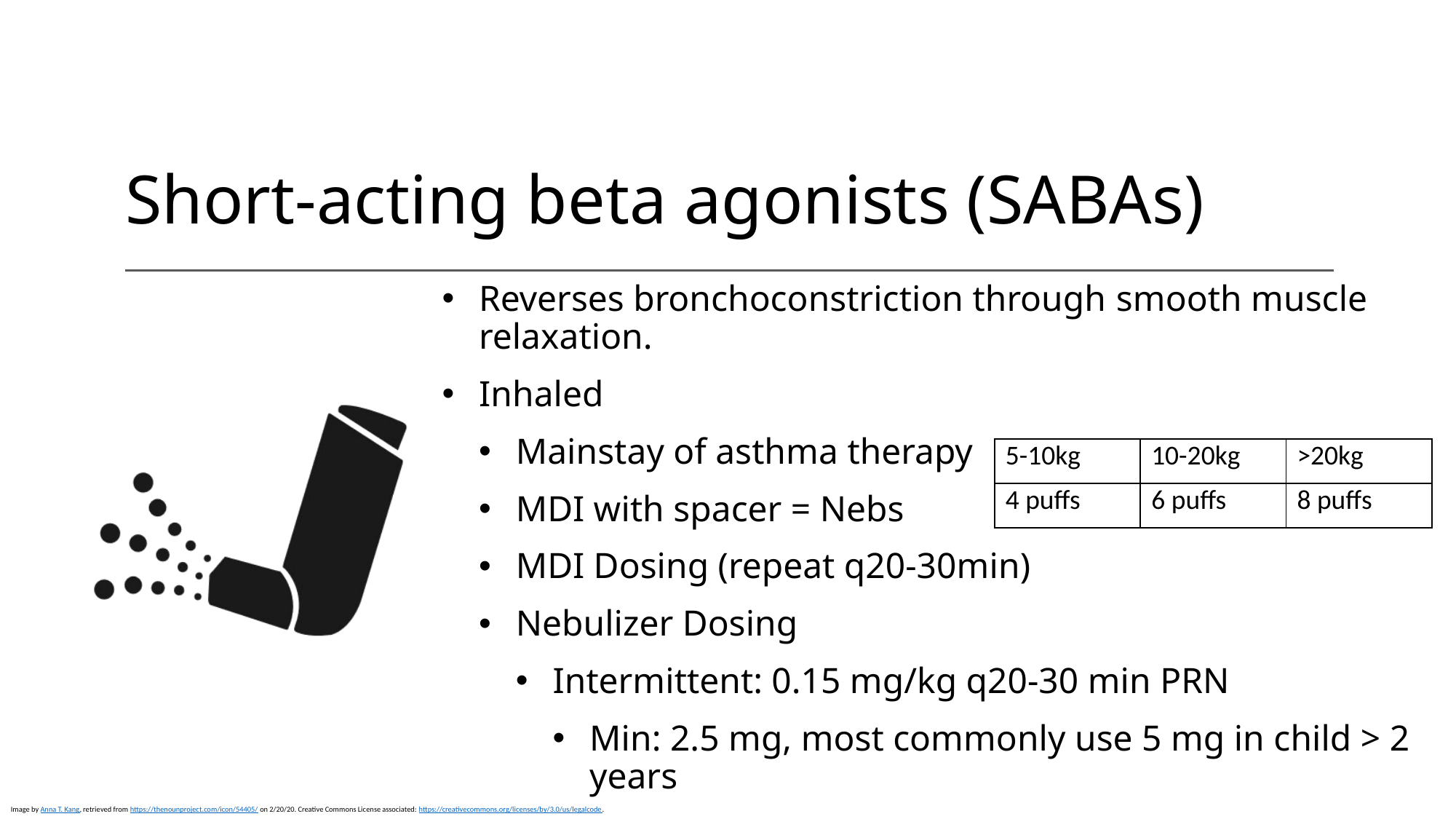

# Short-acting beta agonists (SABAs)
Reverses bronchoconstriction through smooth muscle relaxation.
Inhaled
Mainstay of asthma therapy
MDI with spacer = Nebs
MDI Dosing (repeat q20-30min)
Nebulizer Dosing
Intermittent: 0.15 mg/kg q20-30 min PRN
Min: 2.5 mg, most commonly use 5 mg in child > 2 years
Continuous: 0.5 mg/kg/hr (max 20 mg/hr)
| 5-10kg | 10-20kg | >20kg |
| --- | --- | --- |
| 4 puffs | 6 puffs | 8 puffs |
Image by Anna T. Kang, retrieved from https://thenounproject.com/icon/54405/ on 2/20/20. Creative Commons License associated: https://creativecommons.org/licenses/by/3.0/us/legalcode.

## Slide 15
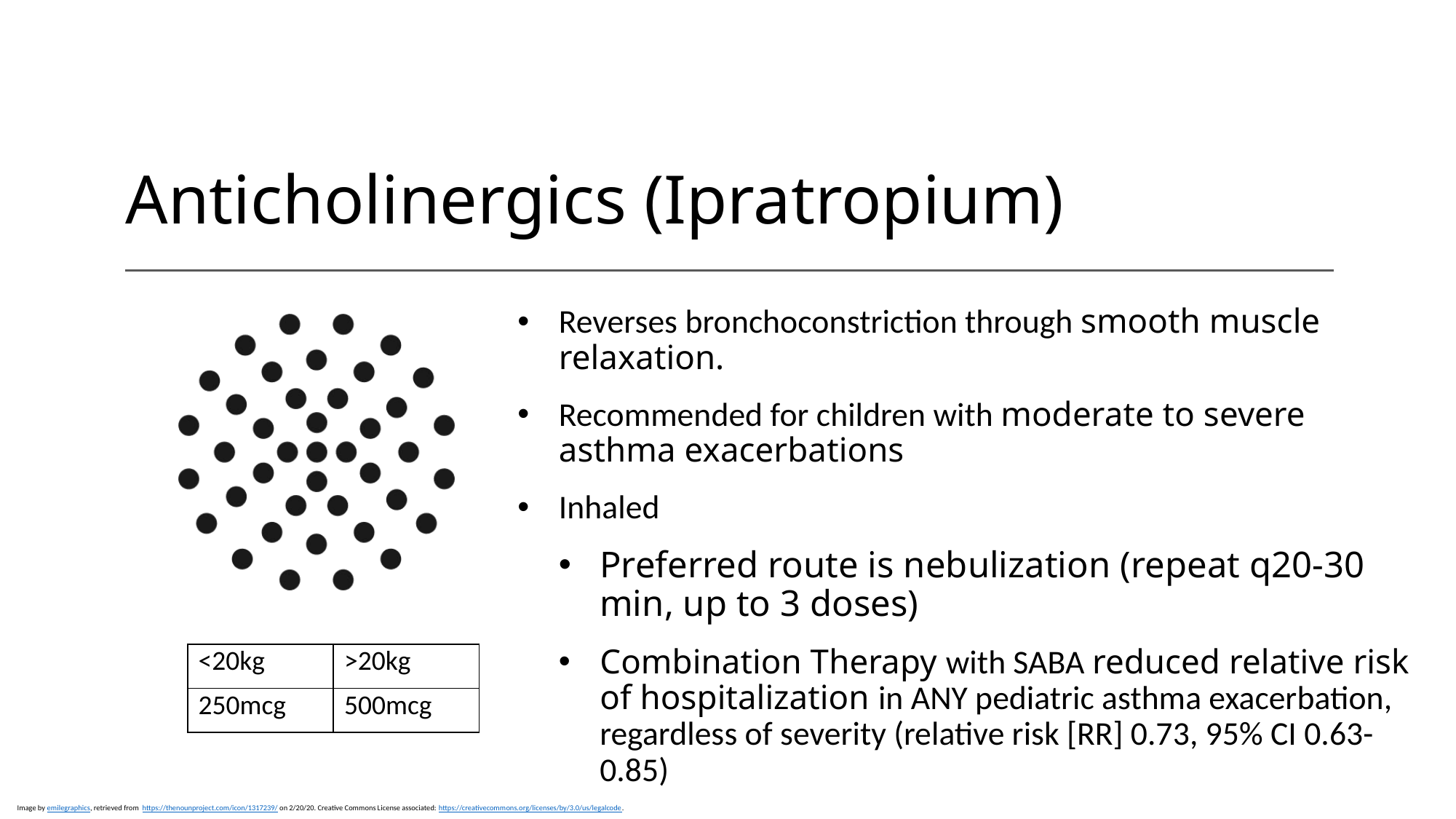

# Anticholinergics (Ipratropium)
Reverses bronchoconstriction through smooth muscle relaxation.
Recommended for children with moderate to severe asthma exacerbations
Inhaled
Preferred route is nebulization (repeat q20-30 min, up to 3 doses)
Combination Therapy with SABA reduced relative risk of hospitalization in ANY pediatric asthma exacerbation, regardless of severity (relative risk [RR] 0.73, 95% CI 0.63-0.85)
| <20kg | >20kg |
| --- | --- |
| 250mcg | 500mcg |
Image by emilegraphics, retrieved from  https://thenounproject.com/icon/1317239/ on 2/20/20. Creative Commons License associated: https://creativecommons.org/licenses/by/3.0/us/legalcode.

## Slide 16
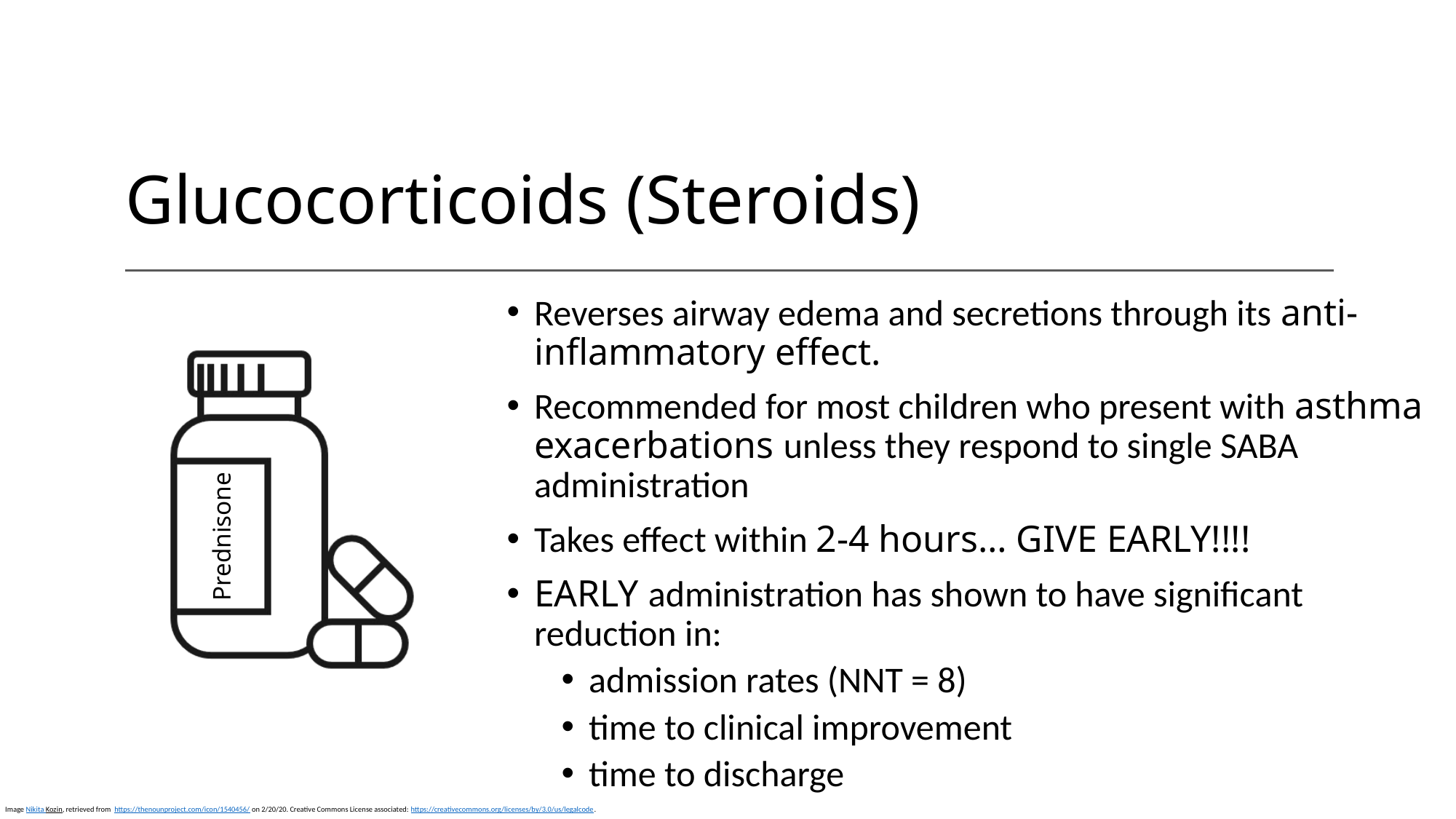

# Glucocorticoids (Steroids)
Reverses airway edema and secretions through its anti-inflammatory effect.
Recommended for most children who present with asthma exacerbations unless they respond to single SABA administration
Takes effect within 2-4 hours… GIVE EARLY!!!!
EARLY administration has shown to have significant reduction in:
admission rates (NNT = 8)
time to clinical improvement
time to discharge
Prednisone
Image Nikita Kozin, retrieved from  https://thenounproject.com/icon/1540456/ on 2/20/20. Creative Commons License associated: https://creativecommons.org/licenses/by/3.0/us/legalcode.

## Slide 17
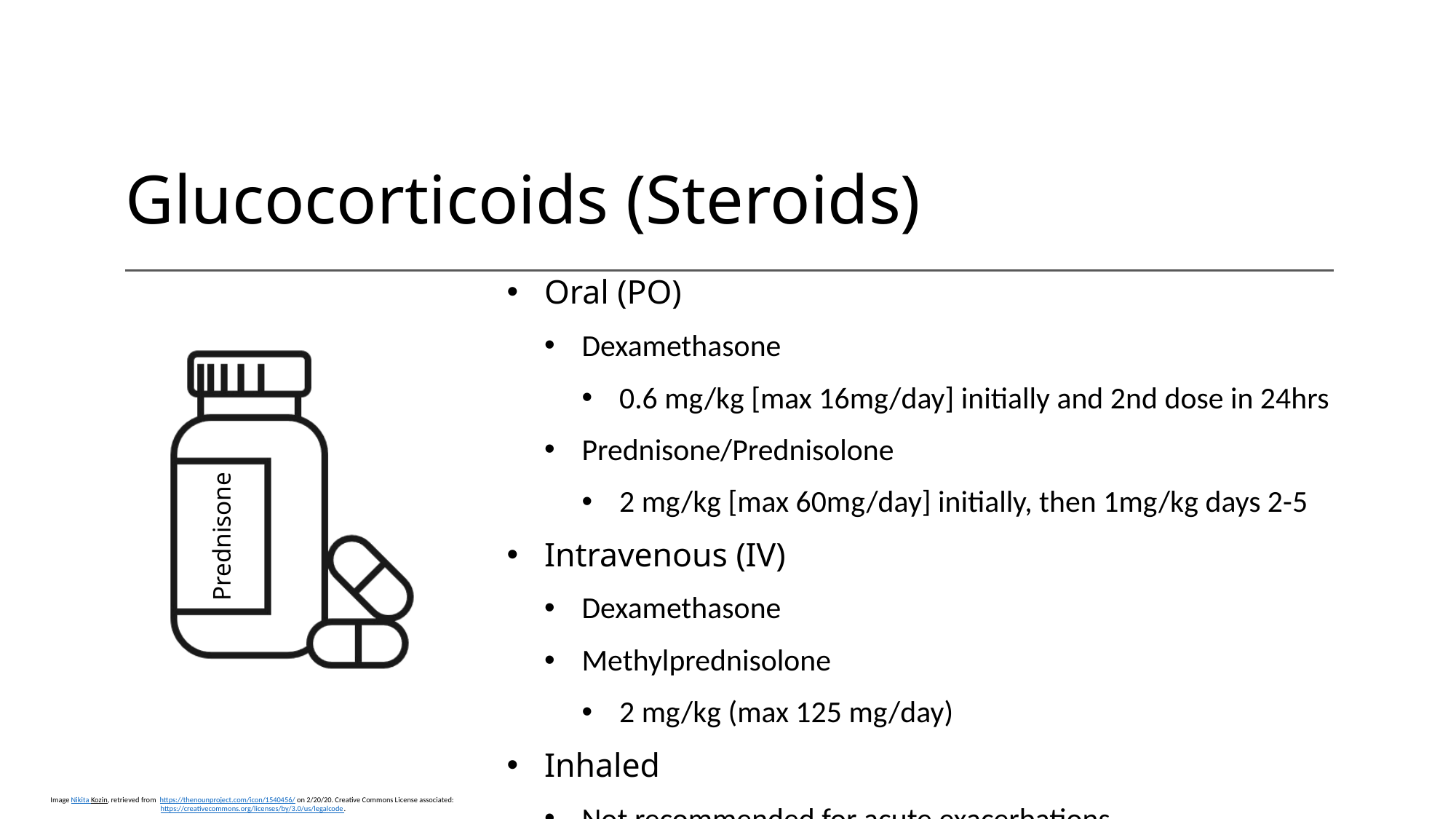

# Glucocorticoids (Steroids)
Oral (PO)
Dexamethasone
0.6 mg/kg [max 16mg/day] initially and 2nd dose in 24hrs
Prednisone/Prednisolone
2 mg/kg [max 60mg/day] initially, then 1mg/kg days 2-5
Intravenous (IV)
Dexamethasone
Methylprednisolone
2 mg/kg (max 125 mg/day)
Inhaled
Not recommended for acute exacerbations
Prednisone
Image Nikita Kozin, retrieved from  https://thenounproject.com/icon/1540456/ on 2/20/20. Creative Commons License associated: https://creativecommons.org/licenses/by/3.0/us/legalcode.

## Slide 18
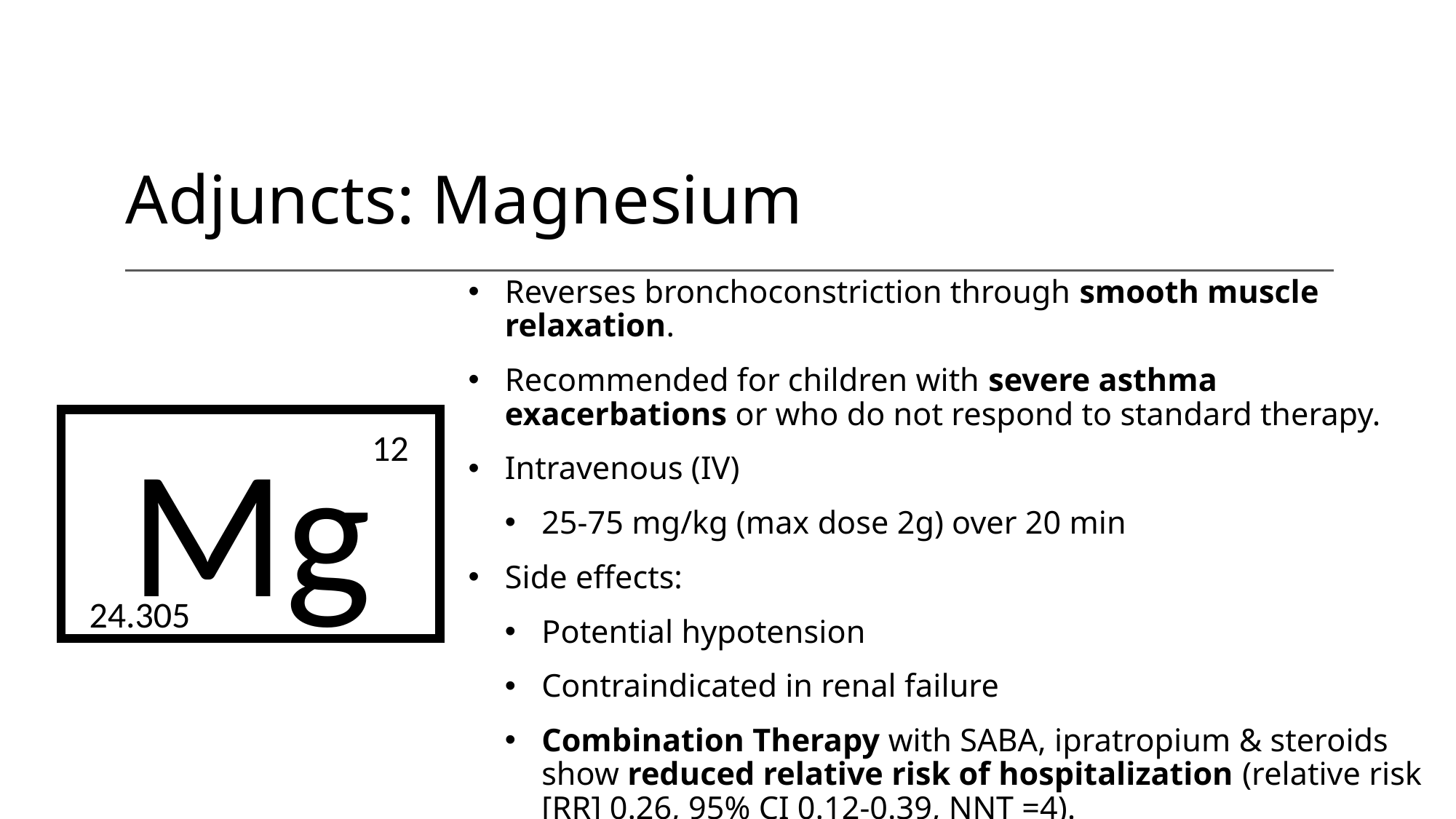

# Adjuncts: Magnesium
Reverses bronchoconstriction through smooth muscle relaxation.
Recommended for children with severe asthma exacerbations or who do not respond to standard therapy.
Intravenous (IV)
25-75 mg/kg (max dose 2g) over 20 min
Side effects:
Potential hypotension
Contraindicated in renal failure
Combination Therapy with SABA, ipratropium & steroids show reduced relative risk of hospitalization (relative risk [RR] 0.26, 95% CI 0.12-0.39, NNT =4).
Mg
12
24.305

## Slide 19
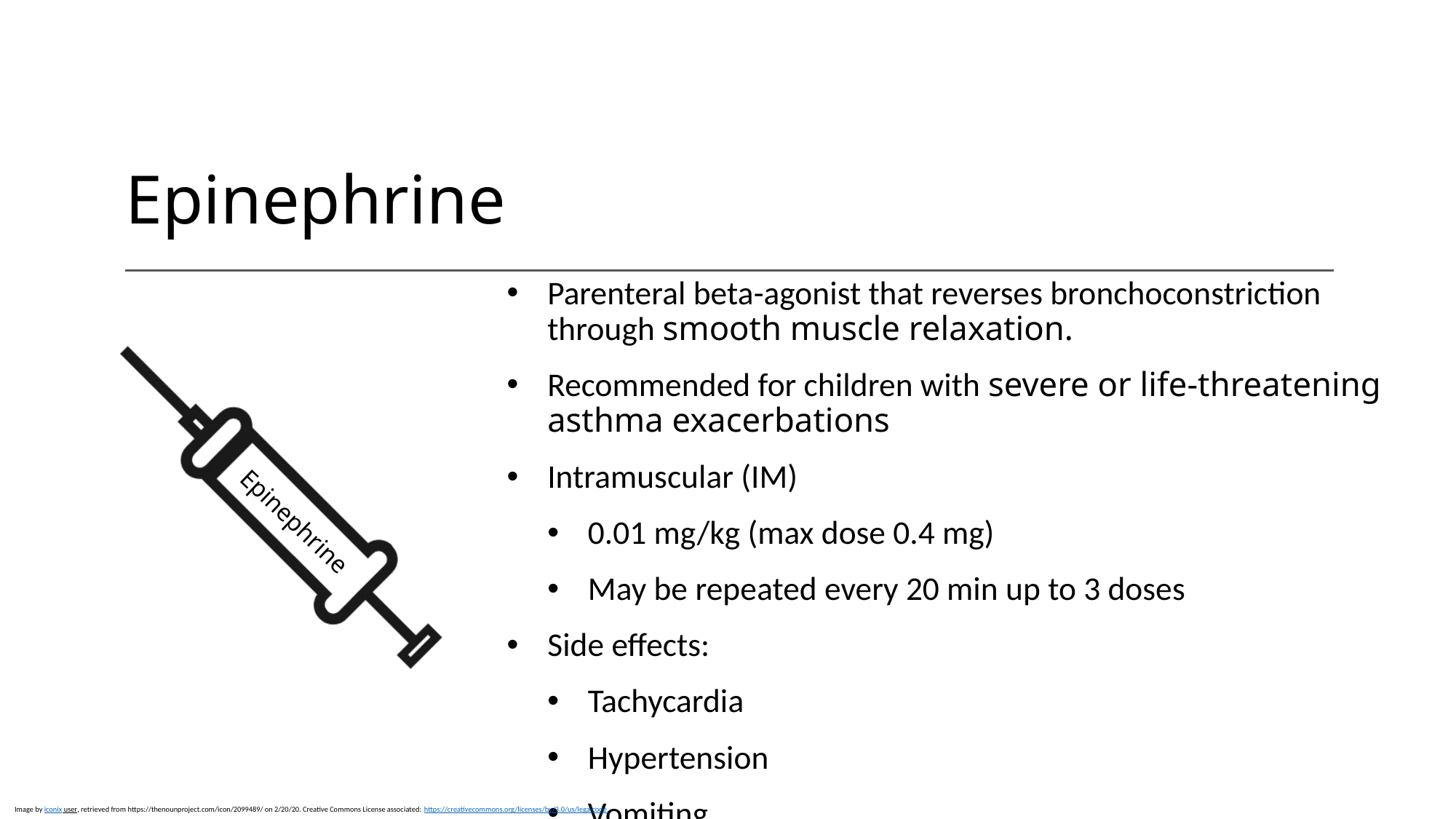

# Epinephrine
Parenteral beta-agonist that reverses bronchoconstriction through smooth muscle relaxation.
Recommended for children with severe or life-threatening asthma exacerbations
Intramuscular (IM)
0.01 mg/kg (max dose 0.4 mg)
May be repeated every 20 min up to 3 doses
Side effects:
Tachycardia
Hypertension
Vomiting
Epinephrine
Image by iconix user, retrieved from https://thenounproject.com/icon/2099489/ on 2/20/20. Creative Commons License associated: https://creativecommons.org/licenses/by/3.0/us/legalcode.

## Slide 20
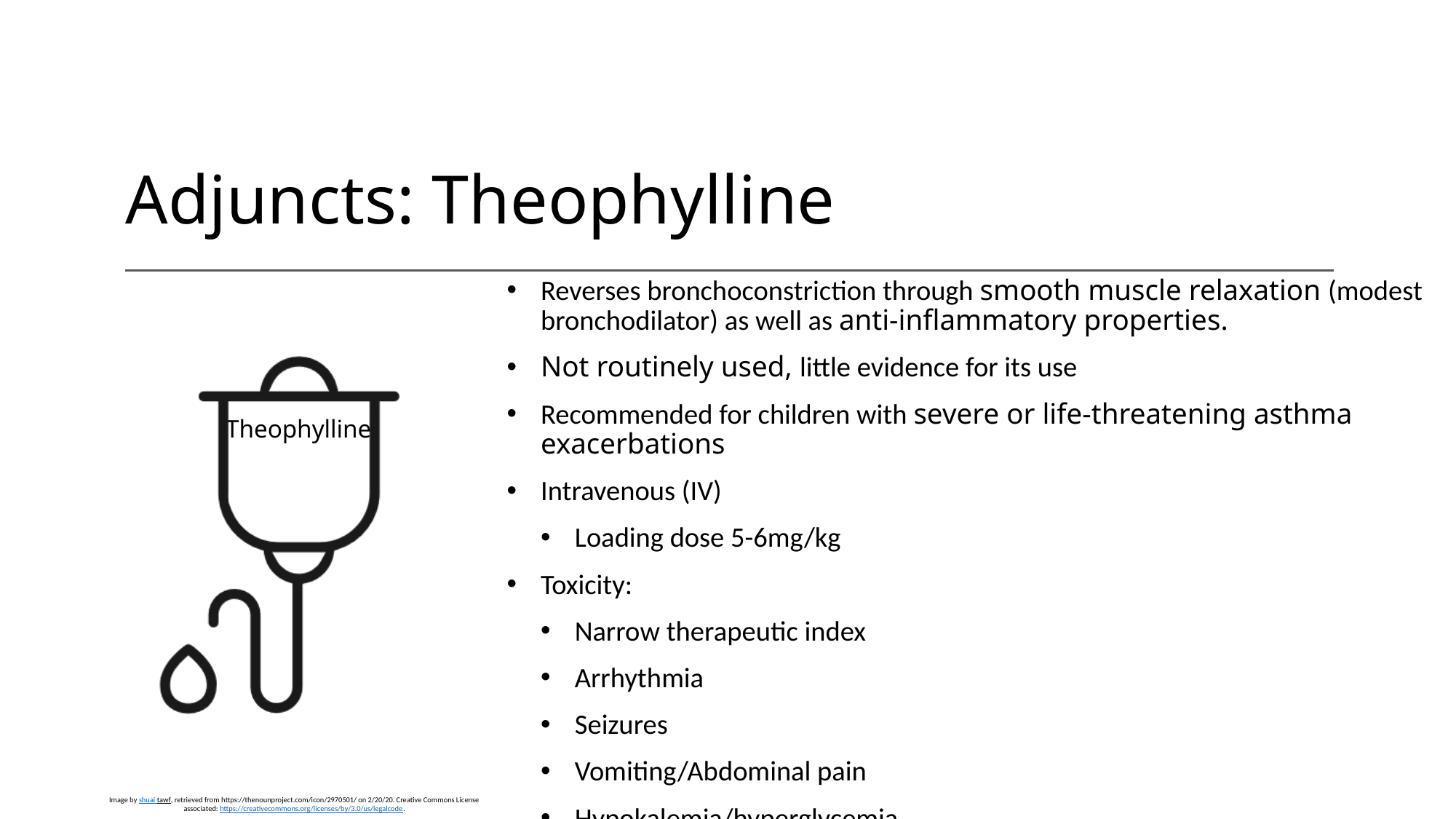

# Adjuncts: Theophylline
Reverses bronchoconstriction through smooth muscle relaxation (modest bronchodilator) as well as anti-inflammatory properties.
Not routinely used, little evidence for its use
Recommended for children with severe or life-threatening asthma exacerbations
Intravenous (IV)
Loading dose 5-6mg/kg
Toxicity:
Narrow therapeutic index
Arrhythmia
Seizures
Vomiting/Abdominal pain
Hypokalemia/hyperglycemia
Theophylline
Image by shuai tawf, retrieved from https://thenounproject.com/icon/2970501/ on 2/20/20. Creative Commons License associated: https://creativecommons.org/licenses/by/3.0/us/legalcode.

## Slide 21
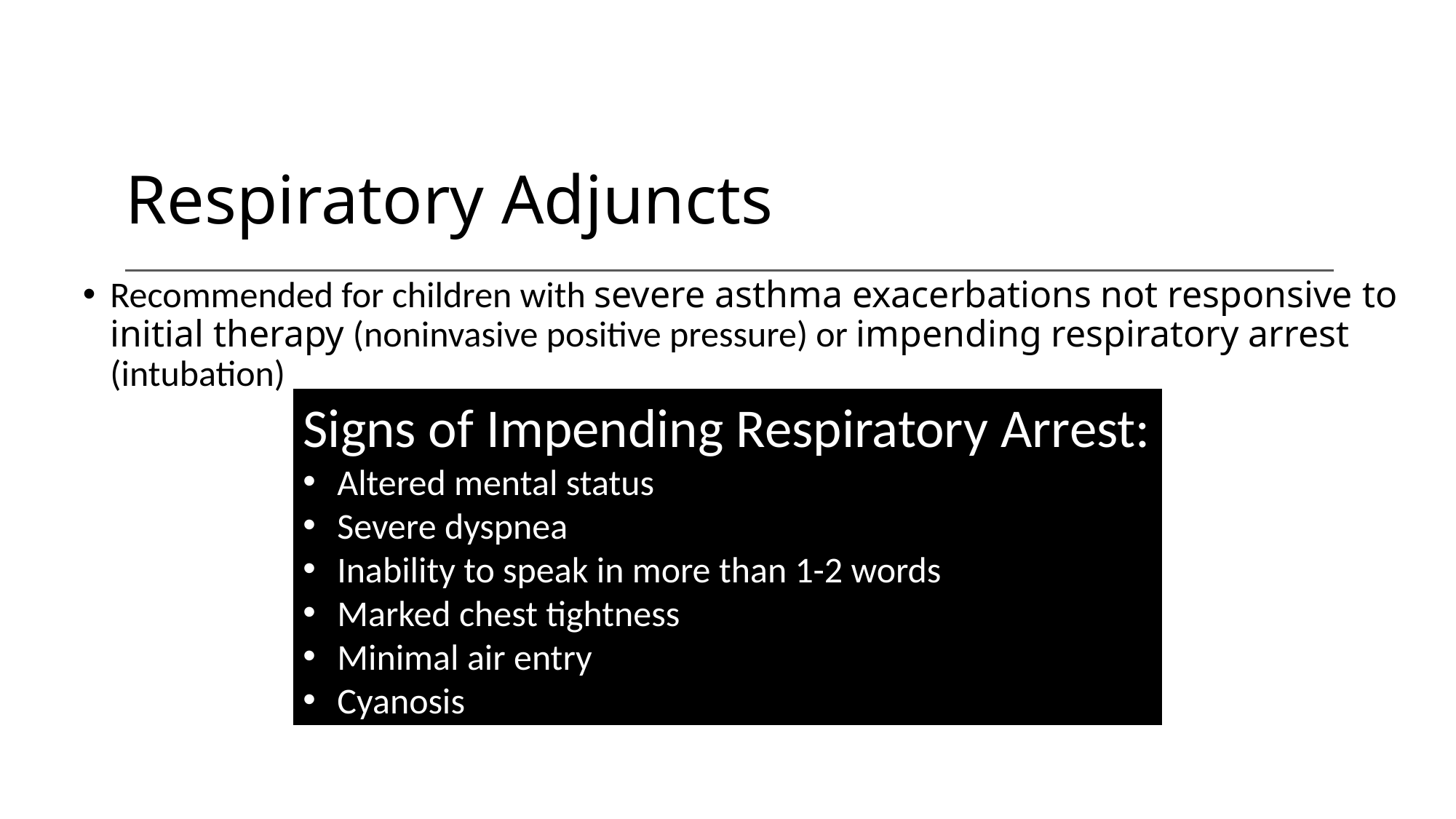

# Respiratory Adjuncts
Recommended for children with severe asthma exacerbations not responsive to initial therapy (noninvasive positive pressure) or impending respiratory arrest (intubation)
Signs of Impending Respiratory Arrest:
Altered mental status
Severe dyspnea
Inability to speak in more than 1-2 words
Marked chest tightness
Minimal air entry
Cyanosis

## Slide 22
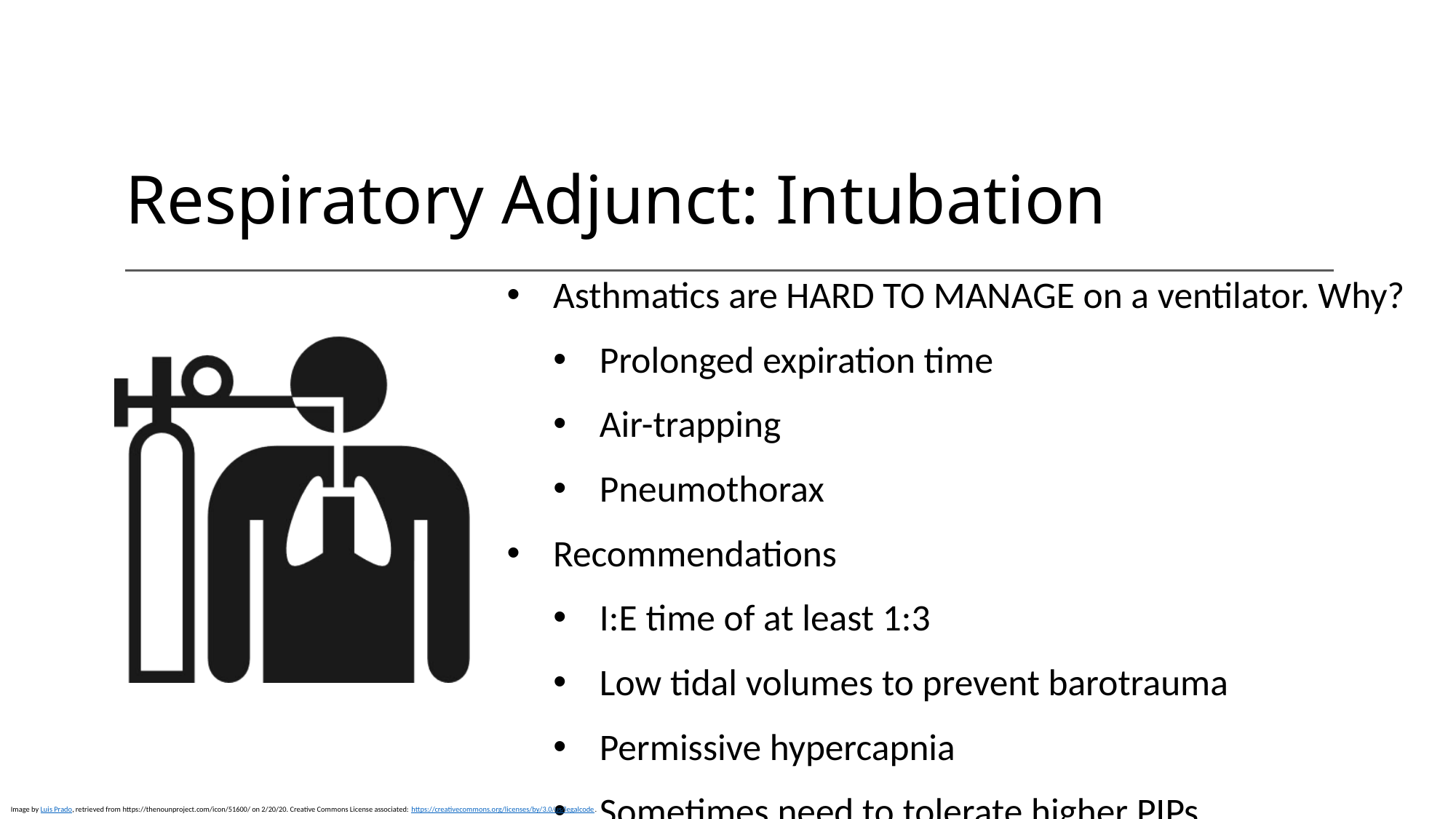

# Respiratory Adjunct: Intubation
Asthmatics are HARD TO MANAGE on a ventilator. Why?
Prolonged expiration time
Air-trapping
Pneumothorax
Recommendations
I:E time of at least 1:3
Low tidal volumes to prevent barotrauma
Permissive hypercapnia
Sometimes need to tolerate higher PIPs
Image by Luis Prado, retrieved from https://thenounproject.com/icon/51600/ on 2/20/20. Creative Commons License associated: https://creativecommons.org/licenses/by/3.0/us/legalcode.

## Slide 23
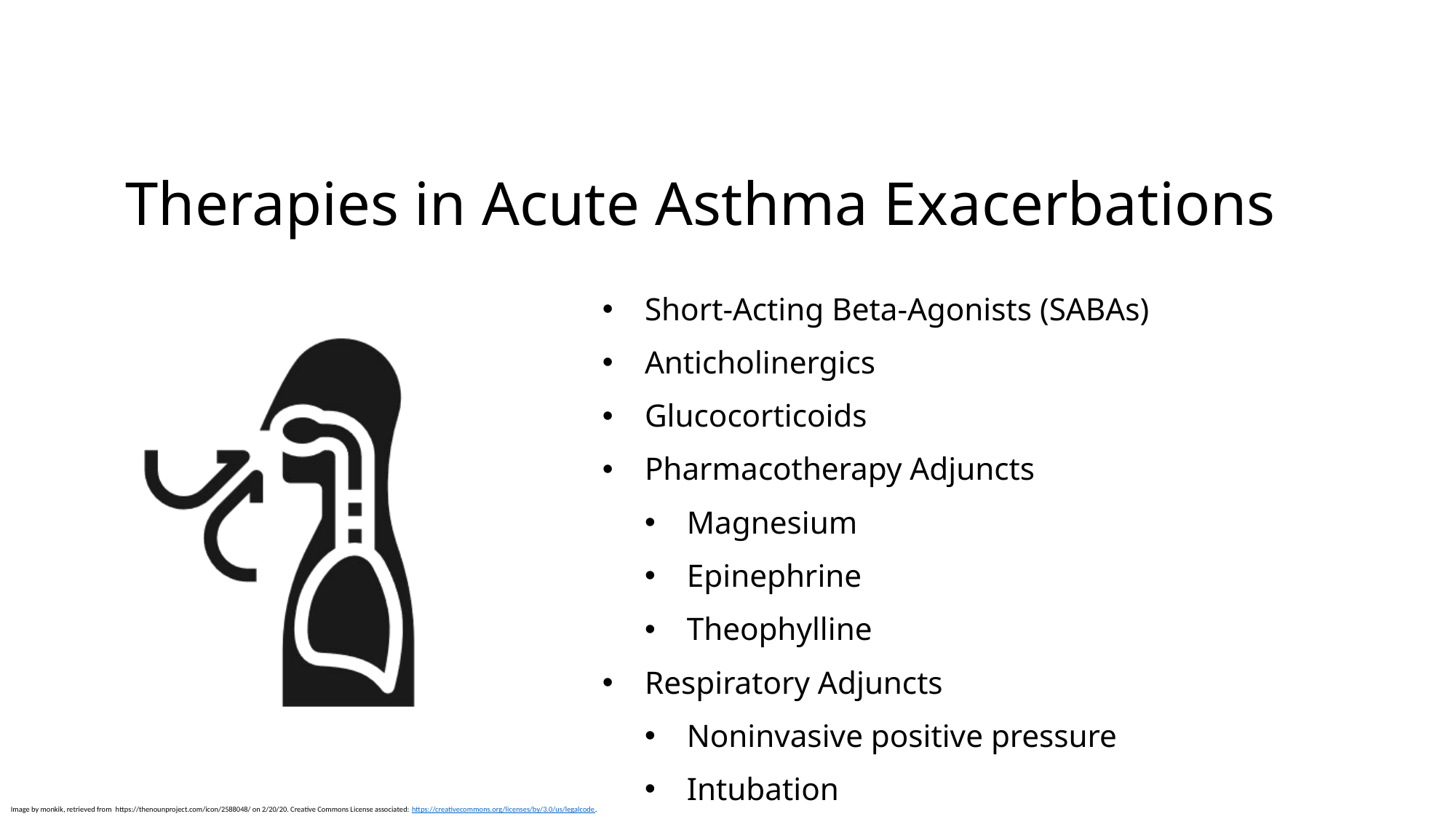

# Therapies in Acute Asthma Exacerbations
Short-Acting Beta-Agonists (SABAs)
Anticholinergics
Glucocorticoids
Pharmacotherapy Adjuncts
Magnesium
Epinephrine
Theophylline
Respiratory Adjuncts
Noninvasive positive pressure
Intubation
Image by monkik, retrieved from  https://thenounproject.com/icon/2588048/ on 2/20/20. Creative Commons License associated: https://creativecommons.org/licenses/by/3.0/us/legalcode.

## Slide 24
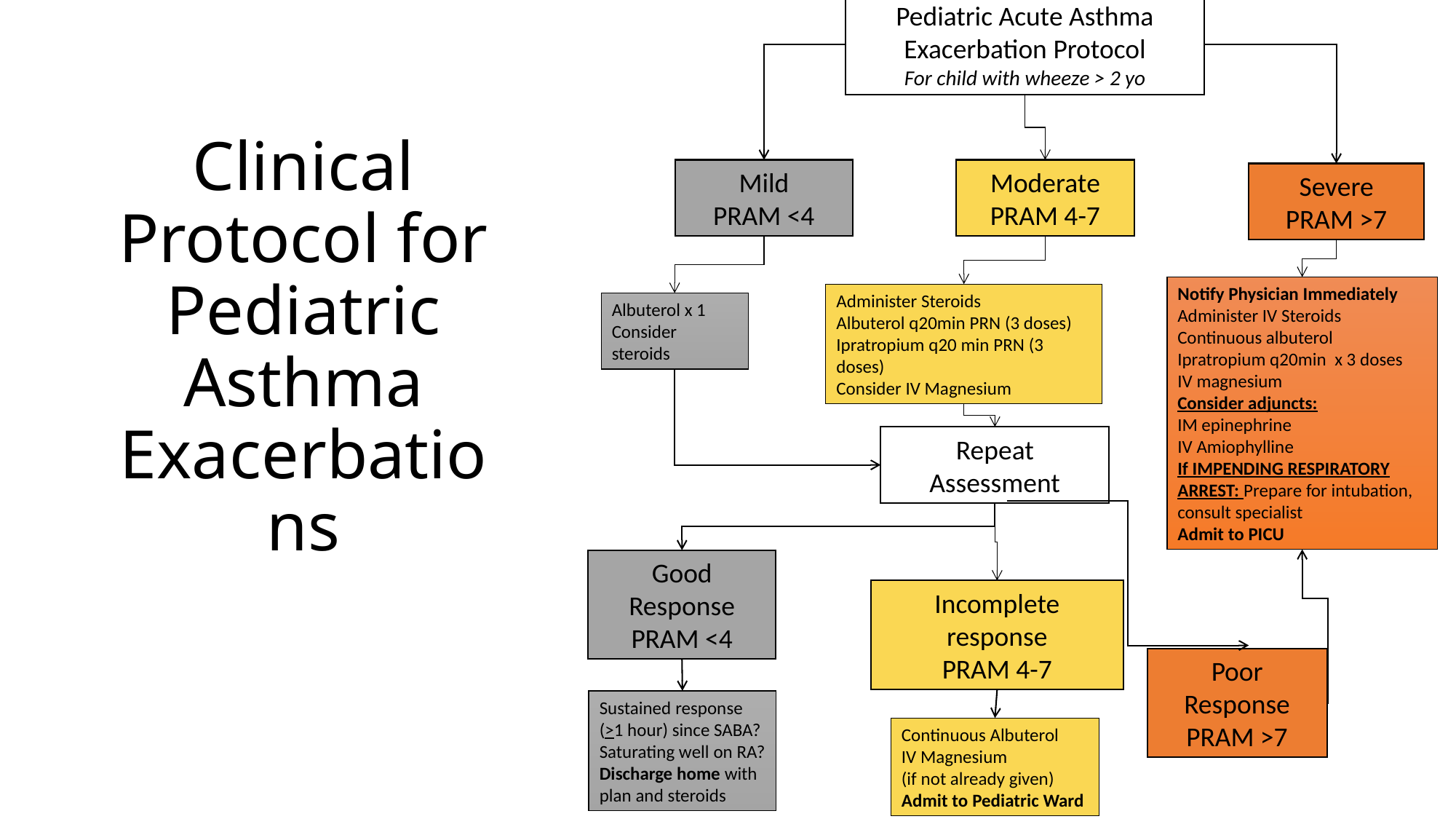

Pediatric Acute Asthma Exacerbation Protocol
For child with wheeze > 2 yo
Mild
PRAM <4
Moderate
PRAM 4-7
Severe
PRAM >7
Clinical Protocol for Pediatric Asthma Exacerbations
Notify Physician Immediately
Administer IV Steroids
Continuous albuterol
Ipratropium q20min x 3 doses
IV magnesium
Consider adjuncts:
IM epinephrine
IV Amiophylline
If IMPENDING RESPIRATORY ARREST: Prepare for intubation, consult specialist
Admit to PICU
Administer Steroids
Albuterol q20min PRN (3 doses)
Ipratropium q20 min PRN (3 doses)
Consider IV Magnesium
Albuterol x 1
Consider steroids
Repeat Assessment
Good Response
PRAM <4
Incomplete response
PRAM 4-7
Poor Response
PRAM >7
Sustained response (>1 hour) since SABA?
Saturating well on RA?
Discharge home with plan and steroids
Continuous Albuterol
IV Magnesium
(if not already given)
Admit to Pediatric Ward

## Slide 25
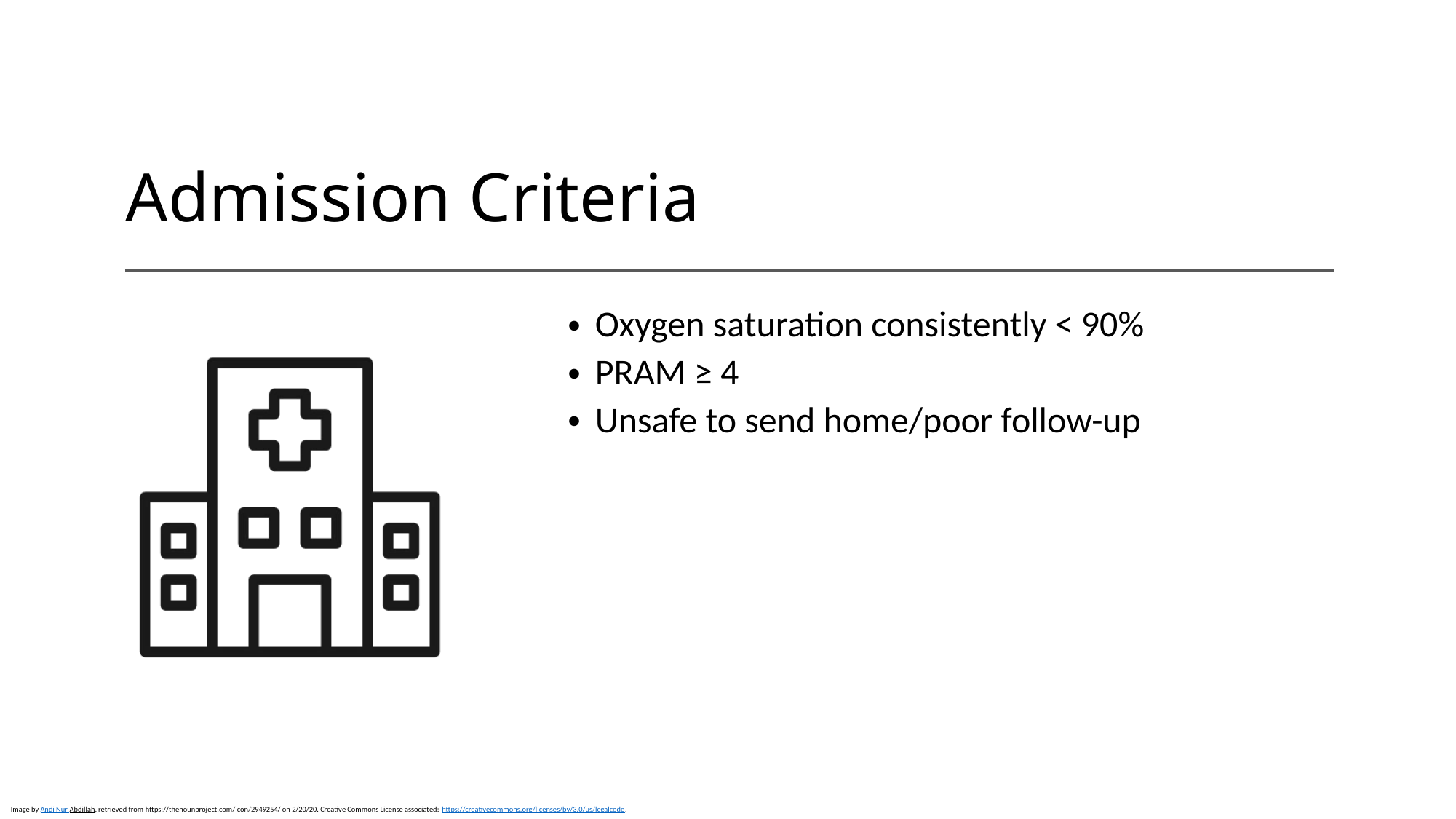

# Admission Criteria
Oxygen saturation consistently < 90%
PRAM ≥ 4
Unsafe to send home/poor follow-up
Image by Andi Nur Abdillah, retrieved from https://thenounproject.com/icon/2949254/ on 2/20/20. Creative Commons License associated: https://creativecommons.org/licenses/by/3.0/us/legalcode.

## Slide 26
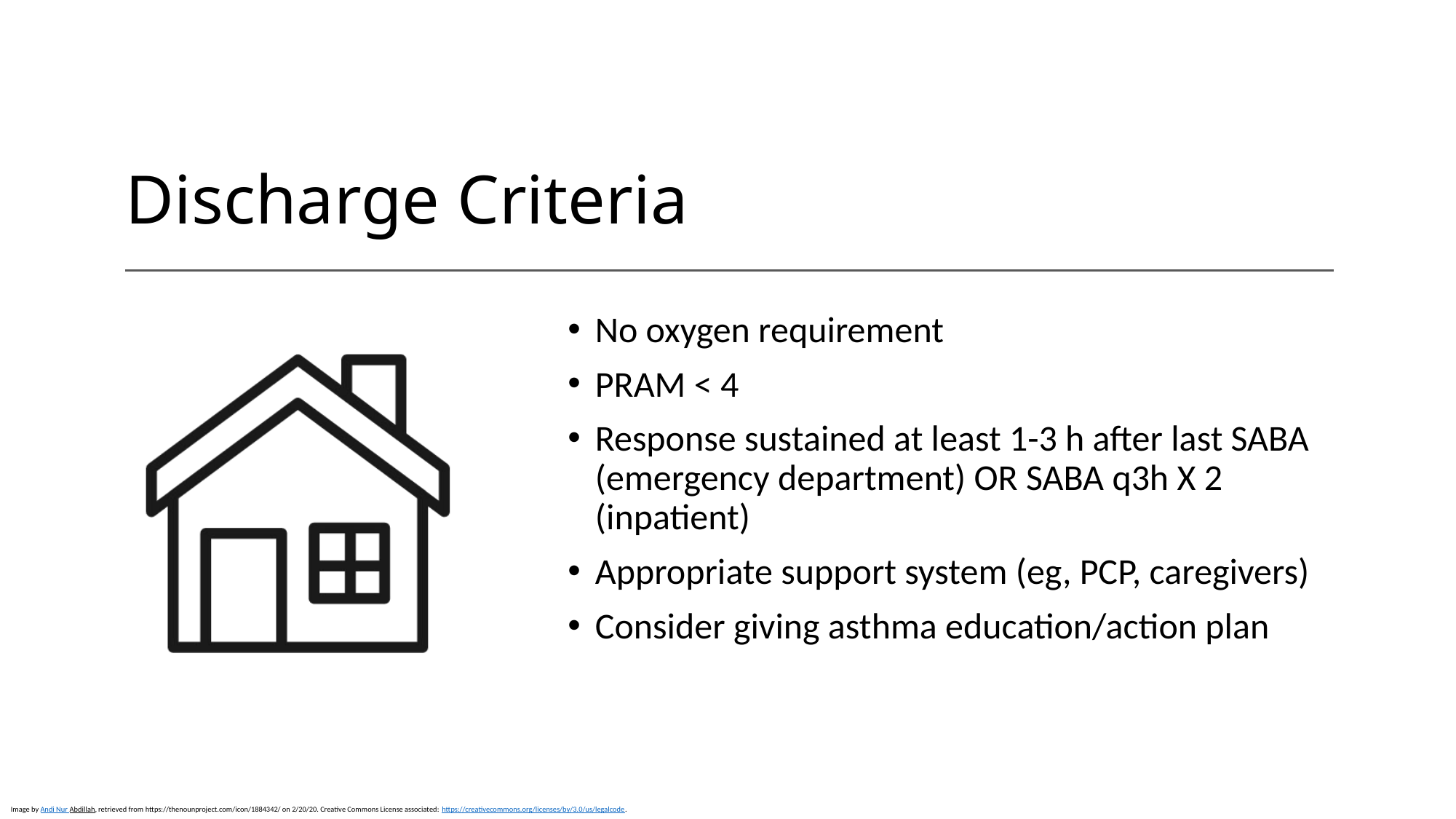

# Discharge Criteria
No oxygen requirement
PRAM < 4
Response sustained at least 1-3 h after last SABA (emergency department) OR SABA q3h X 2 (inpatient)
Appropriate support system (eg, PCP, caregivers)
Consider giving asthma education/action plan
Image by Andi Nur Abdillah, retrieved from https://thenounproject.com/icon/1884342/ on 2/20/20. Creative Commons License associated: https://creativecommons.org/licenses/by/3.0/us/legalcode.

## Slide 27
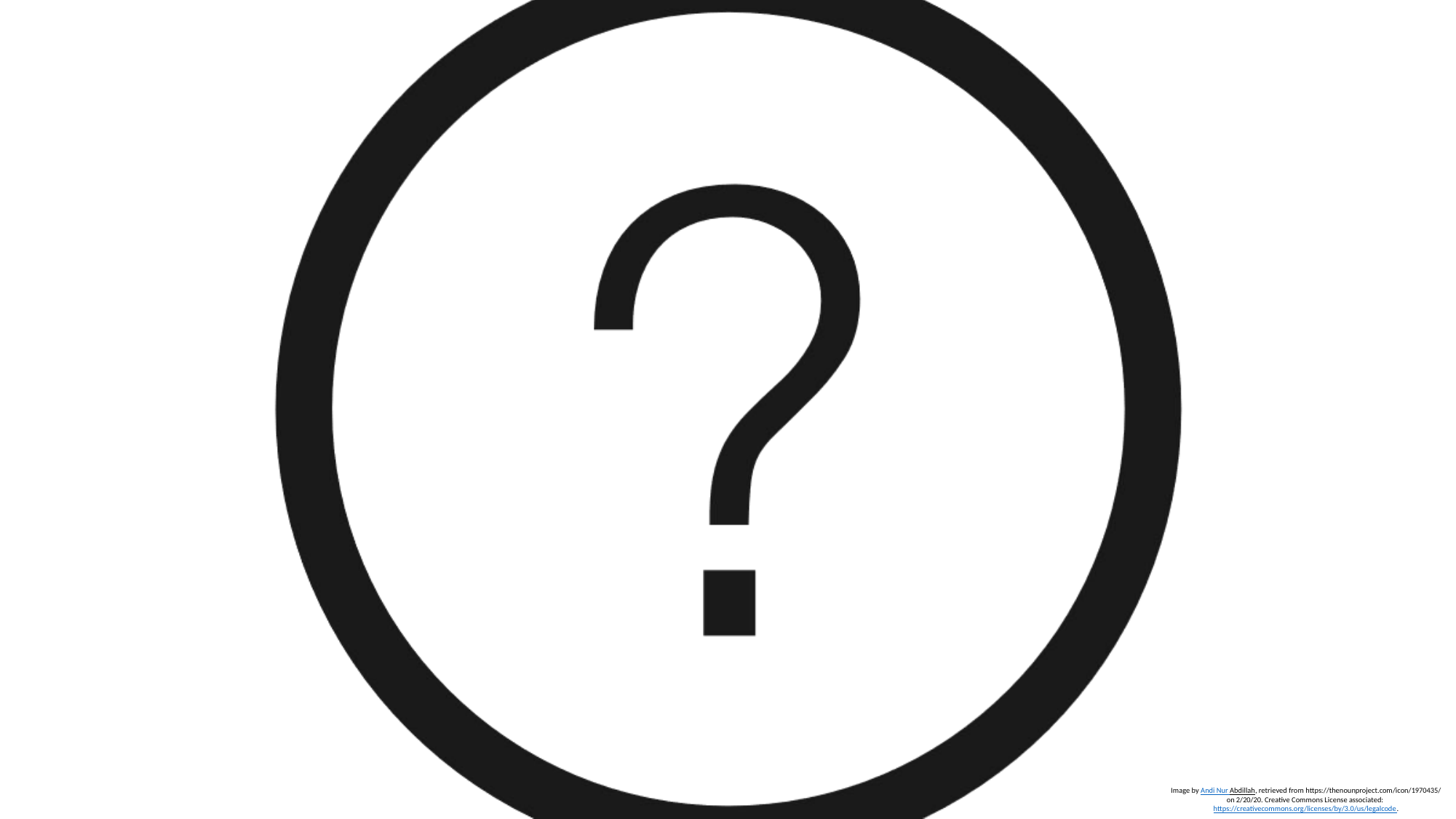

Image by Andi Nur Abdillah, retrieved from https://thenounproject.com/icon/1970435/ on 2/20/20. Creative Commons License associated: https://creativecommons.org/licenses/by/3.0/us/legalcode.
