# Supplementary material for: A Pediatric Emergency Medicine Refresher Course for Generalist Healthcare Providers in Belize: Respiratory Emergencies
Source: J Educ Teach Emerg Med. 2021 Apr 19;6(2):C73–C188. doi: 10.21980/J84063 (PMC10332788; doi:10.21980/J84063)
Supplement: Supplementary file 4 — Please see associated PowerPoint file [file jetem-6-2-c73-AppendixL.pptx]

## Slide 1
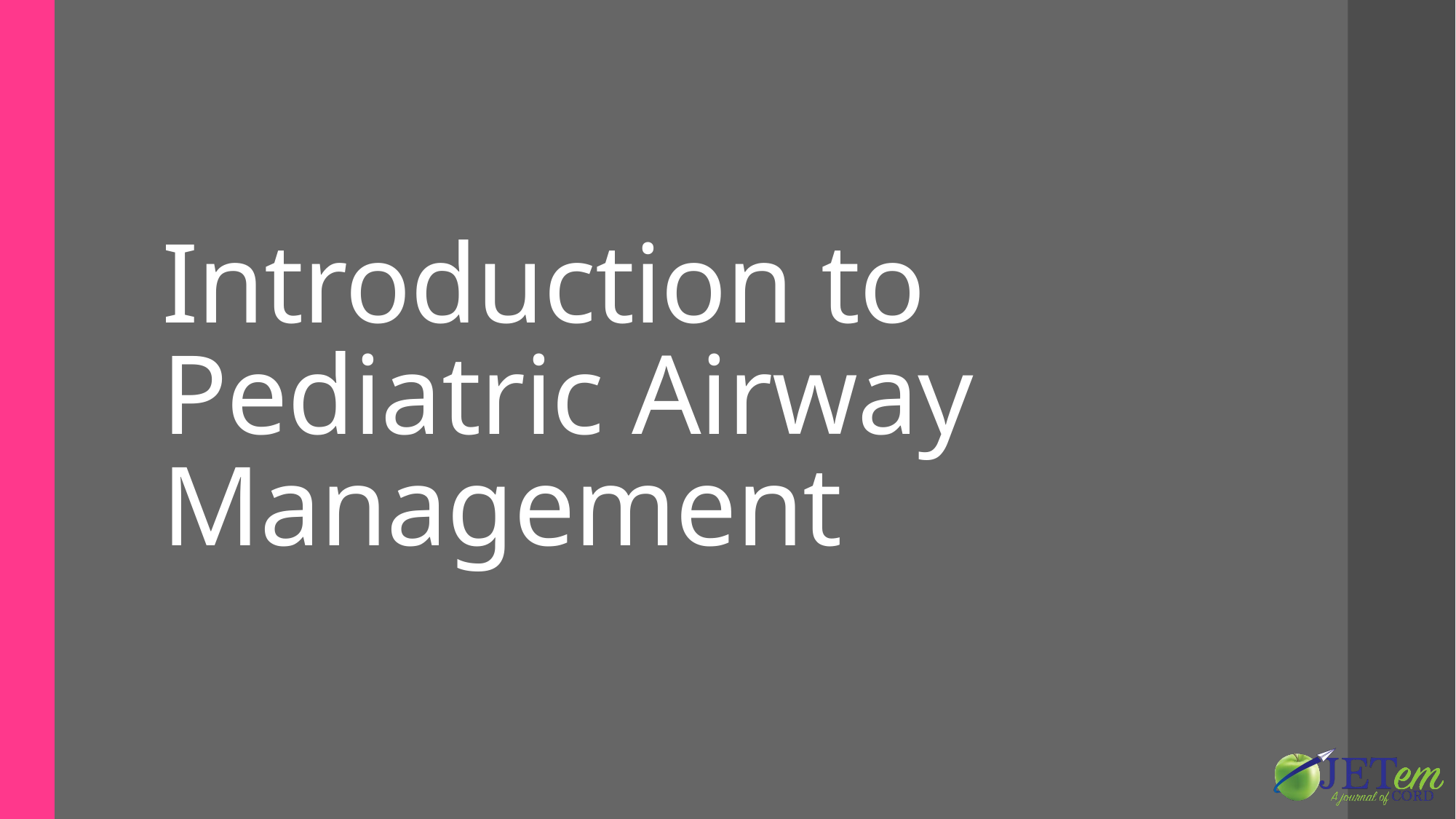

# Introduction to Pediatric Airway Management

## Slide 2
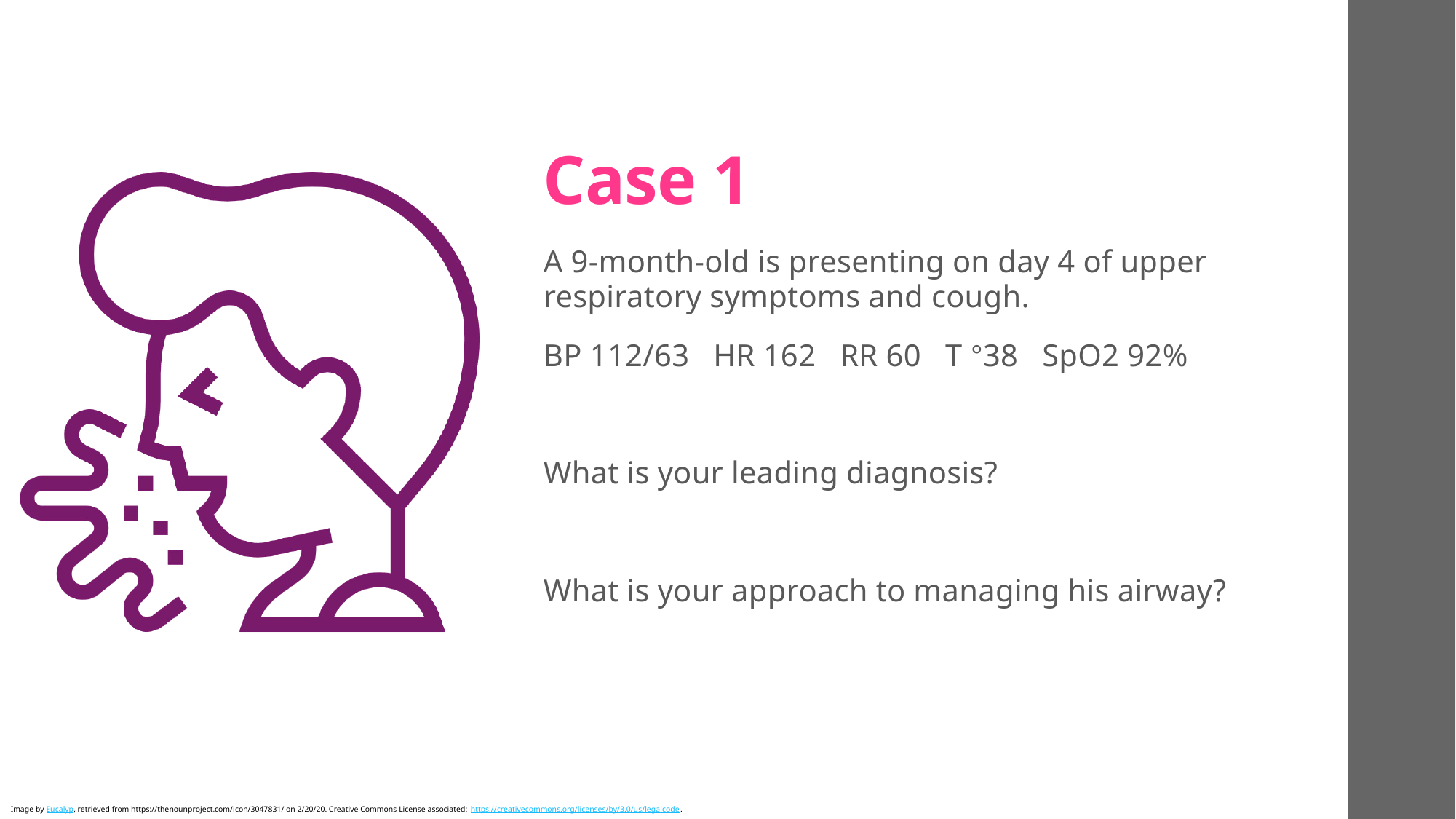

# Case 1
A 9-month-old is presenting on day 4 of upper respiratory symptoms and cough.
BP 112/63 HR 162 RR 60 T °38 SpO2 92%
What is your leading diagnosis?
What is your approach to managing his airway?
Image by Eucalyp, retrieved from https://thenounproject.com/icon/3047831/ on 2/20/20. Creative Commons License associated: https://creativecommons.org/licenses/by/3.0/us/legalcode.

## Slide 3
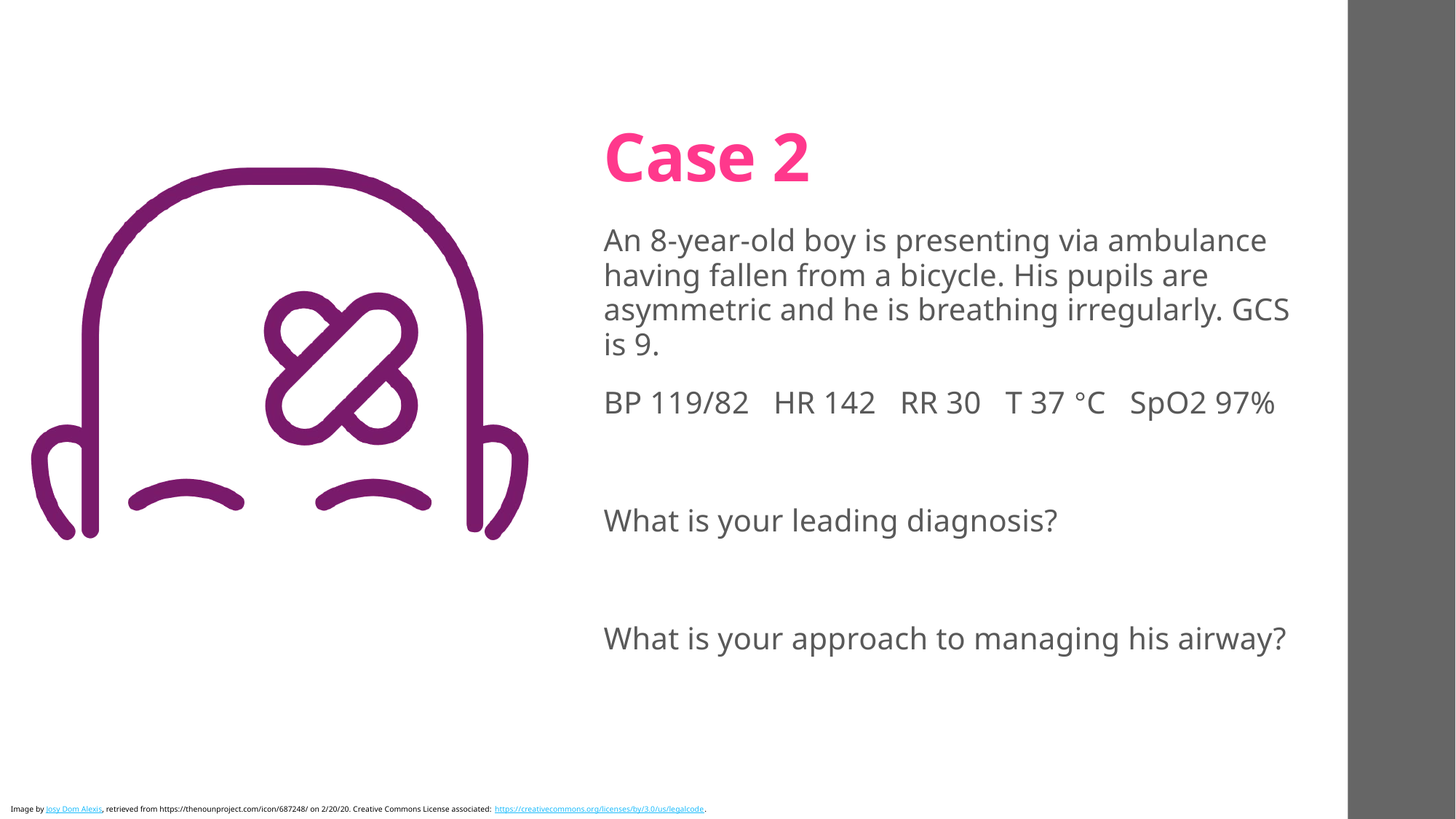

# Case 2
An 8-year-old boy is presenting via ambulance having fallen from a bicycle. His pupils are asymmetric and he is breathing irregularly. GCS is 9.
BP 119/82 HR 142 RR 30 T 37 °C SpO2 97%
What is your leading diagnosis?
What is your approach to managing his airway?
Image by Josy Dom Alexis, retrieved from https://thenounproject.com/icon/687248/ on 2/20/20. Creative Commons License associated: https://creativecommons.org/licenses/by/3.0/us/legalcode.

## Slide 4
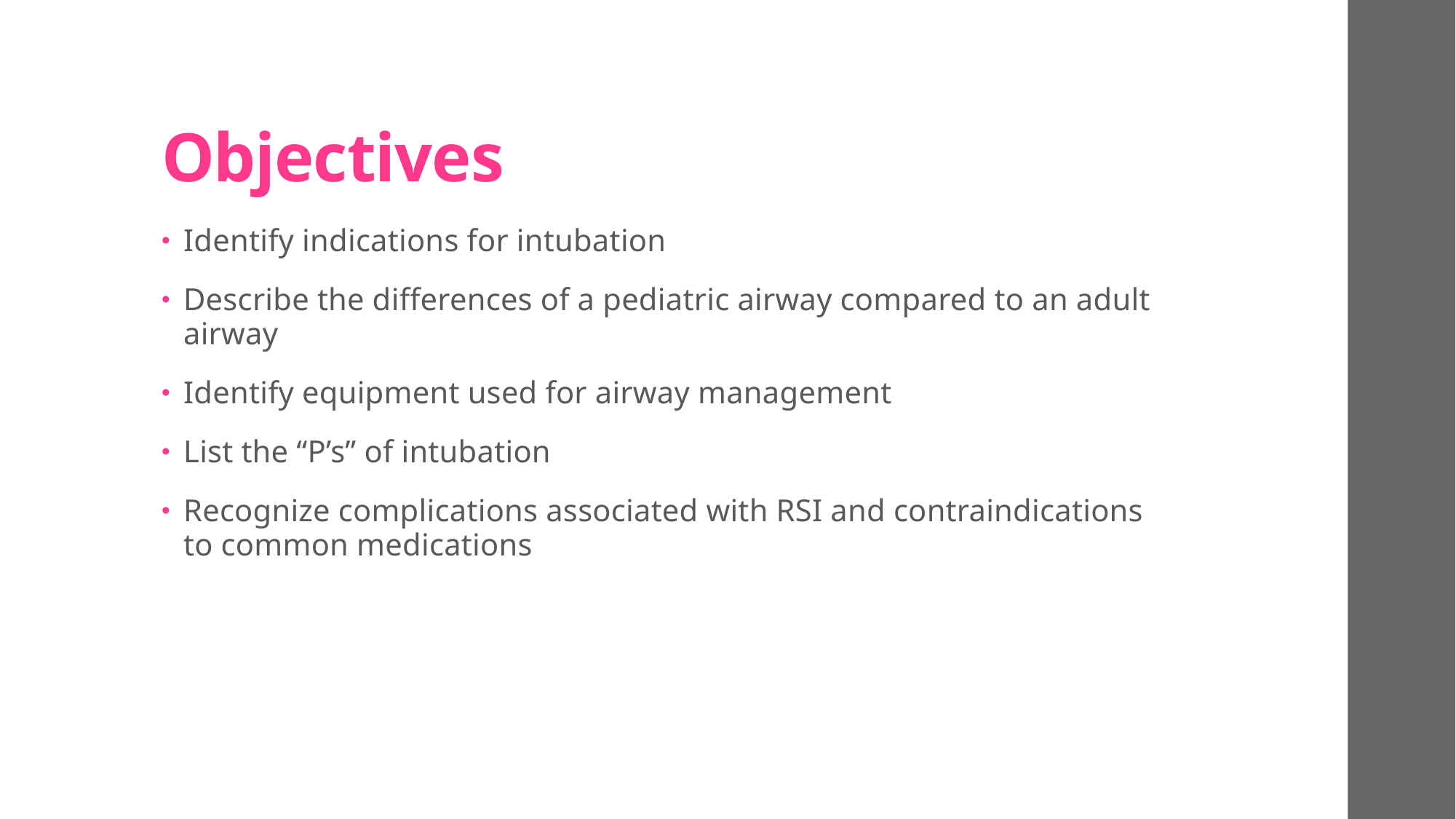

# Objectives
Identify indications for intubation
Describe the differences of a pediatric airway compared to an adult airway
Identify equipment used for airway management
List the “P’s” of intubation
Recognize complications associated with RSI and contraindications to common medications

## Slide 5
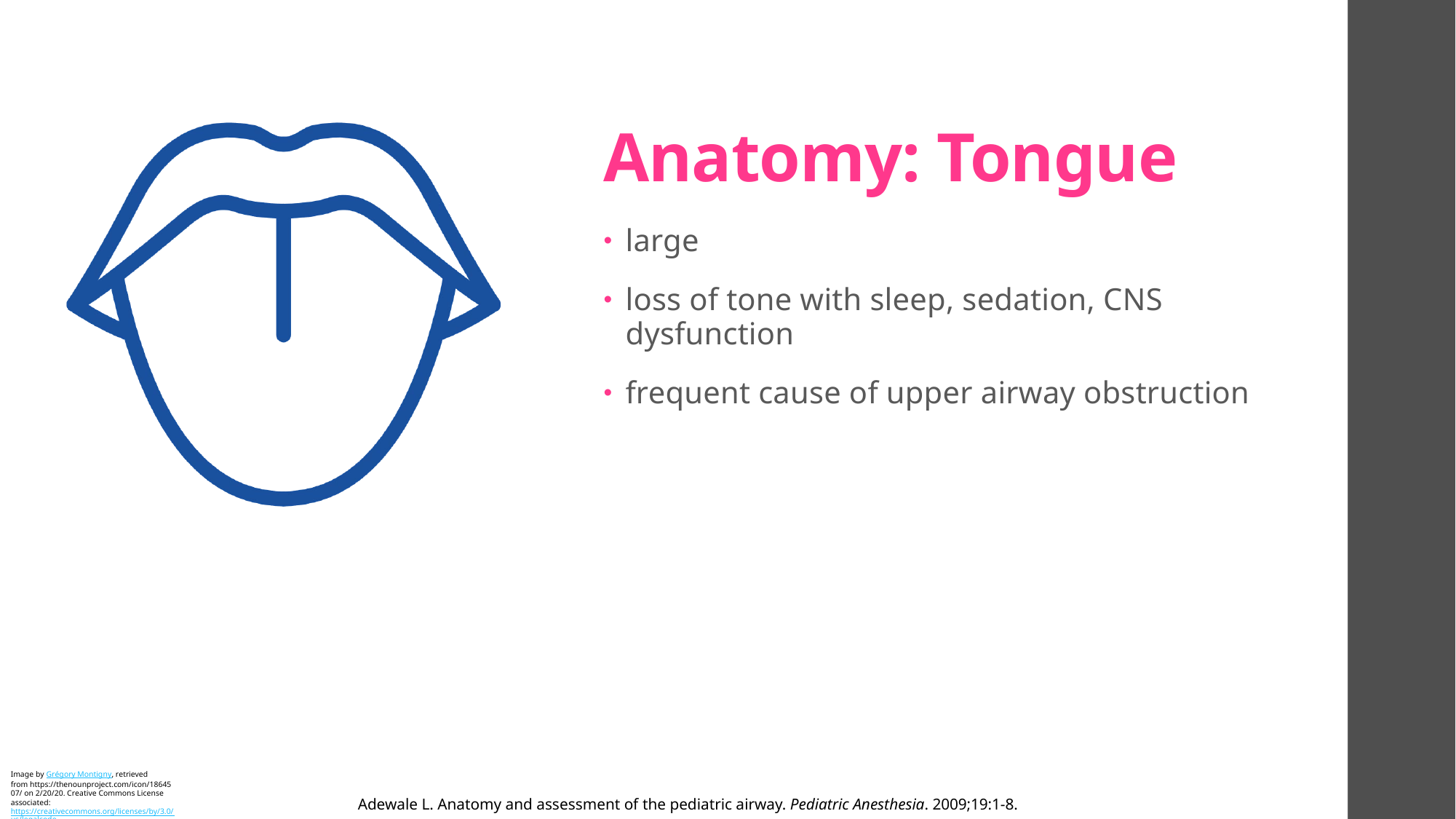

# Anatomy: Tongue
large
loss of tone with sleep, sedation, CNS dysfunction
frequent cause of upper airway obstruction
Image by Grégory Montigny, retrieved from https://thenounproject.com/icon/1864507/ on 2/20/20. Creative Commons License associated: https://creativecommons.org/licenses/by/3.0/us/legalcode.
Adewale L. Anatomy and assessment of the pediatric airway. Pediatric Anesthesia. 2009;19:1-8.

## Slide 6
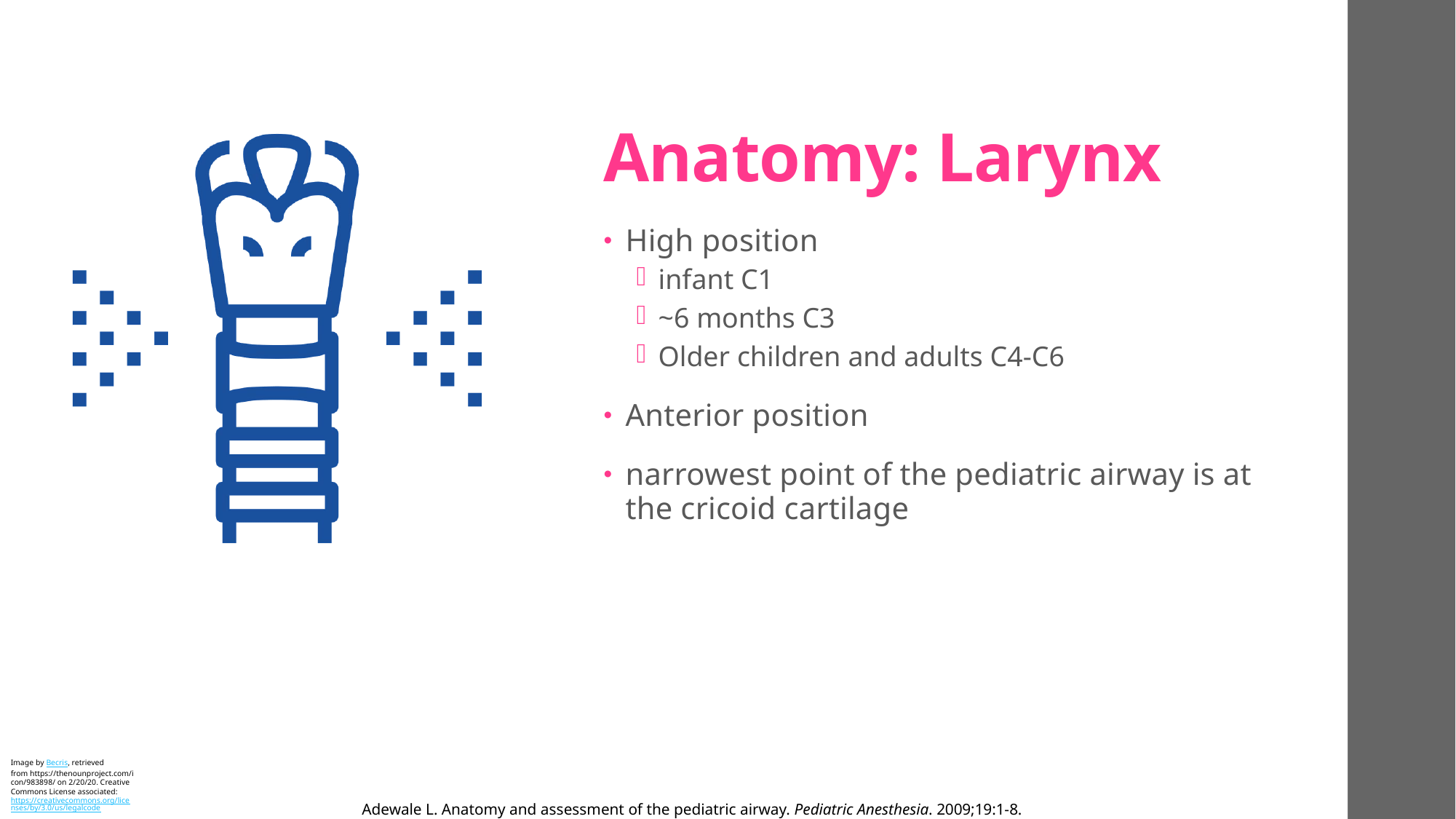

# Anatomy: Larynx
High position
infant C1
~6 months C3
Older children and adults C4-C6
Anterior position
narrowest point of the pediatric airway is at the cricoid cartilage
Image by Becris, retrieved from https://thenounproject.com/icon/983898/ on 2/20/20. Creative Commons License associated: https://creativecommons.org/licenses/by/3.0/us/legalcode.
Adewale L. Anatomy and assessment of the pediatric airway. Pediatric Anesthesia. 2009;19:1-8.

## Slide 7
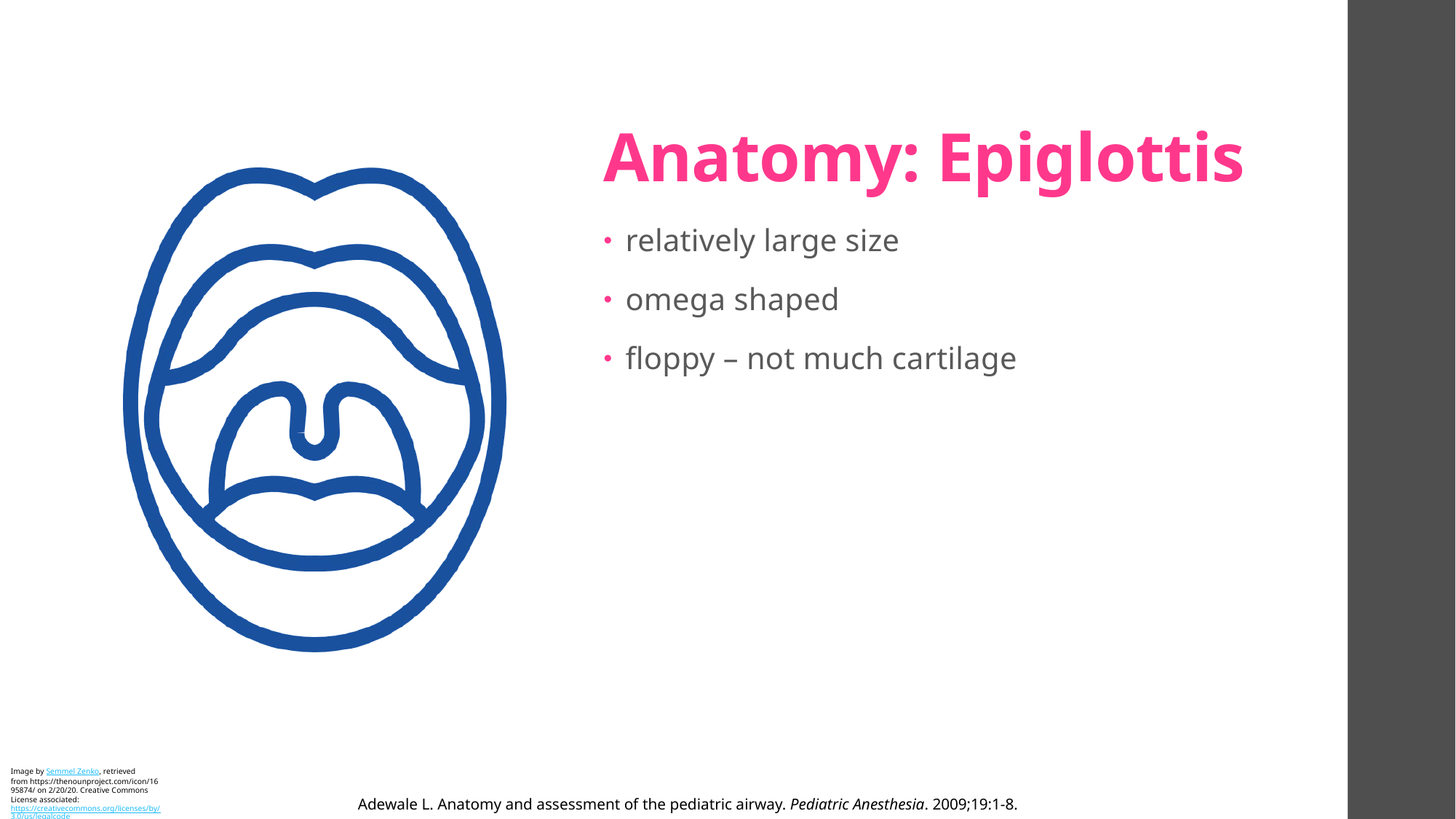

# Anatomy: Epiglottis
relatively large size
omega shaped
floppy – not much cartilage
Image by Semmel Zenko, retrieved from https://thenounproject.com/icon/1695874/ on 2/20/20. Creative Commons License associated: https://creativecommons.org/licenses/by/3.0/us/legalcode.
Adewale L. Anatomy and assessment of the pediatric airway. Pediatric Anesthesia. 2009;19:1-8.

## Slide 8
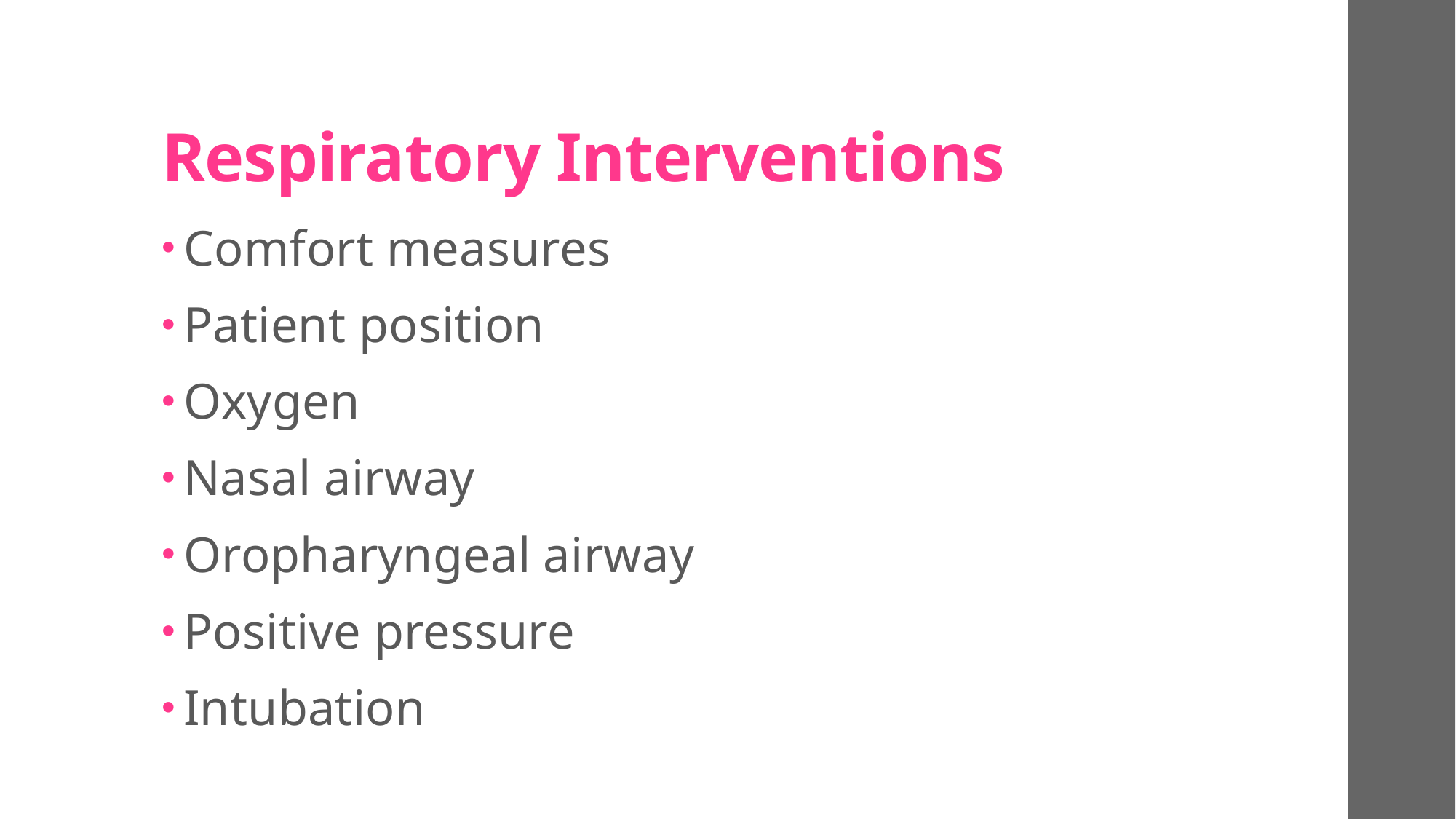

# Respiratory Interventions
Comfort measures
Patient position
Oxygen
Nasal airway
Oropharyngeal airway
Positive pressure
Intubation

## Slide 9
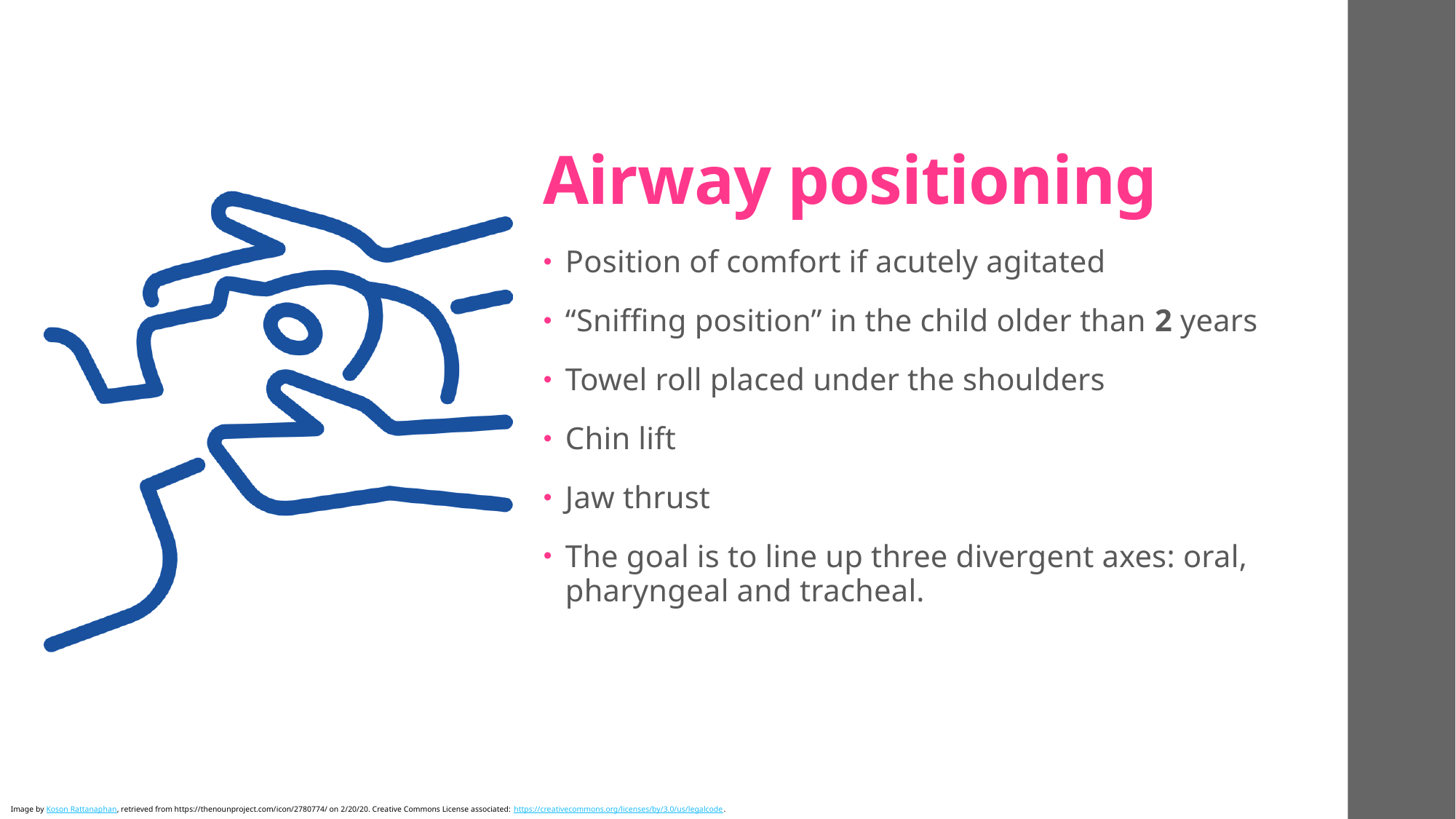

# Airway positioning
Position of comfort if acutely agitated
“Sniffing position” in the child older than 2 years
Towel roll placed under the shoulders
Chin lift
Jaw thrust
The goal is to line up three divergent axes: oral, pharyngeal and tracheal.
Image by Koson Rattanaphan, retrieved from https://thenounproject.com/icon/2780774/ on 2/20/20. Creative Commons License associated: https://creativecommons.org/licenses/by/3.0/us/legalcode.

## Slide 10
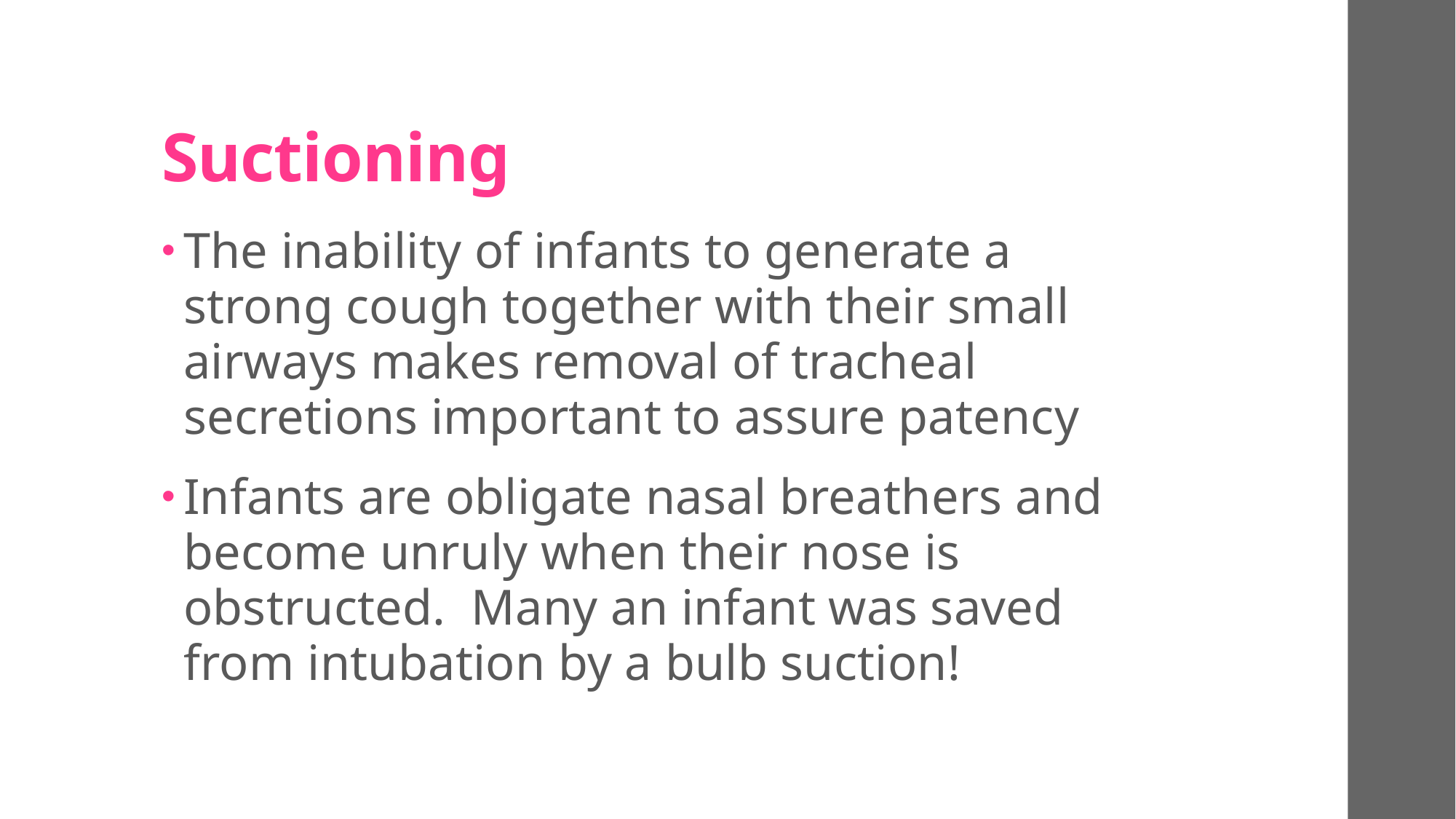

# Suctioning
The inability of infants to generate a strong cough together with their small airways makes removal of tracheal secretions important to assure patency
Infants are obligate nasal breathers and become unruly when their nose is obstructed. Many an infant was saved from intubation by a bulb suction!

## Slide 11
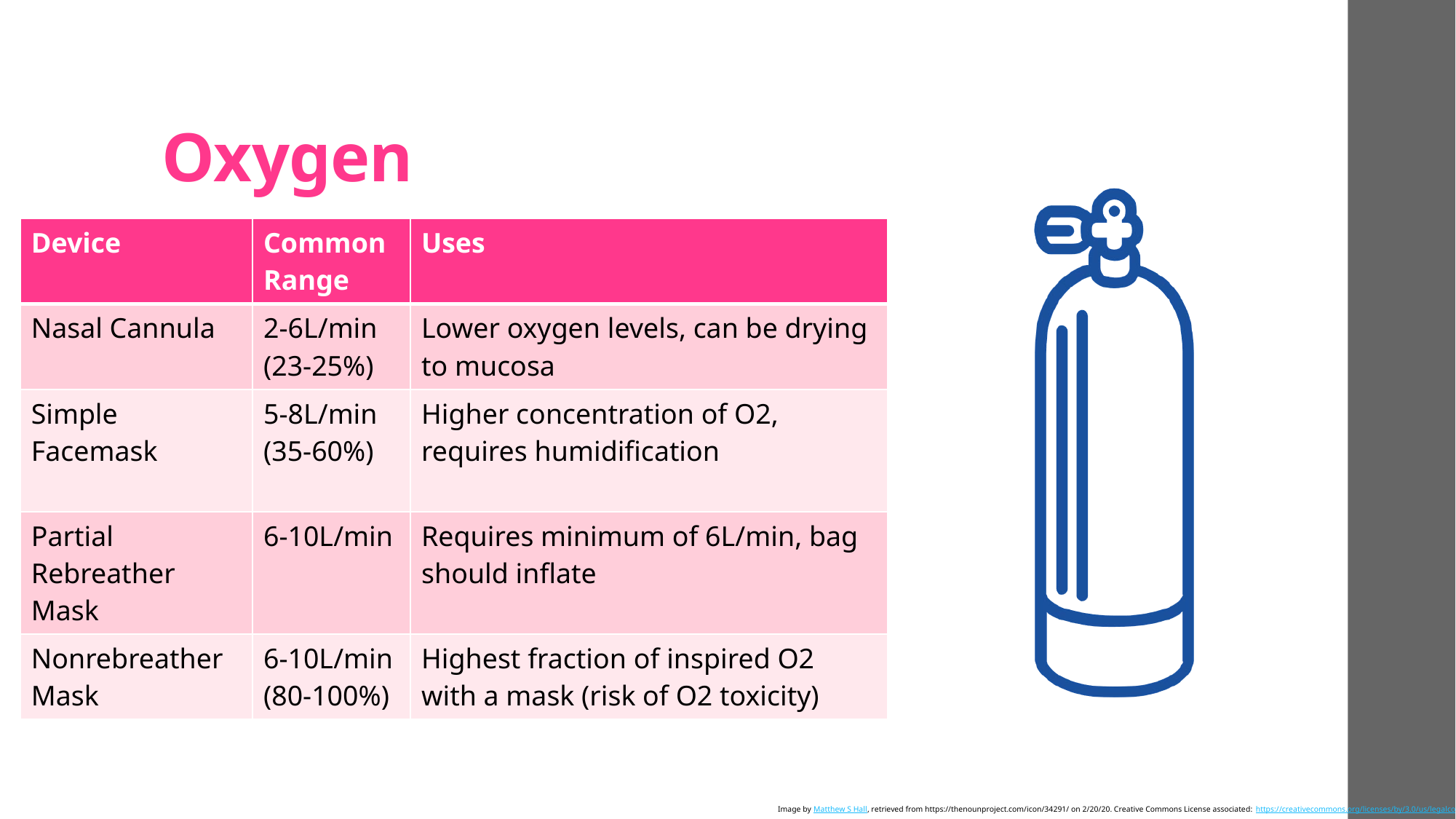

# Oxygen
| Device | Common Range | Uses |
| --- | --- | --- |
| Nasal Cannula | 2-6L/min (23-25%) | Lower oxygen levels, can be drying to mucosa |
| Simple Facemask | 5-8L/min (35-60%) | Higher concentration of O2, requires humidification |
| Partial Rebreather Mask | 6-10L/min | Requires minimum of 6L/min, bag should inflate |
| Nonrebreather Mask | 6-10L/min (80-100%) | Highest fraction of inspired O2 with a mask (risk of O2 toxicity) |
Image by Matthew S Hall, retrieved from https://thenounproject.com/icon/34291/ on 2/20/20. Creative Commons License associated: https://creativecommons.org/licenses/by/3.0/us/legalcode.

## Slide 12
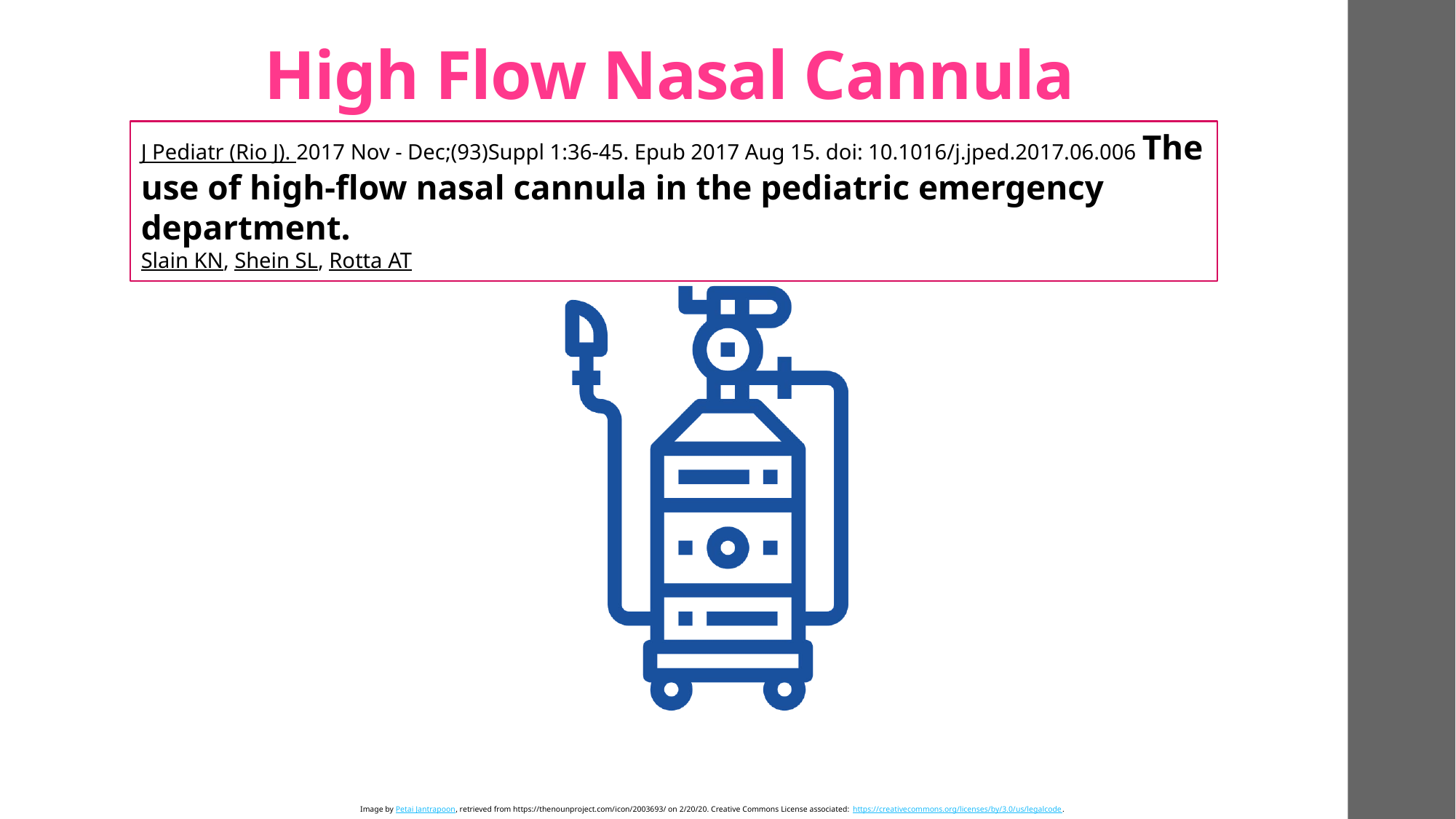

# High Flow Nasal Cannula
J Pediatr (Rio J). 2017 Nov - Dec;(93)Suppl 1:36-45. Epub 2017 Aug 15. doi: 10.1016/j.jped.2017.06.006 The use of high-flow nasal cannula in the pediatric emergency department.
Slain KN, Shein SL, Rotta AT
Image by Petai Jantrapoon, retrieved from https://thenounproject.com/icon/2003693/ on 2/20/20. Creative Commons License associated: https://creativecommons.org/licenses/by/3.0/us/legalcode.

## Slide 13
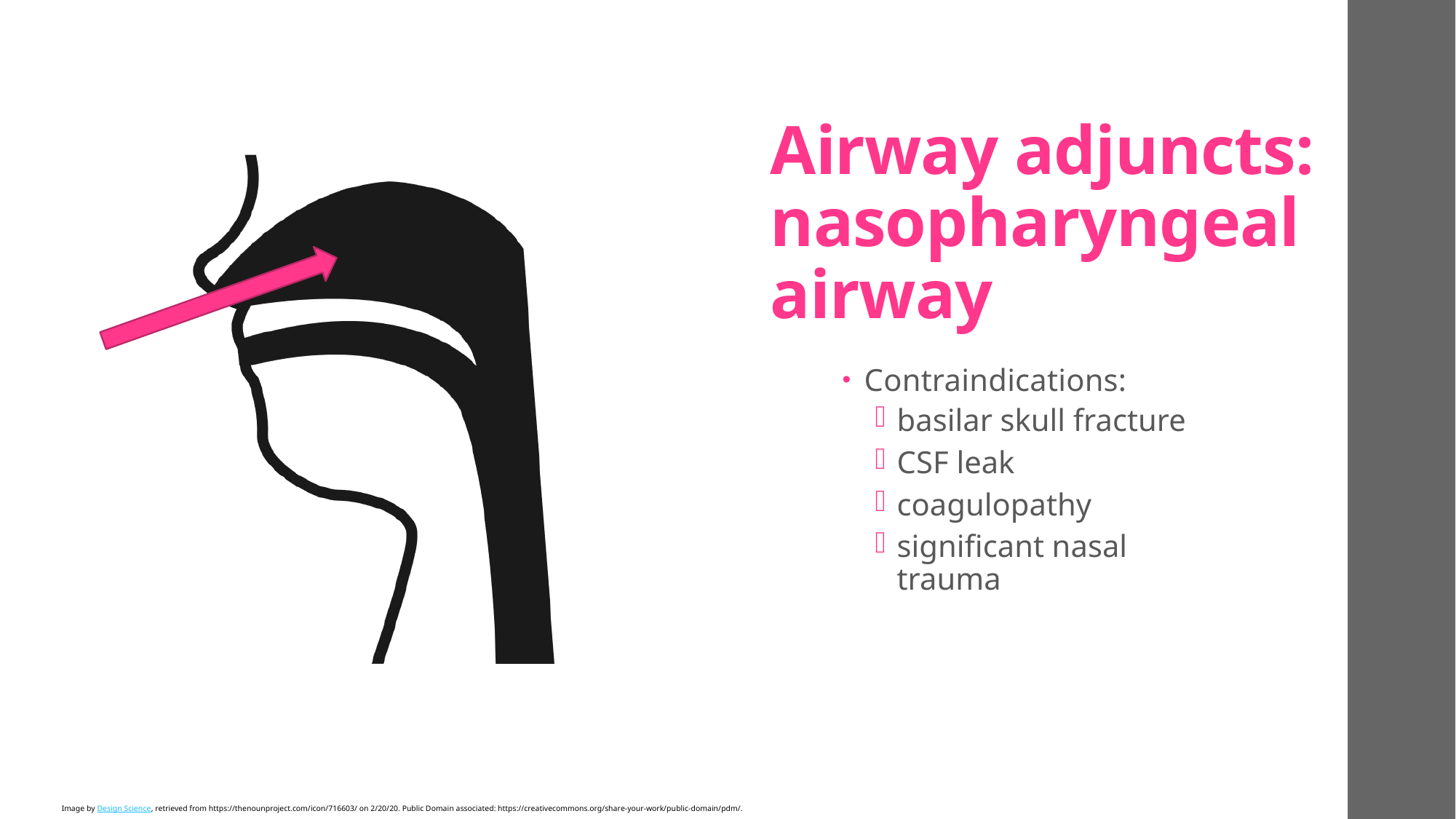

# Airway adjuncts: nasopharyngeal airway
Contraindications:
basilar skull fracture
CSF leak
coagulopathy
significant nasal trauma
Image by Design Science, retrieved from https://thenounproject.com/icon/716603/ on 2/20/20. Public Domain associated: https://creativecommons.org/share-your-work/public-domain/pdm/.

## Slide 14
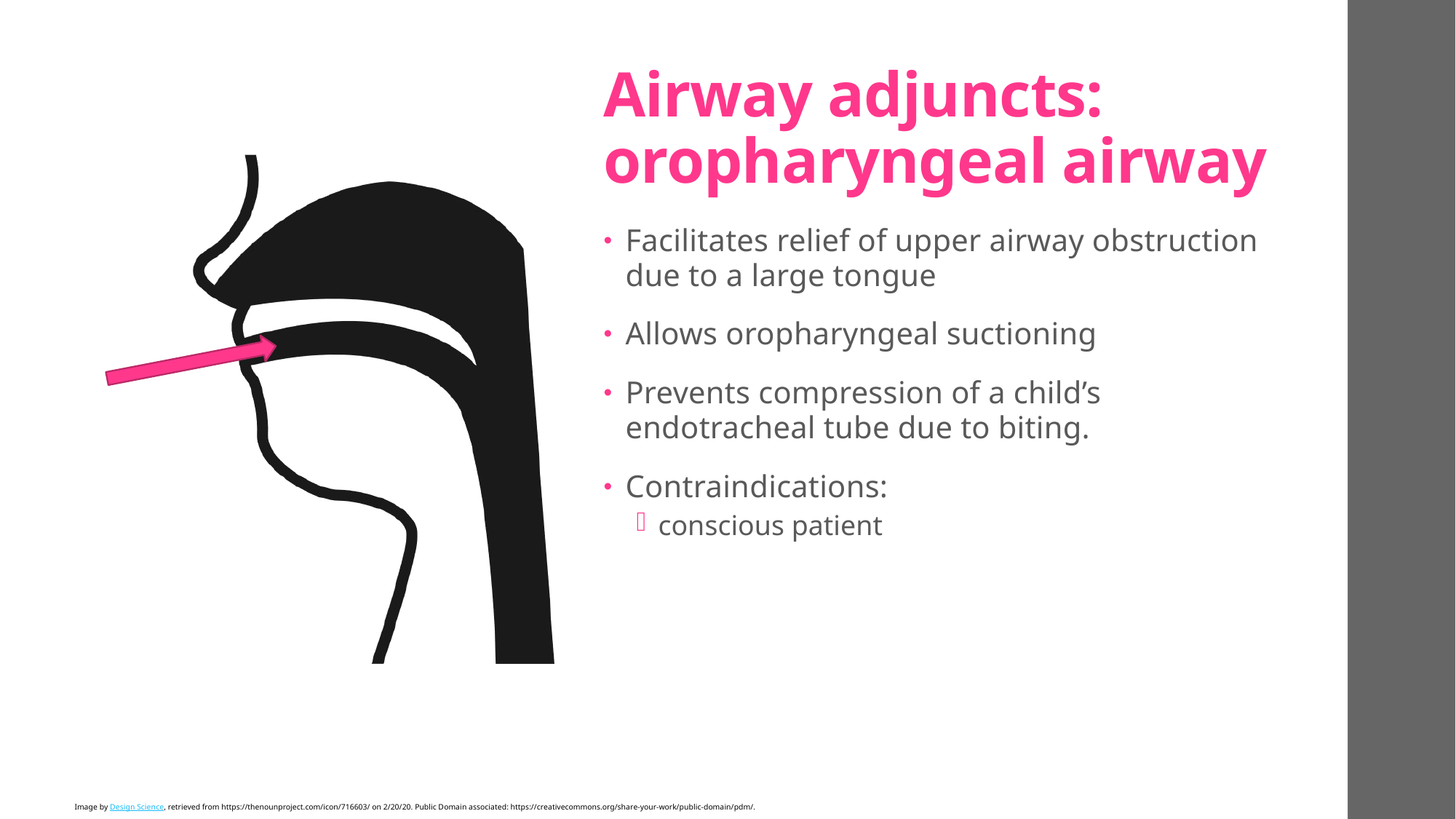

# Airway adjuncts: oropharyngeal airway
Facilitates relief of upper airway obstruction due to a large tongue
Allows oropharyngeal suctioning
Prevents compression of a child’s endotracheal tube due to biting.
Contraindications:
conscious patient
Image by Design Science, retrieved from https://thenounproject.com/icon/716603/ on 2/20/20. Public Domain associated: https://creativecommons.org/share-your-work/public-domain/pdm/.

## Slide 15
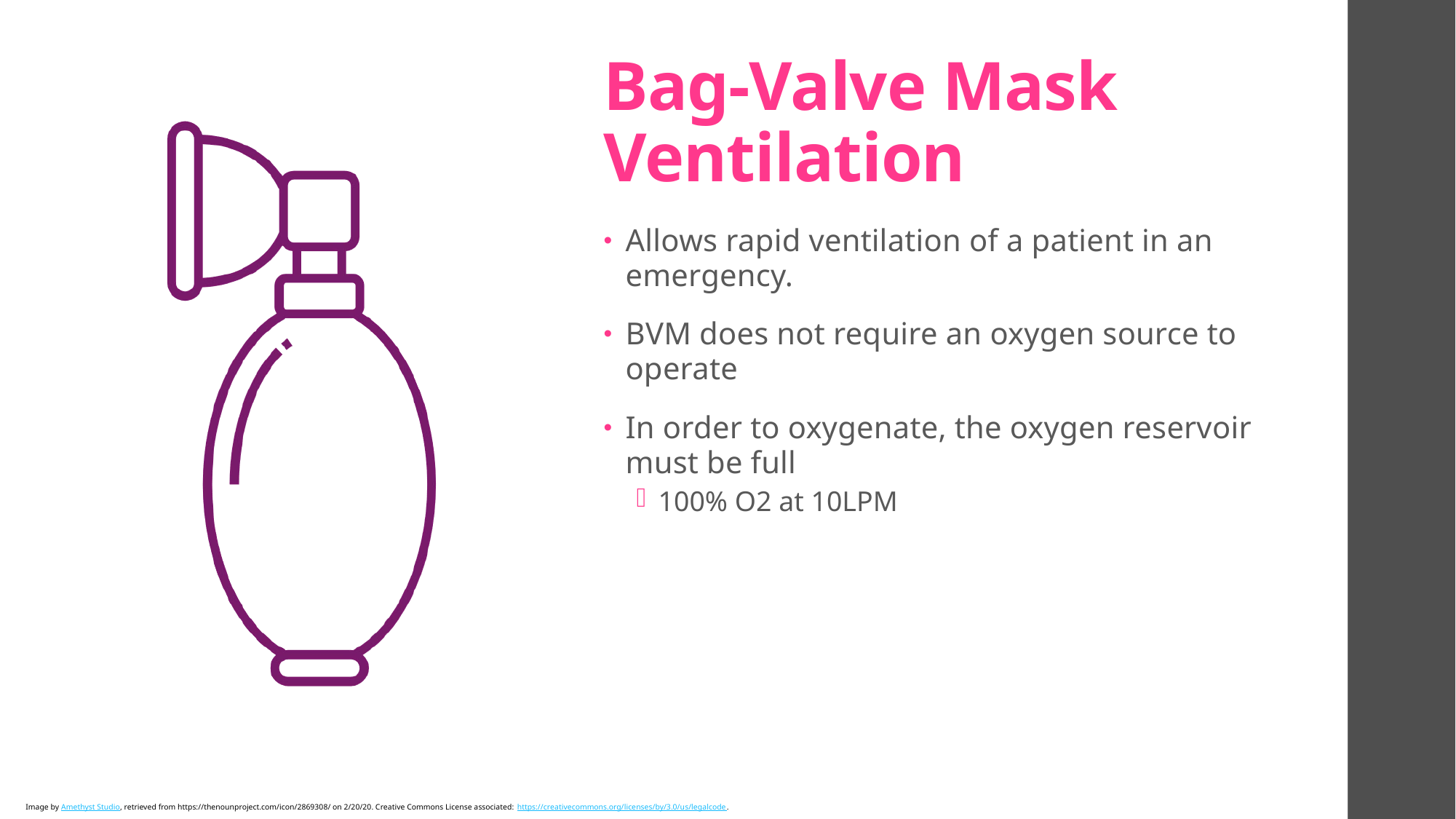

# Bag-Valve Mask Ventilation
Allows rapid ventilation of a patient in an emergency.
BVM does not require an oxygen source to operate
In order to oxygenate, the oxygen reservoir must be full
100% O2 at 10LPM
Image by Amethyst Studio, retrieved from https://thenounproject.com/icon/2869308/ on 2/20/20. Creative Commons License associated: https://creativecommons.org/licenses/by/3.0/us/legalcode.

## Slide 16
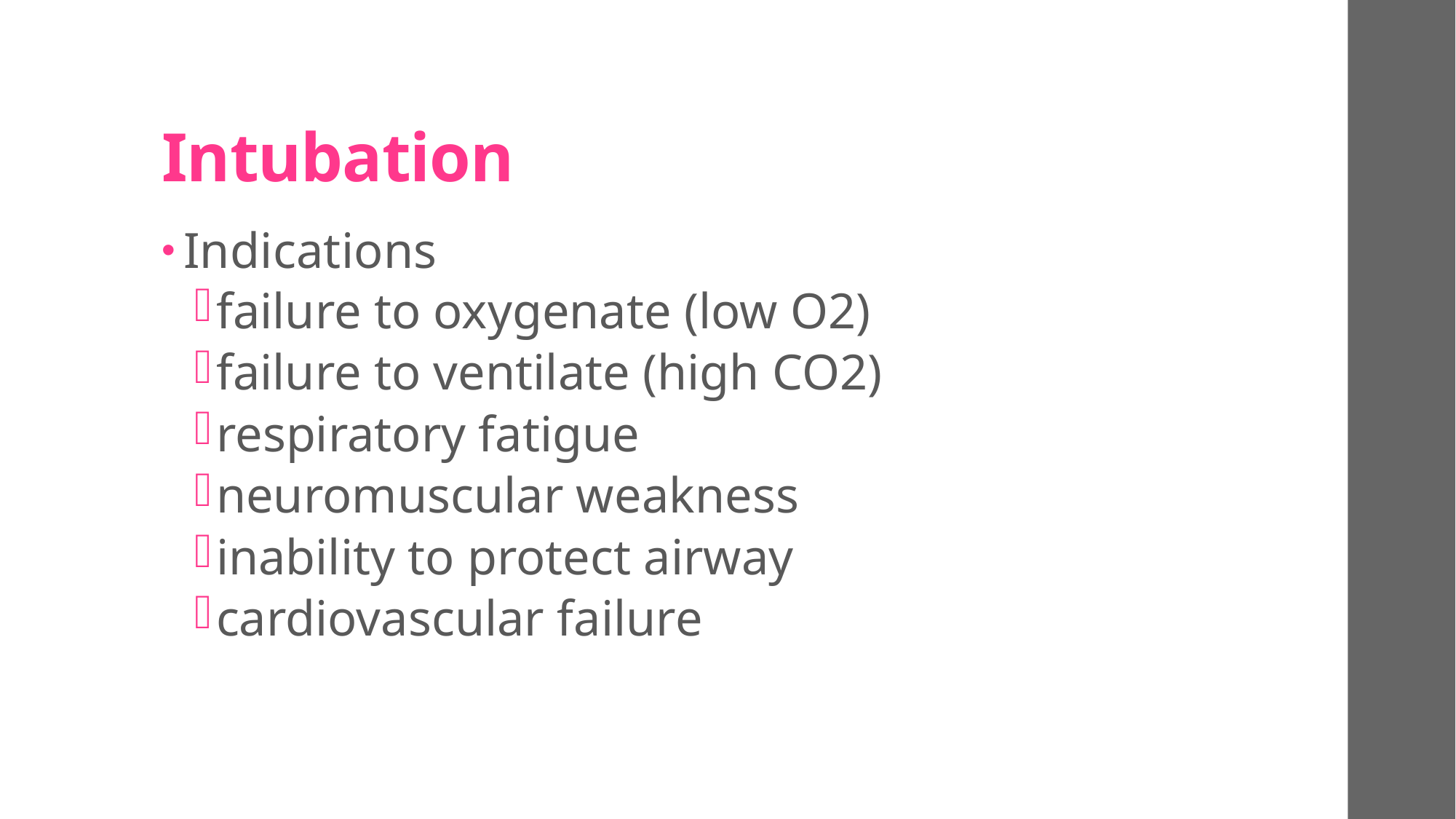

# Intubation
Indications
failure to oxygenate (low O2)
failure to ventilate (high CO2)
respiratory fatigue
neuromuscular weakness
inability to protect airway
cardiovascular failure

## Slide 17
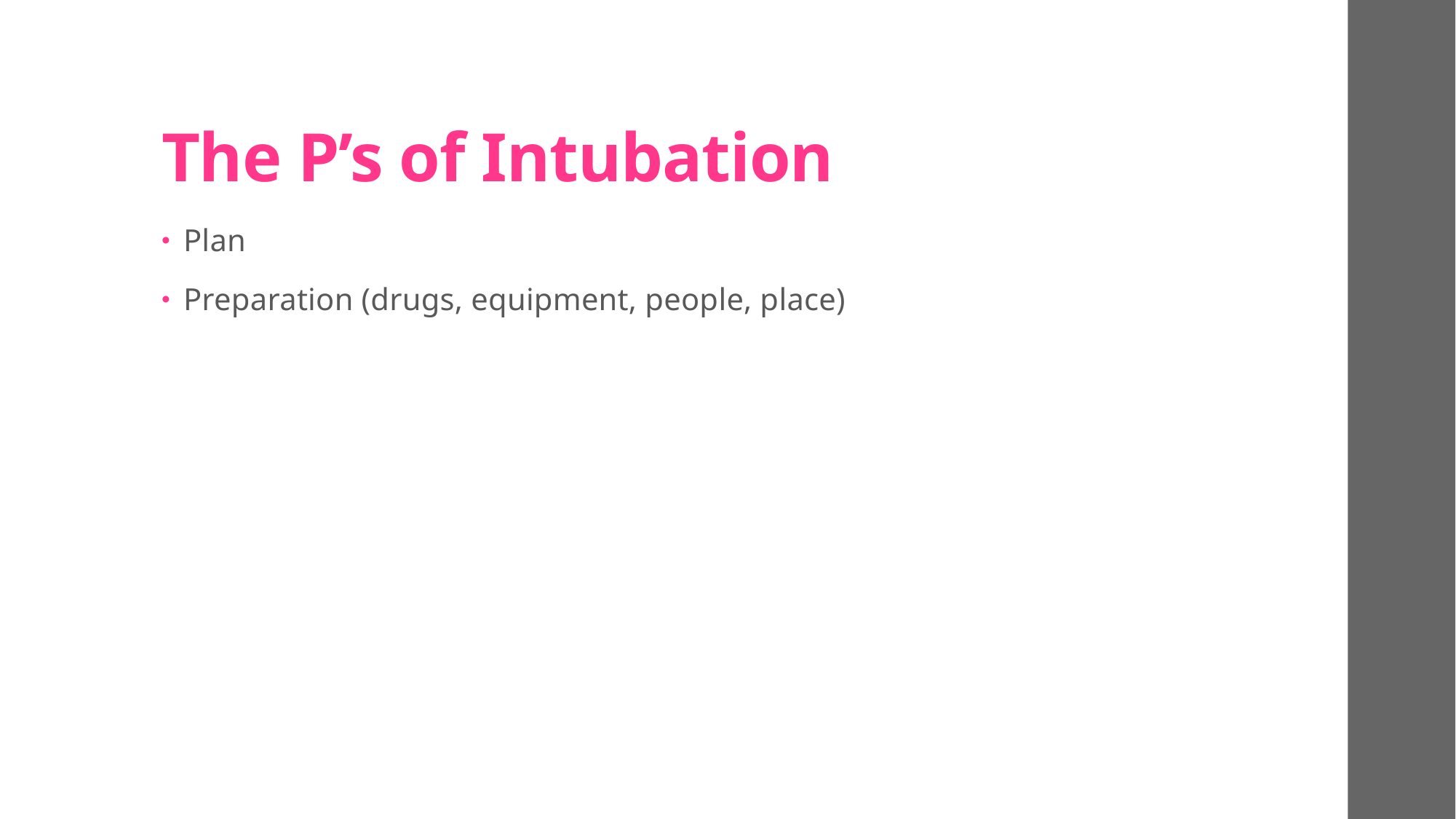

# The P’s of Intubation
Plan
Preparation (drugs, equipment, people, place)

## Slide 18
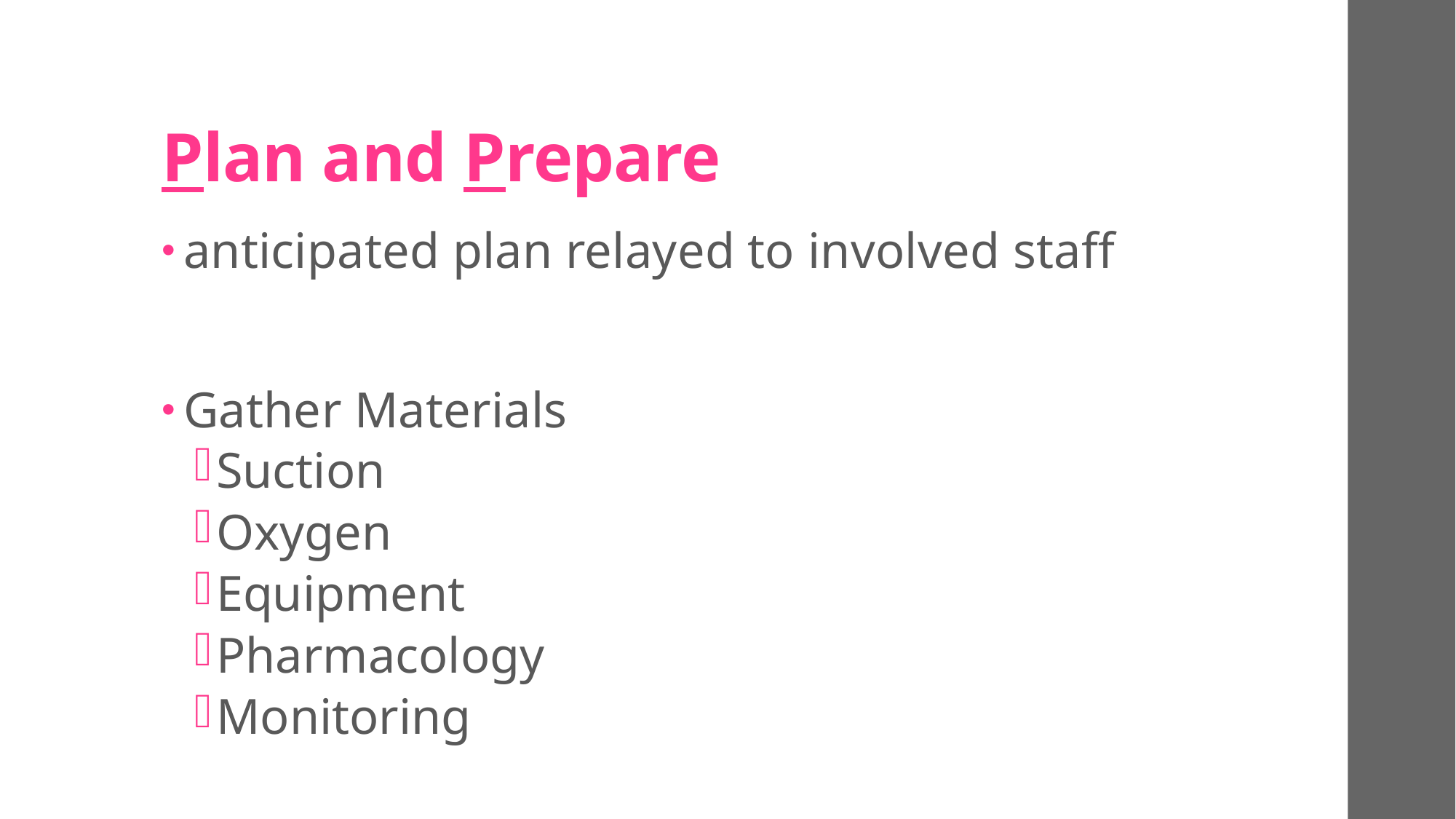

# Plan and Prepare
anticipated plan relayed to involved staff
Gather Materials
Suction
Oxygen
Equipment
Pharmacology
Monitoring

## Slide 19
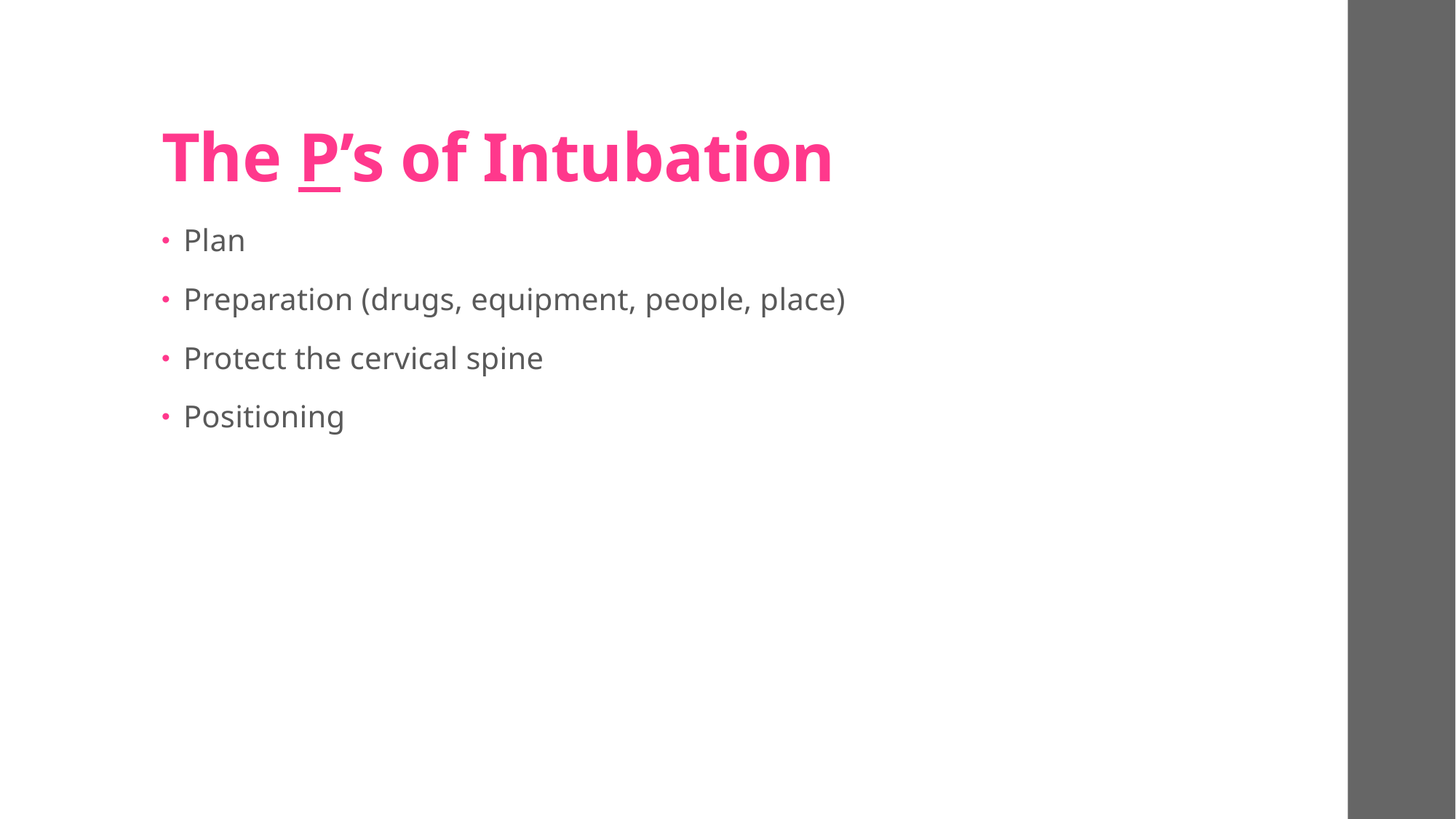

# The P’s of Intubation
Plan
Preparation (drugs, equipment, people, place)
Protect the cervical spine
Positioning

## Slide 20
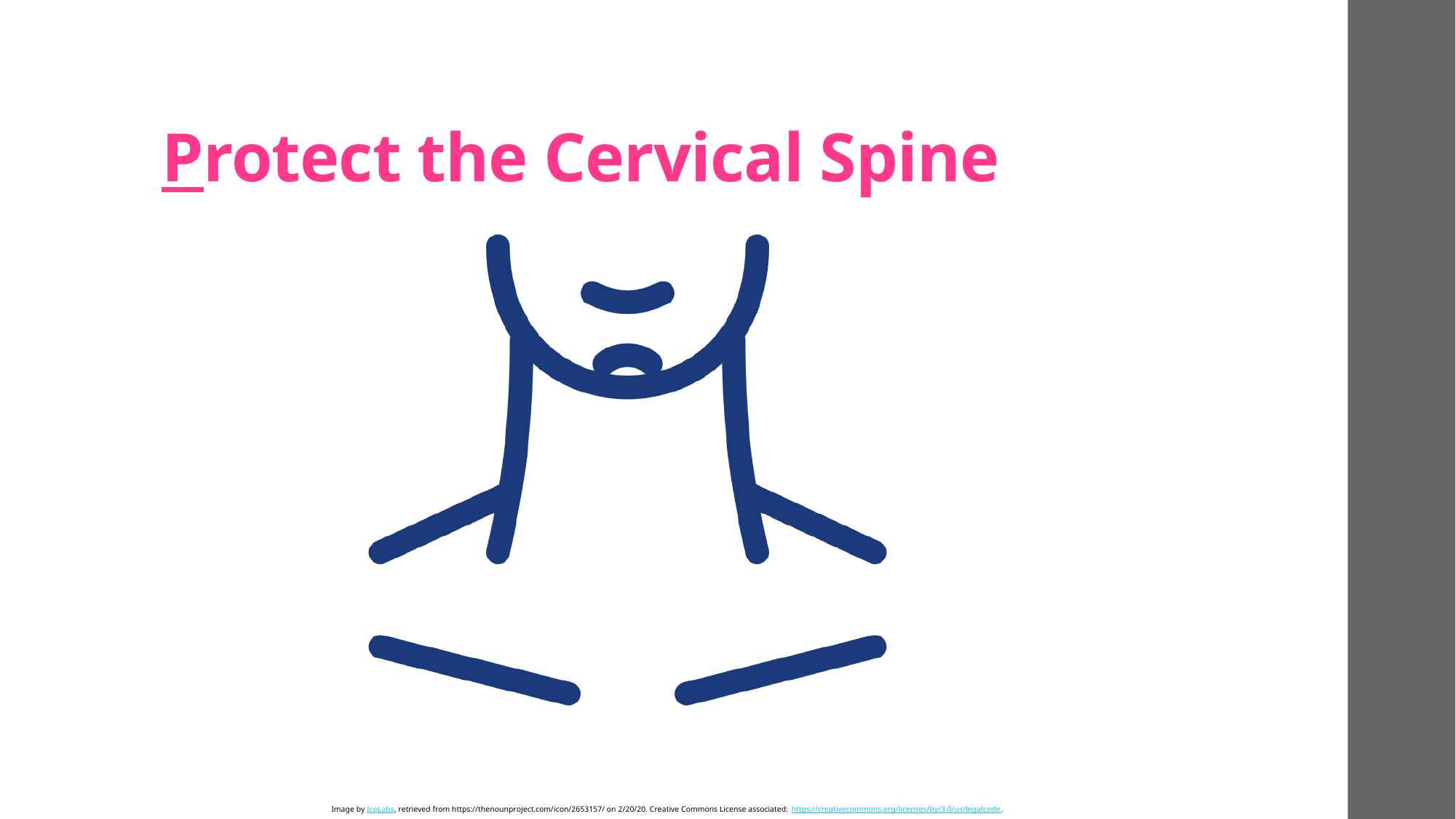

# Protect the Cervical Spine
Image by IcoLabs, retrieved from https://thenounproject.com/icon/2653157/ on 2/20/20. Creative Commons License associated: https://creativecommons.org/licenses/by/3.0/us/legalcode.

## Slide 21
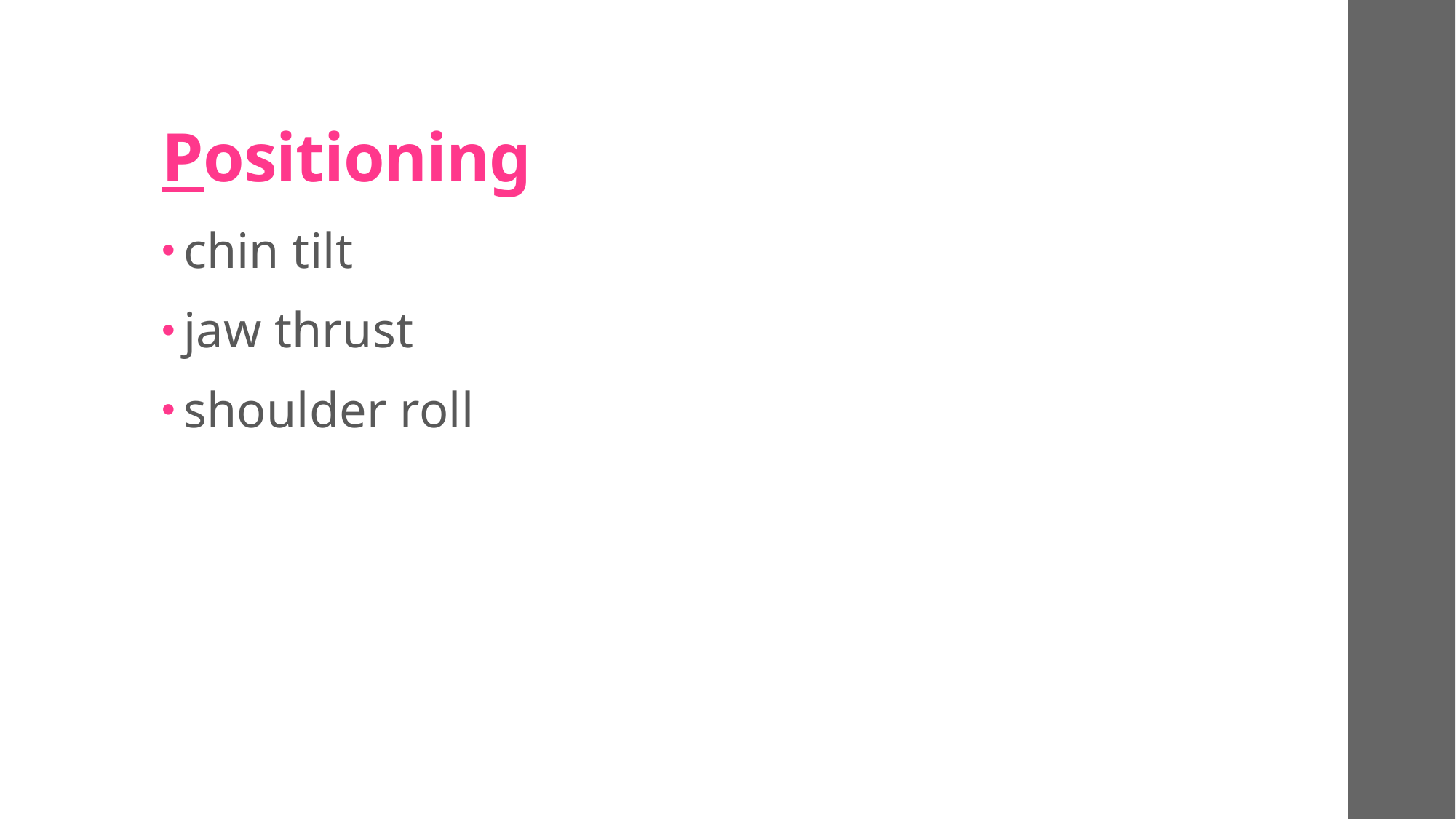

# Positioning
chin tilt
jaw thrust
shoulder roll

## Slide 22
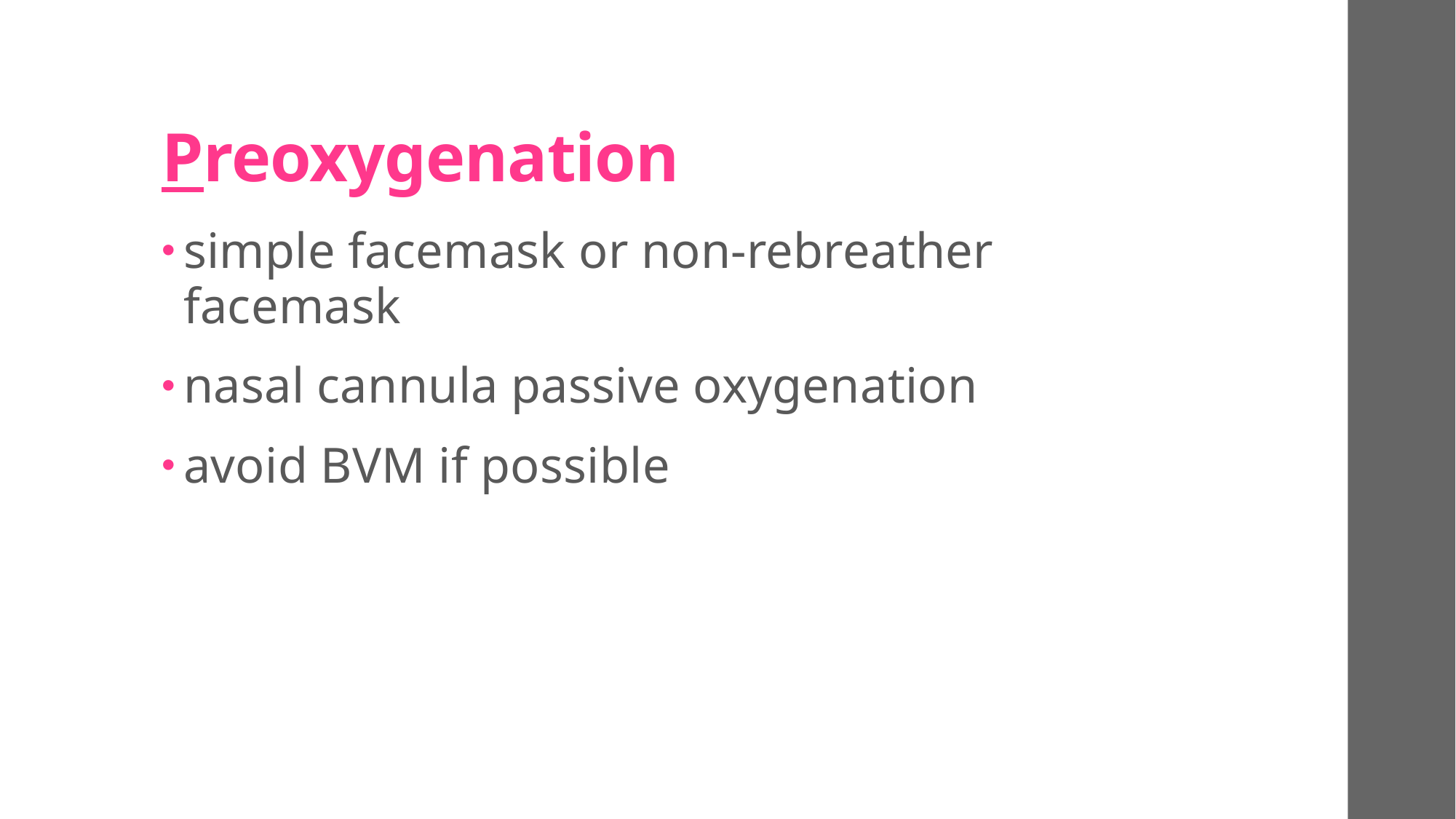

# Preoxygenation
simple facemask or non-rebreather facemask
nasal cannula passive oxygenation
avoid BVM if possible

## Slide 23
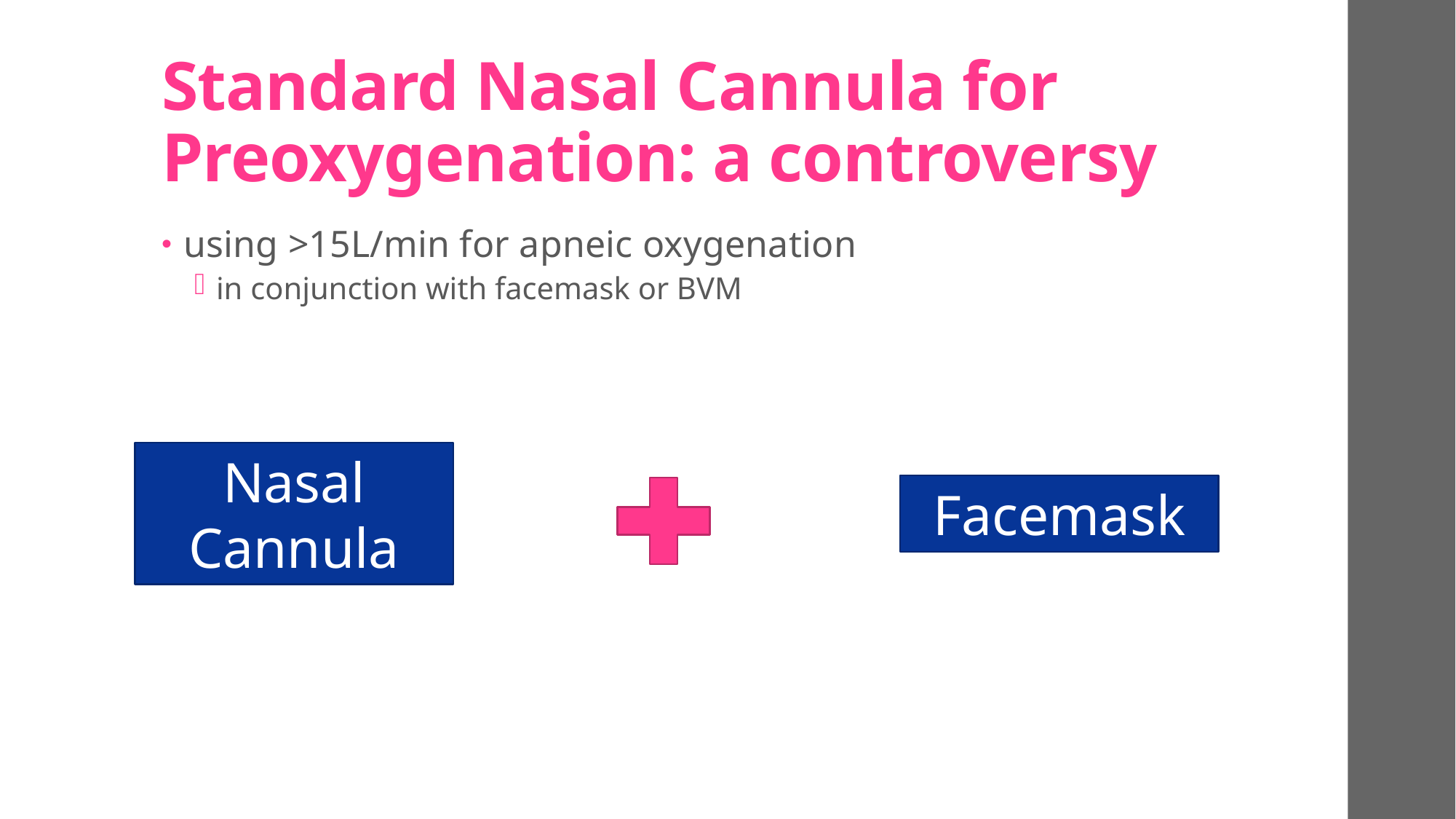

# Standard Nasal Cannula for Preoxygenation: a controversy
using >15L/min for apneic oxygenation
in conjunction with facemask or BVM
Nasal Cannula
Facemask

## Slide 24
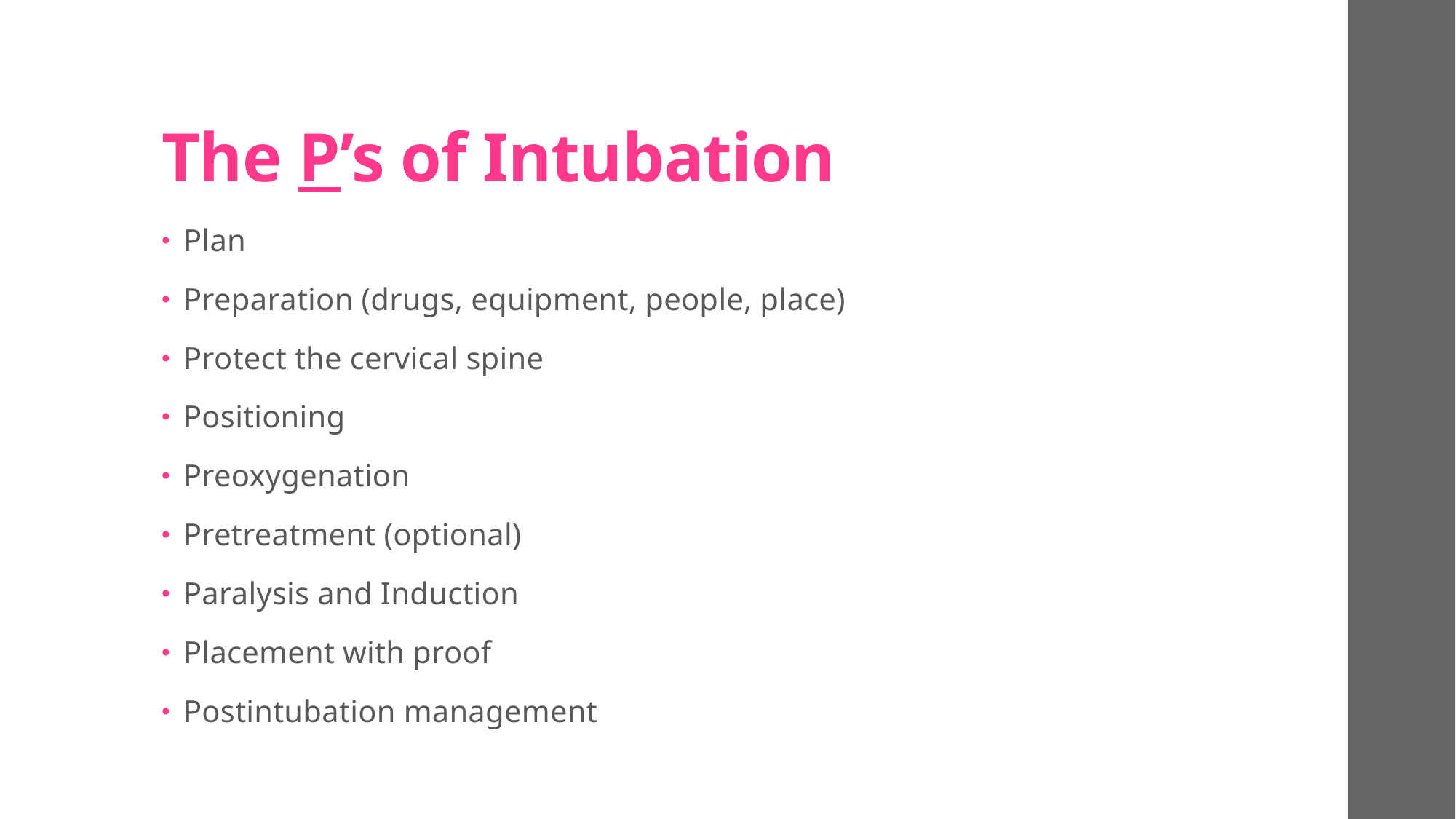

# The P’s of Intubation
Plan
Preparation (drugs, equipment, people, place)
Protect the cervical spine
Positioning
Preoxygenation
Pretreatment (optional)
Paralysis and Induction
Placement with proof
Postintubation management

## Slide 25
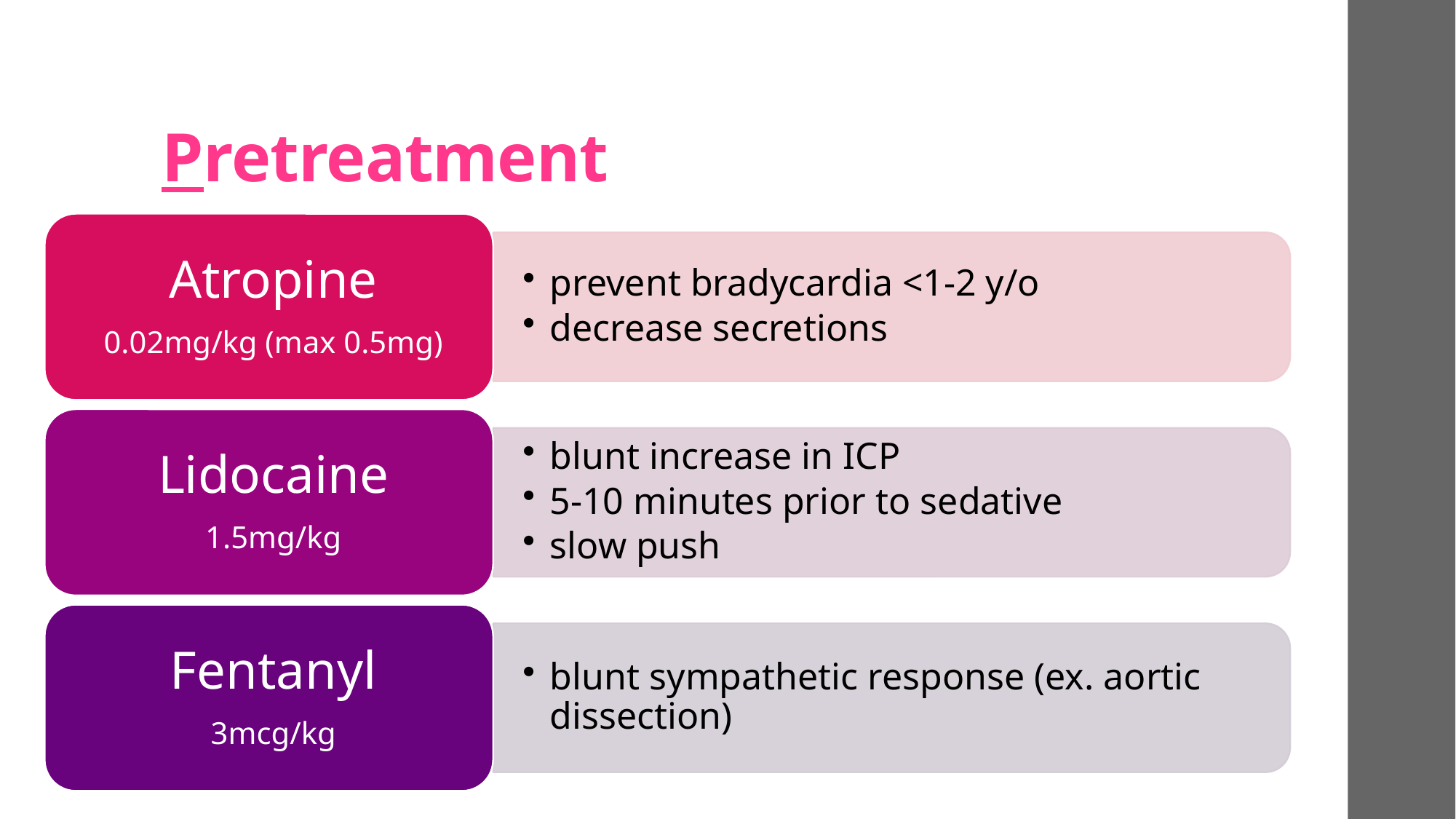

# Pretreatment

## Slide 26
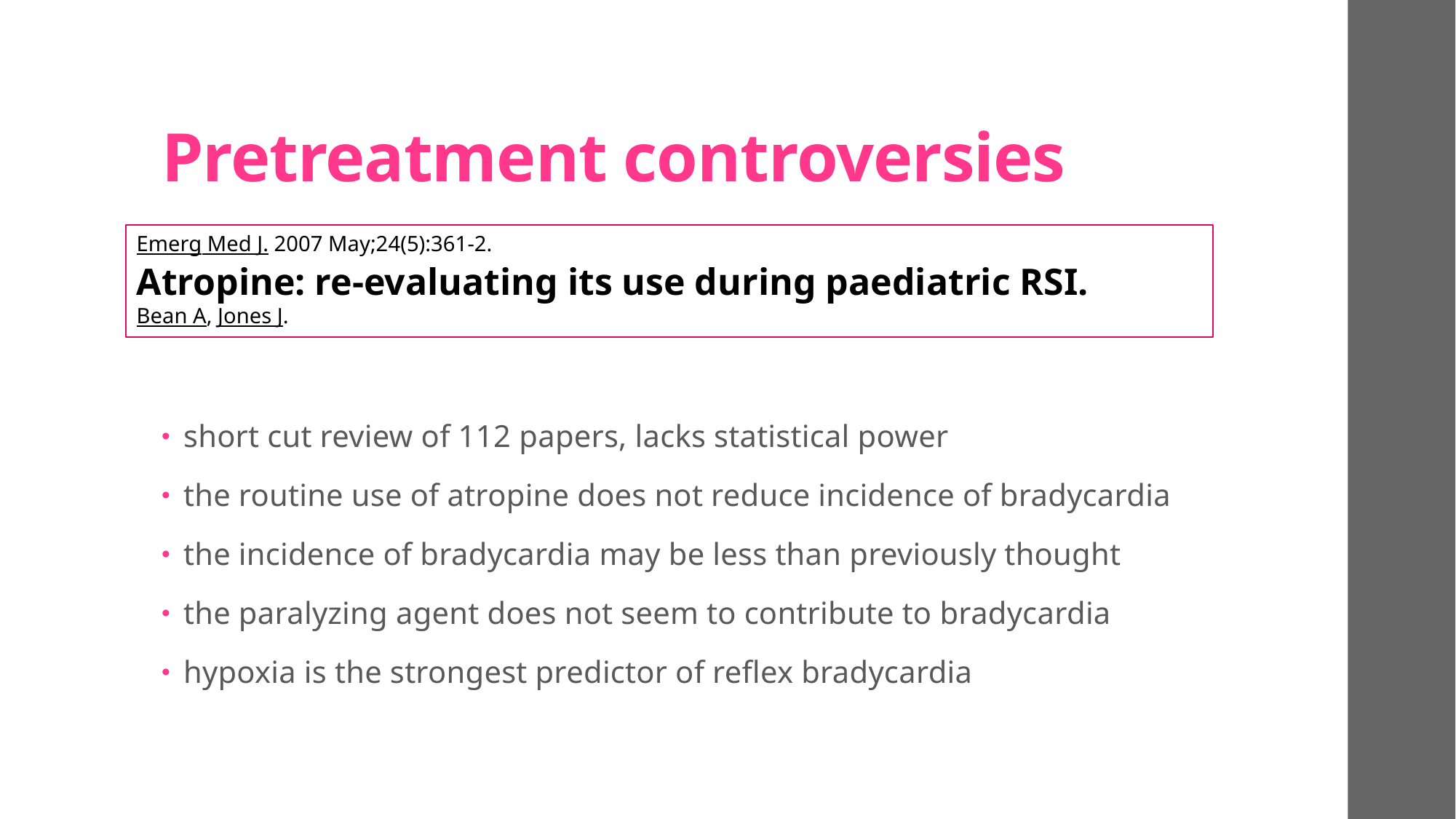

# Pretreatment controversies
Emerg Med J. 2007 May;24(5):361-2.
Atropine: re-evaluating its use during paediatric RSI.
Bean A, Jones J.
short cut review of 112 papers, lacks statistical power
the routine use of atropine does not reduce incidence of bradycardia
the incidence of bradycardia may be less than previously thought
the paralyzing agent does not seem to contribute to bradycardia
hypoxia is the strongest predictor of reflex bradycardia

## Slide 27
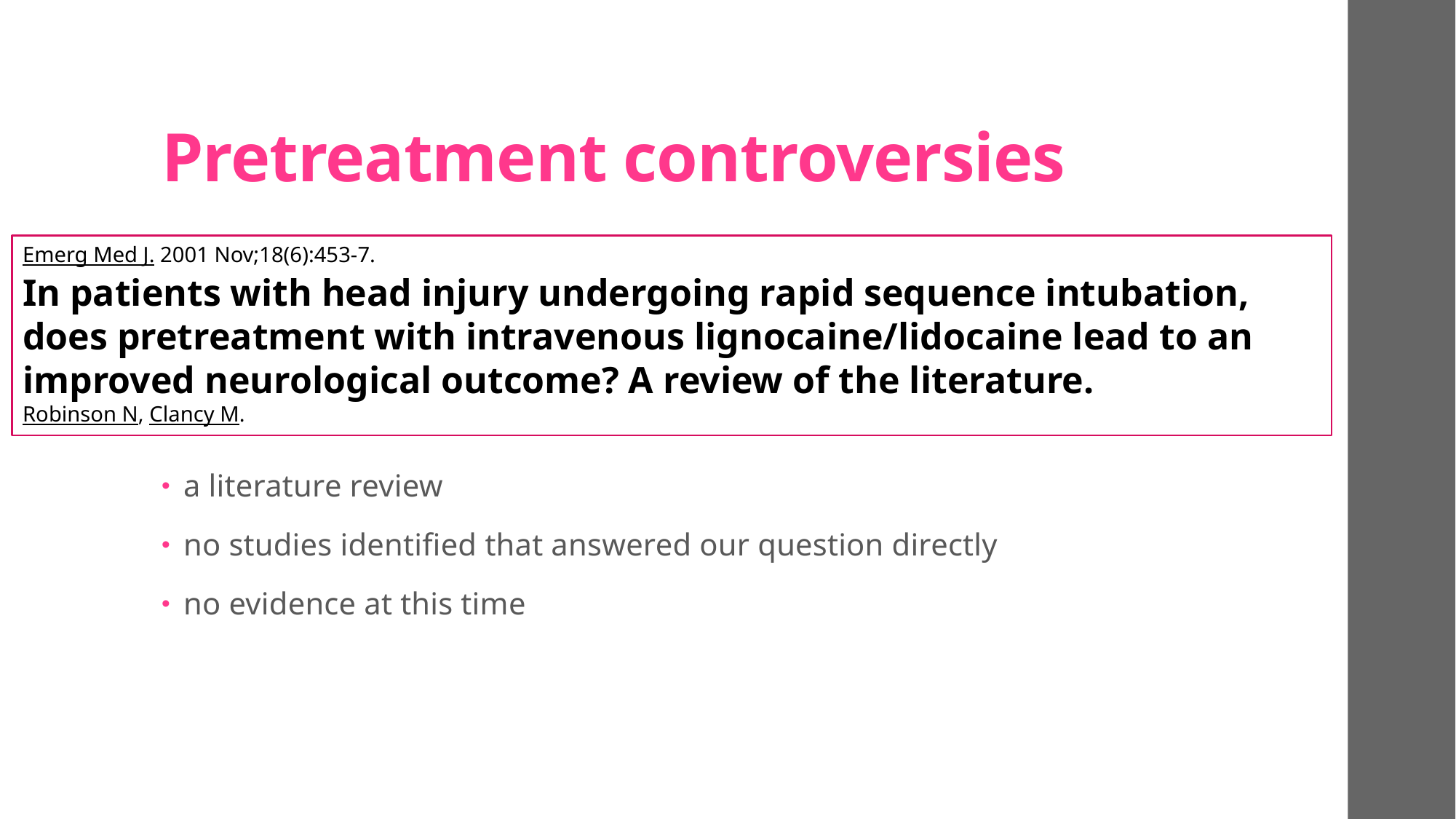

# Pretreatment controversies
Emerg Med J. 2001 Nov;18(6):453-7.
In patients with head injury undergoing rapid sequence intubation, does pretreatment with intravenous lignocaine/lidocaine lead to an improved neurological outcome? A review of the literature.
Robinson N, Clancy M.
a literature review
no studies identified that answered our question directly
no evidence at this time

## Slide 28
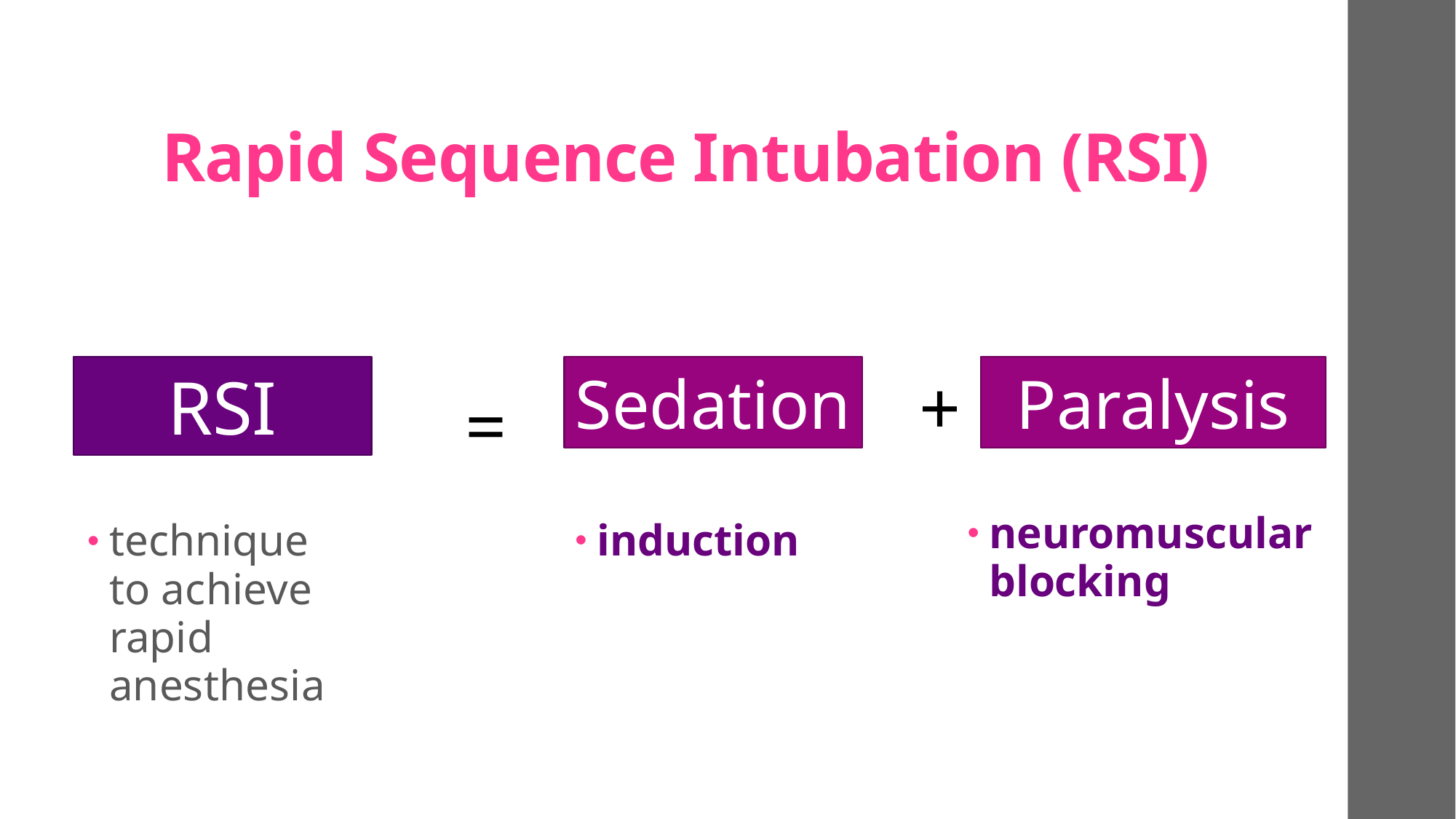

# Rapid Sequence Intubation (RSI)
Paralysis
RSI
Sedation
+
=
neuromuscular blocking
technique to achieve rapid anesthesia
induction

## Slide 29
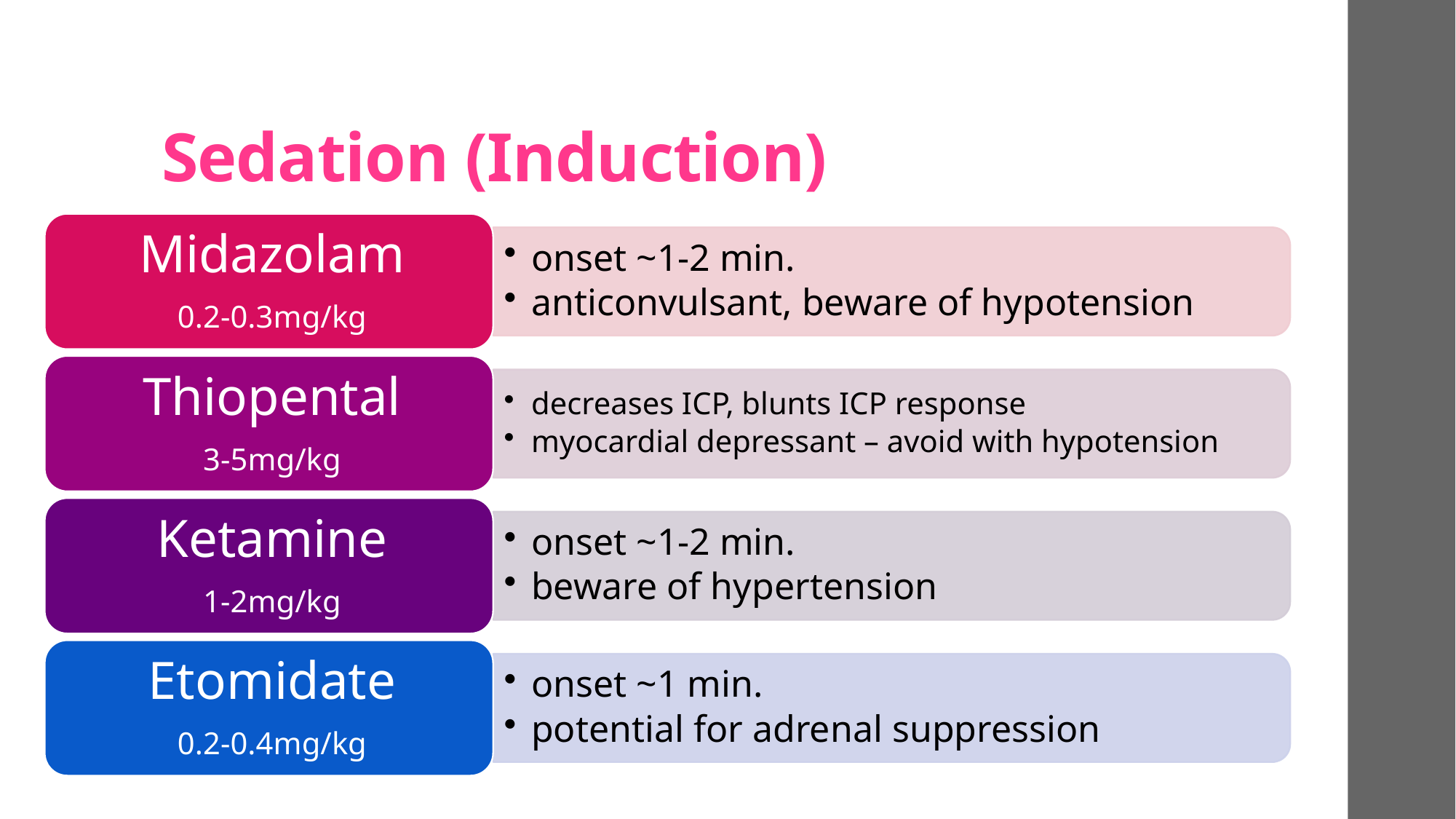

# Sedation (Induction)

## Slide 30
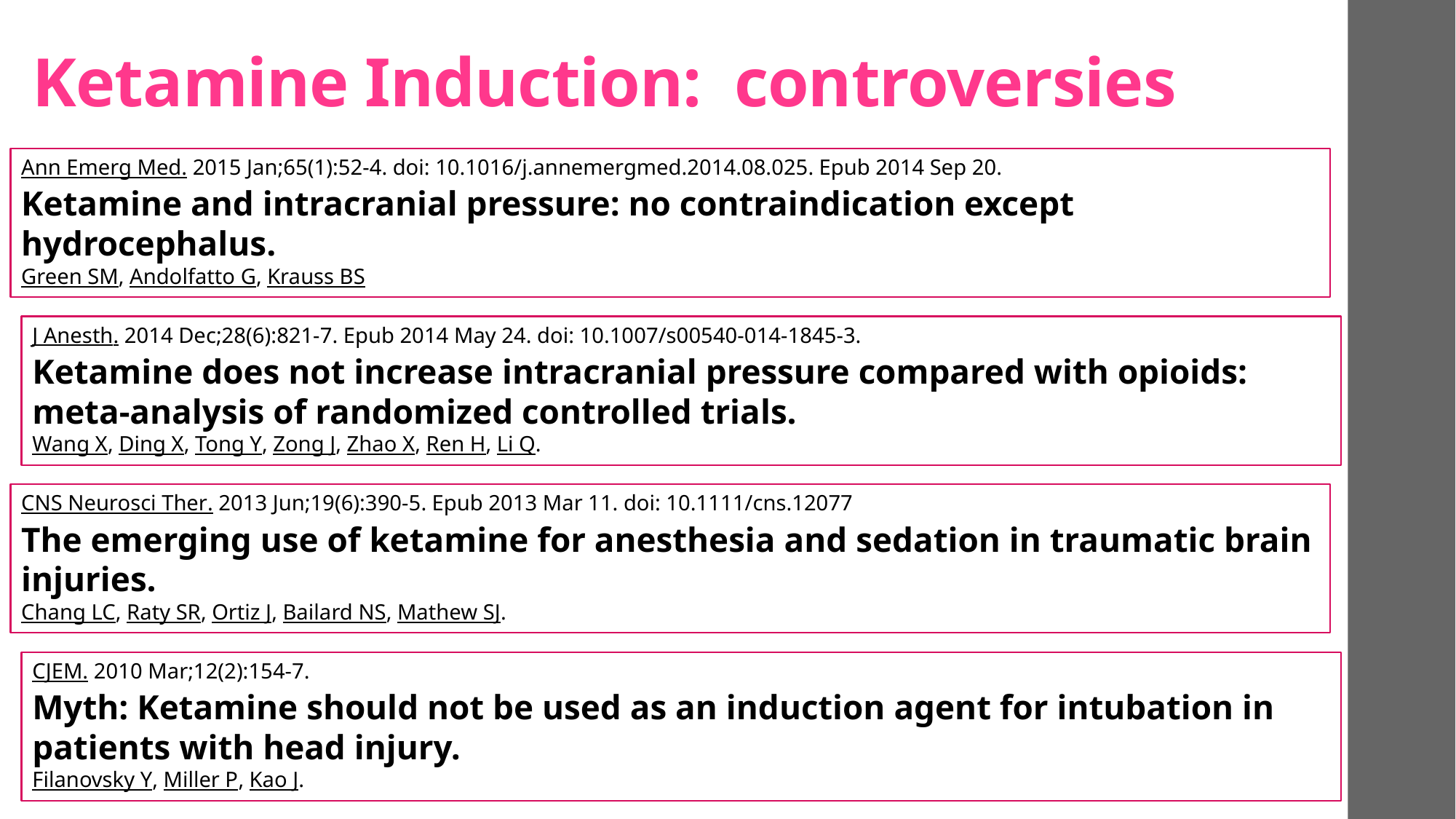

# Ketamine Induction: controversies
Ann Emerg Med. 2015 Jan;65(1):52-4. doi: 10.1016/j.annemergmed.2014.08.025. Epub 2014 Sep 20.
Ketamine and intracranial pressure: no contraindication except hydrocephalus.
Green SM, Andolfatto G, Krauss BS
J Anesth. 2014 Dec;28(6):821-7. Epub 2014 May 24. doi: 10.1007/s00540-014-1845-3.
Ketamine does not increase intracranial pressure compared with opioids: meta-analysis of randomized controlled trials.
Wang X, Ding X, Tong Y, Zong J, Zhao X, Ren H, Li Q.
CNS Neurosci Ther. 2013 Jun;19(6):390-5. Epub 2013 Mar 11. doi: 10.1111/cns.12077
The emerging use of ketamine for anesthesia and sedation in traumatic brain injuries.
Chang LC, Raty SR, Ortiz J, Bailard NS, Mathew SJ.
CJEM. 2010 Mar;12(2):154-7.
Myth: Ketamine should not be used as an induction agent for intubation in patients with head injury.
Filanovsky Y, Miller P, Kao J.

## Slide 31
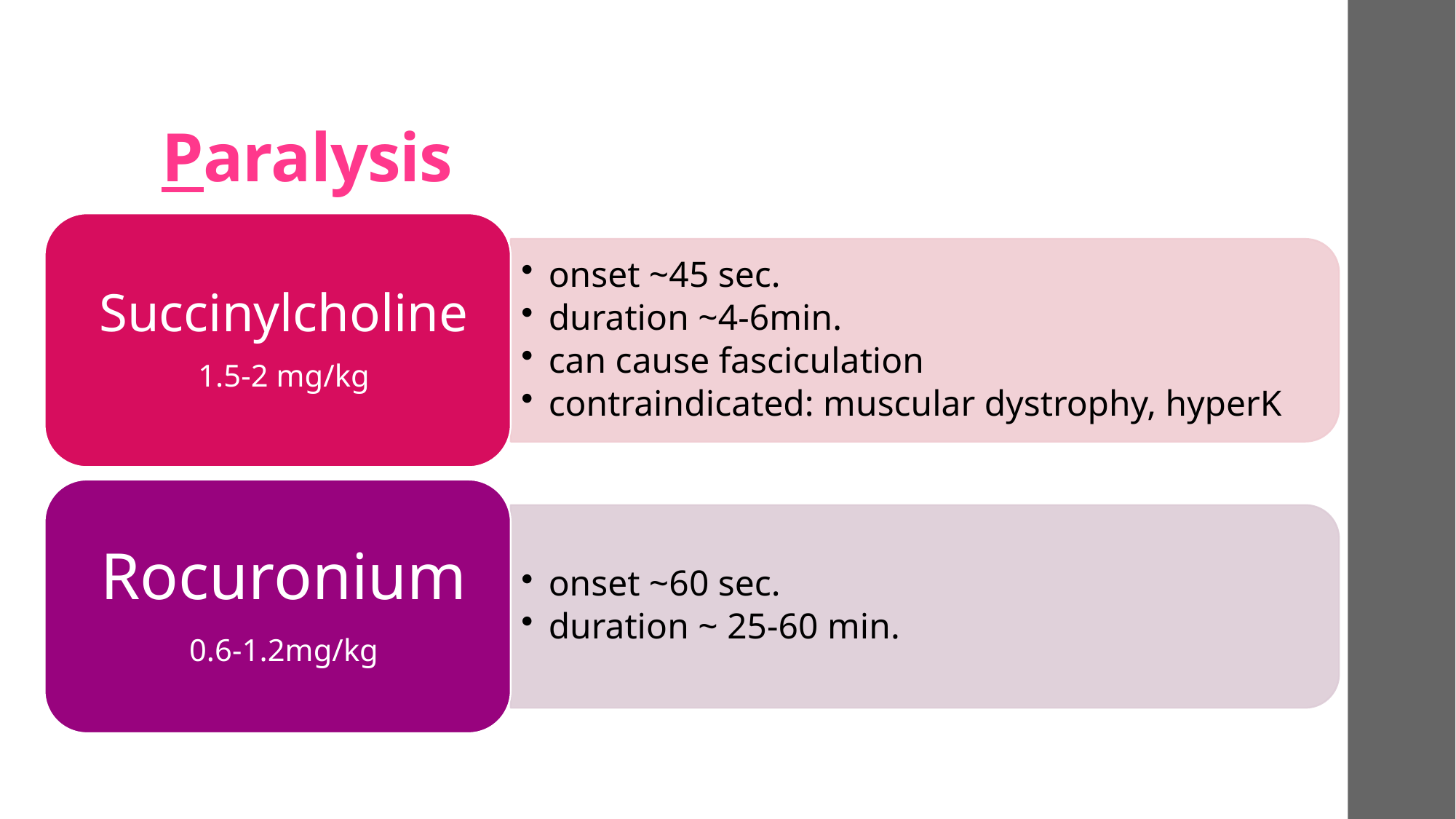

# Paralysis

## Slide 32
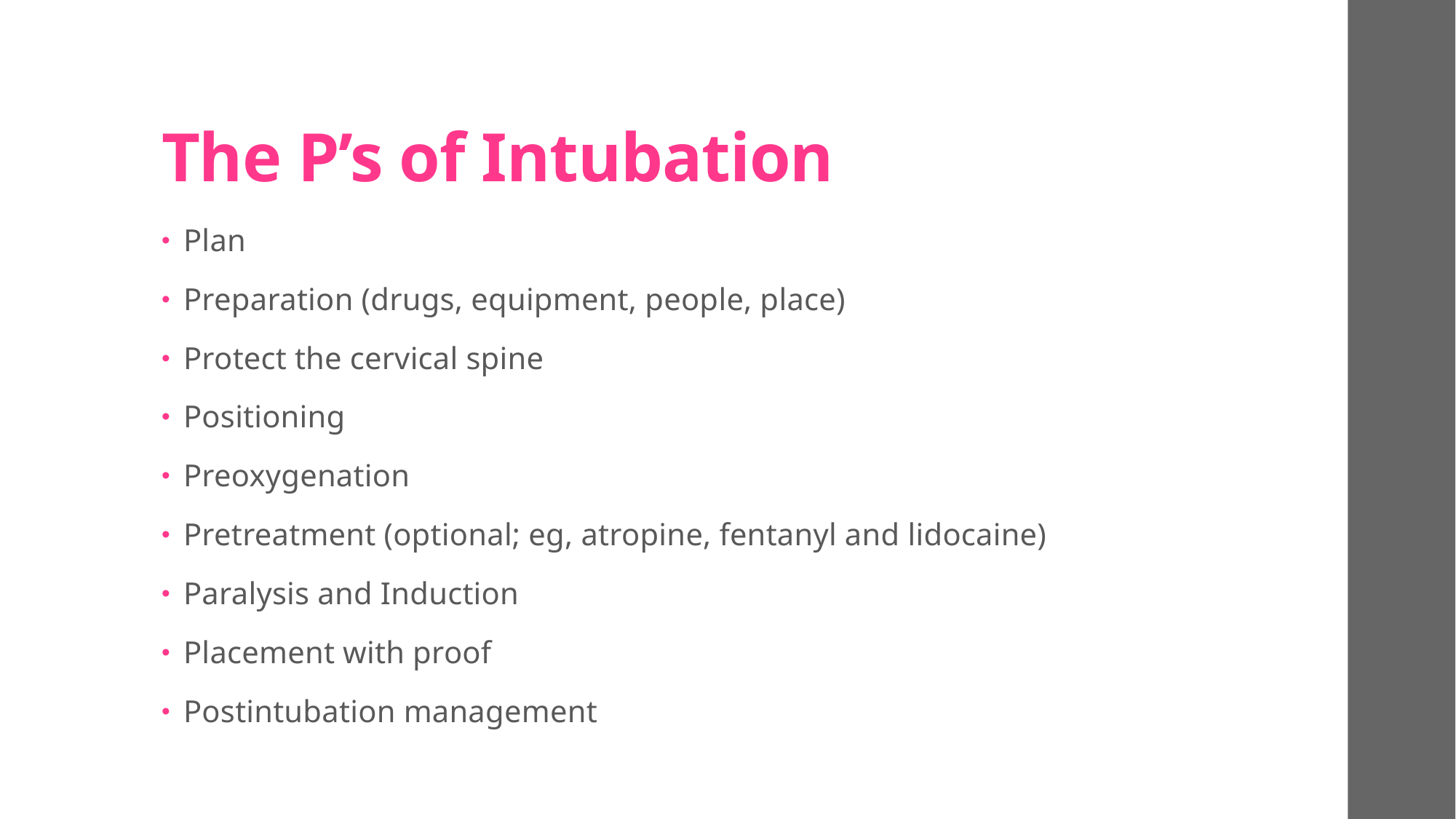

# The P’s of Intubation
Plan
Preparation (drugs, equipment, people, place)
Protect the cervical spine
Positioning
Preoxygenation
Pretreatment (optional; eg, atropine, fentanyl and lidocaine)
Paralysis and Induction
Placement with proof
Postintubation management

## Slide 33
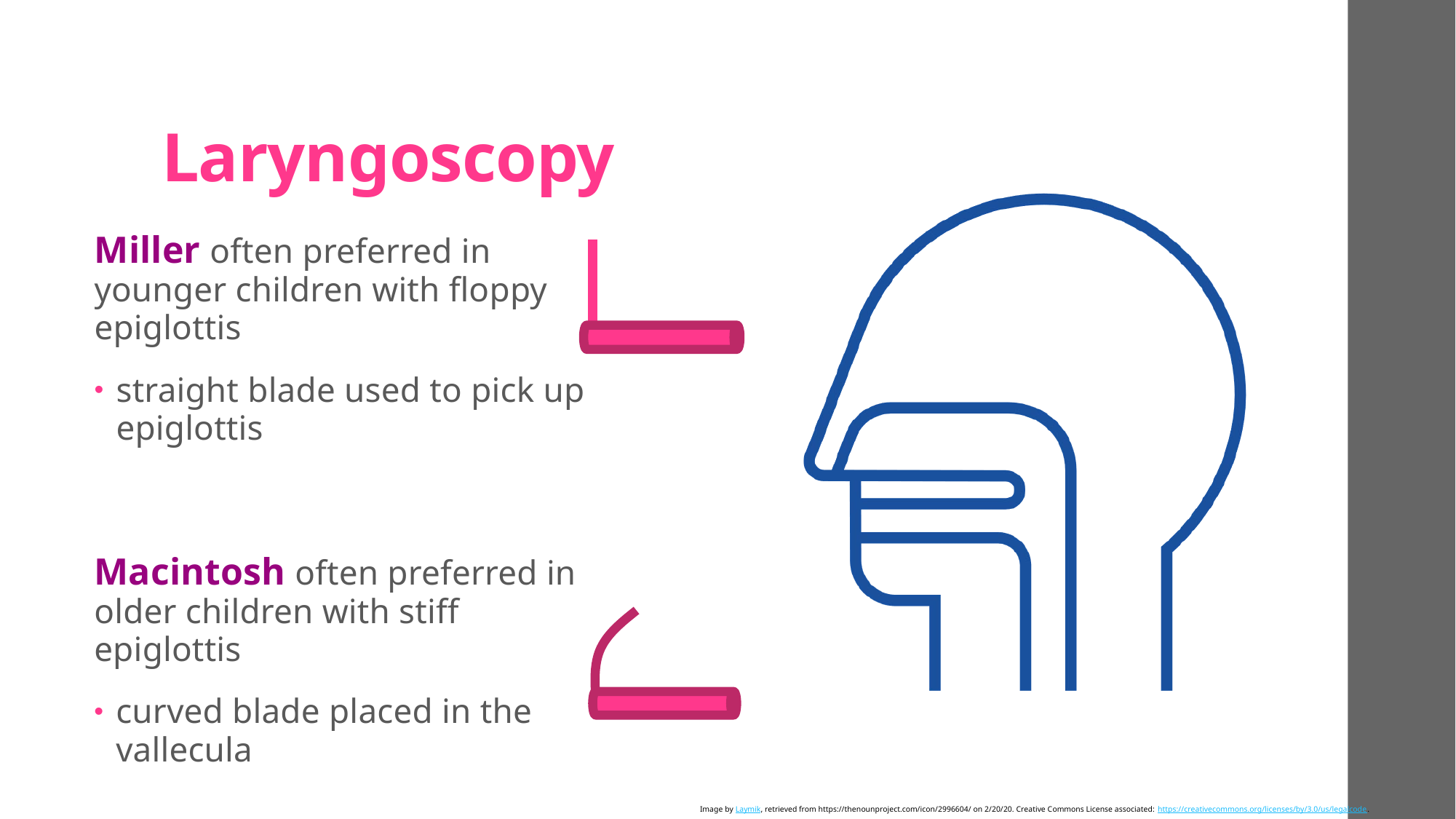

# Laryngoscopy
Miller often preferred in younger children with floppy epiglottis
straight blade used to pick up epiglottis
Macintosh often preferred in older children with stiff epiglottis
curved blade placed in the vallecula
Image by Laymik, retrieved from https://thenounproject.com/icon/2996604/ on 2/20/20. Creative Commons License associated: https://creativecommons.org/licenses/by/3.0/us/legalcode.

## Slide 34
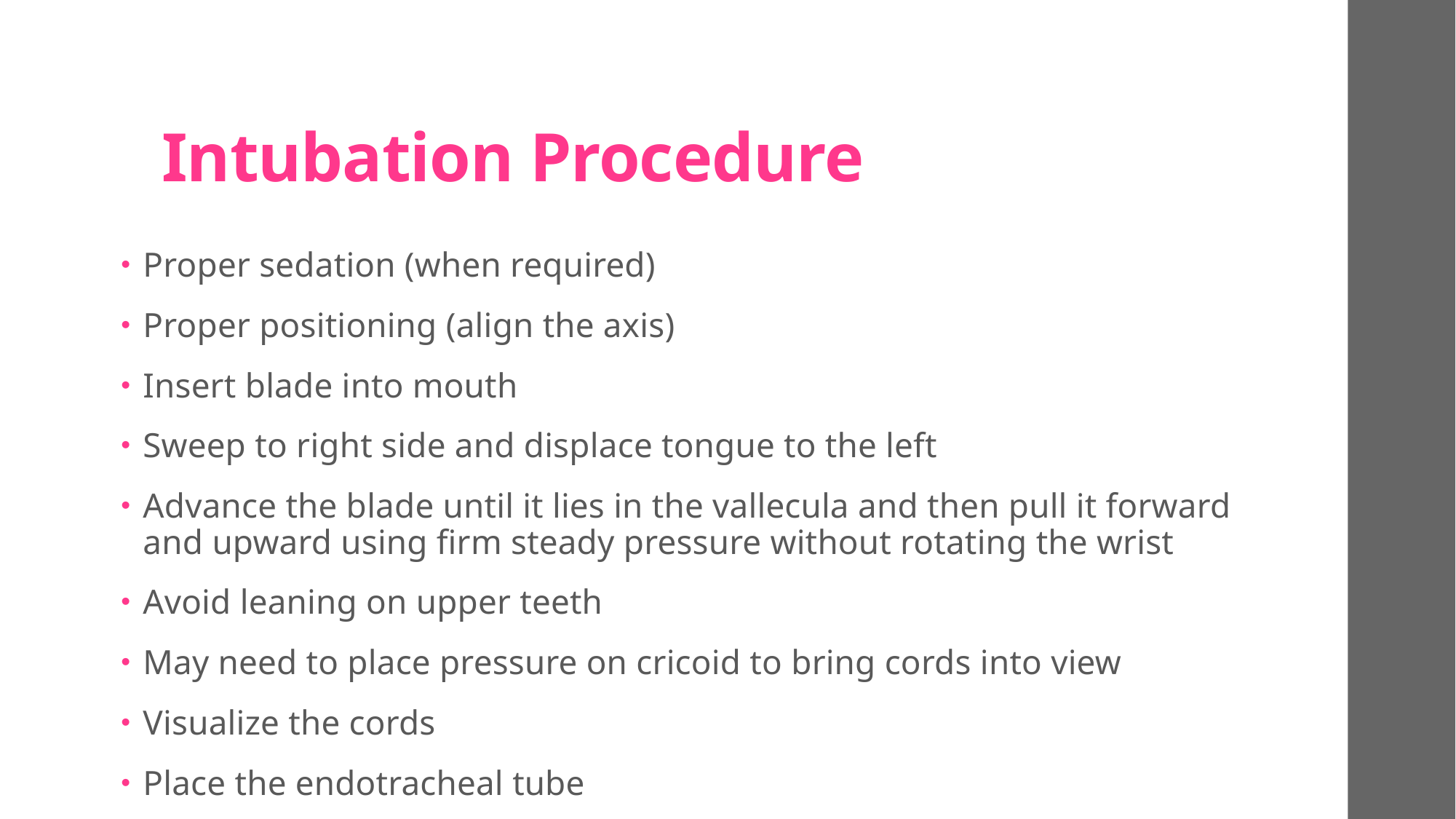

# Intubation Procedure
Proper sedation (when required)
Proper positioning (align the axis)
Insert blade into mouth
Sweep to right side and displace tongue to the left
Advance the blade until it lies in the vallecula and then pull it forward and upward using firm steady pressure without rotating the wrist
Avoid leaning on upper teeth
May need to place pressure on cricoid to bring cords into view
Visualize the cords
Place the endotracheal tube

## Slide 35
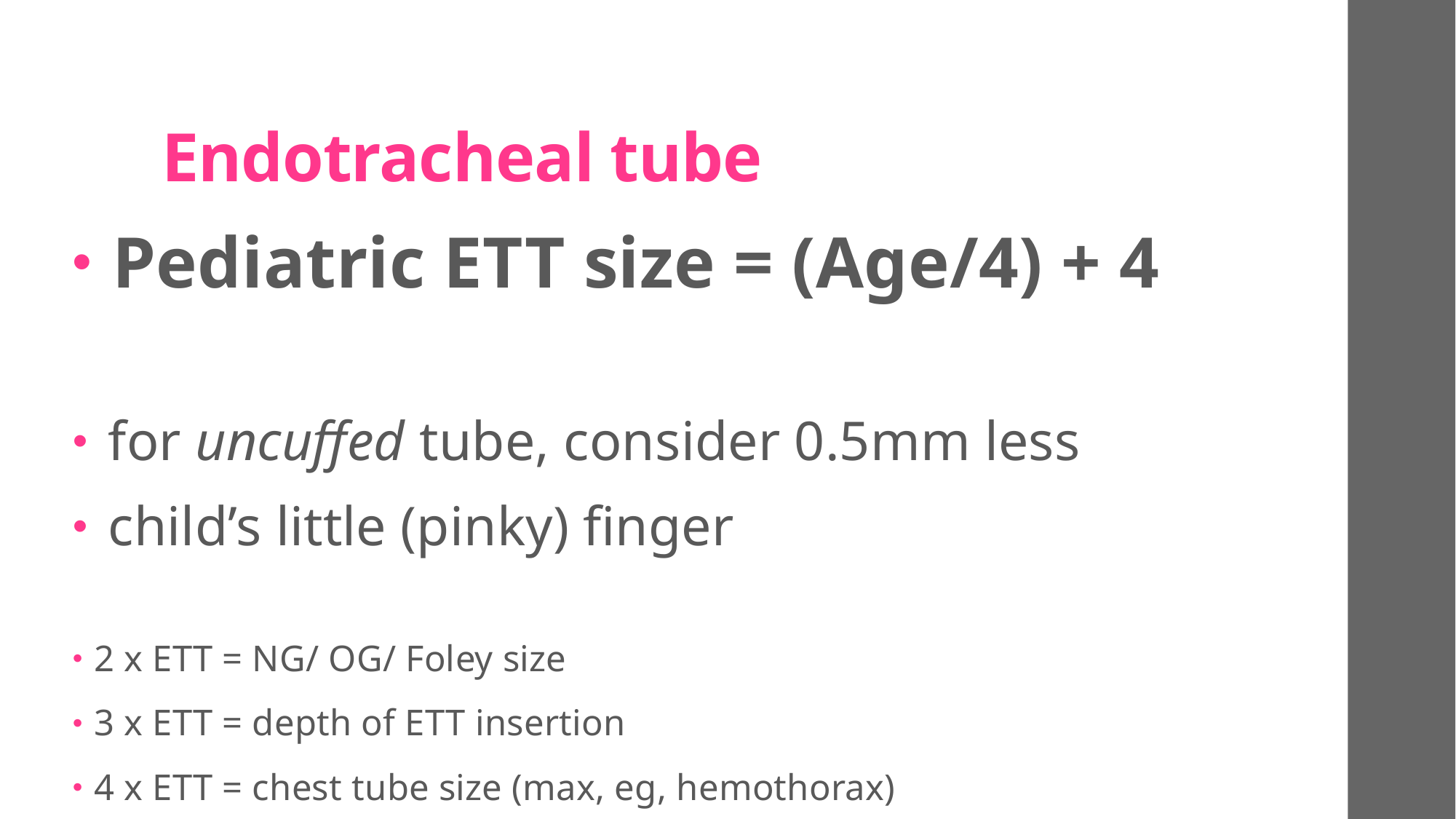

# Endotracheal tube
 Pediatric ETT size = (Age/4) + 4
 for uncuffed tube, consider 0.5mm less
 child’s little (pinky) finger
2 x ETT = NG/ OG/ Foley size
3 x ETT = depth of ETT insertion
4 x ETT = chest tube size (max, eg, hemothorax)

## Slide 36
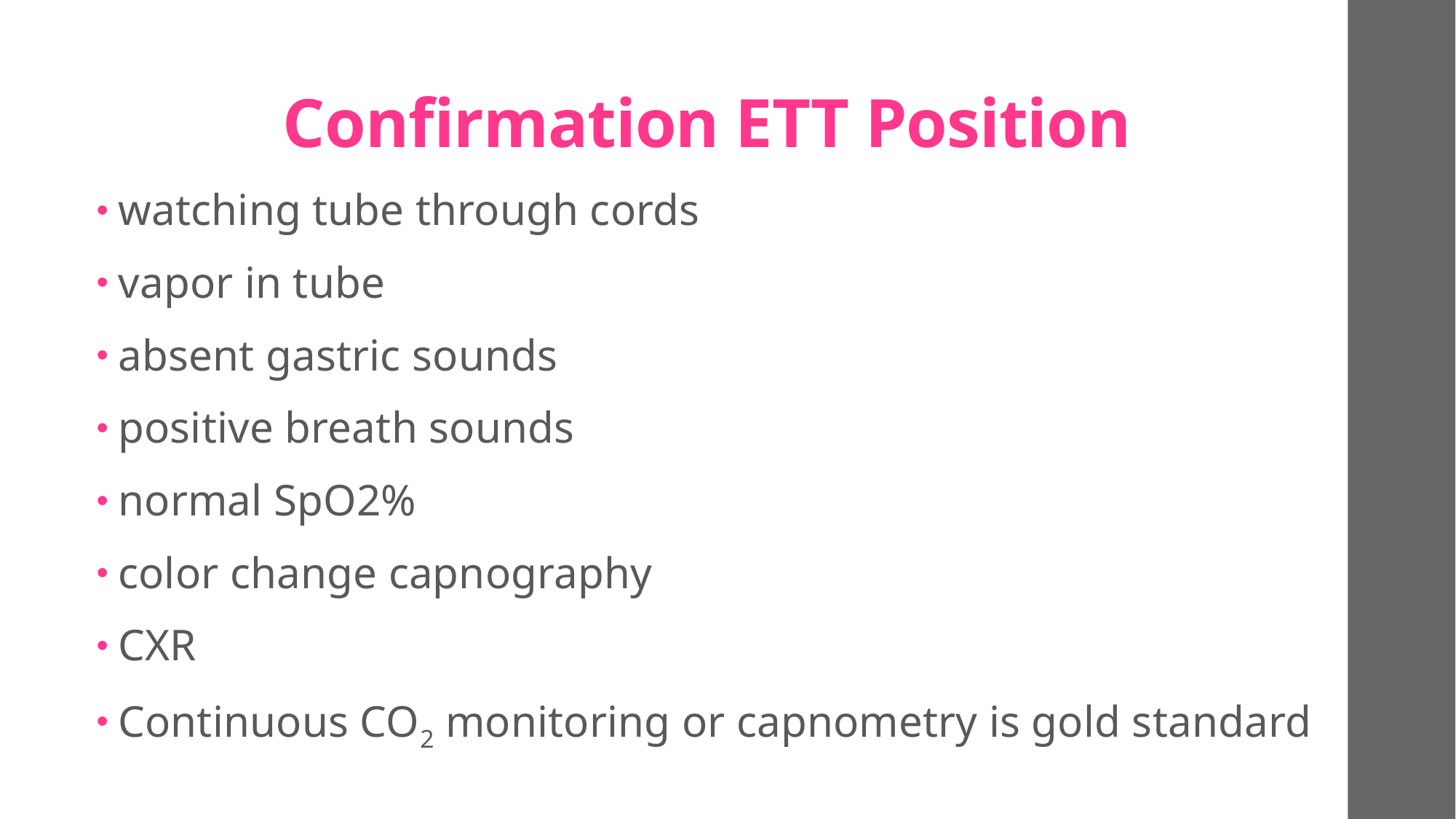

# Confirmation ETT Position
watching tube through cords
vapor in tube
absent gastric sounds
positive breath sounds
normal SpO2%
color change capnography
CXR
Continuous CO2 monitoring or capnometry is gold standard

## Slide 37
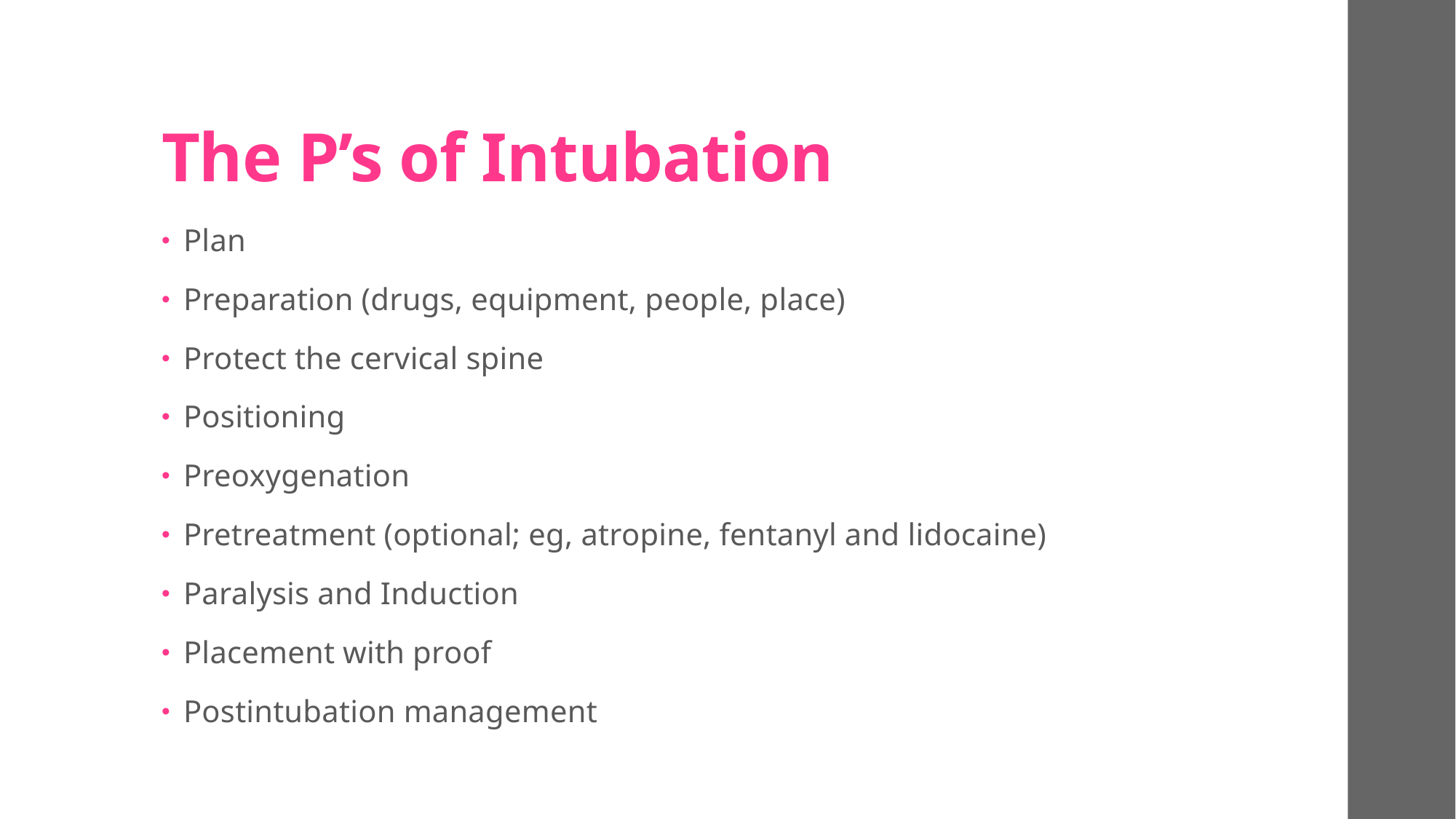

# The P’s of Intubation
Plan
Preparation (drugs, equipment, people, place)
Protect the cervical spine
Positioning
Preoxygenation
Pretreatment (optional; eg, atropine, fentanyl and lidocaine)
Paralysis and Induction
Placement with proof
Postintubation management

## Slide 38
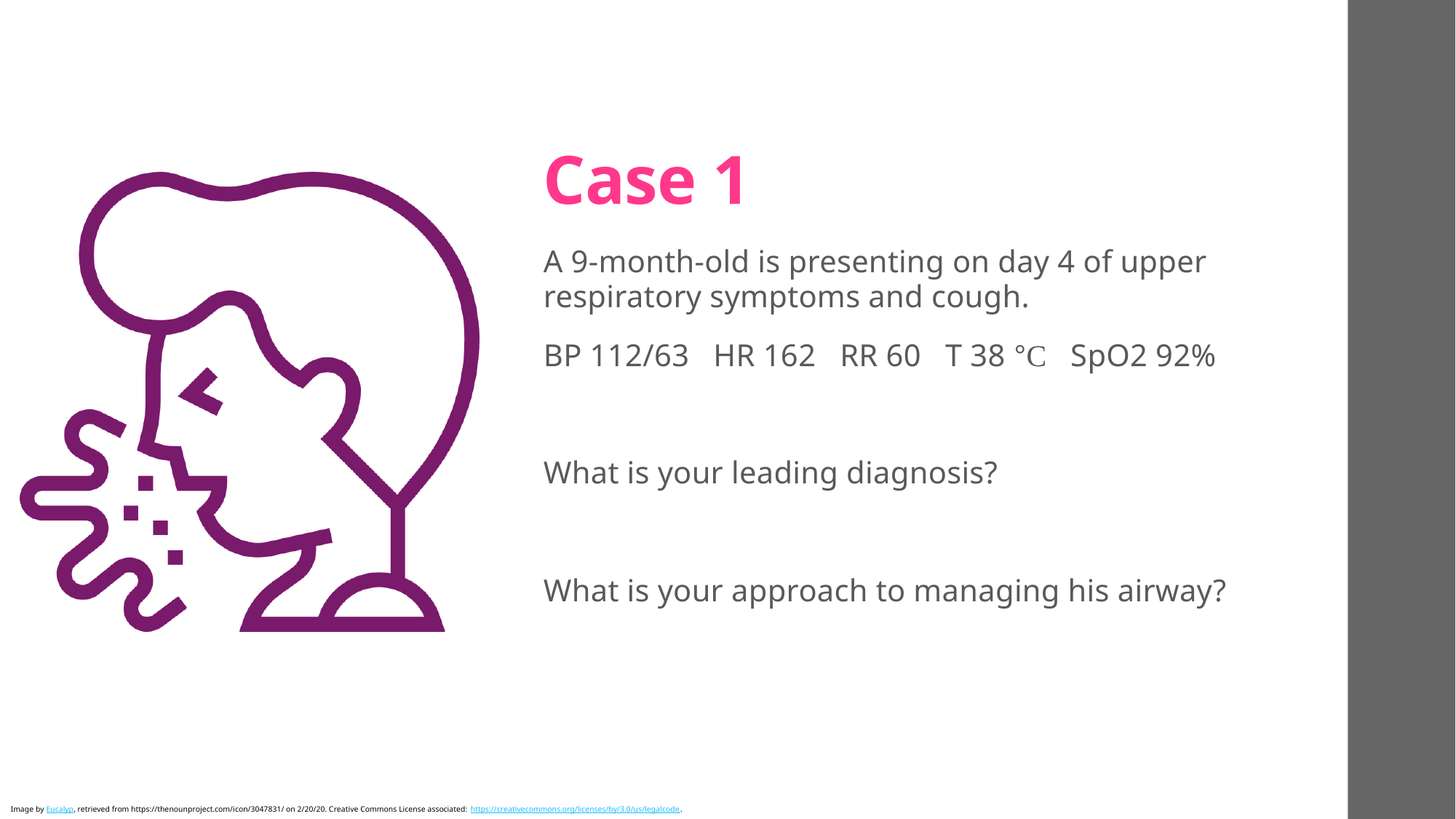

# Case 1
A 9-month-old is presenting on day 4 of upper respiratory symptoms and cough.
BP 112/63 HR 162 RR 60 T 38 °C SpO2 92%
What is your leading diagnosis?
What is your approach to managing his airway?
Image by Eucalyp, retrieved from https://thenounproject.com/icon/3047831/ on 2/20/20. Creative Commons License associated: https://creativecommons.org/licenses/by/3.0/us/legalcode.

## Slide 39
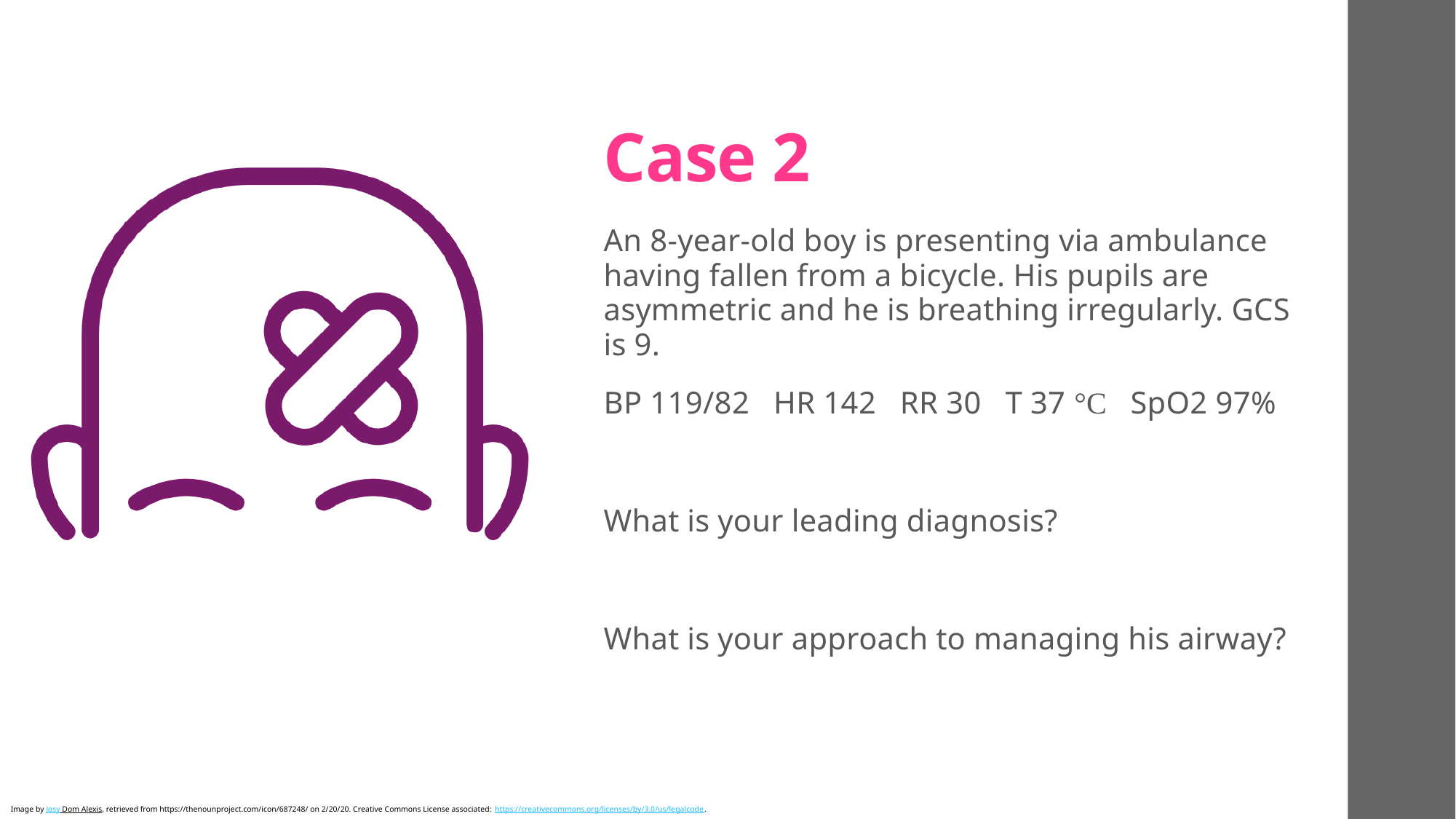

# Case 2
An 8-year-old boy is presenting via ambulance having fallen from a bicycle. His pupils are asymmetric and he is breathing irregularly. GCS is 9.
BP 119/82 HR 142 RR 30 T 37 °C SpO2 97%
What is your leading diagnosis?
What is your approach to managing his airway?
Image by Josy Dom Alexis, retrieved from https://thenounproject.com/icon/687248/ on 2/20/20. Creative Commons License associated: https://creativecommons.org/licenses/by/3.0/us/legalcode.

## Slide 40
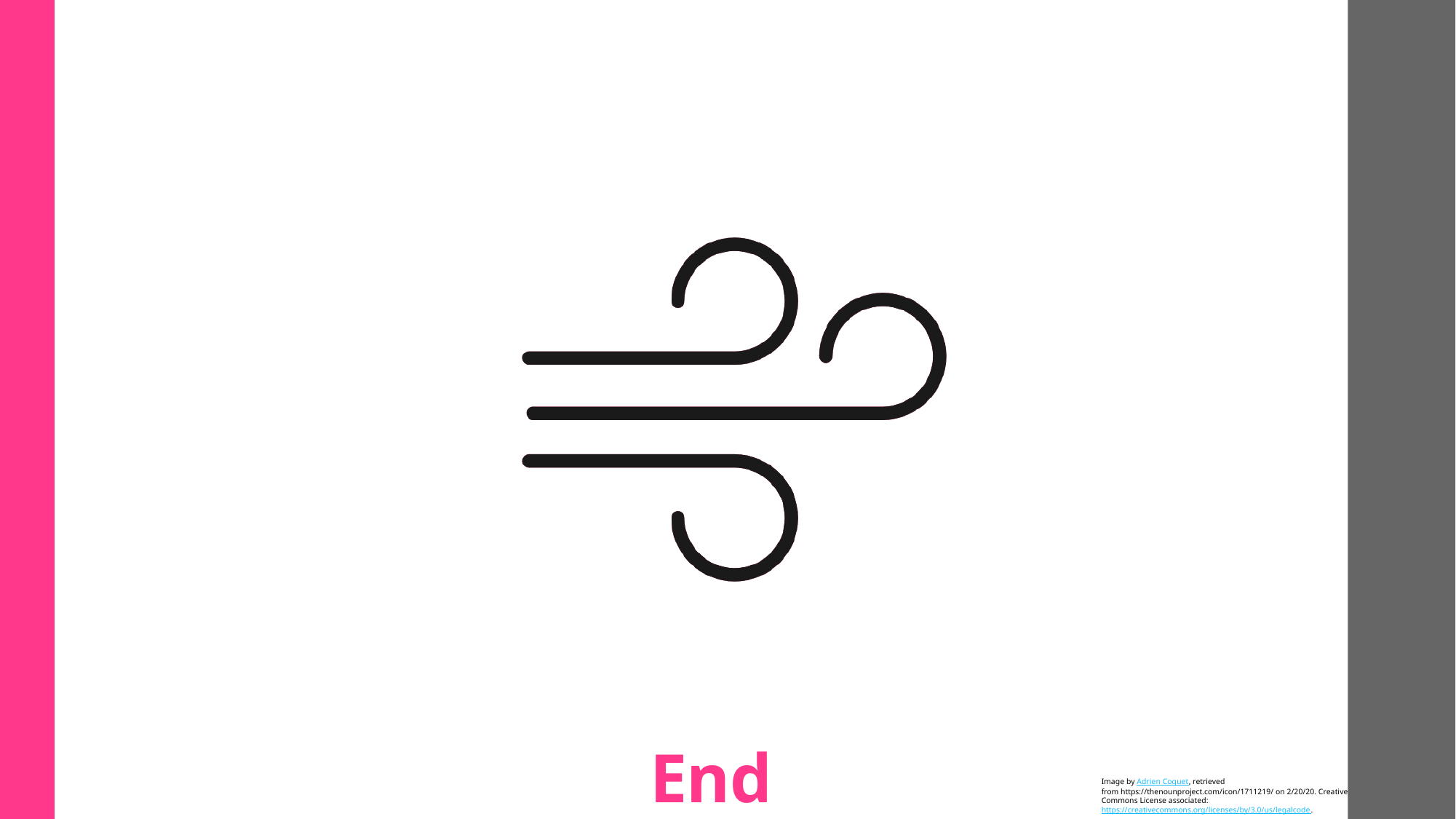

End
Image by Adrien Coquet, retrieved from https://thenounproject.com/icon/1711219/ on 2/20/20. Creative Commons License associated: https://creativecommons.org/licenses/by/3.0/us/legalcode.
