# Supplementary material for: A Pediatric Emergency Medicine Refresher Course for Generalist Healthcare Providers in Belize: Respiratory Emergencies
Source: J Educ Teach Emerg Med. 2021 Apr 19;6(2):C73–C188. doi: 10.21980/J84063 (PMC10332788; doi:10.21980/J84063)
Supplement: Supplementary file 5 — Please see associated PowerPoint file [file jetem-6-2-c73-AppendixR.pptx]

## Slide 1
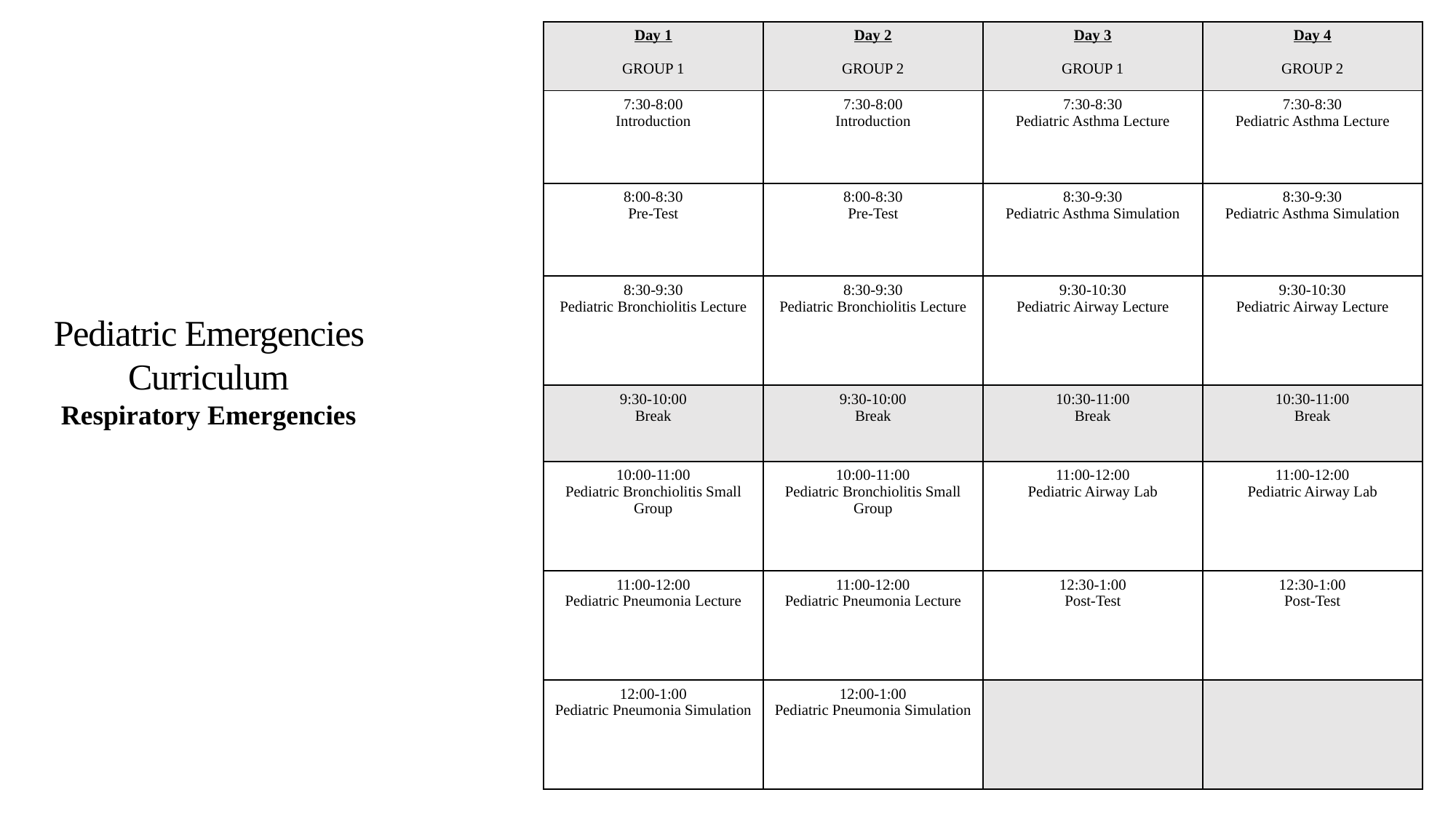

| Day 1 GROUP 1 | Day 2 GROUP 2 | Day 3 GROUP 1 | Day 4 GROUP 2 |
| --- | --- | --- | --- |
| 7:30-8:00 Introduction | 7:30-8:00 Introduction | 7:30-8:30 Pediatric Asthma Lecture | 7:30-8:30 Pediatric Asthma Lecture |
| 8:00-8:30 Pre-Test | 8:00-8:30 Pre-Test | 8:30-9:30 Pediatric Asthma Simulation | 8:30-9:30 Pediatric Asthma Simulation |
| 8:30-9:30 Pediatric Bronchiolitis Lecture | 8:30-9:30 Pediatric Bronchiolitis Lecture | 9:30-10:30 Pediatric Airway Lecture | 9:30-10:30 Pediatric Airway Lecture |
| 9:30-10:00 Break | 9:30-10:00 Break | 10:30-11:00 Break | 10:30-11:00 Break |
| 10:00-11:00 Pediatric Bronchiolitis Small Group | 10:00-11:00 Pediatric Bronchiolitis Small Group | 11:00-12:00 Pediatric Airway Lab | 11:00-12:00 Pediatric Airway Lab |
| 11:00-12:00 Pediatric Pneumonia Lecture | 11:00-12:00 Pediatric Pneumonia Lecture | 12:30-1:00 Post-Test | 12:30-1:00 Post-Test |
| 12:00-1:00 Pediatric Pneumonia Simulation | 12:00-1:00 Pediatric Pneumonia Simulation | | |
Pediatric Emergencies Curriculum
Respiratory Emergencies
